# Regime shift in secondary inorganic aerosol formation and nitrogen deposition in the rural United States

**DOI:** 10.1038/s41561-024-01455-9

**Published:** 2024-06-20

**Authors:** Da Pan, Denise L. Mauzerall, Rui Wang, Xuehui Guo, Melissa Puchalski, Yixin Guo, Shaojie Song, Daniel Tong, Amy P. Sullivan, Bret A. Schichtel, Jeffrey L. Collett, Mark A. Zondlo

**Affiliations:** 1 Department of Civil and Environmental Engineering, Princeton University, Princeton, NJ USA; 2 Department of Atmospheric Science, Colorado State University, Fort Collins, CO USA; 3 Princeton School of Public and International Affairs, Princeton University, Princeton, NJ USA; 4 US Environmental Protection Agency, Office of Air and Radiation, Washington, DC USA; 5 State Environmental Protection Key Laboratory of Urban Ambient Air Particulate Matter Pollution Prevention and Control & Tianjin Key Laboratory of Urban Transport Emission Research, College of Environmental Science and Engineering, Nankai University, Tianjin, China; 6 Atmospheric, Oceanic & Earth Sciences Department and Center for Spatial Information Science and Systems, George Mason University, Fairfax, VA USA; 7 National Park Service, Air Resources Division, Lakewood, CO USA; 8 Cooperative Institute for Research in the Atmosphere, Colorado State University, Fort Collins, CO USA; 9 Present Address: Department of Environmental Sciences, University of Virginia, Charlottesville, VA USA; 10 Present Address: Department of Atmospheric and Oceanic Sciences, Peking University, Beijing, China

**Keywords:** Environmental sciences, Biogeochemistry, Atmospheric chemistry

## Abstract

Secondary inorganic aerosols play an important role in air pollution and climate change, and their formation modulates the atmospheric deposition of reactive nitrogen (including oxidized and reduced nitrogen), thus impacting the nitrogen cycle. Large-scale and long-term analyses of secondary inorganic aerosol formation based on model simulations have substantial uncertainties. Here we improve constraints on secondary inorganic aerosol formation using decade-long in situ observations of aerosol composition and gaseous precursors from multiple monitoring networks across the United States. We reveal a shift in the secondary inorganic aerosol formation regime in the rural United States between 2011 and 2020, making rural areas less sensitive to changes in ammonia concentrations and shortening the effective atmospheric lifetime of reduced forms of reactive nitrogen. This leads to potential increases in reactive nitrogen deposition near ammonia emission hotspots, with ecosystem impacts warranting further investigation. Ammonia (NH_3_), a critical but not directly regulated precursor of fine particulate matter in the United States, has been increasingly scrutinized to improve air quality. Our findings, however, show that controlling NH_3_ became significantly less effective for mitigating fine particulate matter in the rural United States. We highlight the need for more collocated aerosol and precursor observations for better characterization of secondary inorganic aerosols formation in urban areas.

## Abstract (web summary)

Chemical regimes of atmospheric secondary inorganic aerosol formation in rural areas of the United States shifted from NH_3_-sensitive to NH_3_-insensitive between 2011 and 2020, according to analyses of long-term observational data on aerosol composition and gaseous precursors.

## Main

Secondary inorganic aerosols (SIAs) are major components of fine particulate matter (PM_2.5_), which has detrimental impacts on human health and regional visibility and substantially influences the radiative balance of the climate system^1–4^. SIAs are formed predominantly through the oxidation of sulfur dioxide (SO_2_) and nitrogen oxides (NO_*x*_), and subsequent reaction with ammonia (NH_3_)^5^. These processes determine the physical and chemical properties of aerosols, including aerosol acidity, aerosol water uptake and growth, and potentially aerosol toxicity. SIA formation also influences the gas–particle partitioning of semivolatile inorganic reactive nitrogen (N_r_) species, such as NH_3_, ammonium (NH_4_^+^), nitric acid (HNO_3_) and nitrate (NO_3_^−^)^5^. Because gaseous NH_3_ and HNO_3_ species deposit much more quickly than N_r_ compounds in PM_2.5_ (refs. ^6,7^), their phase partitioning modulates the spatial distribution of N_r_ atmospheric deposition, which influences human exposure to PM_2.5_ (and the associated health impacts), loss of biological diversity, soil and water acidification, and surface water eutrophication^8–11^. Therefore, a better understanding of SIA formation can facilitate policy-making in relation to many environmental challenges.

Aerosol thermodynamic analyses using measured gas concentrations and particle composition provide better constraints on SIA formation and the partitioning of semivolatile species than simulations with chemical transport models (CTMs)^12^. Compared to observations, regional and global CTM simulations vary substantially in terms of the simulated aerosol composition and phase partitioning of N_r_ species in the United States^13–16^ (Extended Data Table 1). This variability could result from uncertainties in emission inventories, transport, dry deposition, wet scavenging and/or heterogeneous chemical production^13,14,17–20^. Directly modelling SIA formation with simultaneous measurements of gas concentrations and aerosol composition (that is, concentrations of NH_3_, HNO_3_, NH_4_^+^, NO_3_^−^, SO_4_^2−^, non-volatile cations (NVCs, including sodium, calcium, magnesium and potassium ions) and chloride ion (Cl^−^)) avoids the aforementioned uncertainties^12,21^. However, this is only available at a few sites or from a few intensive field campaigns with limited spatiotemporal coverage in the United States^12,22,23^. Moreover, past measurements are unlikely to reflect the current atmospheric composition due to rapid changes in the emissions of various precursors, impacts on gas–particle partitioning from climate change, and increases in the size and number of wildfires.

In this Article we overcome the above limitations of existing datasets and a lack of constraint on simulated SIA formation by using observations from multiple long-term air-quality-monitoring networks for aerosol thermodynamic analyses. Our results show that chemical regimes of SIA formation in the rural United States shifted from NH_3_-sensitive to NH_3_-insensitive between 2011 and 2020 and led to increases in N_r_ deposition near NH_3_-emission hotspots. Although we focus on the rural United States because of the available observations, we demonstrate the benefits of collocated monitoring for aerosol composition and precursor concentrations, which should be considered for future monitoring network design in the United States and globally.

## Improving constraints on SIA formation

**Extended Data Fig. 1:**
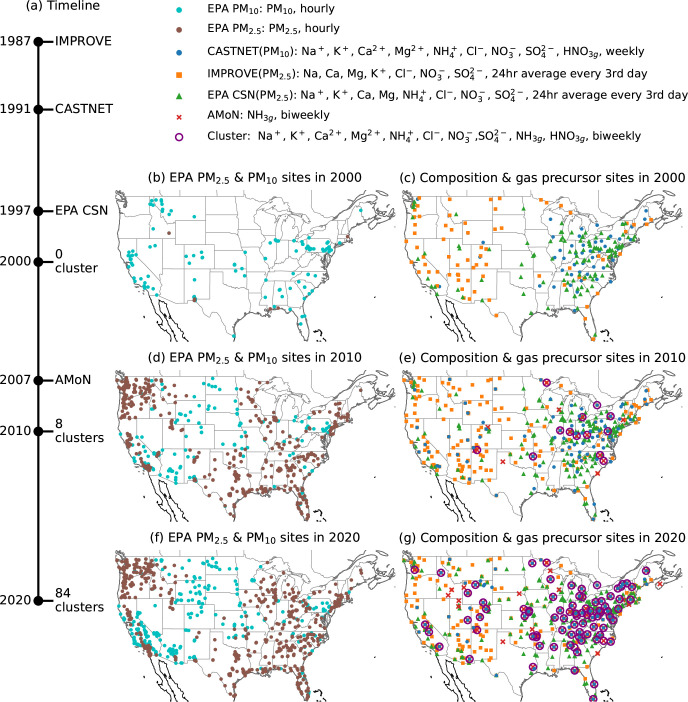
Development of air quality monitoring networks in the U.S. and locations of the monitoring networks. (**a**) Development of air quality monitoring networks in the U.S. and (**b–g**) locations of the monitoring networks. The base map is obtained from Natural Earth.

We identified locations where sites from the monitoring networks provide essential inputs to SIA formation simulations and are located within a spatial window of 50 km (Methods). Several national networks monitor trace-gas precursors and aerosol chemical composition, but observations from an individual network are insufficient for thermodynamic modelling. Integrating collocated observations provides the inputs needed as biweekly means (averaged every 2 weeks). There were 42 and 68 locations that had collocated observations for the periods of 2011–2015 and 2016–2020, respectively (Extended Data Fig. 1 and Supplementary Tables 1 and 2). Although these areas are located outside urban centres, many of them are still in the vicinity of high-population areas, especially in the Midwestern and Northeastern United States. The areas within 50 km of the locations account for 6.7% of the land surface areas, but 9.8%, 7.0%, 8.7% and 7.5% of the population, SO_2_ emissions, NO_*x*_ emissions and NH_3_ emissions in the contiguous United States, respectively^24,25^. Moreover, because the aerosol composition and precursors observed at sites 50–100 km apart still show good agreement (Supplementary Fig. 1), our findings may apply to rural and suburban regions outside major urban centres more generally.

**Extended Data Fig. 2:**
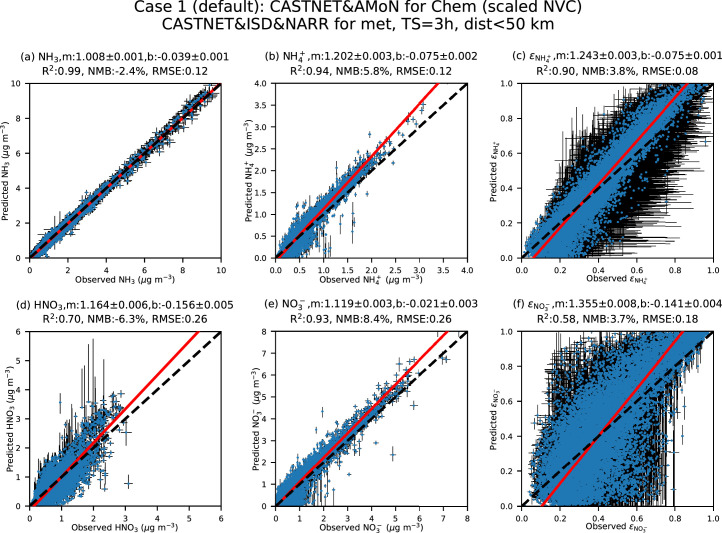
Observed and ISORROPIA-II simulated ${\mathbf{c}}_{{{\mathbf{NH}}}_{\mathbf{3}}}$, ${\mathbf{c}}_{{{\mathbf{NH}}}_{\mathbf{4}}^{\mathbf{+}}}$, ${\mathbf{\varepsilon }}_{{{\mathbf{NH}}}_{\mathbf{4}}^{\mathbf{+}}}$, ${\mathbf{c}}_{{{\mathbf{HNO}}}_{\mathbf{3}}}$, ${\mathbf{c}}_{{{\mathbf{NO}}}_{\mathbf{3}}^{\mathbf{-}}}$, and ${\mathbf{\varepsilon }}_{{{\mathbf{NO}}}_{\mathbf{3}}^{\mathbf{-}}}$. The dots and error bars represent the mean values and the 95% confidence intervals (CI; as the 2.5^th^ and the 97.5^th^ percentiles) of 1000 Monte Carlo simulations, respectively. Red lines show orthogonal distance regression results (prediction = m·observation + b), and corresponding regression parameters and evaluation statistics (determination coefficient (*R*^2^), normalized mean bias (NMB), and root mean square error (RMSE)) are shown in panel titles. Black dashed lines show the 1:1 line. The number of samples is 13813 for all panels.

Using the ISORROPIA-II model^26^ (a full thermodynamic model for inorganic aerosol formation) with the integrated dataset described above, we substaintially reduce uncertainties in simulating SIA formation (Extended Data Fig. 2). Although ISORROPIA-II and other aerosol thermodynamic models have been validated with hourly or daily observations^12,27^, they have not been validated with biweekly observations made with different sampling methods. We conducted sensitivity tests and uncertainty analyses to develop the necessary preprocessing steps to integrate collocated observations (Methods and Supplementary Table 3), reducing the normalized mean biases (NMBs) between simulated and observed ${c}_{{{\rm{NH}}}_{3}}$, ${c}_{{\rm{N}}{{\rm{H}}}_{4}^{+}}$, ${c}_{{\rm{HN}}{{\rm{O}}}_{3}}$ and ${c}_{{\rm{N}}{{\rm{O}}}_{3}^{-}}$ (where *c* denotes concentration, in units of μg per m^3^ of air) from −28 to 11% to −6 to 8% (Supplementary Table 3). The NMBs between CTM simulations and observations are much larger (−65 to 126%) because the built-in aerosol thermodynamic model is driven by inputs determined by emission, oxidation, transport and deposition processes^13–20^. Although simulating these processes links the concentrations of SIA precursors (for example, SO_4_^2−^, total nitrate (NO_3_^T^ = HNO_3_ + NO_3_^−^) and total ammonium (NH_4_^T^ = NH_3_ + NH_4_^+^)) to primary emissions, the large errors in CTMs could alter the SIA formation regime, and observations are needed to constrain these processes. Here, we first investigate regional precursor concentration responses to emission reductions by examining the relationship between precursor concentrations and their emissions. Then, with the improved constraints on SIA formation, we can better quantify the impacts of rapidly changing atmospheric composition on N_r_ deposition, SIA properties and SIA sensitivities to precursor reductions.

## Rapid changes in aerosol composition and acidity

**Fig. 1:**
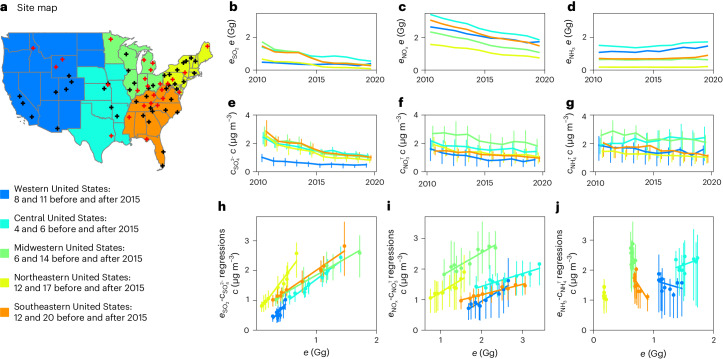
Site locations and relationships between emissions of SO_2_, NO_*x*_ and NH_3_ and concentrations of SO_4_^2−^, NO_3_^T^ and NH_4_^T^. **a**, Site map. Black and red crosses represent measurement sites established before and after 2015, respectively, in the five regions indicated by specific colours. Corresponding site numbers are listed in the legends. The base map was obtained from Natural Earth. The five regions are defined according to the Regional Planning Organizations (Methods). The numbers of samples for these regions for each year are listed in Supplementary Table 2. **b**–**d**, Annual SO_2_ (**b**), NO_*x*_ (**c**) and NH_3_ (**d**) emissions (${e}_{{\rm{S}}{{\rm{O}}}_{2}}$, ${e}_{{\rm{N}}{{\rm{O}}}_{{{x}}}}$ and ${e}_{{\rm{N}}{{\rm{H}}}_{3}}$) in the five regions. **e**–**g**, Annual mean concentrations of SO_4_^2−^ (**e**), NO_3_^T^ (**f**) and NH_4_^T^ (**g**). **h**–**j**, Orthogonal distance regressions of annual mean ${e}_{{\rm{S}}{{\rm{O}}}_{2}}$ and ${c}_{{\rm{S}}{{\rm{O}}}_{4}^{2-}}$ (**h**), ${e}_{{\rm{N}}{{\rm{O}}}_{{{x}}}}$ and ${c}_{{\rm{N}}{{\rm{O}}}_{3}^{{\rm{T}}}}$ (**i**) and ${e}_{{\rm{N}}{{\rm{H}}}_{3}}$ and ${c}_{{\rm{N}}{{\rm{H}}}_{4}^{{\rm{T}}}}$ (**j**), with each dot indicating one year from 2011 to 2020. The vertical bars show the 25th and 75th percentiles of annual mean concentrations observed at locations within a region.

Between 2011 and 2020, all regions in the United States experienced significant decreases in ${c}_{{\rm{S}}{{\rm{O}}}_{4}^{2-}}$ and ${c}_{{\rm{N}}{{\rm{O}}}_{3}^{{\rm{T}}}}$ (Fig. 1e,f), whereas ${c}_{{\rm{N}}{{\rm{H}}}_{4}^{{\rm{T}}}}$ remained relatively stable in the Western and Midwestern United States but decreased in the Central, Northeastern and Southeastern United States (Fig. 1g and Supplementary Table 4). Concentrations of organic aerosols (OAs) also remained relatively stable during this period, except in the Western United States (Supplementary Fig. 2). Their relative contributions to PM_2.5_ concentrations increased significantly because of the reductions in *c*_SIA_ (Supplementary Fig. 2). Annual concentrations of SIAs were still higher than OAs at the locations investigated in the Midwestern, Northeastern and Southeastern United States in 2020.

**Extended Data Fig. 3:**
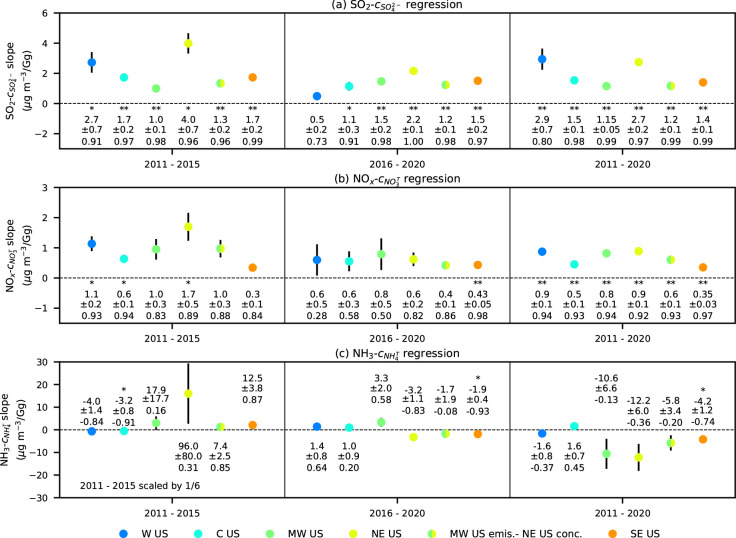
Relationships between emissions and regional mean concentrations. Panels (**a**–**c**) present the orthogonal distance regression slopes of (**a**) SO_2_ emission**-**${c}_{{{\rm{SO}}}_{4}^{2-}}$, (**b**) NO_x_ emissions**-**${c}_{{{\rm{NO}}}_{3}^{{\rm{T}}}}$, and (**c**) NH_3_ emission**-**${c}_{{{\rm{NH}}}_{4}^{{\rm{T}}}}$ regressions for 2011–2015, 2016–2020, and 2011–2020 with observations from long-term sites only. Regional mean concentrations are used in the regressions, and the sample sizes for each region are 5, 5, and 10 for 2011–2015, 2016–2020, and 2011–2020, respectively. ‘^*^’ or ‘^**^’ indicate the regression has a p < 0.05 or <0.01, respectively. The error bars show uncertainties of the regression slopes (95% CI; calculated as ±1.96 standard deviation (SD)). The numbers below are the slopes (mean values), the uncertainties (±1.96 SD), and the Pearson correlation coefficients, respectively. To illustrate NH_3_-${c}_{{{\rm{NH}}}_{4}^{{\rm{T}}}}$ correlation results for 2011–2015 in panel (c), they are scaled by 1/6.

As a consequence of regulations, shifts in energy generation, and implementations of emission control technology, ${e}_{{\rm{S}}{{\rm{O}}}_{2}}$ and ${e}_{{\rm{N}}{{\rm{O}}}_{{{x}}}}$ (Fig. 1b,c; *e* denotes emission in units of Gg) decreased, respectively, by 70% and 50% in the United States between 2011 and 2020^28^. The decreases in ${c}_{{\rm{S}}{{\rm{O}}}_{4}^{2-}}$ and ${c}_{{\rm{N}}{{\rm{O}}}_{3}^{{\rm{T}}}}$ correlate with these emission reductions (Fig. 1h,i), indicating that ${e}_{{\rm{S}}{{\rm{O}}}_{2}}$ and ${e}_{{\rm{N}}{{\rm{O}}}_{{{x}}}}$ reductions have been very effective in reducing ${c}_{{\rm{S}}{{\rm{O}}}_{4}^{2-}}$ and ${c}_{{\rm{N}}{{\rm{O}}}_{3}^{{\rm{T}}}}$. The responses of ${c}_{{\rm{S}}{{\rm{O}}}_{4}^{2-}}$ and ${c}_{{\rm{N}}{{\rm{O}}}_{3}^{{\rm{T}}}}$ to ${e}_{{\rm{S}}{{\rm{O}}}_{2}}$ and ${e}_{{\rm{N}}{{\rm{O}}}_{{{x}}}}$ reductions remained largely unchanged between 2000 and 2021^29^, and this period witnessed 90% and 65% reductions in ${e}_{{\rm{S}}{{\rm{O}}}_{2}}$ and ${e}_{{\rm{N}}{{\rm{O}}}_{{{x}}}}$, respectively^28^. However, the responses could change if SO_2_ and NO_*x*_ emission reductions continue (Supplementary Text 1). In contrast, ${e}_{{\rm{N}}{{\rm{H}}}_{3}}$ has not been directly regulated and remained approximately unchanged. ${c}_{{\rm{N}}{{\rm{H}}}_{4}^{{\rm{T}}}}$ and ${e}_{{\rm{N}}{{\rm{H}}}_{3}}$ are inversely correlated in the Southeastern United States and show no clear correlation in other regions (Extended Data Fig. 3). Regional Kendall tests show that these trends remain consistent with or without the sites established after 2015 (Supplementary Fig. 3 and Supplementary Table 4)^30^. More trend analyses and regression results are presented in Supplementary Figs. 4–6 and Supplementary Text 1. The inverse correlations and less clear ${c}_{{\rm{N}}{{\rm{H}}}_{4}^{{\rm{T}}}}-{e}_{{\rm{N}}{{\rm{H}}}_{3}}$ relationship reflect large uncertainties in NH_3_ emissions and/or increased NH_4_^T^ removal associated with ${c}_{{\rm{N}}{{\rm{O}}}_{3}^{{\rm{T}}}}$ and ${c}_{{\rm{S}}{{\rm{O}}}_{4}^{2-}}$ reductions instead of changes in ${e}_{{\rm{N}}{{\rm{H}}}_{3}}$.

**Fig. 2:**
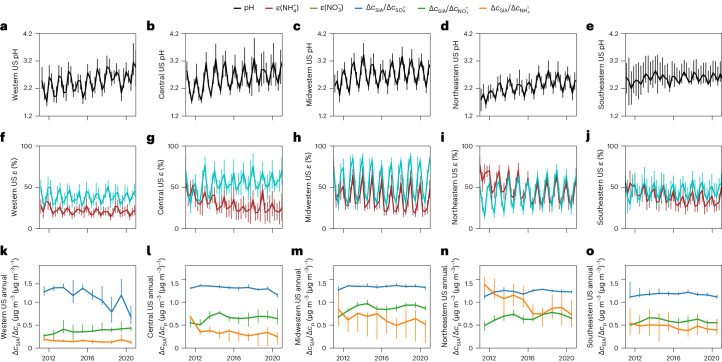
Regional means of aerosol pH, gas–particle partitioning and *c*_SIA_ sensitivities to precursor reductions (Δ*c*_SIA_/Δ*c*_*p*_) from 2011 to 2020. The numbers of samples used to calculate the mean values for each region are listed in Supplementary Table 2. **a**–**e**, Simulated aerosol pH (black lines) in the Western (**a**), Central (**b**), Midwestern (**c**), Northeastern (**d**) and Southeastern (**e**) United States over time. **f**–**j**, Observed molar fractions *ε* of NO_3_^T^ (cyan) and NH_4_^T^ (brown) that partition into the particle phase (${\varepsilon }_{{\rm{N}}{{\rm{O}}}_{3}^{-}}$ and ${\varepsilon }_{{\rm{N}}{{\rm{H}}}_{4}^{+}}$) in the Western (**f**), Central (**g**), Midwestern (**h**), Northeastern (**i**) and Southeastern (**j**) United States over time. **k**–**o**, ${\Delta {c}_{{\rm{SIA}}}/\Delta {c}_{{\rm{S}}{{\rm{O}}}_{4}^{2-}}}$ (blue), ${\Delta {c}_{{\rm{SIA}}}/\Delta {c}_{{\rm{N}}{{\rm{O}}}_{3}^{{\rm{T}}}}}$ (green) and ${\Delta {c}_{{\rm{SIA}}}/\Delta {c}_{{\rm{N}}{{\rm{H}}}_{4}^{{\rm{T}}}}}$ (orange), simulated by reducing the corresponding precursors by 40% in the Western (**k**), Central (**l**), Midwestern (**m**), Northeastern (**n**) and Southeastern (**o**) United States over time. Vertical bars show the 25th and 75th percentiles of the corresponding values observed or simulated at locations within a region to illustrate regional variability.

**Extended Data Fig. 4:**
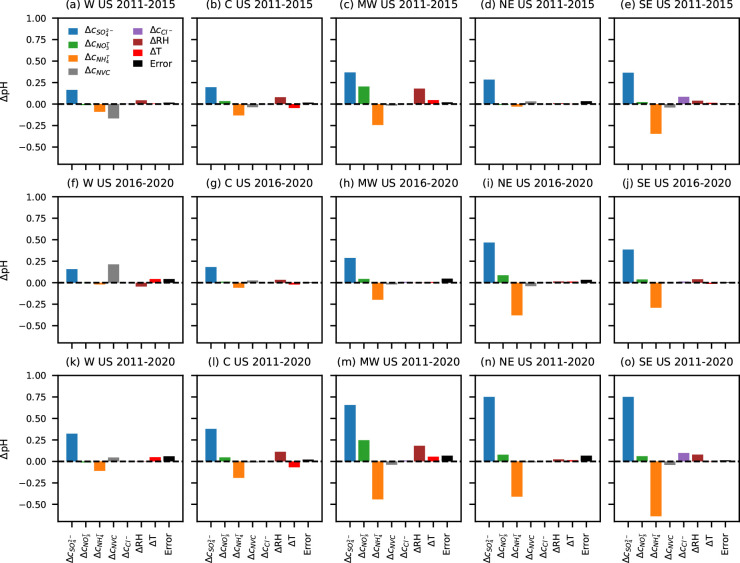
Contributions of changes in ${\mathbf{c}}_{{{\mathbf{SO}}}_{\mathbf{4}}^{\mathbf{2-}}}$, ${\mathbf{c}}_{{{\mathbf{NO}}}_{\mathbf{3}}^{{\mathbf{T}}}}$, ${\mathbf{c}}_{{{\mathbf{NH}}}_{\mathbf{4}}^{{\mathbf{T}}}}$, ${\mathbf{c}}_{{\mathbf{NVC}}}$, ${\mathbf{c}}_{{{\mathbf{Cl}}}^{\mathbf{-}}}$, RH, and T to pH changes. The contributions are cumulative contributions calculated as described in the Methods section (Eq. (2) and (3)).

**Extended Data Fig. 5:**
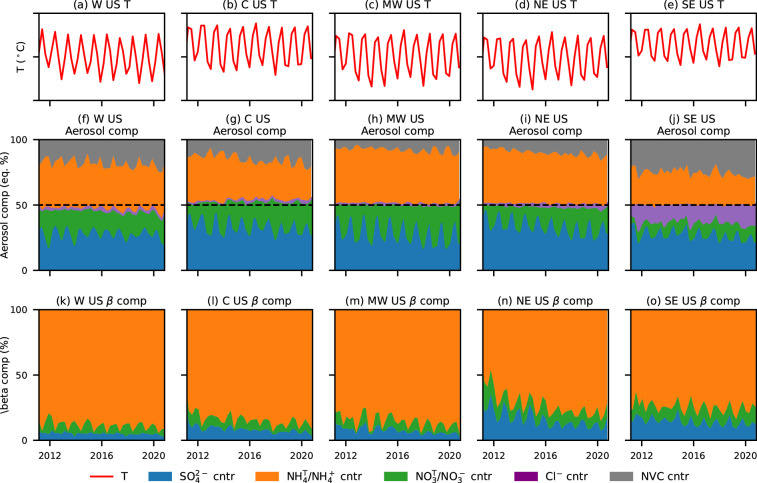
Regional means of temperature, aerosol composition, and pH buffering capacity composition. Regional means of temperature, aerosol composition (calculated using ion-equivalent concentrations to reflect aerosol charge balance), and pH buffering capacity composition from 2011 to 2020. Blue, green, and orange areas in panels (f–o) show contributions (cntr) of SO_4_^2−^, NO_3_^T^, and NH_4_^T^ to aerosol composition or aerosol pH buffering capacity. Grey and purple areas show contributions of non-volatile cations (NVC) and chloride ion (Cl^−^) to aerosol composition.

Influencing aerosol thermodynamic properties, aerosol acidity is a key indicator of potential changes in gas–particle partitioning and SIA formation caused by changes in aerosol composition^31^. Aerosol pH is difficult to measure directly, and is often estimated using aerosol thermodynamic simulations because of the challenges associated with collecting unperturbed samples^31^. Between 2011 and 2020, our simulations show that the annual mean aerosol pH increased by 0.2–0.6 units across the rural United States (Fig. 2a–e). The major contributor to the pH increase was a reduction in ${c}_{{\rm{S}}{{\rm{O}}}_{4}^{2-}}$ (Extended Data Fig. 4) in all regions, and decreases in ${c}_{{\rm{N}}{{\rm{H}}}_{4}^{{\rm{T}}}}$ ameliorated the extent of the pH increases in the Midwestern, Northeastern and Southeastern United States. Aerosol pH was primarily buffered by NH_3_ in the Western, Central and Midwestern United States (Extended Data Fig. 5). Zheng and colleagues^32^ have shown that this buffering regime suppresses the influence of compositional differences on aerosol pH and makes aerosol water content (AWC) and temperature (T) the primary determinants of aerosol pH, leading to larger seasonal variations in aerosol pH in those three regions. The changes in aerosol acidity and its seasonal variations could have implications for aerosol toxicity and the oxidation rates of SO_2_ and NO_*x*_, which requires further investigation. For example, the effectiveness of controlling SO_2_ emissions on reducing ${c}_{{\rm{S}}{{\rm{O}}}_{4}^{2-}}$ could decrease due to enhanced SO_2_ oxidation as aerosol pH increases^17,31,33^.

## Regime changes in SIA formation and N_r_ deposition

Increases in aerosol pH led to decreases of −2 to −4% per year in the molar fraction of NH_4_^+^ in NH_4_^T^ (${\varepsilon }_{{\rm{N}}{{\rm{H}}}_{4}^{+}}$) in all regions (Fig. 2f–j provides a time series and Supplementary Table 4 shows the trends), implying that more NH_4_^T^ remained as NH_3_ in the atmosphere in 2020 than in 2011. Thus, a greater fraction of NH_4_^T^ could deposit near emission sources as NH_3_, because gas-phase NH_3_ deposits more rapidly than PM_2.5_ (ref. ^7^). The decrease in the atmospheric lifetime of NH_4_^T^ could reduce the NH_4_^T^ transported from NH_3_ sources in the Western, Central and Midwestern United States to the Northeastern and Southeastern United States, explaining the decreasing trends of ${c}_{{\rm{N}}{{\rm{H}}}_{4}^{{\rm{T}}}}$ in the Northeastern and Southeastern United States without significant ${e}_{{\rm{N}}{{\rm{H}}}_{3}}$ changes.

**Fig. 3:**
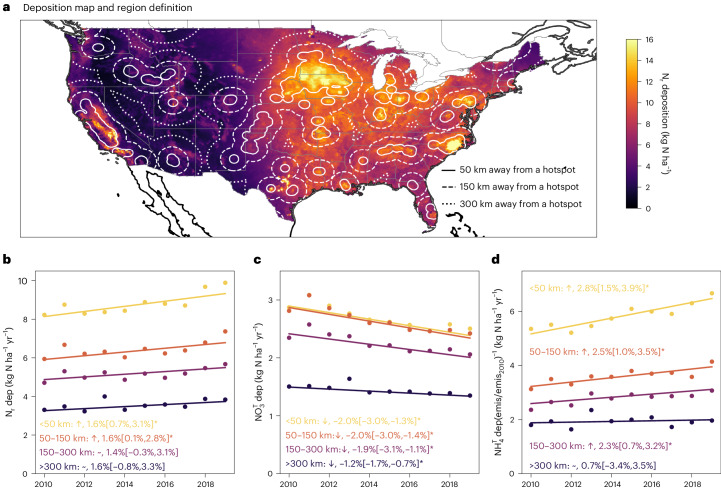
Spatial distribution and trends of total reactive nitrogen and NH_4_^T^ deposition. **a**, The average annual total reactive nitrogen (N_r_) deposition (dep) in the United States between 2010 and 2019. Solid, dashed and dotted lines show the boundaries of the areas within 50 km, 150 km and 300 km of an NH_3_-emission hotspot (Supplementary Text 2). The base map was obtained from Natural Earth. **b**–**d**, The 2010–2019 trends of annual total N_r_ deposition (**b**), NO_3_^T^ deposition (**c**) and NH_4_^T^ deposition normalized by NH_3_ emission (emis) (**d**) trends relative to the 2010 level (emis_2010_). The trends and relative annual change rates are determined using the Mann–Kendall test and Theil–Sen regression with a sample size of 10 (ref. ^50^). Numbers in the brackets are the 95% confidence intervals of the regressions (mean ± 1.96 s.d.). ‘↑’, ‘↓’ and ‘~’ indicate increasing trend, decreasing trend and no trend, respectively. *Statistically significant trend with *P* < 0.05 based on the Mann–Kendall test.

**Extended Data Fig. 6:**
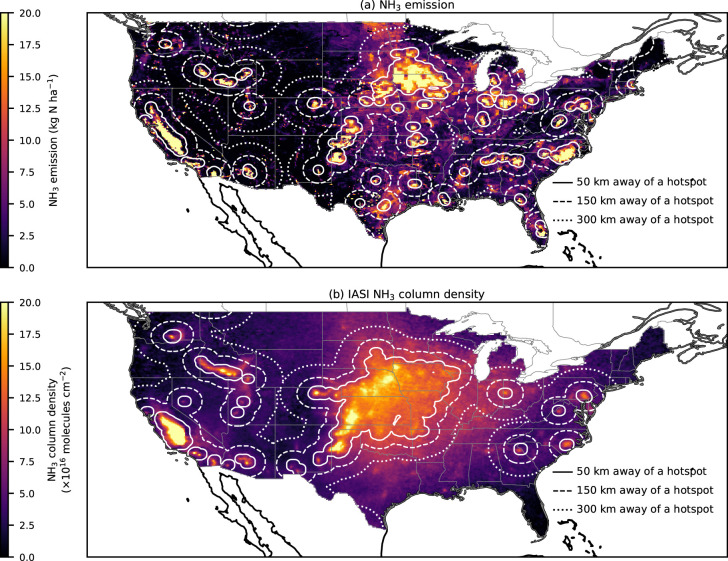
Differences in ammonia emission hotspots and satellite observed ammonia hotspots. **Differences in NH**_**3**_ hotspots defined based on the 2017 emission inventory and satellite observations. (**a**) NH_3_ emissions and (**b**) NH_3_ column densities from the Infrared Atmospheric Sounding Interferometer (IASI). IASI NH_3_ column densities are derived from observations between 2008–2017 (IASI v2.2R)^37^. Solid, dashed, and dotted lines show the boundaries of the areas <50 km, 50–150 km, 150–300 km within an NH_3_ emission hotspot in panel (a) or an NH_3_ hotspot in panel (b). NH_3_ emission hotspots in panel (a) are the areas of the areas of the 95^th^ or high NH_3_ emission rates in 2017 in the Contiguous US. NH_3_ hotspots in panel (b) are the areas of the 95^th^ percentile NH_3_ column density in the Contiguous US. The base map is obtained from Natural Earth.

By dividing the contiguous United States into four zones according to their distances to the nearest NH_3_-emission hotspot (<50 km, 50–150 km, 150–300 km and >300 km), we analysed the trend of annual N_r_ total deposition from the ‘Total Deposition Estimates Using the Measurement Model Fusion’ (TDep MMF)^34^ model between 2010 and 2019 (Fig. 3a and Supplementary Text 2). N_r_ total deposition showed statistically significant increasing trends in areas within 150 km of an NH_3_-emission hotspot (Fig. 3b) and insignificant trends at >150 km from these hotspots, despite reductions in NO_3_^T^ deposition (Fig. 3c). NH_4_^T^ deposition increased more quickly than NH_3_ emissions in the corresponding zones (Fig. 3d). These results are indicative of increased NH_4_^T^ near the source and probably the results of decreased ${\varepsilon }_{{\rm{N}}{{\rm{H}}}_{4}^{+}}$ and higher dry deposition rates of NH_3_ relative to NH_4_^+^. There are large discrepancies between the hotspots defined by NH_3_ emissions and those identified by satellite observations^35–37^ (Extended Data Fig. 6 and Supplementary Figs. 7 and 8), highlighting the need for more NH_3_ observations.

**Extended Data Fig. 7:**
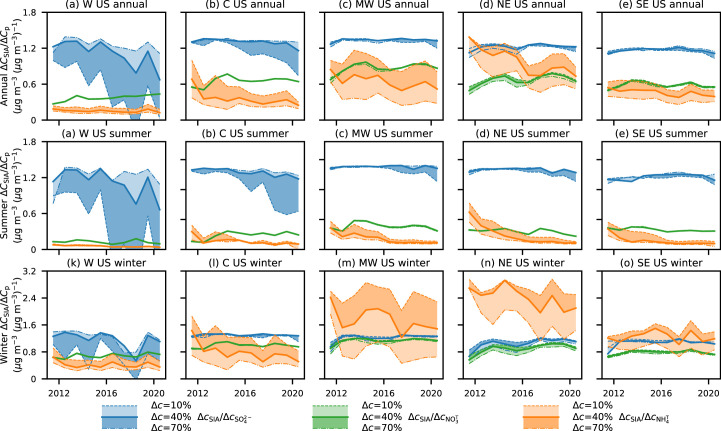
Regional means of annual, summer, and winter SIA sensitivities to precursor reductions ${\mathbf{(}}{\boldsymbol{\Delta}} {\mathbf{c}}_{{\mathbf{SIA}}}{\mathbf{/}}{\boldsymbol{\Delta}} {\mathbf{c}}_{\mathbf{p}}{\mathbf{)}}$ at different levels from 2011 to 2020. Blue, green, and orange solid lines show $\Delta {c}_{{\rm{SIA}}}/\Delta {c}_{{{\rm{SO}}}_{4}^{2-}}$, $\Delta {c}_{{\rm{SIA}}}/\Delta {c}_{{{\rm{NO}}}_{3}^{{\rm{T}}}}$, and $\Delta {c}_{{\rm{SIA}}}/\Delta {c}_{{{\rm{NH}}}_{4}^{{\rm{T}}}}$ at a reduction level of 40%, respectively. Blue, green, and orange shaded areas in the right panels represent the variabilities of $\Delta {c}_{{\rm{SIA}}}/\Delta {c}_{{{\rm{SO}}}_{4}^{2-}}$, $\Delta {c}_{{\rm{SIA}}}/\Delta {c}_{{{\rm{NO}}}_{3}^{{\rm{T}}}}$, and $\Delta {c}_{{\rm{SIA}}}/\Delta {c}_{{{\rm{NH}}}_{4}^{{\rm{T}}}}$ at reduction levels ranging from 10% (brighter areas with dashed-line boundaries) to 70% (darker areas with dotted-line boundaries).

**Extended Data Fig. 8:**
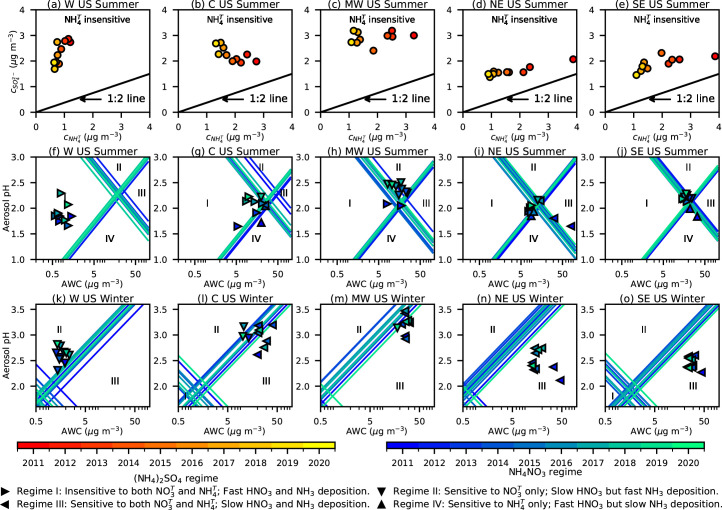
Chemical regimes for SIA formation in summer and winter. Panels (**a**)–(**e**) show the summer chemical regimes of (NH_4_)_2_SO_4_ formation. The lines in panels (**a**–**e**) indicate the condition that NH_4_^T^ explicitly balances SO_4_^2−^ to form (NH_4_)_2_SO_4_ (1:2 line) for a system that is only consist of NH_4_^T^ and SO_4_^2−^. Above the line, reducing NH_4_^T^ only removes NH_3g_ from the system, such that$\Delta {c}_{{\rm{SIA}}}/\Delta {c}_{{{\rm{NH}}}_{4}^{{\rm{T}}}}=0$. Panels (**f**)–(**o**) show NH_4_NO_3_ evaporation plays a major role. The framework of using aerosol pH and aerosol water content to determine NH_4_NO_3_ regime developed by Nenes et al.^38^ is used here (Text S3)^20,21^. The four regimes shown in panel (**f**)–(**o**) are relatively consistent when aerosol composition changes. The colour of the markers and the lines in panels (**f**)–(**o**) indicate the year.

As the aerosol composition changed and NH_4_^T^ partitioned less into aerosols, the SIA formation regime became less sensitive to ${c}_{{\rm{N}}{{\rm{H}}}_{4}^{{\rm{T}}}}$ in the rural United States (Fig. 2k–o). Although NH_4_NO_3_-containing SIA always responds to ${c}_{{\rm{N}}{{\rm{H}}}_{4}^{{\rm{T}}}}$ changes to some degree, a boundary is needed to distinguish NH_3_-sensitive and NH_3_-insensitive regimes to facilitate decision-making for air-quality and nitrogen-deposition purposes. Here, we define the boundary using both comparative and aerosol property-based approaches. In the comparative approach, we simulate *c*_SIA_ changes (${\Delta c}_{{\rm{SIA}}}$) caused by 10%, 40% and 70% reductions in each precursor (${\Delta c}_{p}$, *p* = NH_4_^T^, NO_3_^T^ or SO_4_^2−^). Instead of comparing ${\Delta {c}_{{\rm{SIA}}}}$, which scales with ${\Delta c}_{p}$ (Supplementary Figs. 9–11), we compare ${\Delta c}_{{\rm{SIA}}}/{\Delta c}_{p}$, which reflects chemical and meteorological conditions more appropriately (Supplementary Text 3). A regime is considered NH_3_-insensitive if ${\Delta c}_{{\rm{SIA}}}/{\Delta c}_{{{\rm{NH}}}_{4}^{{\rm{T}}}}$ is smaller than ${\Delta c}_{{\rm{SIA}}}/{\Delta c}_{{\rm{N}}{{\rm{O}}}_{3}^{{\rm{T}}}}$ and ${\Delta c}_{{\rm{SIA}}}/{\Delta c}_{{{\rm{SO}}}_{4}^{2-}}$. Figure 2k–o shows ${\Delta c}_{{\rm{SIA}}}/{\Delta c}_{p}$ with 40% reduction in each precursor, and Extended Data Fig. 7 shows ${\Delta c}_{{\rm{SIA}}}/{\Delta c}_{p}$ with 10% and 70% reductions. With a 40% reduction, annual ${\Delta {c}}_{\rm{SIA}}/{\Delta {c}}_{{\rm{N}{H}}_{4}^{\rm{T}}}$ decreased by 2–5% per year in all regions between 2011 and 2020 (Supplementary Table 4). As a result, by 2020, SIA formation became NH_3_-insensitive in all regions except the Northeastern United States using the comparative approach. Seasonally, SIA formation was still NH_3_-sensitive in 2020 in the winter in the Midwestern, Northeastern and Southeastern United States (Extended Data Fig. 7). We found a similar regime shift trend using the aerosol property-based approach developed by Nenes and colleagues^38^ (Supplementary Text 3 and Extended Data Fig. 8)^38^. The rapid decrease in ${\Delta {c}_{{\rm{SIA}}}/\Delta {c}_{{{\rm{NH}}}_{4}^{{\rm{T}}}}}$ highlights the importance of the SIA formation regime change between 2011 and 2020 and indicates that NH_3_ controls will be less effective for PM_2.5_ reduction in 2020 than in 2011.

## Air quality and N_r_ deposition implications

Past studies have identified NH_3_ controls as potentially effective PM_2.5_ mitigation measures in the United States, because emissions have not been directly controlled and the marginal cost for low-level reductions from agricultural sources is relatively low^39,40^. Gu and colleagues^39^ argued that the US abatement cost of NH_3_ emissions is one-tenth the cost of NO_*x*_ controls, while bringing similar welfare benefits in preventing mortality by reducing PM_2.5_ levels^39^. More broadly, of 17 studies (from 2007 to 2021) that compared the effectiveness of SO_2_, NO_*x*_ and NH_3_ emission controls in the United States, eight found that controlling NH_3_ emissions is the most effective way to reduce PM_2.5_ concentrations^39–41^ (Supplementary Table 5 provides a full list of the studies reviewed). Because of these studies and legal action by environmental organizations, in 2016 the US Environmental Protection Agency (EPA) asked state and regional air-quality regulators to evaluate potential control measures for NH_3_ when designing State Implementation Plans (SIPs) for PM_2.5_ National Ambient Air Quality Standards (NAAQS)^42^. Despite the updated requirements, most relevant regulatory agencies found additional NH_3_-emission controls unnecessary, and only one PM_2.5_ NAAQS nonattainment area (Imperial County, California) included a new rule to control NH_3_ emissions^43^. For the Regional Haze Rule, which aims to restore visibility in national parks and wilderness areas in the United States, the US EPA recommends that states ignore NH_3_ in their SIPs^44^.

Our results show that the United States has missed an opportunity to more efficiently improve air quality in rural regions by controlling NH_3_ emissions, especially from agricultural sources, as SIA formation transitioned from more NH_4_^T^-sensitive to less NH_4_^T^-sensitive between 2011 and 2020. In the early 2010s, reducing ${{\rm{c}}}_{{\rm{N}}{{\rm{H}}}_{4}^{{\rm{T}}}}$ could bring significant reductions in *c*_SIA_ in all regions except the Western United States. In 2020, however, deep ${{\rm{c}}}_{{\rm{N}}{{\rm{H}}}_{4}^{{\rm{T}}}}$ (40–70%) reductions would be needed to achieve reductions in annual *c*_SIA_ similar to those resulting from 10–40% reductions in ${c}_{{\rm{S}}{{\rm{O}}}_{4}^{2-}}$ and ${c}_{{\rm{N}}{{\rm{O}}}_{3}^{{\rm{T}}}}$ in all regions except the Northeastern United States. Reducing ${{\rm{c}}}_{{\rm{N}}{{\rm{H}}}_{4}^{{\rm{T}}}}$ in winter, when *c*_SIA_ loadings are high, was still an effective complementary measure to SO_2_- and NO_*x*_-emission controls for PM_2.5_ reductions in 2020 in the rural Midwestern, Northeastern and Southeastern United States (Extended Data Fig. 7 and 8). However, wintertime NH_3_ emissions were low in these regions, especially from agricultural sources (Supplementary Table 6), and NH_3_-emission reductions from vehicular and industrial sources might be needed to achieve the required reductions. Recent studies have shown that NH_3_ emissions from mobile and industrial sources are significantly underestimated^45^. Finally, the shift towards an NH_4_^T^-insensitive regime and the lack of incentive for NH_3_ controls for air-quality purposes in the rural United States (for example, the Regional Haze Rule) are likely to continue in rural areas as climate policies increase renewable power generation and electrify transportation. SO_2_ and NO_*x*_ emissions from fuel combustion are expected to decrease further^46^.

More importantly, our analyses also show that the inorganic N_r_ deposition regime shifted due to SO_2_- and NO_*x*_-emission reductions. As NO_*x*_ emissions decreased, reduced-form N_r_ deposition became the dominant component of N_r_ deposition and a major concern in many sensitive ecosystems^47^. Our results further illustrate that deposition patterns could change as more gaseous NH_3_ deposits near sources rather than being converted into SIAs and being transported away, shortening the effective atmospheric lifetime of reduced forms of N_r_. On the one hand, NH_3_ mitigation will be needed to protect sensitive ecosystems and reduce coastal eutrophication caused by increased N_r_ deposition near hotspots. Pan and colleagues found that 26 national parks in the United States are within 200 km of an NH_3_ hotspot (identified by satellite observations)^35^. On the other hand, increased N_r_ deposition, together with CO_2_ fertilization, has enhanced terrestrial carbon uptake, and it is unclear how the terrestrial ecosystems will respond to N_r_ deposition pattern and composition changes^48^. More flux and ecological observations are needed to investigate the multifaceted impacts of increasingly inhomogeneous N_r_ deposition.

**Extended Data Fig. 9:**
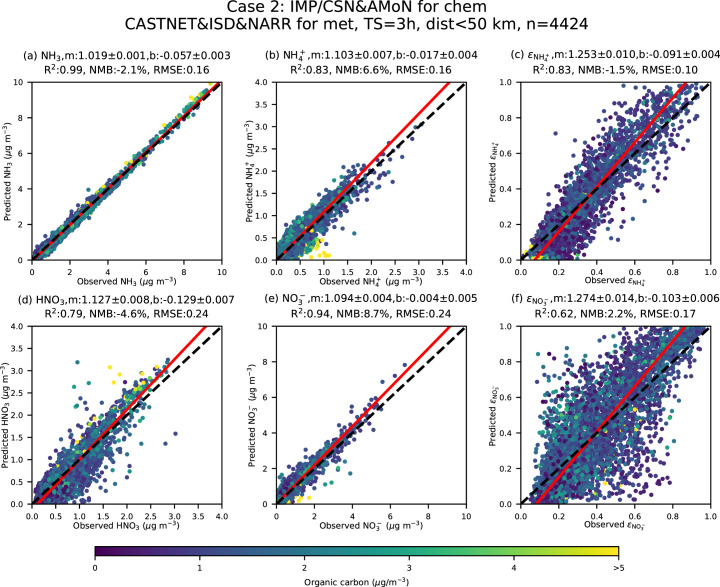
Impacts of organic compounds on ISORROPIA-II performance. Panels (**a**–**f**) show observed and ISORROPIA-II simulated NH_3_, NH_4_^+^, HNO_3_, NO_3_^−^, ${\varepsilon }_{{{\rm{NO}}}_{3}^{-}}$, and ${\varepsilon }_{{{\rm{NH}}}_{4}^{+}}$, respectively. Inputs from CASTNET, IMPROVE, EPA CSN, AMoN, ISD, and NARR are preprocessed as described as Case 2 in Table S4 and ISRROPIA-II was run with a time step (TS) of three hours. The dots are coloured by concentrations of aerosol organic carbon from IMPROVE or EPA CSN. Red lines show orthogonal distance regression results for all data, and corresponding regression parameters and evaluation statistics are shown in panel titles. Black dashed lines show the 1:1 line. The number of samples (n = 4424) is listed in the title.

Our method can be applied to routine monitoring for faster environmental policy evaluation and provides a rationale for new integrated monitoring networks in urban areas and regions impacted by enhanced wildfire and dust emissions. The integrated data and thermodynamic analysis with uncertainty estimates can also be used to improve CTMs. Our conclusions are limited to the rural United States, and urban conditions might be different. However, the approach demonstrated in this work can be used to characterize the SIA response to precursor reductions in urban regions in the United States if simultaneous observations of gaseous NH_3_ and HNO_3_, aerosol composition and meteorological conditions become available. As wildfires increase and US EPA lowers the current NAAQS for PM_2.5_ to 9 µg m^−3^ (ref. ^49^), the impacts on SIA formation of NVCs from dust and organic compounds from wildfires will probably become important for air-quality management in rural regions and warrants further investigation. For example, OAs are not considered in the inorganic aerosol model used in this study. Although organic acids could influence SIA formation, we do not find significant impacts of OAs on model performance, except for wildfire episodes with extremely high *c*_OA_ (Extended Data Fig. 9). During those events, the model underestimates both ${\varepsilon }_{{\rm{N}}{{\rm{H}}}_{4}^{+}}$ and ${\varepsilon }_{{\rm{N}}{{\rm{O}}}_{3}^{-}}$, which needs more examination with speciated OA observations. Finally, the benefits of collocated monitoring for aerosol composition and precursor concentrations demonstrated here should be considered in countries developing their own aerosol-monitoring networks.

## Methods

### Integration of the monitoring networks of gaseous precursors, aerosol composition and meteorological conditions

Several national aerosol-monitoring networks have been created in the United States since the signing of the 1990 US Clean Air Act Amendments and the 1999 Regional Haze Rule. Those providing various suites of trace-gas precursors and chemical compositions of PMs are the Clean Air Status and Trends Network (CASTNET), the Interagency Monitoring of Protected Visual Environments (IMPROVE) network, US EPA’s PM_2.5_ Chemical Speciation Monitoring Network (CSN) and the Ammonia Monitoring Network (AMoN). Extended Data Fig. 1 shows the spatial distributions of their monitoring sites in 2000, 2010 and 2020. A summary of the networks is provided in the following.

CASTNET is the only network that consistently reports weekly mean concentrations of gaseous HNO_3_ and SO_2_ in the United States in addition to aerosol composition (concentrations of SO_4_^2−^, NO_3_^−^, NH_4_^+^, Cl^−^, Na^+^, Ca^2+^, Mg^2+^ and K^+^)^51,52^. IMPROVE uses four separate modules to collect samples for speciated PM_2.5_, gravimetric PM_2.5_ and PM_10_ measurements^53^. Samples are collected for 24 h every third day. Concentrations of anions (${c}_{{{\rm{SO}}}_{4}^{2-}}$, ${c}_{{{\rm{NO}}}_{3}^{-}}$ and ${c}_{{{\rm{Cl}}}^{-}}$) are measured using ion chromatography (IC), and ${c}_{{{\rm{NH}}}_{4}^{+}}$ is reconstructed by assuming all elemental sulfur (S) and nitrogen (N) are in the forms of (NH_4_)_2_SO_4_ and NH_4_NO_3_ (ref. ^54^). This assumption could be violated when *c*_NVC_ is high or the aerosol is extremely acidic. Therefore, the reconstructed ${c}_{{{\rm{NH}}}_{4}^{+}}$ has a larger uncertainty than that of CASTNET. IMPROVE also measures concentrations of trace elements, including Na, Ca, Mg and K, using energy-dispersive X-ray fluorescence (EDXRF)^55^. EPA CSN uses similar sampling and analysis methods as those of IMPROVE. However, unlike IMPROVE, EPA CSN analyses NH_4_^+^ and Na^+^ directly using IC, which is the major difference between IMPROVE and EPA CSN^55^. AMoN is the only network providing a consistent and long-term record of gaseous NH_3_ across the United States. At AMoN sites, NH_3_ concentrations in the air are measured by Radiello passive diffusion samplers with phosphorous acid and are reported biweekly^56^.

The potential biases and observation precisions of the networks are summarized in Supplementary Table 7. There are two critical issues that could affect model simulation and validation. First, Lavery and colleagues^52^ found that CASTNET could overestimate ${c}_{{{\rm{HNO}}}_{3}}$ by 5% and underestimate ${c}_{{{\rm{NO}}}_{3}^{-}}$ by 15%, because NH_4_NO_3_ could volatilize from the Nylon filter. However, ${c}_{{{\rm{NO}}}_{3}^{{\rm{T}}}}$ is generally conserved. Therefore, the biases only impact observed ${\varepsilon }_{{{\rm{NO}}}_{3}^{-}}$ and are unlikely to influence model simulation. Second, Puchalski and colleagues^57^ reported a mean relative negative bias of 10% for ${c}_{{{\rm{NH}}}_{3}}$ from AMoN. Adjusting this potential bias, however, does not change trend analyses, and its impacts on model simulation will be discussed later (aerosol thermodynamic modelling) with sensitivity tests.

Only a fraction of CASTNET sites provide meteorological observations, which are also critical for thermodynamic analyses. CASTNET sites sponsored by the US EPA were terminated in 2011 to support AMoN operations. Consequently, there was no overlap between NH_3_ and temperature (*T*) and relative humidity (RH) observations for these sites. Therefore, meteorological observations from the Integrated Surface Database (ISD) are also included in the integration, which consists of global hourly and synoptic observations compiled from numerous sources^58^. However, there are still gaps in *T* (12%) and RH (15%) observations, and 2-m data from the North American Regional Reanalysis (NARR) with a resolution of 32 km are used to fill the gap.

To integrate the monitoring networks, we first identified the spatial window for collocation determination by comparing ${c}_{{{\rm{SO}}}_{4}^{2-}}$, ${c}_{{{\rm{NO}}}_{3}^{-}}$ and ${c}_{{{\rm{NH}}}_{4}^{+}}$ observations from the CASTNET, IMPROVE and EPA CSN sites as well as *T* and RH observations from CASTNET and ISD located within 10, 25, 50 and 100 km of each other (Supplementary Figs. 1 and 12 and Supplementary Table 8). ${c}_{{{\rm{NH}}}_{4}^{+}}$, ${c}_{{{\rm{SO}}}_{4}^{2-}}$ and ${c}_{{{\rm{NO}}}_{3}^{-}}$ from different monitoring networks generally agreed, and no significant difference was found with different spatial windows. However, *T* and RH from CASTNET and ISD significantly differ when a spatial window of 100 km is used. Therefore, a spatial window of 50 km was selected for observation integration. With this spatial window, we found 68 AMoN sites with at least CASTNET and ISD sites located within 50 km. Combining observations from these three networks provided all the inputs needed for aerosol thermodynamic modelling. All observations were averaged biweekly to match the start and end dates of AMoN observations, as it has the lowest sampling frequency.

Sites with integrated observations are shown in Fig. 1a. The black and red crosses in Fig. 1a are sites established before and after 2015, respectively. The sites are grouped according to the five US Regional Planning Organizations (RPOs): the Western Regional Air Partnership (WRAP), the Central States Air Resource Agencies (CENSARA), the Lake Michigan Air Directors Consortium (LADCO), the Mid-Atlantic/Northeast Visibility Union (MANE-VU) and the Southeastern Air Pollution Control Agencies (SESARM). These RPOs help state and county agencies develop regional strategies to achieve their air-quality goals. Here, these RPOs are referred to as the Western (WRAP), Central (CENSARA), Midwestern (LADCO), Northeastern (MANE-VU) and Southeastern (SESARM) United States, respectively. Observations from each site are shown in Supplementary Figs. 13–17. Annual numbers of biweekly observations are listed in Supplementary Table 2. Only sites with more than 70% seasonal coverage since establishment are included in the following analyses. Excluding the sites established after 2015 does not change our trend analyses (Extended Data Fig. 3, Supplementary Fig. 3 and Supplementary Table 4) and therefore the simulation results or conclusions. The regional Mann–Kendall test was used to derive consistent regional trends^30^, and only statistically significant trends (*P* < 0.05) are reported (Supplementary Table 4).

Although the sites are considered rural, they are generally representative of regional population density and emissions, especially in the Midwestern and Northeastern United States (Supplementary Table 2). Sites in the Western and Central United States are slightly more remote, with lower-than-average population densities and SO_2_ and NO_*x*_ emissions. Although some AMoN sites have been reported to be impacted by nearby agricultural emission sources^37,59^, they are not collocated with CASTNET sites. About 50% of SO_2_ emissions in the United States came from power plants and were mostly located in rural regions in 2017^25^. Highway vehicle emissions accounted for one-third of NO_*x*_ emissions in 2017, which were spread across the United States. In 2017, 10% and 5% of NO_*x*_ emissions were related to power generation and oil and gas production outside urban areas^25^. Therefore, the majority of the rural sites discussed in this study are representative of regional conditions.

### Aerosol thermodynamic modelling

We use ISORROPIA-II, a full thermodynamic model for inorganic aerosol formation, to simulate the aerosol properties and sensitivities of SIA formation to precursors. ${c}_{{{\rm{NH}}}_{4}^{{\rm{T}}}}$, ${c}_{{{\rm{NO}}}_{3}^{{\rm{T}}}}$, ${c}_{{{\rm{SO}}}_{4}^{2-}}$, ${c}_{{{\rm{Cl}}}^{-}}$, ${c}_{{{\rm{Na}}}^{+}}$, ${c}_{{{\rm{K}}}^{+}}$, ${c}_{{{\rm{Mg}}}^{2+}}$, *T* and RH from the integrated dataset are used as inputs to ISORROPIA-II. The model is run in the ‘forward mode’ to simulate gas–particle partitionings of NH_4_^T^ and NO_3_^T^. Although ISORROPIA-II has been validated with observations from intensive field campaigns, using it with biweekly averaged observations from monitoring networks has not been tested before and requires careful evaluation. We conducted nine case studies to investigate the impacts of measurement biases and low temporal resolutions (Supplementary Table 3). Following refs. ^21^ and ^12^, we evaluated the model performance by comparing simulated and observed partitionings of NH_4_^T^ and NO_3_^T^.

The simulation results shown in this study include preprocessing of the integrated observations from the monitoring networks (case 1, Extended Data Fig. 2), because running ISORROPIA-II with raw CASTNET inputs and a time step of two weeks (case 3, Supplementary Fig. 18) leads to large errors in both ${\varepsilon }_{{{\rm{NH}}}_{4}^{+}}$ and ${\varepsilon }_{{{\rm{NO}}}_{3}^{-}}$.

CASTNET utilizes an open-face filter and collects both fine- and coarse-mode aerosols. Because ISORROPIA-II does not consider aerosol size and its mixing state^31^, using ${c}_{{{\rm{Na}}}^{+}}$, ${c}_{{{\rm{Ca}}}^{2+}}$, ${c}_{{{\rm{Mg}}}^{2+}}$ and ${c}_{{{\rm{Cl}}}^{-}}$ observations from CASTNET directly could cause an overestimation of ${c}_{{{\rm{NO}}}_{3}^{-}}$ and an underestimation of ${c}_{{{\rm{NH}}}_{4}^{+}}$. Replacing CASTNET observations of NVCs and Cl^−^ with those from IMPROVE/CSN (case 2, Extended Data Fig. 9), which use an aerodynamic filter to collect PM_2.5_ samples, reduces the NMB of ${\varepsilon }_{{{\rm{NH}}}_{4}^{+}}$ from −28% (case 4, Supplementary Fig. 19) to −2%. However, not all sites have collocated IMPROVE or CSN sites. Therefore, in the default preprocessing (case 1), CASTNET observations of ${c}_{{{\rm{Na}}}^{+}}$, ${c}_{{{\rm{Ca}}}^{2+}}$, ${c}_{{{\rm{Mg}}}^{2+}}$ and ${c}_{{{\rm{Cl}}}^{-}}$ are scaled using the orthogonal distance regressions (ODRs) between concentrations of the corresponding elements or ions measured by IMPROVE or EPA CSN and those measured by CASTNET. When there is no collocated IMPROVE or EPA CSN in a site or the correlation is weak (*r* < 0.3 or *P* > 0.05), the regression result from the closest site that meets the requirements is used (Supplementary Fig. 20).

Case 2 also provides an opportunity to investigate the impacts of OAs on model performance, because IMPROVE and CSN report concentrations of organic carbon in PM_2.5_ (Extended Data Fig. 9). ISORROPIA-II overestimated gaseous NH_3_ and HNO_3_ but underestimated NH_4_^+^ and NO_3_^−^ during periods with high concentrations of organic carbon (>5 µg m^−3^). These periods also have high *c*_K_, indicating they originated from biomass burning^60^. ISORROPIA-II failing to reproduce ${\varepsilon }_{{{\rm{NH}}}_{4}^{+}}$ and ${\varepsilon }_{{{\rm{NO}}}_{3}^{-}}$ might be because the observations were averaged biweekly and could not capture the rapidly changing conditions when wildfire plumes passed by the sites or both NH_4_^+^ and NO_3_^−^ were combined with OAs. It is also unclear how the increased OAs from wildfires affect aerosol acidity. More observations are needed to investigate the impacts of OAs.

A lack of daily and diel variations of *T* and RH leads to significant underestimation of ${\varepsilon }_{{{\rm{NO}}}_{3}^{-}}$ (case 5, Supplementary Fig. 21), with an NMB of −13% and an ODR slope of 1.63. Thus, for all case studies except for cases 3 and 5, ISORROPIA-II was run with a time step of 3 h to reflect the diel patterns of *T* and RH, while ${c}_{{{\rm{SO}}}_{4}^{2-}}$, ${c}_{{{\rm{NH}}}_{4}^{{\rm{T}}}}$, ${c}_{{{\rm{NO}}}_{3}^{{\rm{T}}}}$, ${\mathrm{c}}_{{\mathrm{NVC}}}$ and ${\mathrm{c}}_{{\mathrm{cl}}^-}$ at each time step were the same as their biweekly average. The impacts of the diel patterns of the chemical inputs are considered in cases 6 (Supplementary Fig. 22) and 7 (Supplementary Fig. 23), which moderately improve the model performance. However, they were not used in the default case because they require additional empirical assumptions. Cases 8 (Supplementary Fig. 24) and 9 (Supplementary Fig. 25) show that potential sampling biases do not affect model evaluation.

Additional simulations were conducted with 10%, 40% and 70% reductions in ${c}_{{{\rm{NH}}}_{4}^{{\rm{T}}}}$, ${c}_{{{\rm{NO}}}_{3}^{{\rm{T}}}}$ and ${c}_{{{\rm{SO}}}_{4}^{2-}}$ from default preprocessing to derive ${\Delta {c}_{{\rm{SIA}}}/\Delta {c}_{{{\rm{NH}}}_{4}^{{\rm{T}}}}}$, ${\Delta {c}_{{\rm{SIA}}}/\Delta {c}_{{{\rm{NO}}}_{3}^{{\rm{T}}}}}$ and ${\Delta {c}_{{\rm{SIA}}}/\Delta {c}_{{{\rm{SO}}}_{4}^{2-}}}$. We compare ${\Delta {c}_{{\rm{SIA}}}/\Delta {c}_{p}}$ to determine the effectiveness of controlling different precursors instead of ${\Delta {c}_{{\rm{SIA}}}}$ directly (Supplementary Figs. 9–11), because ${\Delta {c}_{{\rm{SIA}}}/\Delta {c}_{{\rm{p}}}}$ is determined mostly by the SIA formation regime, whereas ${\Delta {c}_{{\rm{SIA}}}}$ also depends on the precursor concentration when a fractional reduction is considered. Simulated results for each site are shown in Supplementary Figs. 26–30. Regional results are summarized in Supplementary Tables 9–20.

Simulation uncertainties related to observation precisions and detection limits (Supplementary Table 7) were estimated using a Monte Carlo approach. Observation uncertainties were calculated using the corresponding precisions unless the absolute values were smaller than their detection limits, in which case the uncertainties were set to the detection limits. Additional uncertainties (100%) were added to NVCs to account for uncertainties introduced by the scaling processes. Assuming that the observation uncertainties are independent of each other and are normally distributed, we generated 1,000 sets of inputs randomly for the default preprocessing (case 1) and ran ISORROPIA-II 1,000 times. For ${\Delta {c}_{{\rm{SIA}}}/\Delta {c}_{{{\rm{NH}}}_{4}^{{\rm{T}}}}}$, ${\Delta {c}_{{\rm{SIA}}}/\Delta {c}_{{{\rm{NO}}}_{3}^{{\rm{T}}}}}$ and ${\Delta {c}_{{\rm{SIA}}}/\Delta {c}_{{{\rm{SO}}}_{4}^{2-}}}$, 500 simulations were conducted for each reduction level. The 2.5th and 97.5th percentiles of the simulated results were used as the lower and upper bounds (LB and UB) of uncertainties. The mean relative LB and UB uncertainties of all sites were −21% and 28% for simulated ${\varepsilon }_{{{\rm{NH}}}_{4}^{+}}$ and −21% and 22% for simulated ${\varepsilon }_{{{\rm{NO}}}_{3}^{-}}$. The relative LB and UB uncertainties for ${\varepsilon }_{{{\rm{NO}}}_{3}^{-}}$ were much larger than those of the observed ${\varepsilon }_{{{\rm{NO}}}_{3}^{-}}$ (−6% and 6% on average), highlighting that NO_3_^T^ partitioning is very sensitive to input errors. The regional mean uncertainties are summarized in Supplementary Tables 9–20.

### Aerosol pH

Aerosol acidity is a critical characteristic of the multiphase system^61^. Aerosol acidity, together with AWC, drives partitionings of the NH_4_^T^ and NO_3_^T^. Directly measuring aerosol acidity and AWC is challenging^31^. Chemical transport models have been used to simulate aerosol pH in the United States and globally^16,32^. Thermodynamic models have also been used to estimate aerosol pH based on simultaneous observations of gas and particle compositions in California, northeastern United States, southeastern United States and southeastern Canada^12,22,23,27^. Here we estimate aerosol pH using ISORROPIA-II with the integrated dataset for the rural United States from 2011 to 2020. Aerosol pH is calculated as

1 $${\rm{pH}}=-{\log }_{10}({\gamma }_{{{\rm{H}}}^{+}}{m}_{{{\rm{H}}}^{+}})$$

where ${\gamma }_{{{\rm{H}}}^{+}}$ and ${m}_{{{\rm{H}}}^{+}}$ are the molality-based activity coefficient and molality (mol per kg water) of hydrogen ions, respectively. ${\gamma }_{{{\rm{H}}}^{+}}$ is assumed to be unity in ISORROPIA-II when single-ion activities for H^+^ are required, introducing only minor uncertainties.

Guo and colleagues^21^ have shown the validity of using ISORROPIA-II with high-frequency in situ measurements from intensive field campaigns, and this method has been used to study aerosol composition and acidity changes around the world^12,62,63^. However, comparisons of pH estimated by different thermodynamic models showed that relatively constant biases exist, which should not affect the trend analyses shown here^31,64^. In addition to the assumption of ${\gamma }_{{{\rm{H}}}^{+}}={1}$, the pH simulated in this study could be slightly biased because the model only considers inorganic compounds. Previous studies have shown that organic compounds only have minor impacts on aerosol pH in the Southeastern United States where concentrations of organic compounds are high^12,21^.

To understand the drivers of aerosol pH trends, we use a first-order approximation to attribute contributions of each factor to pH changes annually. For a site at time *t* of the year (for example, 1 January 2012, 3:00), the inputs to ISORROPIA-II are ${c}_{{{\rm{SO}}}_{4}^{2-}}^{t}$, ${c}_{{{\rm{NO}}}_{3}^{{\rm{T}}}}^{t}$, ${c}_{{{\rm{NH}}}_{4}^{{\rm{T}}}}^{t}$, ${c}_{{\rm{NVC}}}^{t}$, ${c}_{{{\rm{Cl}}}^{-}}^{t}$, *T*^*t*^ and ${{\rm{RH}}}^{t}$, and change by ${\Delta {c}_{{{\rm{SO}}}_{4}^{2-}}^{t}}$, ${\Delta {c}_{{{\rm{NO}}}_{3}^{{\rm{T}}}}^{t}}$, ${\Delta {c}_{{{\rm{NH}}}_{4}^{{\rm{T}}}}^{t}}$, ${\Delta {c}_{{\rm{NVC}}}^{t}}$, ${\Delta {c}_{{{\rm{Cl}}}^{-}}^{t}}$, ${\Delta {{T}}}^{t}$ and ${\Delta {\rm{RH}}}^{t}$ in a year (that is, ${c}_{{{\rm{SO}}}_{4}^{2-}}={c}_{{{\rm{SO}}}_{4}^{2-}}^{t}+{\Delta }{c}_{{{\rm{SO}}}_{4}^{2-}}^{t}$ at 1 January 2013, 3:00). Then, the change in pH resulting from the change in an input variable (*v*; ${\Delta {\rm{pH}}}_{v}^{t}$) is estimated as a sum of pH changes caused by the same variable but with a smaller change (10% of the annual change):

2 $$\begin{array}{l}{\Delta {\rm{pH}}}_{v}^{t}=\sum _{f=0,\,0.1,\,\ldots ,\,0.9}\left[{\rm{pH}}({c}_{{{\rm{SO}}}_{4}^{2-}}^{t},\,\ldots ,\,{v}^{t},\,\ldots ,\,{{\rm{RH}}}^{t})\right.\\\qquad\qquad-{\rm{pH}}\left({c}_{{{\rm{SO}}}_{4}^{2-}}^{t}\left.+f\Delta {c}_{{{\rm{SO}}}_{4}^{2-}}^{t},\,\ldots {v}^{t}+(\,f+0.1)\Delta {v}^{t},\,\ldots ,\,{{\rm{RH}}}^{t}+f{\Delta {\rm{RH}}}^{t}\right)\right]\end{array}$$

Estimating ${\Delta {\rm{pH}}}_{v}^{t}$ using equation (2) minimizes the nonlinear pH response to chemical regime shift due to large changes in input variables. We calculate the annual mean contribution of a variable (ΔpH_*v*_) to the annual mean pH change (ΔpH) as the time average of equation (2). We estimate the error of this attribution method as

3 $${\rm{Error}}={\Delta {\rm{pH}}-\sum _{v={c}_{{{\rm{SO}}}_{4}^{2-}},\,\ldots ,\,{\rm{RH}}}{\Delta {\rm{pH}}}_{v}}$$

The results for each site are shown in Supplementary Figs. 31–35. The results shown in Extended Data Fig. 4 are cumulative contributions for 2011–2015, 2016–2020 and 2011–2020.

Aerosol pH buffering capacities of HSO_4_^−^/SO_4_^2−^, HNO_3_/NO_3_^−^ and NH_4_^+^/NH_3_ acid–base conjugate pairs were also estimated using the multiphase buffer theory developed by Zheng and colleagues^32^. The buffering capacity is defined as the ratio between the amount of acid or base added to the system (*n*_acid_ or *n*_base_ in moles per kg solution) and the associated pH change. The analytical expression for the buffering capacity ${(\beta =\frac{{\rm{d}}{n}_{{\rm{acid}}}}{{\rm{dpH}}}=\frac{{\rm{d}}{n}_{{\rm{base}}}}{{\rm{dpH}}})}$ in an aerosol multiphase buffer system is

4 $${\beta }={2.303}\left[{c}_{{{\rm{H}}}^{+}}/{\mu }_{{{\rm{H}}}^{+}}+{c}_{{{\rm{OH}}}^{-}}/{\mu }_{{{\rm{OH}}}^{-}}+\sum_{i={{\rm{SO}}}_{4}^{2-},\,{{\rm{NO}}}_{3}^{{\rm{T}}},\,{{\rm{NH}}}_{4}^{{\rm{T}}}}\displaystyle\frac{{K}_{a,\,i}^{\ast }{c}_{{{\rm{H}}}^{+}}/{\mu }_{{{\rm{H}}}^{+}}}{{({K}_{a,\,i}^{\ast }+{c}_{{{\rm{H}}}^{+}}/{\mu }_{{{\rm{H}}}^{+}})}^{2}}{c}_{i}\right]$$

where ${\mu }_{{{\rm{H}}}^{+}}$ and ${\mu }_{{{\rm{OH}}}^{-}}$ are the molar masses of H^+^ and OH^−^, *c*_*i*_ is the total concentration of the buffering agent in µmol per m^3^ air, and only HSO_4_^−^/SO_4_^2−^, HNO_3_/NO_3_^−^ and NH_4_^+^/NH_3_ are considered. The effective acid dissociation constant, ${K}_{a,\,i}^{\ast }$ (in µmol m^−3^), is

5 $${K}_{a,\,i}^{\ast }=\left\{\begin{array}{cc}{K}_{a,\,{\rm{BOH}}}\frac{{\rm{AWC}}}{{\rho }_{w}}\left(\,1+\frac{{\rho }_{w}}{{H}_{i}\,{\rm{RT}}\,{\rm{AWC}}}\right)\,, & {\rm{for}}\,{\rm{volatile}}\,{\rm{base}}\,{\rm{BOH}}\\ {K}_{a,\,{\rm{HA}}}\frac{{\rm{AWC}}}{{\rho }_{w}}/\left(\,1+\frac{{\rho }_{w}}{{H}_{i}\,{\rm{RT}}\,{\rm{AWC}}}\right)\,, & {\rm{for}}\,{\rm{volatile}}\,{\rm{acid}}\,{\rm{HA}}\end{array}\right.$$

where *K*_*a*, BOH_ and *K*_*a*, HA_ are the liquid-phase acid dissociation constant for BOH and HA expressed in molality^32^, *H*_*i*_ is the Henry’s law constant for BOH or HA in molality (mol kg^−1^ atm^−1^)^32^, and the contributions of HSO_4_^−^/SO_4_^2−^, HNO_3_/NO_3_^−^ and NH_4_^+^/NH_3_ acid–base conjugate pairs to the total buffering capacity can be expressed as

6 $${\frac{{\beta }_{i}}{\beta }}\times {100}={\frac{\frac{{K}_{a,\,i}^{\ast }[{{\rm{H}}}^{+}]}{{\mu }_{{{\rm{H}}}^{+}}}}{{\left({K}_{a,\,i}^{\ast }+\frac{[{{\rm{H}}}^{+}]}{{\mu }_{{{\rm{H}}}^{+}}}\right)}^{2}}}{c}_{i}/{\beta \times 100}$$

### Reporting summary

Further information on research design is available in the Nature Portfolio Reporting Summary linked to this Article.

## Online content

Any methods, additional references, Nature Portfolio reporting summaries, source data, extended data, supplementary information, acknowledgements, peer review information; details of author contributions and competing interests; and statements of data and code availability are available at 10.1038/s41561-024-01455-9.

### Supplementary information

Supplementary Information Supplementary text 1–3, Figs. 1–35 and Tables 1–20.

Reporting Summary

## Figures and Tables

**Extended Data Table 1:**
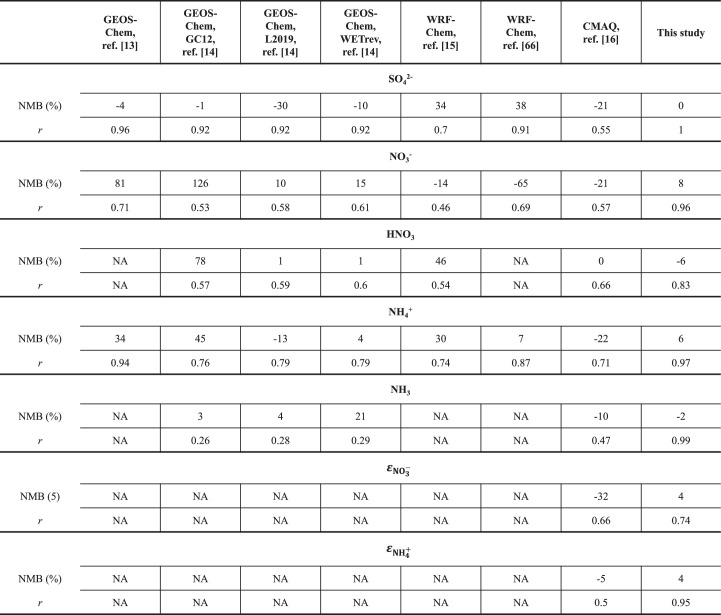
Model performance for simulating SIA formation

Normalized mean biases (NMBs, %) and Pearson correlation coefficients (*r*) between observed and simulated annual mean values are listed for sulfate (SO_4_^2−^), nitrate (NO_3_^−^), nitric acid (HNO_3_), ammonium (NH_4_^+^), ammonia (NH_3_), molar fraction of NO_3_^−^ in total nitrate (${\varepsilon }_{{{\rm{NO}}}_{3}^{-}}$), and molar fraction of NH_4_^+^ in total ammonium (${\varepsilon }_{{{\rm{NH}}}_{4}^{+}})$. Performance of chemical transport models from references (refs. ^13–16,66^) are included for comparison.

## Data Availability

The integrated observation data that support the findings of this study and the source data for figures presented in the main text, Extended Data and Supplementary Information are available in Dryad with the identifier 10.5061/dryad.zpc866tg3 (ref. ^65^).

## Code availability

The ISORROPIA-II model is available at https://nenes.eas.gatech.edu/ISORROPIA/index_old.html.

## References

[1] Bellouin N (2011). Aerosol forcing in the Climate Model Intercomparison Project (bCMIP5) simulations by HadGEM2-ES and the role of ammonium nitrate. J. Geophys. Res. Atmos.

[2] Heal MR, Kumar P, Harrison RM (2012). Particles, air quality, policy and health. Chem. Soc. Rev..

[3] Myhre, G. et al. in *Climate Change 2013: the Physical Science Basis* Ch. 8, 659–740 (Cambridge Univ. Press, 2014).

[4] Pope Iii CA (2002). Lung cancer, cardiopulmonary mortality and long-term exposure to fine particulate air pollution. JAMA.

[5] Seinfeld, J. H. & Pandis, S. N. *Atmospheric Chemistry and Physics*, *from Air Pollution to Climate Change* (Wiley, 1997).

[6] Zhang L (2023). A database of modeled gridded dry deposition velocities for 45 gaseous species and three particle size ranges across North America. J. Environ. Sci..

[7] Nenes A (2021). Aerosol acidity and liquid water content regulate the dry deposition of inorganic reactive nitrogen. Atmos. Chem. Phys..

[8] Clark CM, Tilman D (2008). Loss of plant species after chronic low-level nitrogen deposition to prairie grasslands. Nature.

[9] Phoenix GK (2012). Impacts of atmospheric nitrogen deposition: responses of multiple plant and soil parameters across contrasting ecosystems in long‐term field experiments. Glob. Change Biol..

[10] Holtgrieve GW (2011). A coherent signature of anthropogenic nitrogen deposition to remote watersheds of the northern hemisphere. Science.

[11] Janssens I (2010). Reduction of forest soil respiration in response to nitrogen deposition. Nat. Geosci..

[12] Weber RJ, Guo H, Russell AG, Nenes A (2016). High aerosol acidity despite declining atmospheric sulfate concentrations over the past 15 years. Nat. Geosci..

[13] Zhang L (2012). Nitrogen deposition to the United States: distribution, sources and processes. Atmos. Chem. Phys..

[14] Luo G, Yu F, Moch JM (2020). Further improvement of wet process treatments in GEOS-Chem v12. 6.0: impact on global distributions of aerosols and aerosol precursors. Geosci. Model Dev..

[15] Yahya K, Wang K, Gudoshava M, Glotfelty T, Zhang Y (2015). Application of WRF/Chem over North America under the AQMEII Phase 2: Part I. Comprehensive evaluation of 2006 simulation. Atmos. Environ..

[16] Chen Y, Shen H, Russell AG (2019). Current and future responses of aerosol pH and composition in the US to declining SO_2_ emissions and increasing NH_3_ emissions. Environ. Sci. Technol..

[17] Shah V (2018). Chemical feedbacks weaken the wintertime response of particulate sulfate and nitrate to emissions reductions over the eastern United States. Proc. Natl Acad. Sci. USA.

[18] Heald CL (2012). Atmospheric ammonia and particulate inorganic nitrogen over the United States. Atmos. Chem. Phys..

[19] Holt J, Selin NE, Solomon S (2015). Changes in inorganic fine particulate matter sensitivities to precursors due to large-scale US emissions reductions. Environ. Sci. Technol..

[20] Bash JO, Cooter EJ, Dennis RL, Walker JT, Pleim JE (2013). Evaluation of a regional air-quality model with bidirectional NH_3_ exchange coupled to an agroecosystem model. Biogeosciences.

[21] Guo H (2015). Fine-particle water and pH in the southeastern United States. Atmos. Chem. Phys..

[22] Guo H (2017). Fine particle pH and gas-particle phase partitioning of inorganic species in Pasadena, California, during the 2010 CalNex campaign. Atmos. Chem. Phys..

[23] Guo H (2016). Fine particle pH and the partitioning of nitric acid during winter in the northeastern United States. J. Geophys. Res. Atmos..

[24] *Center for International Earth Science Information Network (CIESIN) Revision 11* (NASA Socioeconomic Data and Applications Center, 2018).

[25] Foley KM (2023). 2002–2017 anthropogenic emissions data for air quality modeling over the United States. Data Brief..

[26] Fountoukis C, Nenes A (2007). ISORROPIA II: a computationally efficient thermodynamic equilibrium model for K^+^-Ca^2+^-Mg^2+^-NH^4+^-Na^+^-SO_4_^2−^-NO_3_^−^-Cl^−^-H_2_O aerosols. Atmos. Chem. Phys..

[27] Tao Y, Murphy JG (2019). The sensitivity of PM_2.5_ acidity to meteorological parameters and chemical composition changes: 10-year records from six Canadian monitoring sites. Atmos. Chem. Phys..

[28] *Air Pollutant Emissions Trends Data Air Pollutant Emissions Trends Data* (US EPA, 2023).

[29] Hand JL, Prenni AJ, Schichtel BA (2023). Trends in seasonal mean speciated aerosol composition in remote areas of the United States from 2000 through 2021. J. Geophys. Res. Atmos..

[30] Helsel DR, Frans LM (2006). Regional Kendall test for trend. Environ. Sci. Technol..

[31] Pye HO (2020). The acidity of atmospheric particles and clouds. Atmos. Chem. Phys..

[32] Zheng G (2020). Multiphase buffer theory explains contrasts in atmospheric aerosol acidity. Science.

[33] Thurston GD, Chen LC, Campen M (2022). Particle toxicity’s role in air pollution. Science.

[34] National Atmospheric Deposition Program; https://nadp.slh.wisc.edu/

[35] Pan D (2021). Ammonia dry deposition in an alpine ecosystem traced to agricultural emission hotpots. Environ. Sci. Technol..

[36] Van Damme M (2018). Industrial and agricultural ammonia point sources exposed. Nature.

[37] Wang R (2021). Monthly patterns of ammonia over the contiguous United States at 2‐km resolution. Geophys. Res. Lett..

[38] Nenes A, Pandis SN, Weber RJ, Russell A (2020). Aerosol pH and liquid water content determine when particulate matter is sensitive to ammonia and nitrate availability. Atmos. Chem. Phys..

[39] Gu B (2021). Abating ammonia is more cost-effective than nitrogen oxides for mitigating PM_2.5_ air pollution. Science.

[40] Lee CJ (2015). Response of global particulate-matter-related mortality to changes in local precursor emissions. Environ. Sci. Technol..

[41] Lelieveld J, Evans JS, Fnais M, Giannadaki D, Pozzer A (2015). The contribution of outdoor air pollution sources to premature mortality on a global scale. Nature.

[42] US EPA. (2016). Fine particulate matter national ambient air quality standards: state implementation plan requirements. Fed. Register.

[43] *State Implementation Plan for the Imperial County 12* *µg/m*^*3*^*PM2.5 Annual Standard* (California Air Resources Board, 2018).

[44] Tsirigotis, P. *Guidance on Regional Haze State Implementation Plans for the Second Implementation Period* (US EPA, 2019).

[45] Chen Z-L (2022). Significant contributions of combustion-related sources to ammonia emissions. Nat. Commun..

[46] Gidden MJ (2019). Global emissions pathways under different socioeconomic scenarios for use in CMIP6: a dataset of harmonized emissions trajectories through the end of the century. Geosci. Model Dev..

[47] Li Y (2016). Increasing importance of deposition of reduced nitrogen in the United States. Proc. Natl Acad. Sci. USA.

[48] O’sullivan M (2022). Process-oriented analysis of dominant sources of uncertainty in the land carbon sink. Nat. Commun..

[49] US EPA. (2024). Reconsideration of the national ambient air quality standards for particulate matter. Fed. Register.

[50] Sen PK (1968). Estimates of the regression coefficient based on Kendall’s tau. J. Am. Stat. Assoc..

[51] US Environmental Protection Agency Clean Air Markets Division; https://campd.epa.gov/

[52] Lavery TF, Rogers CM, Baumgardner R, Mishoe KP (2009). Intercomparison of clean air status and trends network nitrate and nitric acid measurements with data from other monitoring programs. J. Air Waste Manag. Assoc..

[53] The Federal Land Manager Environmental Database; https://views.cira.colostate.edu/fed/

[54] Malm WC, Sisler JF, Huffman D, Eldred RA, Cahill TA (1994). Spatial and seasonal trends in particle concentration and optical extinction in the United States. J. Geophys. Res. Atmos..

[55] Solomon PA (2014). US national PM_2.5_ chemical speciation monitoring networks—CSN and IMPROVE: description of networks. J. Air Waste Manag. Assoc..

[56] Puchalski MA (2011). Passive ammonia monitoring in the United States: comparing three different sampling devices. J. Environ. Monit..

[57] Puchalski M (2015). A statistical comparison of active and passive ammonia measurements collected at Clean Air Status and Trends Network (CASTNET) sites. Environ. Sci. Process Impacts..

[58] Smith A, Lott N, Vose R (2011). The integrated surface database: recent developments and partnerships. Bull. Am. Meteorol. Soc..

[59] Nair AA, Yu F, Luo G (2019). Spatioseasonal variations of atmospheric ammonia concentrations over the United States: comprehensive model‐observation comparison. J. Geophys. Res. Atmos..

[60] Pachon JE, Weber RJ, Zhang X, Mulholland JA, Russell AG (2013). Revising the use of potassium (K) in the source apportionment of PM_2.5_. Atmos. Pollut. Res..

[61] Tilgner A (2021). Acidity and the multiphase chemistry of atmospheric aqueous particles and clouds. Atmos. Chem. Phys..

[62] Cheng Y (2016). Reactive nitrogen chemistry in aerosol water as a source of sulfate during haze events in China. Sci. Adv..

[63] Guo H (2018). Effectiveness of ammonia reduction on control of fine particle nitrate. Atmos. Chem. Phys..

[64] Peng X (2019). Detailed analysis of estimated pH, activity coefficients and ion concentrations between the three aerosol thermodynamic models. Environ. Sci. Technol..

[65] Pan, Da et al. Regime shift in secondary inorganic aerosol formation and nitrogen deposition in the rural US. *Dryad*10.5061/dryad.zpc866tg3 (2024). 10.1038/s41561-024-01455-9 PMC11245397 39006244

[66] Tessum C, Hill J, Marshall J (2015). Twelve-month, 12 km resolution North American WRF-Chem v3.4 air quality simulation: performance evaluation. Geosci. Model Dev..

