# Supplementary material for: Regime shift in secondary inorganic aerosol formation and nitrogen deposition in the rural United States
Source: Nat Geosci. 2024 Jun 20;17(7):617–23. doi: 10.1038/s41561-024-01455-9 (PMC11245397; doi:10.1038/s41561-024-01455-9)
Supplement: Supplementary file 1 — Supplementary text 1–3, Figs. 1–35 and Tables 1–20. [file 41561_2024_1455_MOESM1_ESM.pdf]

nature geoscience

Supplementary information

https://doi.org/10.1038/s41561-024-01455-9

Regime shift in secondary inorganic aerosol formation and nitrogen deposition in the rural United States

In the format provided by the authors and unedited

TABLE OF CONTENTS

This file includes:

Supplementary Texts

Supplementary Text 1. Relationships between emissions and observed concentrations.

Supplementary Text 2. Reactive nitrogen deposition trends.

Supplementary Text 3. The chemical regime of SIA formation and inorganic N_r deposition.

Supplementary Figures

Supplementary Figure 1. Intercomparison of weekly integrated $c_{SO_4^{2-}}$, $c_{NO_3^T}$, and $c_{NH_4^T}$ observations from CASTNET, IMPROVE, and EPA CSN networks.

Supplementary Figure 2. Mean annual, summer, and winter $PM_{2.5}$ composition observed at IMPROVE and CSN sites with collocated observations.

Supplementary Figure 3. Relationships between emissions and regional mean concentrations observed at long-term sites.

Supplementary Figure 4. SO_2 emission and $c_{SO_4^{2-}}$ trends.

Supplementary Figure 5. NO_x emission and $c_{NO_3^T}$ trends.

Supplementary Figure 6. NH_3 emission and $c_{NH_4^T}$ trends.

Supplementary Figure 7. Trend analysis of total reactive nitrogen deposition.

Supplementary Figure 8. Same as Supplementary Fig. 7 but regions are defined using satellite NH_3 hotspots.

Supplementary Figure 9. Regional means of annual, summer, and winter c_{SIA} changes (Δc_{SIA}) due to 10% precursor reductions levels from 2011 to 2020.

Supplementary Figure 10. Regional means of annual, summer, and winter c_{SIA} changes (Δc_{SIA}) due to 40% precursor reductions levels from 2011 to 2020.

Supplementary Figure 11. Regional means of annual, summer, and winter c_{SIA} changes (Δc_{SIA}) due to 70% precursor reductions levels from 2011 to 2020.

Supplementary Figure 12. Intercomparison of biweekly averaged T and RH observations from CASTNET, ISD, and NARR datasets.

Supplementary Figure 13. T, RH, $\varepsilon_{NH_4^+}$ and $\varepsilon_{NO_3^-}$, and aerosol composition from sites within the Western US (WRAP).

Supplementary Figure 14. Same as Supplementary Fig. 13 but for the Central US (CENSARA).

Supplementary Figure 16. Same as Supplementary Fig. 13 but for the Northeastern US (MANE-VU).

Supplementary Figure 17. Same as Supplementary Fig. 13 but for the Southeastern US (SESARM).

Supplementary Figure 18. Observed and ISORROPIA-II (Case 3) simulated (a) $c_{\mathrm{NH_3}}$, (b) $c_{\mathrm{NH_4^+}}$, (c) $c_{\mathrm{HNO_3}}$ (d) $c_{\mathrm{NO_3^-}}$, (e) $\varepsilon_{\mathrm{NO_3^-}}$, and (f) $\varepsilon_{\mathrm{NH_4^+}}$.

Supplementary Figure 19. Same as Supplementary Fig. 18 but for Case 4.

Supplementary Figure 20. Ratios of CASTNET and IMPROVE/EPA CSN NVC and Cl^- observations (circles) and interpolated values for sites without IMPROVE/EPA CSN sites (crosses).

Supplementary Figure 21. Same as Supplementary Fig. 18 but for Case 5.

Supplementary Figure 22. Same as Supplementary Fig. 18 but for Case 6.

Supplementary Figure 23. Same as Supplementary Fig. 18 but for Case 7.

Supplementary Figure 24. Same as Supplementary Fig. 18 but for Case 8.

Supplementary Figure 25. Same as Supplementary Fig. 18 but for Case 9.

Supplementary Figure 26. Simulated $\Delta c_{\mathrm{SIA}}/\Delta c_{\mathrm{SO_4^{2-}}}$, $\Delta c_{\mathrm{SIA}}/\Delta c_{\mathrm{NO_3^T}}$, and $\Delta c_{\mathrm{SIA}}/\Delta c_{\mathrm{NH_4^T}}$ from sites within the Western US (WRAP).

Supplementary Figure 27. Same as Supplementary Fig. 26 but for the Central US (CENSARA).

Supplementary Figure 28. Same as Supplementary Fig. 26 but for the Midwestern (LADCO).

Supplementary Figure 29. Same as Supplementary Fig. 26 but for the Northeastern US (MANE-VU).

Supplementary Figure 30. Same as Supplementary Fig. 26 but for the Southeastern US (SESARM).

Supplementary Figure 31. ISORROPIA-II Simulated aerosol acidity related properties from sites within the Western US (WRAP).

Supplementary Figure 32. Same as Supplementary Fig. 31 but for the Central US (CENSARA).

Supplementary Figure 33. Same as Supplementary Fig. 31 but for the Midwestern US (LADCO).

Supplementary Figure 34. Same as Supplementary Fig. 31 but for the Northeastern US (MANE-VU).

Supplementary Figure 35. Same as Supplementary Fig. 31 but for the Southeastern US (SESARM).

Supplementary Tables

Supplementary Table 1. Observations available at each site.

Supplementary Table 2. Region definition, information, and sites within each region.

Supplementary Table 3. Case studies of pre-processing of integrated observations for ISORROPIA simulations.

Supplementary Table 4. Region trends derived from Theil-Sen regressions and Mann-Kendall tests.

Supplementary Table 5. Literature review of studies that compared effectiveness of SO_2, NO_x, and NH_3 emission abatements on $PM_{2.5}$ reduction.

Supplementary Table 6. Annual and December NH_3 emissions in 2017 in different regions from all sources and agricultural sources.

Supplementary Table 7. Potential biases, precisions, and detection limits of observations from different monitoring networks.

Supplementary Table 8. Intercomparison of data from CASTNET, IMPROVE, CSN, ISD, and NARR at different spatial windows.

Supplementary Table 9. Observed and simulated annual $\varepsilon_{\mathrm{NH_4^+}}$.

Supplementary Table 10. Observed and simulated annual $\varepsilon_{\mathrm{NO_3^-}}$.

Supplementary Table 11. Simulated annual pH and aerosol water content (AWC).

Supplementary Table 12. Simulated annual $\Delta c_{\mathrm{SIA}}/\Delta c_{\mathrm{SO_4^{2-}}}$, $\Delta c_{\mathrm{SIA}}/\Delta c_{\mathrm{NO_3^T}}$, and $\Delta c_{\mathrm{SIA}}/\Delta c_{\mathrm{NH_4^T}}$ with 10% reductions.

Supplementary Table 13. Simulated annual $\Delta c_{\mathrm{SIA}}/\Delta c_{\mathrm{SO_4^{2-}}}$, $\Delta c_{\mathrm{SIA}}/\Delta c_{\mathrm{NO_3^T}}$, and $\Delta c_{\mathrm{SIA}}/\Delta c_{\mathrm{NH_4^T}}$ with 40% reductions.

Supplementary Table 14. Simulated annual $\Delta c_{\mathrm{SIA}}/\Delta c_{\mathrm{SO_4^{2-}}}$, $\Delta c_{\mathrm{SIA}}/\Delta c_{\mathrm{NO_3^T}}$, and $\Delta c_{\mathrm{SIA}}/\Delta c_{\mathrm{NH_4^T}}$ with 70% reductions.

Supplementary Table 15. Simulated summer $\Delta c_{\mathrm{SIA}}/\Delta c_{\mathrm{SO_4^{2-}}}$, $\Delta c_{\mathrm{SIA}}/\Delta c_{\mathrm{NO_3^T}}$, and $\Delta c_{\mathrm{SIA}}/\Delta c_{\mathrm{NH_4^T}}$ with 10% reductions.

Supplementary Table 16. Simulated summer $\Delta c_{\mathrm{SIA}}/\Delta c_{\mathrm{SO_4^{2-}}}$, $\Delta c_{\mathrm{SIA}}/\Delta c_{\mathrm{NO_3^T}}$, and $\Delta c_{\mathrm{SIA}}/\Delta c_{\mathrm{NH_4^T}}$ with 40% reductions.

Supplementary Table 17. Simulated summer $\Delta c_{\mathrm{SIA}}/\Delta c_{\mathrm{SO_4^{2-}}}$, $\Delta c_{\mathrm{SIA}}/\Delta c_{\mathrm{NO_3^T}}$, and $\Delta c_{\mathrm{SIA}}/\Delta c_{\mathrm{NH_4^T}}$ with 70% reductions.

Supplementary Table 18. Simulated winter $\Delta c_{\mathrm{SIA}}/\Delta c_{\mathrm{SO_4^{2-}}}$, $\Delta c_{\mathrm{SIA}}/\Delta c_{\mathrm{NO_3^T}}$, and $\Delta c_{\mathrm{SIA}}/\Delta c_{\mathrm{NH_4^T}}$ with 10% reductions.

Supplementary Table 19. Simulated winter $\Delta c_{\mathrm{SIA}}/\Delta c_{\mathrm{SO_4^{2-}}}$, $\Delta c_{\mathrm{SIA}}/\Delta c_{\mathrm{NO_3^T}}$, and $\Delta c_{\mathrm{SIA}}/\Delta c_{\mathrm{NH_4^T}}$ with 40% reductions.

Supplementary Table 20. Simulated winter $\Delta c_{\mathrm{SIA}}/\Delta c_{\mathrm{SO_4^{2-}}}$, $\Delta c_{\mathrm{SIA}}/\Delta c_{\mathrm{NO_3^T}}$, and $\Delta c_{\mathrm{SIA}}/\Delta c_{\mathrm{NH_4^T}}$ with 70% reductions.

Supplementary Texts

Supplementary Text 1. Relationships between emissions and observed concentrations.

Results of orthogonal distance regressions (ODR) between annual mean concentrations and emissions for the five regions are shown in Extended Data Fig. 3. Trends and correlations for 2011 – 2015 and 2016 – 2020 were also examined separately (Supplementary Table 4 and Extended Data Fig. 3a). The slope for SO_2-$c_{SO_4^{2-}}$ varied significantly from 2011 – 2015 to 2016 – 2020 in the Western US, likely because the region experienced more cross-border transport and wildfires[1]. Wildfire contribution to SO_2 emissions in the Western US increased from 3 – 14% between 2011 – 2015 to 5 – 30% between 2016 – 2020[2]. The Northeastern US has a high slope, likely because SO_2 emissions in the region were already low, and the region benefited from upwind SO_2 emission reductions in the Midwestern US. In fact, $c_{SO_4^{2-}}$ in the Northeastern US correlated better with SO_2 emissions from the Midwestern US. Accounting for this, the regression slopes for all regions except for the Western US are relatively consistent and in agreement with previous studies for SO_2-$c_{SO_4^{2-}}$ regressions[3]. This highlights the importance of regional transport and makes the case for cross-region efforts to mitigate $PM_{2.5}$ precursors. The effectiveness of SO_2 emission abatement on $c_{SO_4^{2-}}$ reduction could decrease as reductions in SO_2 and NO_x emissions continue because of enhanced SO_2 oxidation as the acidity of aerosols and fog and cloud droplets lowered[4-6]. However, this effect is more pronounced in winter and colder regions, where sulfate containing SIA may not be as important as NH_4NO_3.

Extended Data Fig. 3b shows that NO_x-$c_{NO_3^T}$ slopes were comparable in the Western, Midwestern, and Northeastern US but were low in the Central and Southeastern US. There have been discussions about the discrepancies between bottom-up and top-down NO_x emission estimates in the US, which has been most apparent in the Central and Southeastern US[7]. The low NO_x-$c_{NO_3^T}$ slopes could result from real-world NO_x emission reductions being lower than EPA estimates in these regions[7]. Jiang et al. found that in the Central US, the decrease in power plant emissions of NO_x slowed after 2011[7]. Satellite observations

indicate that the unexpected slowdown of NO_x emission reduction in the Southeastern US is linked to enhanced oil and natural gas activities[8,9]. $c_{NO_3^T}$ responses to NO_x emission reductions may change dramatically as NO_x emission decreases. It was recently shown that the NO_x-to-NO_3^T oxidation rate can be evaluated using the NO_x-volatile organic compounds (VOC) framework for ozone (O_3) formation[10]. Certain regions could be NO_3^T limited for NH_4NO_3 formation but NO_x-saturated for NO_3^T formation at the same time[10]. Controlling VOC in this case would be the best strategy to reduce NH_4NO_3 formation initially. However, similar to the O_3 control strategy, as NO_x emission reduces, the chemical regime would become NO_x limited, and controlling NO_x becomes the most effective strategy in reducing NO_3^T formation.

Supplementary Text 2. Reactive nitrogen deposition trends.

To better illustrate changes in N_r deposition, we investigated changes of N_r deposition near and away from NH_3 emission hotspots utilizing the Total Deposition Estimates Using the Measurement Model Fusion (TDep MMF) data[11,12]. The TDep MMF combines wet deposition observations from the National Trends Network (NTN), ambient air monitoring data from CASTNET, and simulations from the EPA's Air Quality Time Series (EQUATES) project[2,13]. EQUATES includes 2002 - 2019 air quality modeling using the Community Multiscale Air Quality (CMAQ) modeling system (v5.3.2) for the CONUS domain using a 12 km horizontal grid spacing[13,14]. The Air Pollutant Emissions Trends Data shown in Fig. 1 are consistent with the EQUATES emissions[15]. The CMAQ simulations in EQUATES consider the bidirectional exchange of NH_3 for fertilizer emissions using the Environmental Policy Integrated Climate (EPIC) Model[16]. For species measured by CASTNET (HNO_3, SO_4^{2-}, NO_3^-, and NH_4^+), the TDep MMF estimates dry deposition using observed concentrations and CMAQ modeled deposition velocities[11,12]. NH_3 observations from AMoN were used to evaluate CMAQ simulations but were not included in the deposition estimates[11]. The TDep MMF wet deposition estimates were calculated from the annual precipitation-weighted concentrations from NTN and a modified version of the annual precipitation estimates obtained from the PRISM Climate Group[11]. We did not include organic N_r deposition when calculating total N_r because of the large uncertainty[17].

Dividing the CONUS into four zones (<50 km, 50 - 150 km, 150 - 300 km, and >300 km), we analyze the trends of annual N_r total deposition and N_r deposition components based on the TDep MMF between 2010 and 2019 (Fig. 3 and Supplementary Fig. 7). The areas of the 95th percentile of NH_3 emission rates across the U.S. based on the 2017 EQUATES NH_3 emissions[2] are considered NH_3 emission hotspots, except for sporadic locations with just one 12 km × 12 km grid. Figure 3a shows the contours of the four zones. The trends, slopes (m), intercepts (b), and relative change rate ($m_r = m/b$) are determined using the Mann-Kendall test and the Theil-Sen regression ($p<0.05$) [18]. Despite the reductions in NO_3^T deposition, N_r total deposition showed statistically significant increasing trends only in areas within 150

km of a NH_3 emission hotspot between 2010 and 2019 because of the rapid increases in NH_4^T deposition. Because of the rapid growths of NH_4^T dry deposition, the relative increasing rates in NH_4^T deposition were faster than increases in NH_3 emissions in the corresponding zones. This corroborates our aerosol thermodynamic analysis that indicates increased N_r deposition near NH_3 hotspots.

The zones and trends change significantly when NH_3 hotspots identified by satellite observations are used to define the zones (Supplementary Fig. 8), highlighting the uncertainty of NH_4^T deposition from the TDep MMF data. These hotspots are defined as the areas of the 95th percentile of NH_3 column amounts across the US. Extended Data Fig. 6 compares the 2017 EQUATES NH_3 emissions and a high-resolution (~2 km) annual NH_3 column map derived from Infrared Atmospheric Sounding Interferometer (IASI, v2.2R) observations from 2008 – 2017[19]. The EQUATES emission hotspots in the Central and Southeastern US are not identified by the satellite map. Consequently, areas far away (>300 km) from a satellite hotspot encompass several EQUATES emission hotspots and have the fastest relative increasing rate of NH_4^T total deposition based on the TDep MMF data (Supplementary Fig. 8). The significant differences show the uncertainties of current NH_4^T deposition estimates and highlight the need for more NH_3 concentration and flux observations. More observations are needed in these areas in the Central and Southeastern US, where emission and satellite hotspots show the largest discrepancy but only a few AMoN sites are available.

Supplementary Text 3. The chemical regime of SIA formation and inorganic N_r deposition.

SIA response to a reduction in $c_{\mathrm{NH_4^T}}$ is highly sensitive to the SIA formation regime. When NH_4^T exists fully as gaseous NH_3, reducing $c_{\mathrm{NH_4^T}}$ decreases $c_{\mathrm{NH_3}}$ but not c_{SIA} (NH_4^T-insensitive). When NH_4^T is limited relative to H_2SO_4 and partitions fully to the aerosol, reducing $c_{\mathrm{NH_4^T}}$ removes particulate NH_4^+ and the reduction in c_{SIA} (Δc_{SIA}) is equivalent to the $c_{\mathrm{NH_4^T}}$ removed ($\Delta c_{\mathrm{NH_4^T}}$). When NH_4^T exceeds H_2SO_4, ammonium nitrate (NH_4NO_3) can form via reaction of NH_3 and HNO_3. The extent of NH_4NO_3 formation depends on the product of NH_3 and HNO_3 concentrations and on environmental conditions. Increased NH_4NO_3 formation occurs at lower temperature and higher relative humidity. Reducing NH_4^T in this regime can also remove HNO_3 from the aerosol, producing a multiplier effect because NH_4^+ and NO_3^- are removed together, leading to a $\Delta c_{\mathrm{SIA}}/\Delta c_{\mathrm{NH_4^T}}$ of as much as 4.4[20]. Overall, NH_4^T partitions between NH_3g and particulate NH_4^+ as it neutralizes SO_4^{2-} and NO_3^-, with the partitioning dependent on aerosol composition (including $c_{\mathrm{NO_3^T}}$, $c_{\mathrm{SO_4^{2-}}}$, and c_{NVC} that can also neutralize sulfate and nitrate), temperature, and relative humidity. Therefore, $\Delta c_{\mathrm{SIA}}/\Delta c_{\mathrm{NH_4^T}}$ is highly condition-dependent and can change with the size of reduction of any precursor.

We use ISOROPIA II to simulate Δc_{SIA} with three reduction levels (10%, 40%, and 70%) for each precursor from 2011-2020 (Supplementary Fig. 9 – 11). The sensitivities of c_{SIA} to precursor reductions are calculated as the ratios between changes in Δc_{SIA} and corresponding changes in precursor concentrations (Δc_p). $\Delta c_{\mathrm{SIA}}/\Delta c_{\mathrm{NH_4^T}}$ generally varies more from low to high reduction levels (orange areas in Extended Data Fig. 7) than $\Delta c_{\mathrm{SIA}}/\Delta c_{\mathrm{SO_4^{2-}}}$ and $\Delta c_{\mathrm{SIA}}/\Delta c_{\mathrm{NO_3^T}}$ (blue and green areas in Extended Data Fig. 7, respectively). Note that $\Delta c_{\mathrm{SIA}}/\Delta c_{\mathrm{SO_4^{2-}}}$ would decrease significantly leading to larger variations than $\Delta c_{\mathrm{SIA}}/\Delta c_{\mathrm{NH_4^T}}$ with a 70% reduction in $c_{\mathrm{SO_4^{2-}}}$ in the Western and Central US because of substantial increases in $c_{\mathrm{NO_3^-}}$ in aerosols needed to balance NVCs. The delicate balance between sulfate and NVCs also leads to the large uncertainty of $\Delta c_{\mathrm{SIA}}/\Delta c_{\mathrm{SO_4^{2-}}}$ in the Western US (Supplementary Table 14). The

large variability of $\Delta c_{\mathrm{SIA}}/\Delta c_{\mathrm{NH_4^T}}$ highlights the importance of accurately determining SIA formation regime when evaluating the effectiveness of NH_3 control.

The SIA formation regime is also evaluated using aerosol property-based regime diagrams. Extended Data Fig. 8 illustrates the chemical regime changes and associated impacts on SIA formation and N_r deposition. In summer, warm conditions favor the formation of $(NH_4)_2SO_4$, and the "1:2 line" indicates the condition that two molecules of NH_4^+ explicitly balance one molecule of SO_4^{2-} to form $(NH_4)_2SO_4$ with no NH_{3g}. From 2011 to 2020, mean conditions in all RPOs started above the line and moved further away from the line (Extended Fig. 8a – e), indicating that $c_{\mathrm{SO_4^{2-}}}$ was the limiting factor. It also shows that there were ample amounts of NH_4^T, and the importance of NH_4NO_3 formation has increased between 2011-2022, even in the summer.

The chemical regime of NH_4NO_3 formation depends not only on $c_{\mathrm{NH_4^T}}$ and $c_{\mathrm{NO_3^T}}$ but also on T, RH, $c_{\mathrm{SO_4^{2-}}}$, c_{NVC}. Rather than using $c_{\mathrm{NH_4^T}}$ and $c_{\mathrm{NO_3^T}}$ to determine the chemical regime of NH_4NO_3 formation, we use the chemical and deposition regime definition developed by Nenes et al. (2020) [20,21] in this study, and a brief overview is provided here. Because of the semi-volatile nature of NH_4NO_3, both NH_4^T and NO_3^T remain partially in the gas phase. The sensitivity of c_{SIA} to changes in $c_{\mathrm{NO_3^T}}$ is proportional to the changes in $c_{\mathrm{NO_3^-}}$:

$$\frac{\partial c_{\mathrm{SIA}}}{\partial c_{\mathrm{NO_3^T}}} = \zeta \frac{\partial c_{\mathrm{NO_3^-}}}{\partial c_{\mathrm{NO_3^T}}} = \zeta \varepsilon_{\mathrm{NO_3^-}} \tag{S1}$$

where $\varepsilon_{\mathrm{NO_3^-}}$ is the fraction of NO_3^T that partitions into aerosols. For NH_4NO_3 aerosol, $\zeta = \frac{80}{62} \approx 1.29$. Similarly, the sensitivity of c_{SIA} to changes in $c_{\mathrm{NH_4^T}}$ can be expressed as:

$$\frac{\partial c_{\mathrm{SIA}}}{\partial c_{\mathrm{NH_4^T}}} = \lambda \frac{\partial c_{\mathrm{NH_4^+}}}{\partial c_{\mathrm{NH_4^T}}} = \lambda \varepsilon_{\mathrm{NH_4^+}} \tag{S2}$$

where $\varepsilon_{\mathrm{NH_4^+}}$ is the fraction of NH_4^T that partitions into aerosols. For NH_4NO_3 aerosol, $\lambda = \frac{80}{17} \approx 4.4$.

Similarly, deposition of NO_3^T and NH_4^T can be expressed using $\varepsilon_{NO_3^-}$ and $\varepsilon_{NH_4^+}$, respectively, as:

$$F_{NO_3^T} = \frac{v_g c_{HNO_{3g}}}{\mu_{HNO_{3g}}} + \frac{v_p c_{NO_3^-}}{\mu_{NO_3^-}} = v_p\left(k + (1-k)\varepsilon_{NO_3^-}\right) c_{NO_3^T}/\mu_{NO_3^-} \quad (S3)$$

$$F_{NH_4^T} = v_g/\mu_{NH_{3g}} + v_p c_{NH_4^+}/\mu_{NH_4^+} = v_p\left(k + (1-k)\varepsilon_{NH_4^+}\right) c_{NH_4^T}\ /\mu_{NH_4^+} \quad (S4)$$

where v_g and v_p are the gas- and particulate-phase deposition velocities, respectively, and $k = v_g/v_p$. k is of the order around 10[21].

Nenes et al. (2020) [20] showed that $\varepsilon_{NO_3^-}$ and $\varepsilon_{NH_4^+}$ can be expressed as

$$\varepsilon_{NO_3^-} = \frac{\Psi\ \mathrm{AWC}}{10^{-\mathrm{pH}} + \Psi\ \mathrm{AWC}}, \Psi = \frac{RTK_{n1}H_{HNO_3}}{\gamma_{H^+}\gamma_{NO_3^-}} \quad (S5)$$

$$\varepsilon_{NH_4^+} = \frac{\Phi\ 10^{-\mathrm{pH}}\ \mathrm{AWC}}{1 + 10^{-\mathrm{pH}}\ \Phi\ \mathrm{AWC}}, \Phi = \frac{\gamma_{H^+}}{\gamma_{NH_4^+}}\frac{H_{NH_3}}{K_a}RT \quad (S6)$$

where H_{HNO_3}, K_{n1} , R, $\gamma_{H^+}, \gamma_{NO_3^-}$ are the Henry's law coefficient of HNO_3, acid dissociation constant of HNO_3, the universal gas constant, the single-ion activity of H^+ and NO_3^-, respectively; and H_{NH_3} and K_a are the Henry's law and dissociation constants for NH_3, respectively; and $\gamma_{NH_4^+}$ is the single-ion activity coefficient for NH_4^+.

Defining critical fractions of $\varepsilon_{NO_3^-}$ and $\varepsilon_{NH_4^+}$ as α and β, respectively, it is said that the aerosol is sensitive to NH_4^T or NO_3^T, when $\varepsilon_{NO_3^-} > \alpha$ or $\varepsilon_{NH_4^+} > \beta$. The relationship is opposite for NO_3^T and NH_4^T deposition. The deposition is considered fast, when $\varepsilon_{NO_3^-} < \alpha$ or $\varepsilon_{NH_4^+} < \beta$. Based on Eq. (10) and (11), the critical pH that determines NO_3^T and NH_4^T sensitive regime can be derived as:

$$\mathrm{pH}' = -\log\left(\frac{1-\alpha}{\alpha}\ \Psi\ \mathrm{AWC}\right) \quad (S7)$$

$$\mathrm{pH}'' = \log\left(\frac{1-\beta}{\beta}\ \Phi\ \mathrm{AWC}\right) \quad (S8)$$

To show a balanced picture for both SIA formation and N_r deposition, α and β are both set to 0.5, which divides the aerosol pH and AWC domain the four regimes listed in Extended Data Fig. 8f - o. However, even with this approach, there are still intra-annual variabilities of the chemical regime boundaries, highlighting the strong dependence of NH_4NO_3 formation on ambient conditions. Chemical regimes in all RPOs generally shifted towards regimes I and II, where SIA formation is less sensitive to NH_4^T and NH_4^T deposits as fast as NH_3. In 2020, only MANE-VU and SESARM remained sensitive to both NH_4^T and NO_3^T reductions (Regime III) during the winter but were also approaching the boundaries.

Supplementary Figures

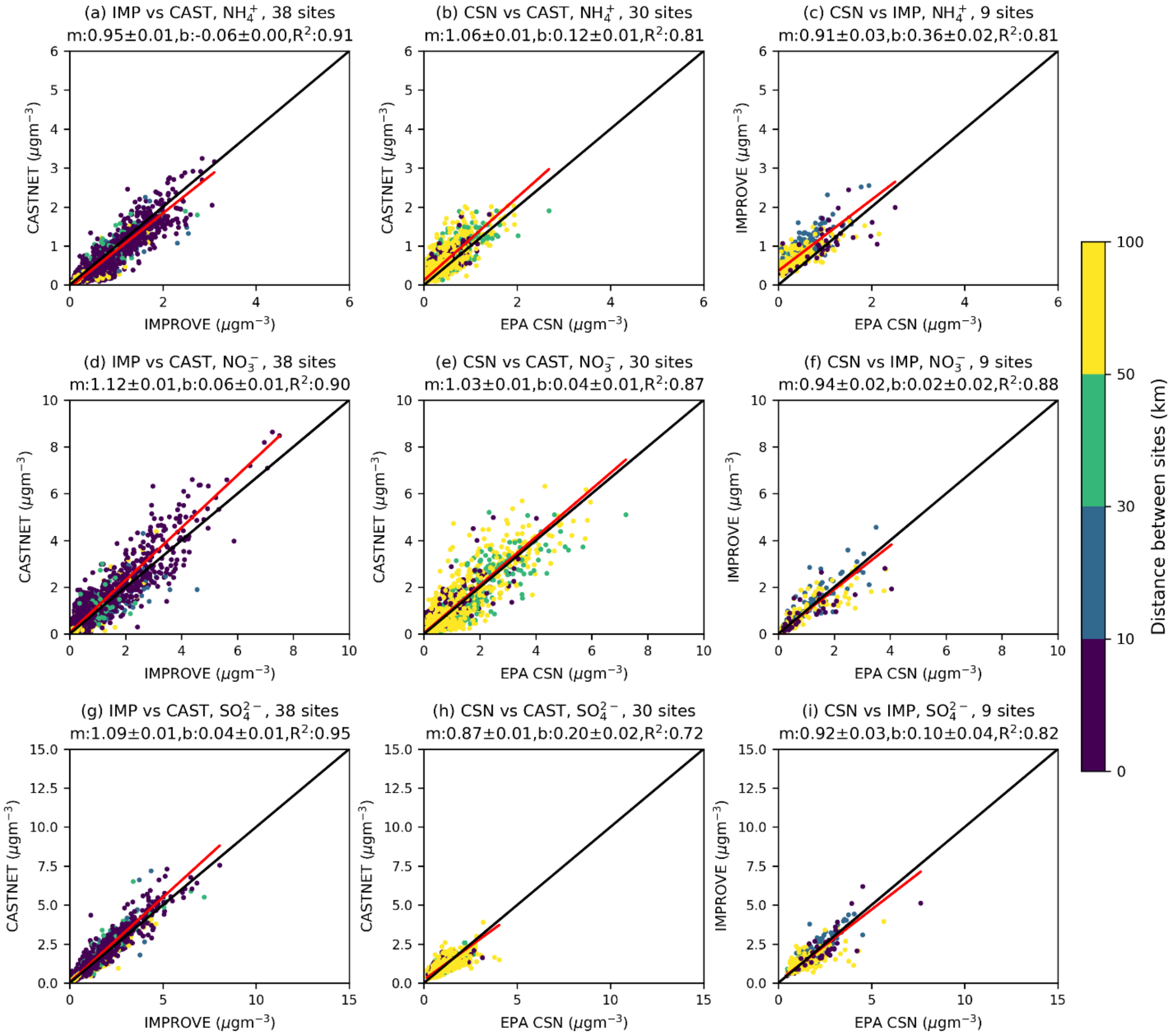

Supplementary Figure 1. Intercomparison of weekly integrated $c_{SO_4^{2-}}$, $c_{NO_3^T}$, and $c_{NH_4^T}$ observations from CASTNET, IMPROVE, and EPA CSN networks. Data points are colored by distances between the sites. Red lines show the orthogonal distance regression results, and the slopes, the offsets, and the determination coefficients (R^2) are listed below panel titles. Black lines show the 1:1 line. The uncertainties of the slopes and the offsets are the 95% confidence intervals (CI; mean ± 1.96 standard deviation (SD)). The numbers of samples are listed in Supplementary Table 8.

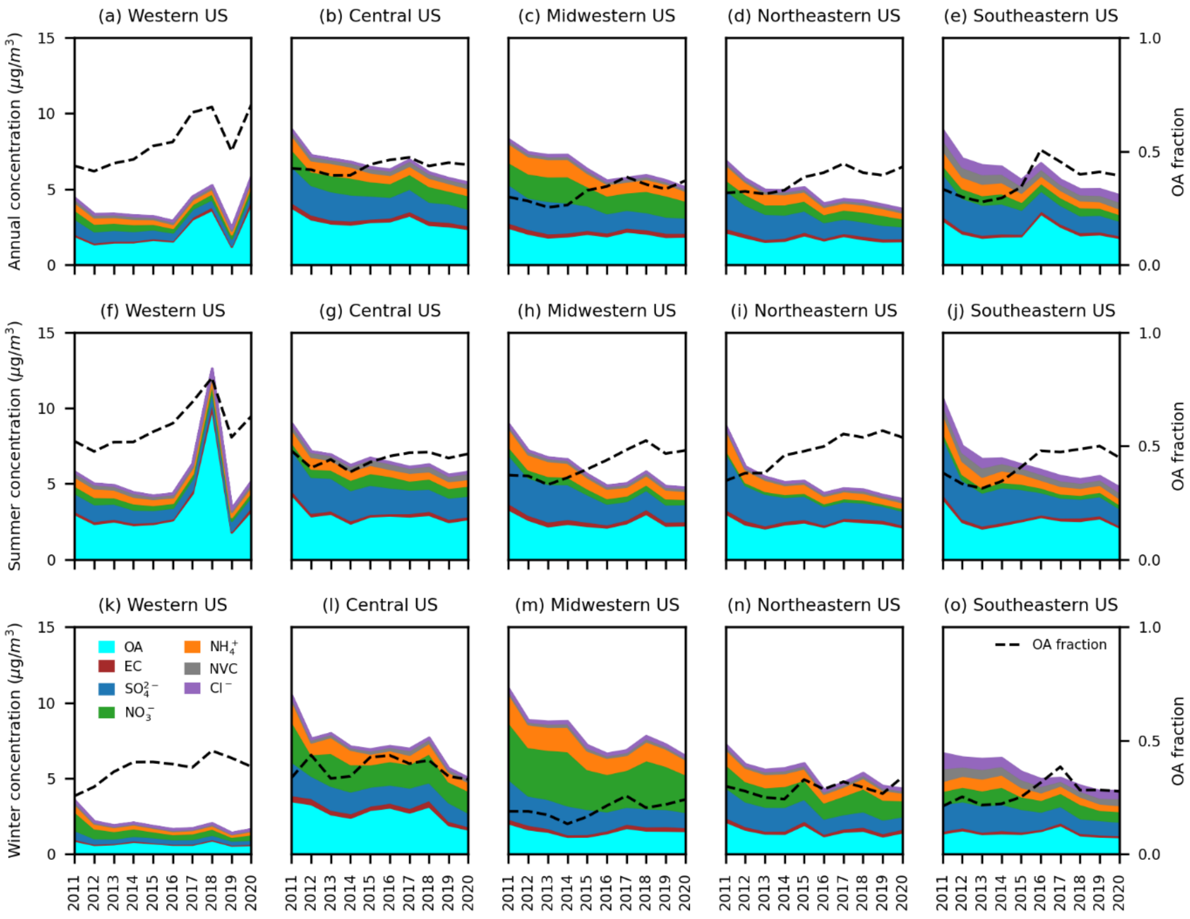

Supplementary Figure 2. Mean annual, summer, and winter $PM_{2.5}$ composition observed at IMPROVE and CSN sites with collocated observations. Cyan, brown, blue, green, orange, grey, and purple areas are organic aerosols (OA; calculated as 1.8 times the total organic carbon measured on the quartz filter), elemental carbon (EC), sulfate (SO_4^{2-}), nitrate (NO_3^-), ammonium (NH_4^+), non-volatile cations (NVC), and chloride (Cl^-), respectively. Black dashed lines show relative mass contributions of OA to $PM_{2.5}$.

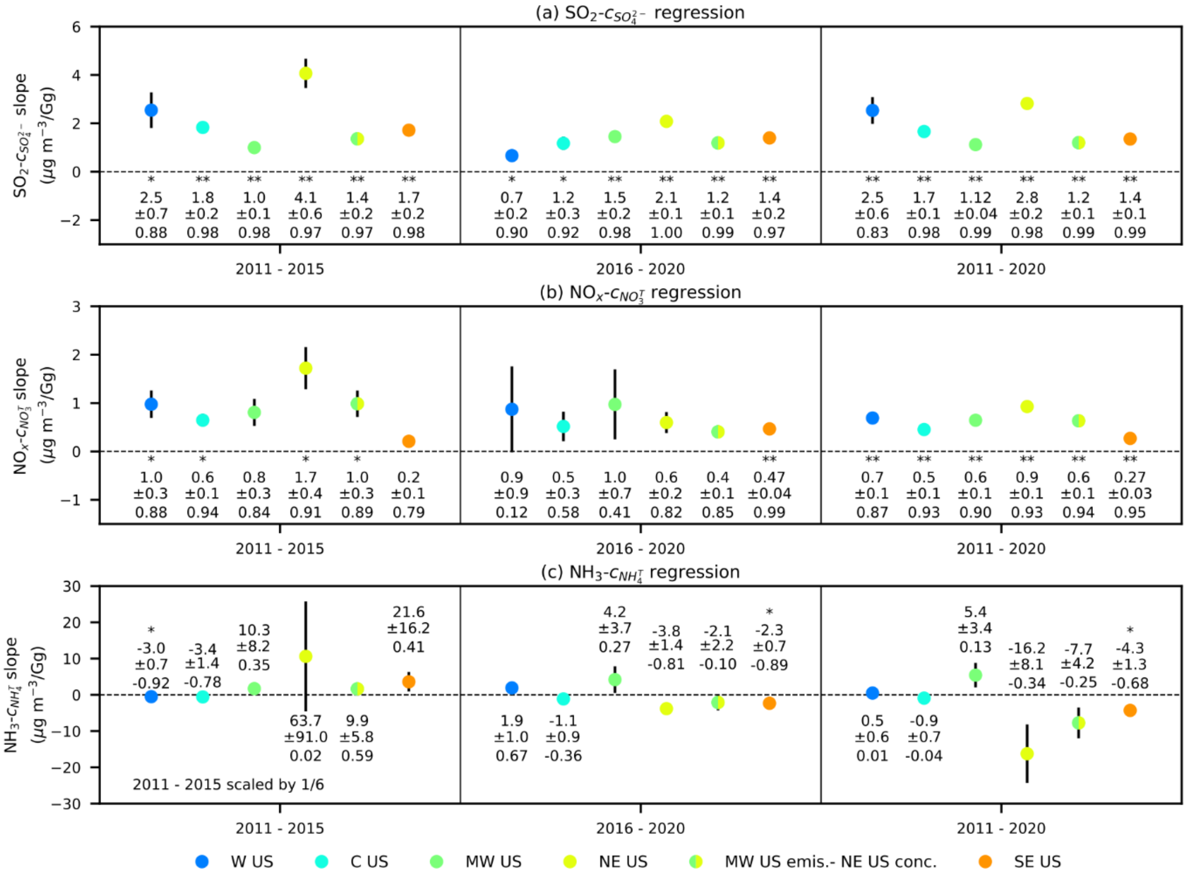

Supplementary Figure 3. Relationships between emissions and regional mean concentrations observed at long-term sites. Panels (a – c) present the orthogonal distance regression slopes of (a) SO_2 emission-$c_{SO_4^{2-}}$, (b) NO_x emissions-$c_{NO_3^T}$, and (c) NH_3 emission-$c_{NH_4^T}$ regressions for 2011 – 2015, 2016 – 2020, and 2011 – 2020 with observations from long-term sites only. Long-term sites are the ones established before 2015. "W US", "C US", "MW US", "NE US", "SE US" stand for the Western, Central, Midwestern, Northeastern, Southeastern US, respectively. Regional mean concentrations are used in the regressions, and the sample sizes for each region are 5, 5, and 10 for 2011 – 2015, 2016 – 2020, and 2011 – 2020, respectively. "*" or "**" indicate the regression has a $p<0.05$ or <0.01, respectively. The error bars show uncertainties of the regression slopes (95% CI; calculated as ±1.96 standard deviation (SD)). The numbers below are the slopes (mean values), the uncertainties (±1.96 SD), and the Pearson correlation coefficients, respectively. To illustrate NH_3-$c_{NH_4^T}$ correlation results for 2011 – 2015 in panel (c), they are scaled by 1/6.

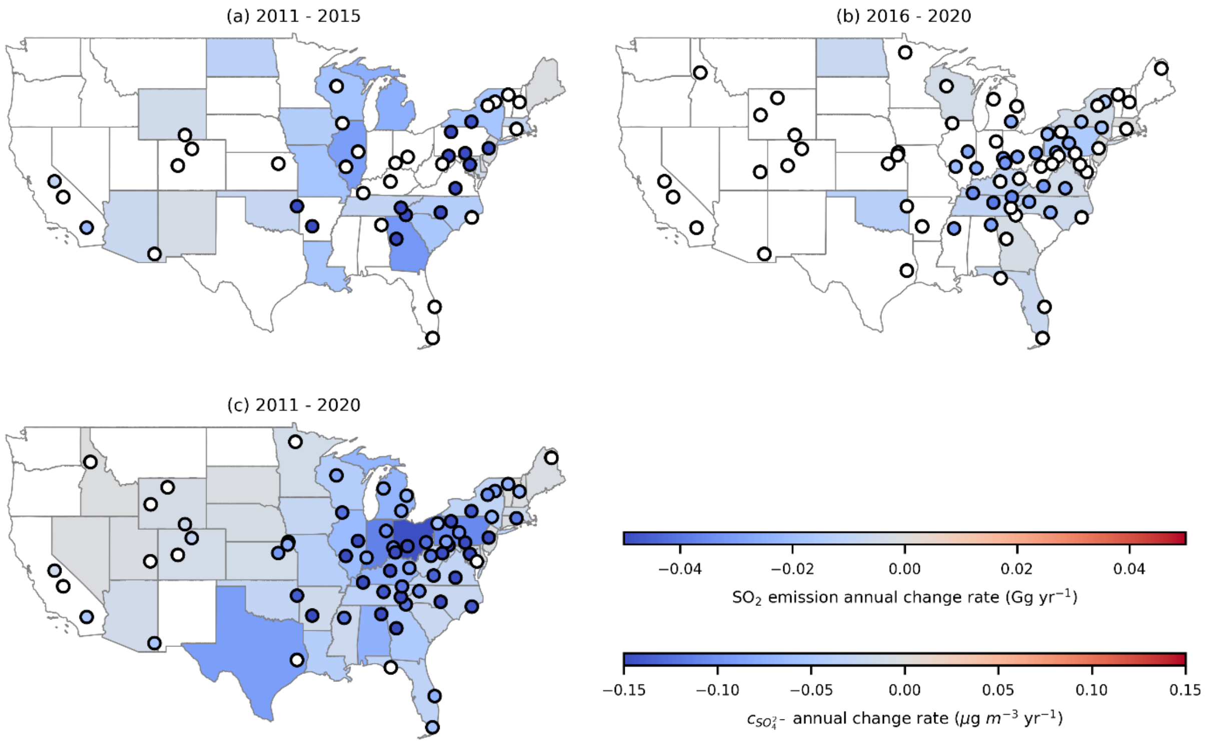

Supplementary Figure 4. SO_2 emission and $c_{SO_4^{2-}}$ trends. Emission and concentration trends are derived from Theil-Sen regressions of 5-, 5-, and 10-years data for the periods of 2011 – 2015, 2016 – 2020, and 2011 – 2020 (sample size = 5, 5, and 10), respectively. Emission trends for the states and sites are shown by state color and circle color, respectively. The regions with p<0.05 from the Mann-Kendall (MK) test are white. The base map is obtained from Natural Earth.

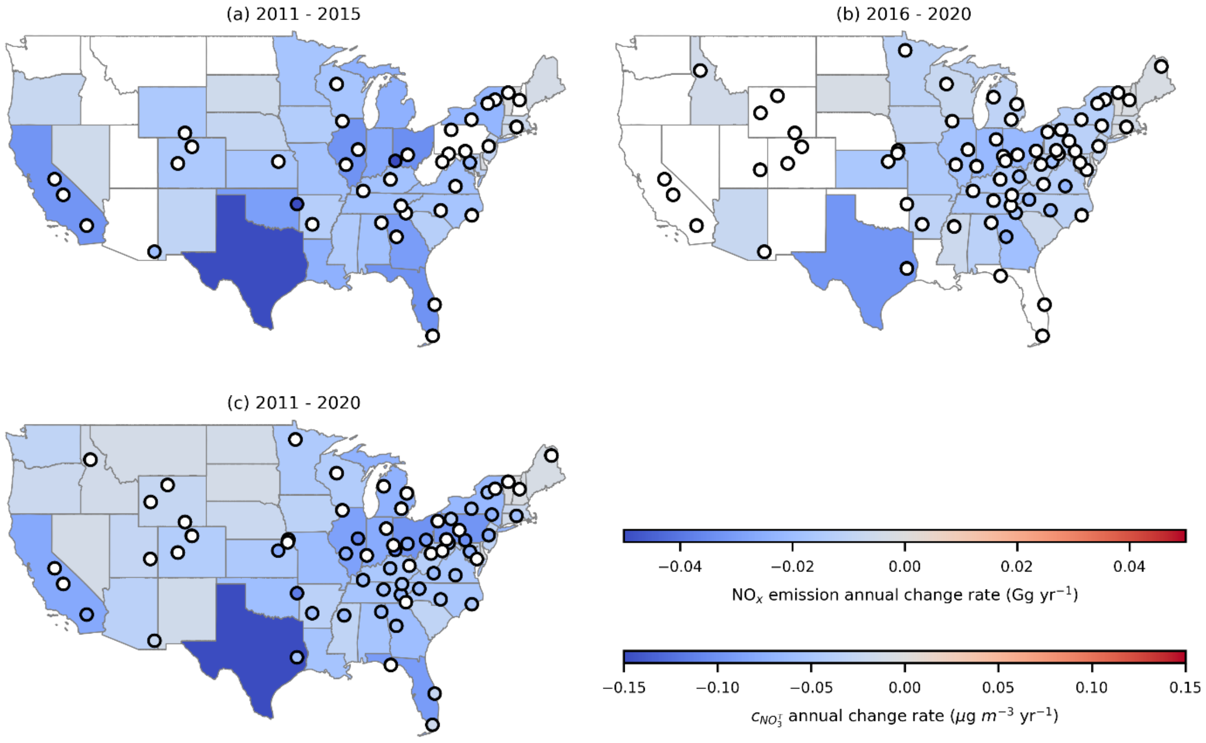

Supplementary Figure 5. NO_x emission and $c_{NO_3^-}$ trends. Emission and concentration trends are derived from Theil-Sen regressions of 5-, 5-, and 10-years data for the periods of 2011 – 2015, 2016 – 2020, and 2011 – 2020 (sample size = 5, 5, and 10), respectively. Emission trends for the states and sites are shown by state color and circle color, respectively. The regions with $p<0.05$ from the Mann-Kendall (MK) test are white. The base map is obtained from Natural Earth.

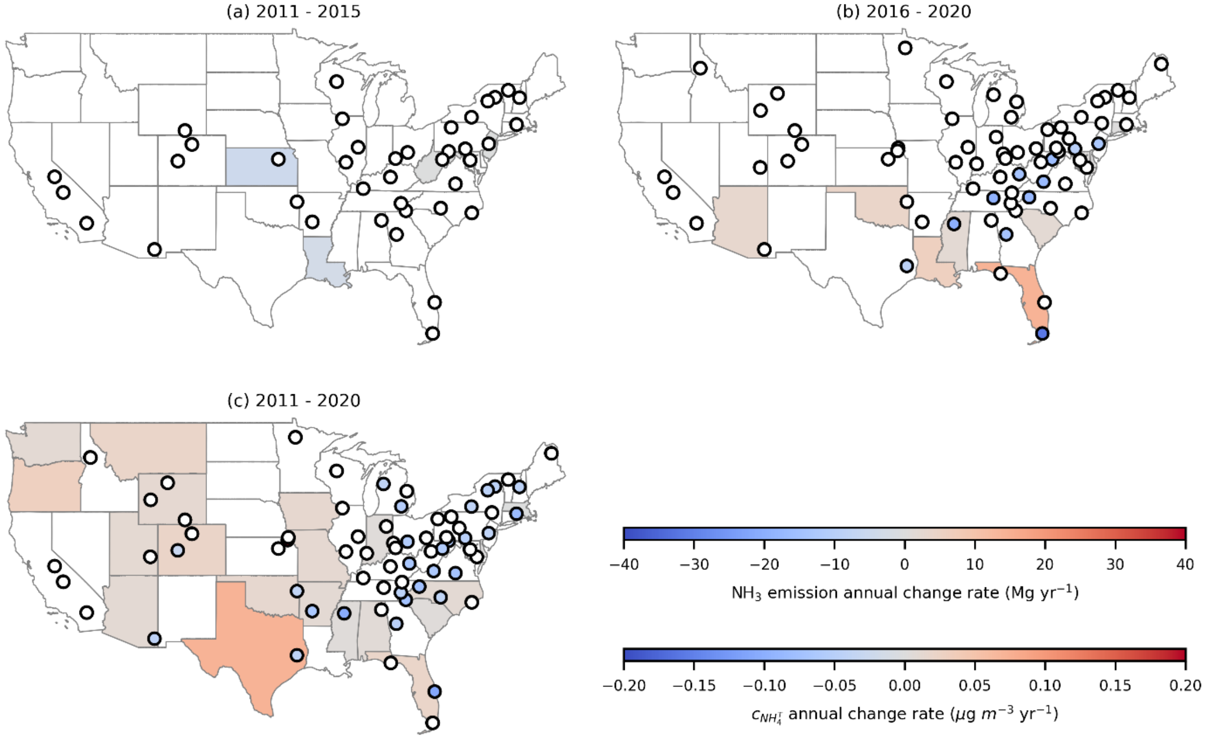

Supplementary Figure 6. NH_3 emission and $c_{NH_4^T}$ trends. Emission and concentration trends are derived from Theil-Sen regressions of 5-, 5-, and 10-years data for the periods of 2011 – 2015, 2016 – 2020, and 2011 – 2020 (sample size = 5, 5, and 10), respectively. Emission trends for the states and sites are shown by state color and circle color, respectively. The regions with p<0.05 from the Mann-Kendall (MK) test are white. The base map is obtained from Natural Earth.

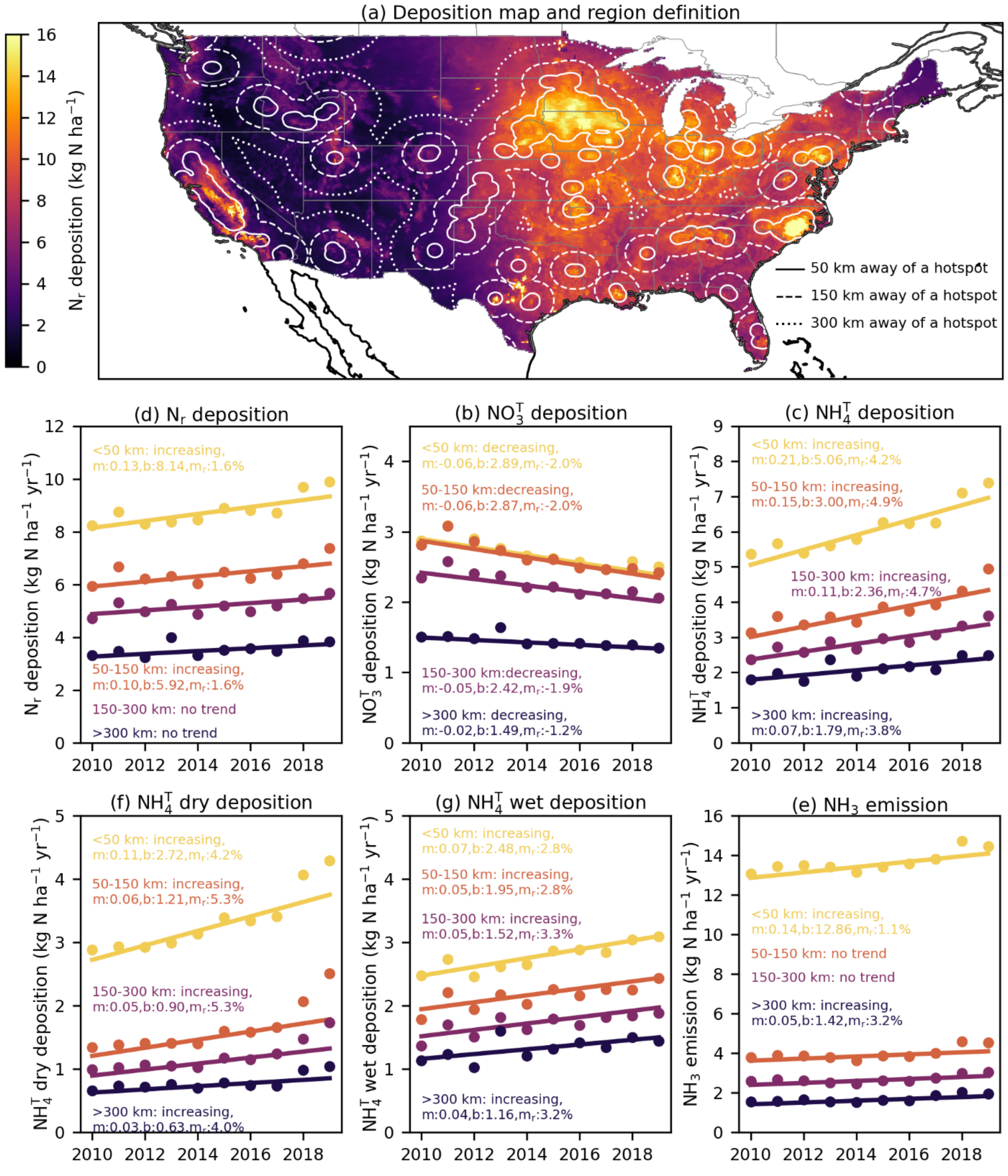

Supplementary Figure 7. Trend analysis of total reactive nitrogen deposition. Panel (a) shows the average annual total reactive nitrogen (N_r) deposition in the US between 2010 and 2019. Solid, dashed, and dotted lines show the boundaries of the areas within 50 km, 150 km, and 300 km of an NH_3 emission hotspot. The base map is from Natural Earth. Panels (b - g) show trends of total N_r deposition, total NO_3^T deposition, total NH_4^T deposition, dry NH_4^T deposition, and wet NH_4^T deposition, and NH_3 emissions, respectively. Yellow, orange, purple, and blue dots are average deposition rates in the areas <50 km, 50 – 150 km, 150 – 300 km, and >300 km from an NH_3 emission hotspot, respectively. NH_3 emission hotspots are defined as the areas of the 95th or high NH_3 emission rates in 2017 in the Contiguous US[2]. The trends and relative annual change rates are determined using the Mann-Kendall test and Theil-Sen regression with a sample size of 10[22]. Trends with $p<0.05$ are considered statistically significant.

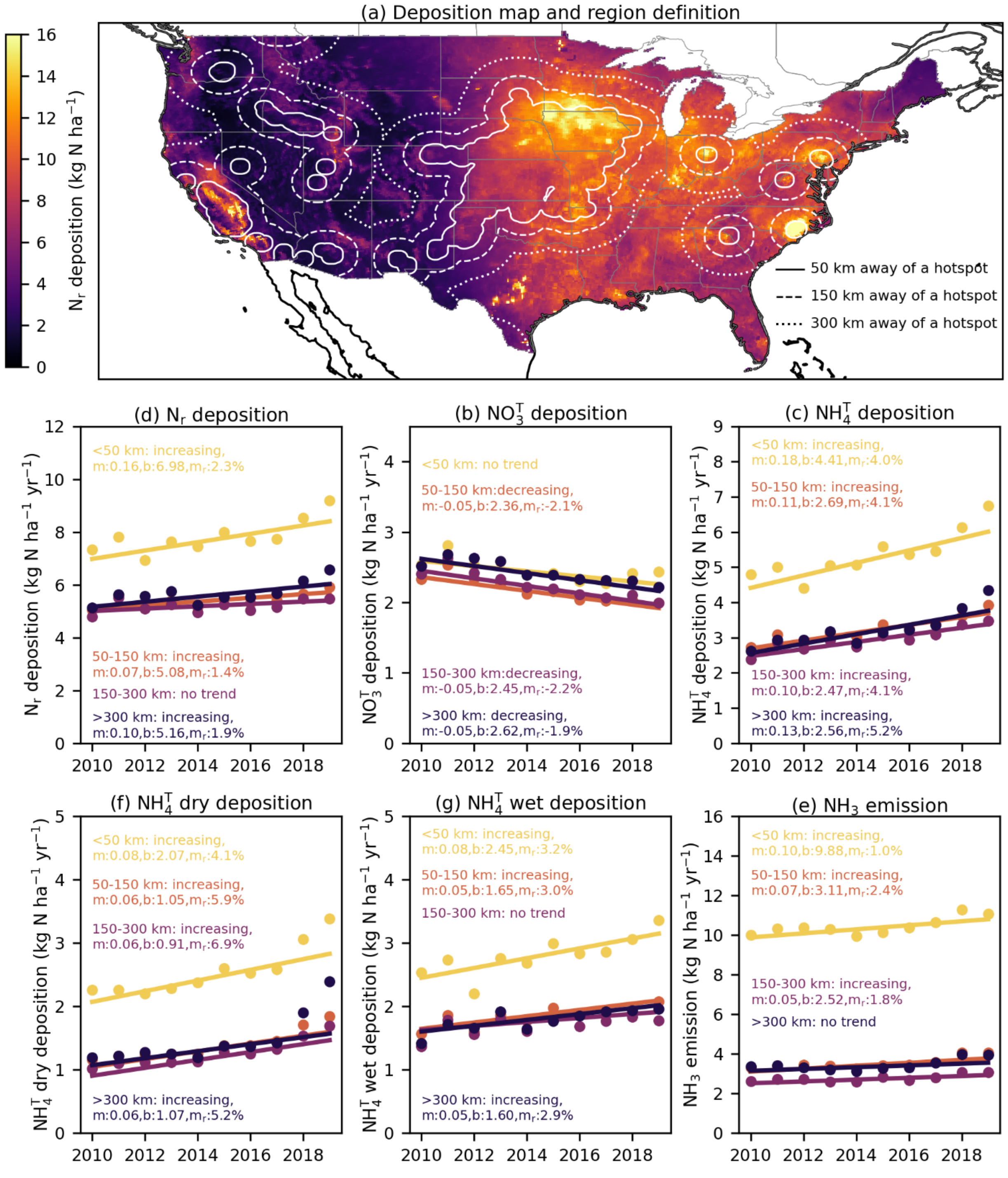

Supplementary Figure 8. Same as Supplementary Fig. 7 but regions are defined using satellite NH_3 hotspots. The trends and relative annual change rates are determined using the Mann-Kendall test and Theil-Sen regression with a sample size of 10^{22}. The base map is obtained from Natural Earth.

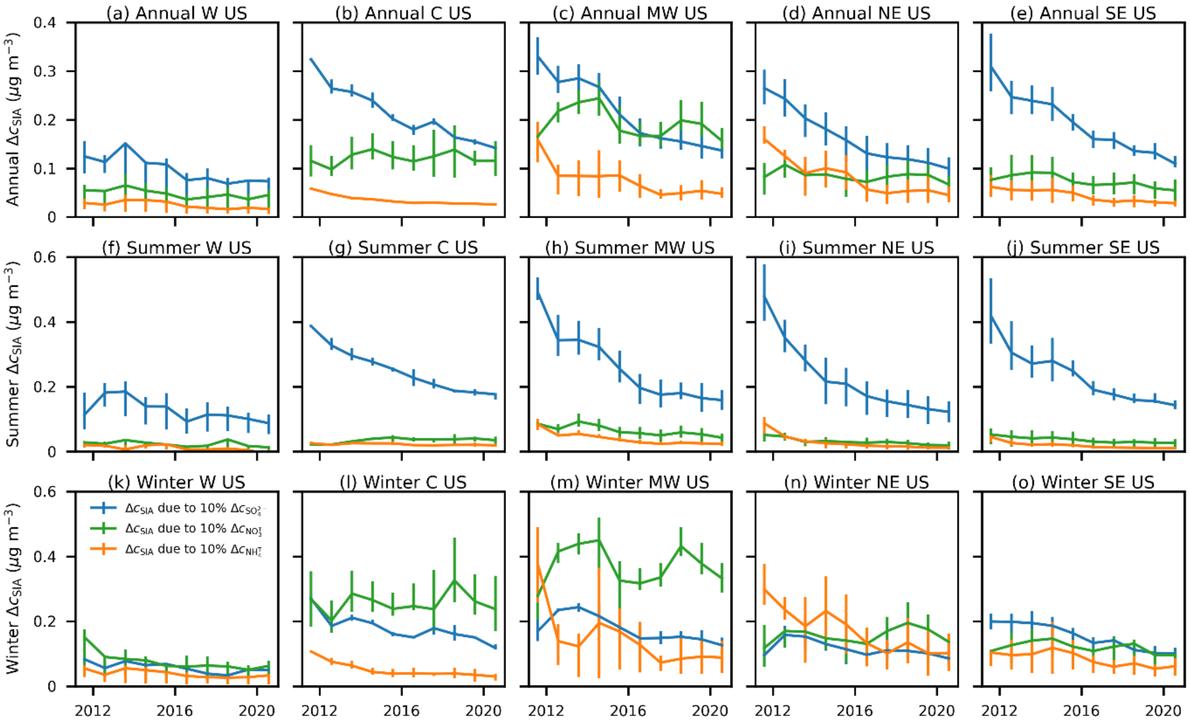

Supplementary Figure 9. Regional means of annual, summer, and winter c_{SIA} changes (Δc_{SIA}) due to 10% precursor reductions levels from 2011 to 2020. Blue, green, and orange solid lines in panels show Δc_{SIA} due to 10% reductions in $c_{SO_4^{2-}}$, $c_{NO_3^T}$, and $c_{NH_4^T}$, respectively. The vertical bars show the regional 25th and 75th percentiles of Δc_{SIA} within a region. "W US", "C US", "MW US", "NE US", "SE US" stand for the Western, Central, Midwestern, Northeastern, Southeastern US, respectively. The numbers of samples used to calculate the mean values for each region are listed in Supplementary Table 2.

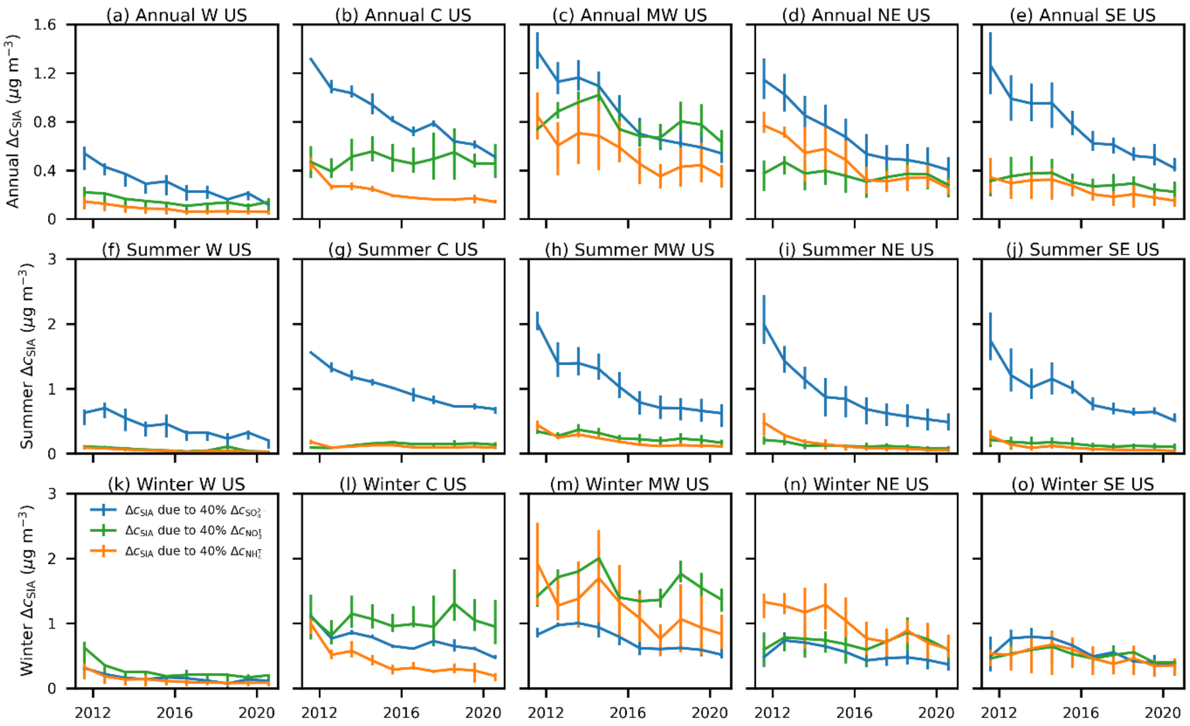

Supplementary Figure 10. Regional means of annual, summer, and winter c_{SIA} changes (Δc_{SIA}) due to 40% precursor reductions levels from 2011 to 2020. Blue, green, and orange solid lines in panels show Δc_{SIA} due to 40% reductions in $c_{SO_4^{2-}}$, $c_{NO_3^T}$, and $c_{NH_4^T}$, respectively. The vertical bars show the 25th and the 75th percentiles of Δc_{SIA} within a region. "W US", "C US", "MW US", "NE US", "SE US" stand for the Western, Central, Midwestern, Northeastern, Southeastern US, respectively. The numbers of samples used to calculate the mean values for each region are listed in Supplementary Table 2.

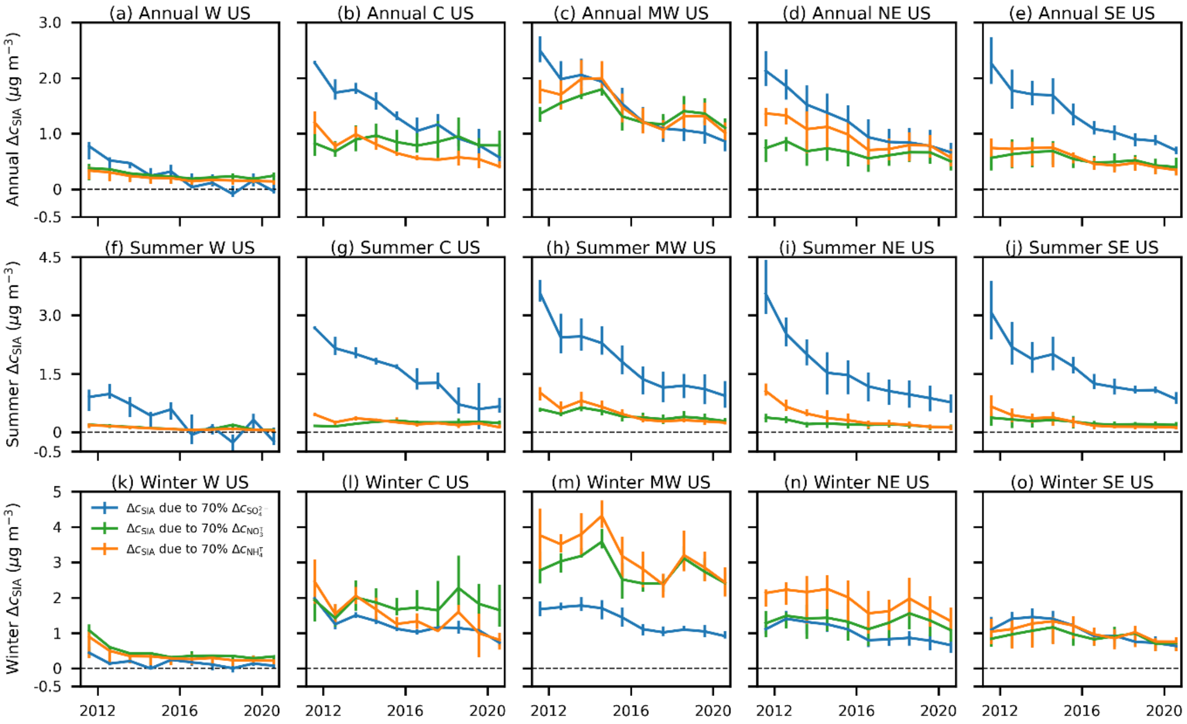

Supplementary Figure 11. Regional means of annual, summer, and winter c_{SIA} changes (Δc_{SIA}) due to 70% precursor reductions levels from 2011 to 2020. Blue, green, and orange solid lines in panels show Δc_{SIA} due to 40% reductions in $c_{SO_4^{2-}}$, $c_{NO_3^T}$, and $c_{NH_4^T}$, respectively. The vertical bars show the 25th and 75th percentiles of Δc_{SIA} within a region. "W US", "C US", "MW US", "NE US", "SE US" stand for the Western, Central, Midwestern**,** Northeastern, Southeastern US, respectively. The numbers of samples used to calculate the mean values for each region are listed in Supplementary Table 2.

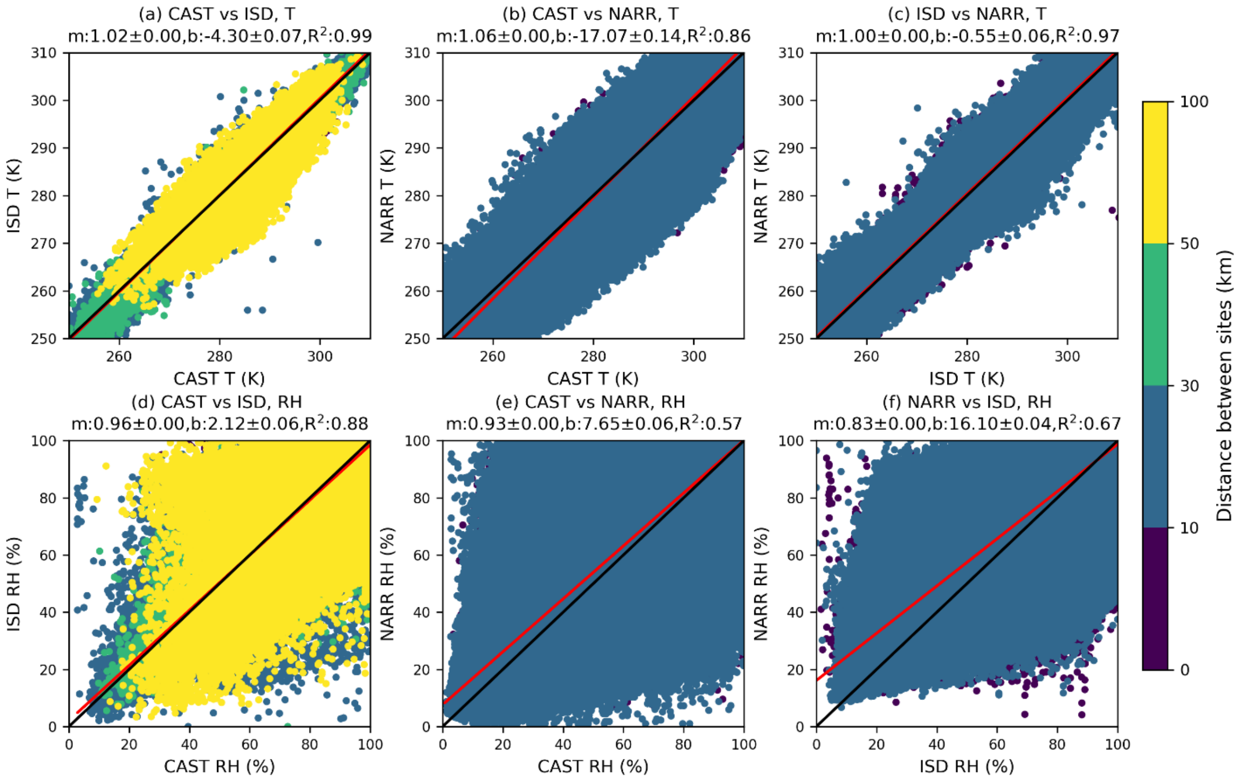

Supplementary Figure 12. Intercomparison of biweekly averaged T and RH observations from CASTNET, ISD, and NARR datasets. Data points are colored by distances between the sites. Red lines show the orthogonal distance regression results, and the slopes, offsets, and determination coefficients (R^2) are listed below panel titles. Black lines show the 1:1 line. The uncertainties of the slopes and the offsets are the 95% confidence intervals (CI; ±1.96 (SD)). The numbers of samples are listed in Supplementary Table 8.

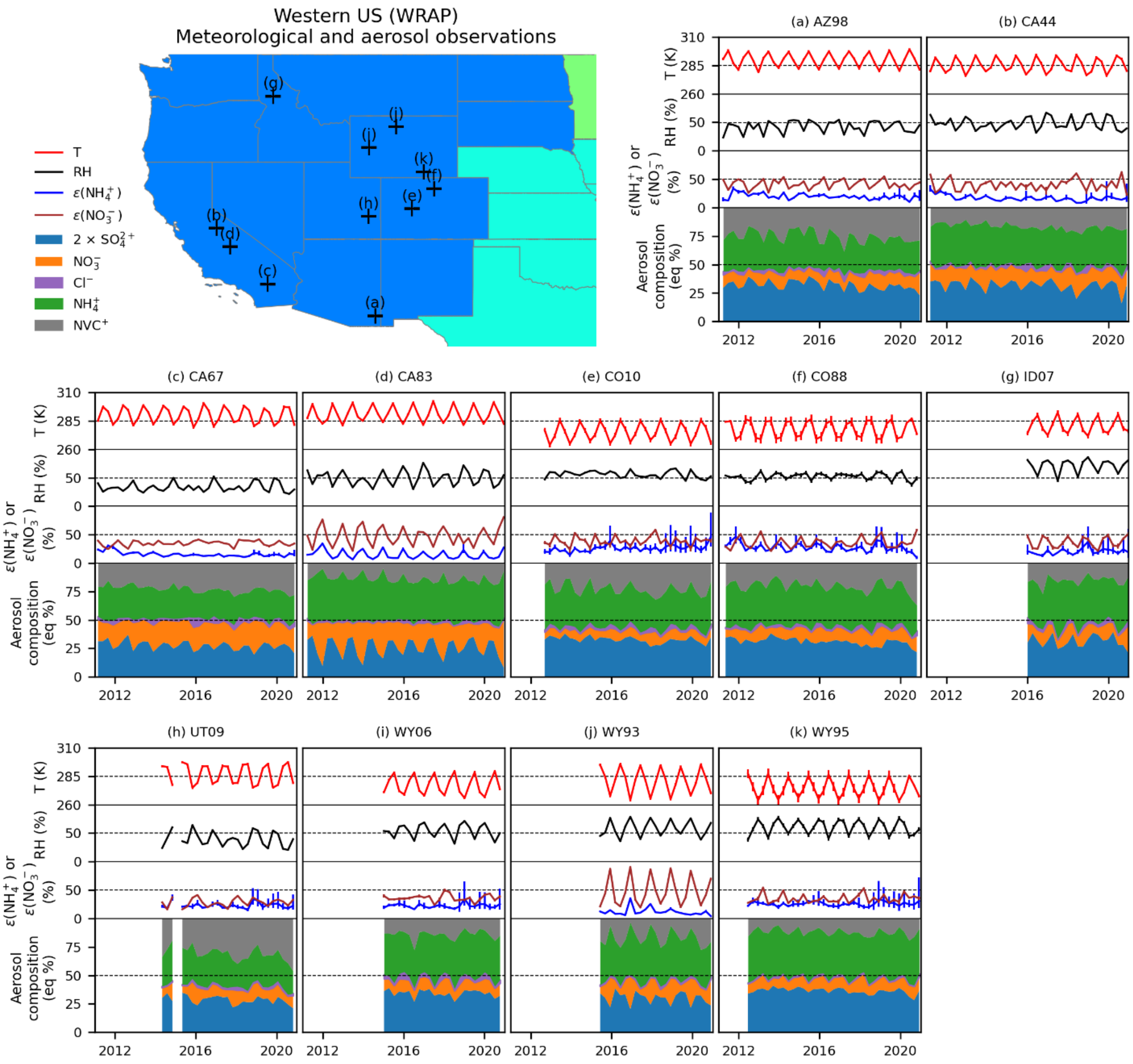

Supplementary Figure 13. T, RH, $\varepsilon_{NH_4^+}$ and $\varepsilon_{NO_3^-}$, and aerosol composition from sites within the Western US (WRAP). Aerosol composition is calculated using ion-equivalent concentrations to reflect aerosol charge balance. Lines and bars in the top panels represent the mean values and the 95% CI (as the 2.5th to the 97.5th percentiles) of the corresponding variables derived from 1000 Monte Carlo simulations. The base map is obtained from Natural Earth.

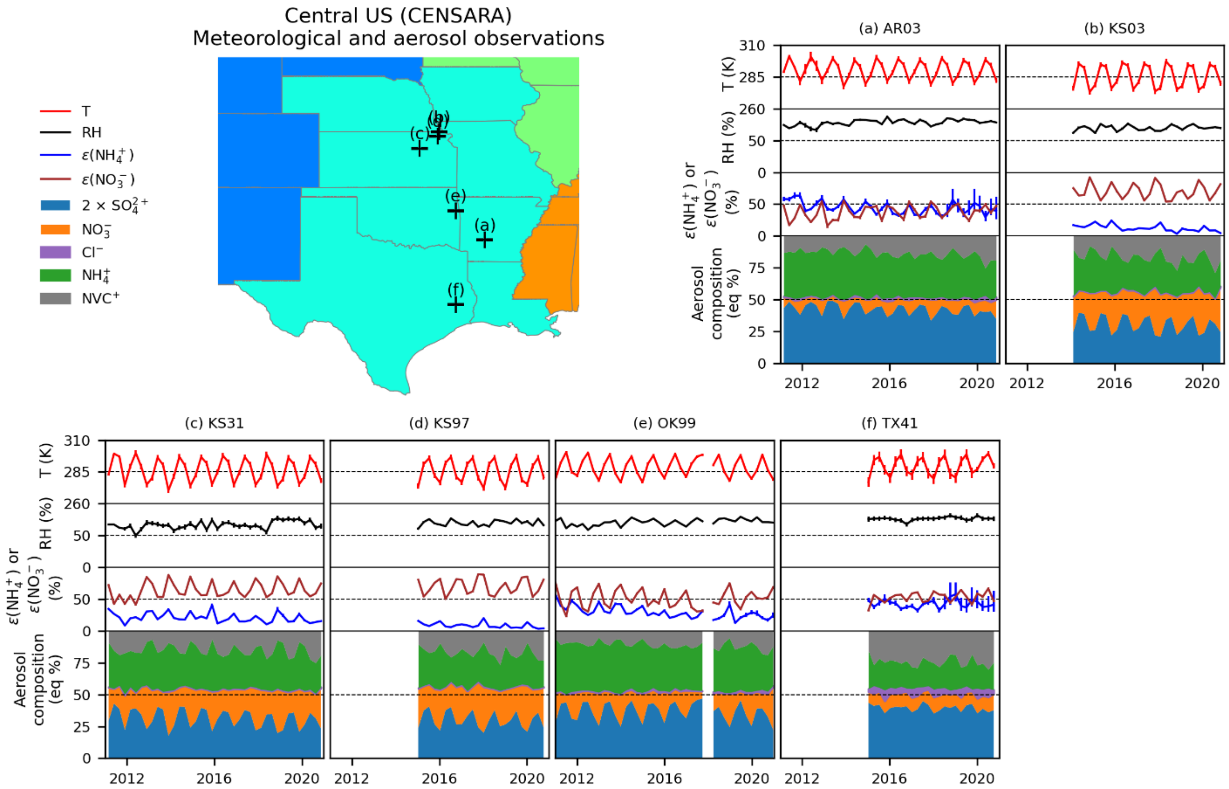

Supplementary Figure 14. Same as Supplementary Fig. 13 but for the Central US (CENSARA). Lines and bars in the top panels represent the mean values and the 95% CI (as the 2.5th to the 97.5th percentiles) of the corresponding variables derived from 1000 Monte Carlo simulations. The base map is obtained from Natural Earth.

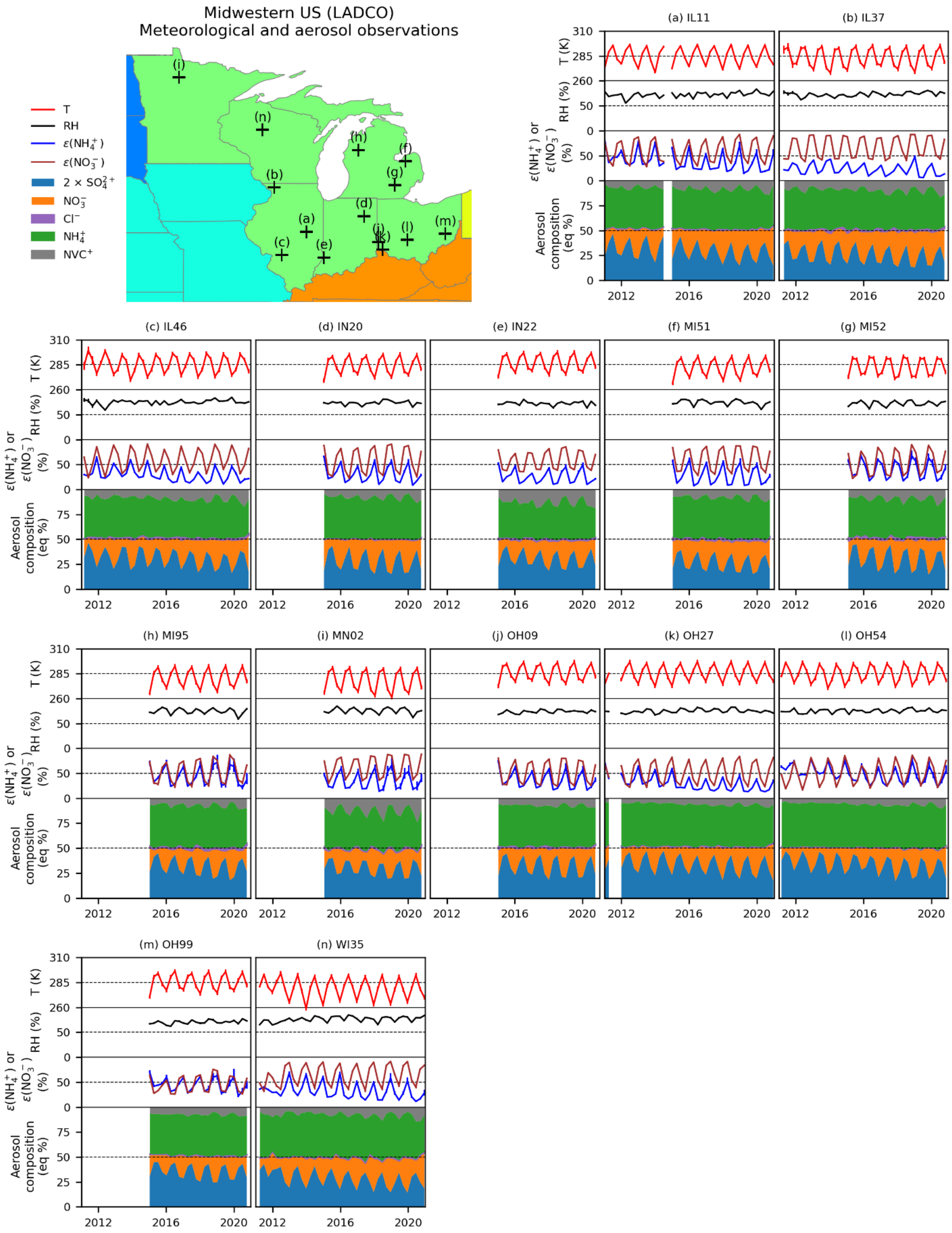

Supplementary Figure 15. Same as Supplementary Fig. 13 but for the Midwestern US (LADCO). Lines and bars in the top panels represent the mean values and the 95% CI (as the 2.5th to the 97.5th percentiles) of the corresponding variables derived from 1000 Monte Carlo simulations. The base map is obtained from Natural Earth.

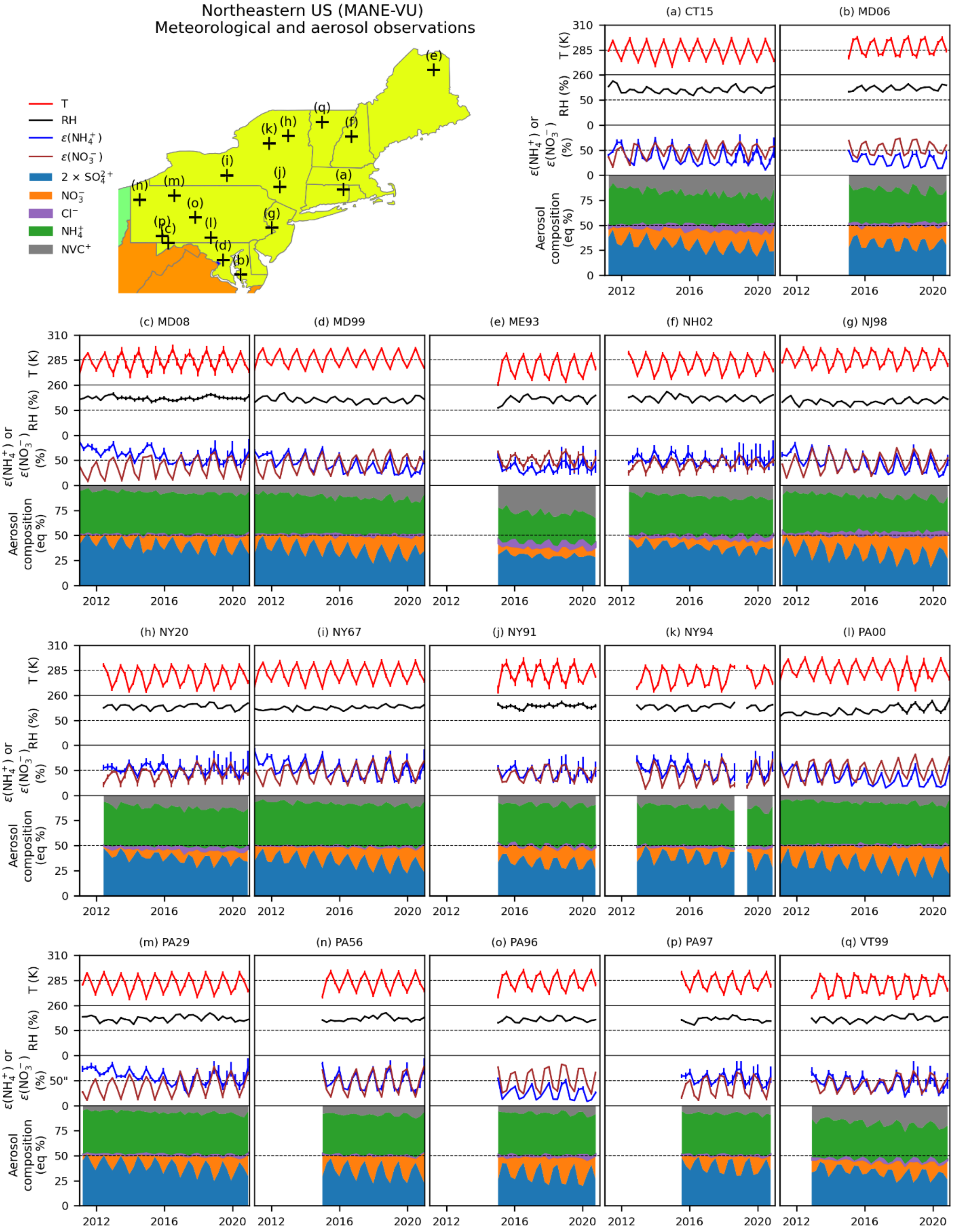

Supplementary Figure 16. Same as Supplementary Fig. 13 but for the Northeastern US (MANE-VU). Lines and bars in the top panels represent the mean values and the 95% CI (as the 2.5th to the 97.5th percentiles) of the corresponding variables derived from 1000 Monte Carlo simulations. The base map is obtained from Natural Earth.

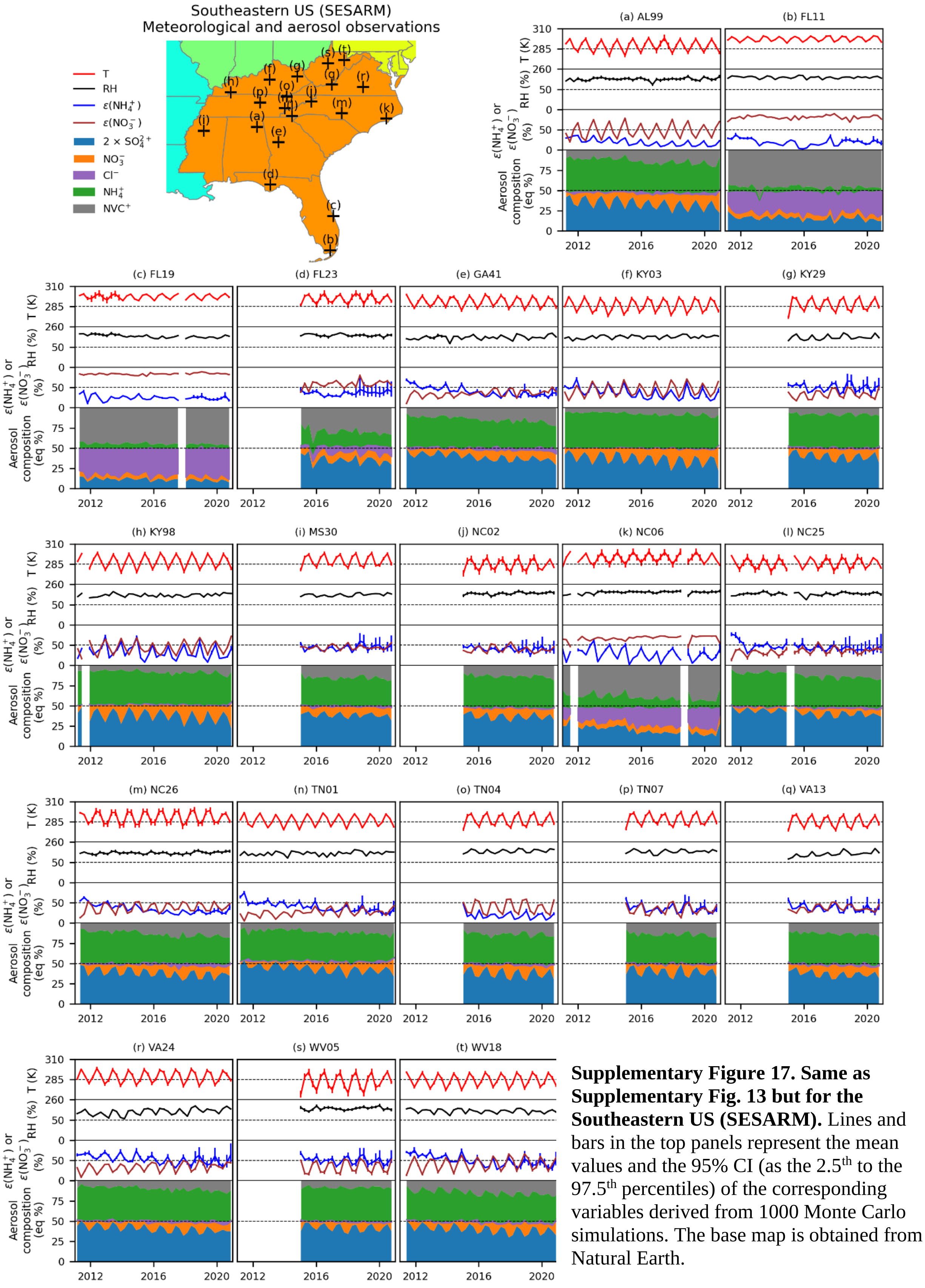

Supplementary Figure 17. Same as Supplementary Fig. 13 but for the Southeastern US (SESARM). Lines and bars in the top panels represent the mean values and the 95% CI (as the 2.5th to the 97.5th percentiles) of the corresponding variables derived from 1000 Monte Carlo simulations. The base map is obtained from Natural Earth.

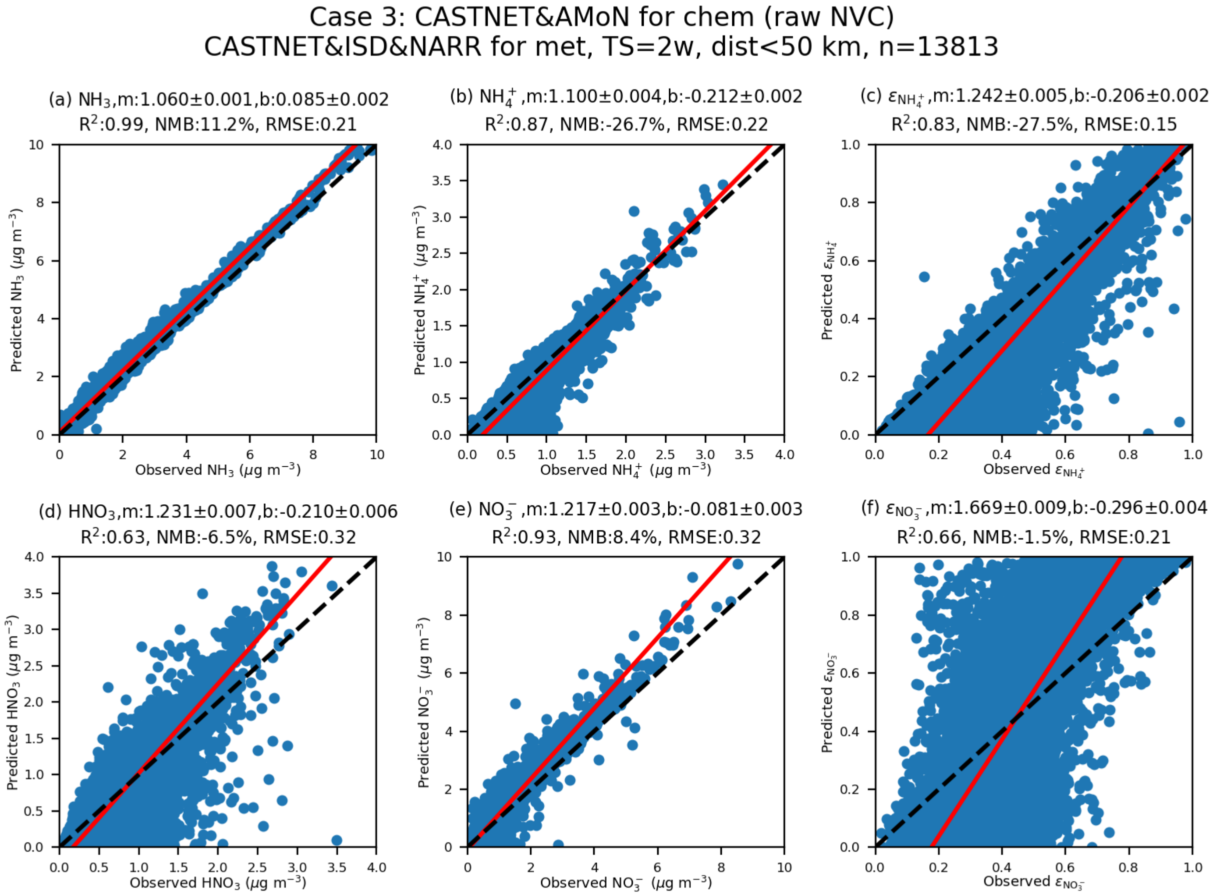

Supplementary Figure 18. Observed and ISORROPIA-II (Case 3) simulated (a) c_{NH_3}, (b) $c_{NH_4^+}$, (c) c_{HNO_3} (d) $c_{NO_3^-}$, (e) $\varepsilon_{NO_3^-}$, and (f) $\varepsilon_{NH_4^+}$. Inputs of the ISSORROPIA simulations are described in Supplementary Table 3. Red lines show orthogonal distance regression results (prediction = m·observation + b), and corresponding regression parameters and evaluation statistics (determination coefficient (R^2), normalized mean bias (NMB), and root mean square error (RMSE)) are shown in panel titles. The slope and offset uncertainties are the 95% CI of the regression (±1.96SD). Black dashed lines show the 1:1 line. The number of samples is 13813 for all panels.

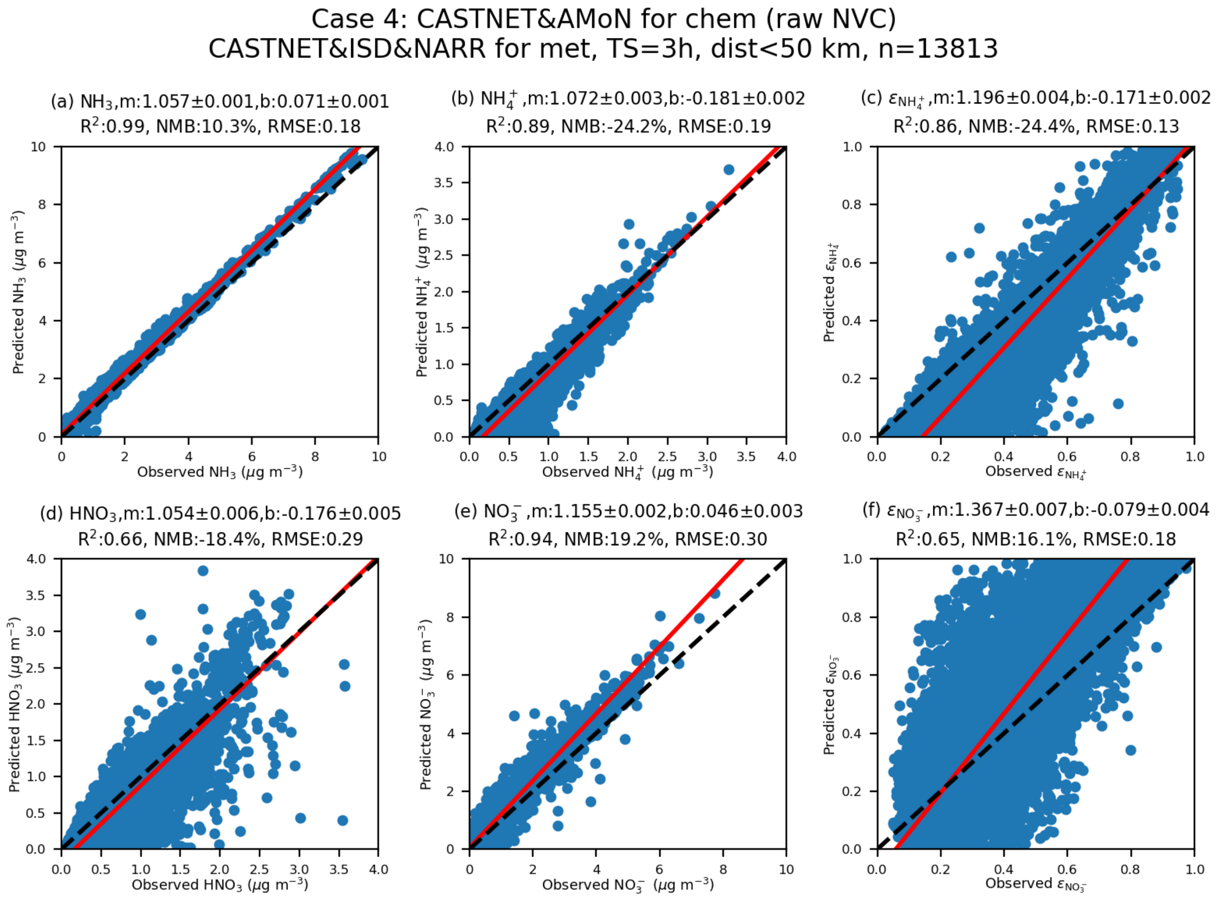

Supplementary Figure 19. Same as Supplementary Fig. 18 but for Case 4. The slope and offset uncertainties are the 95% CI of the regression (±1.96SD). Black dashed lines show the 1:1 line. The number of samples is 13813 for all panels.

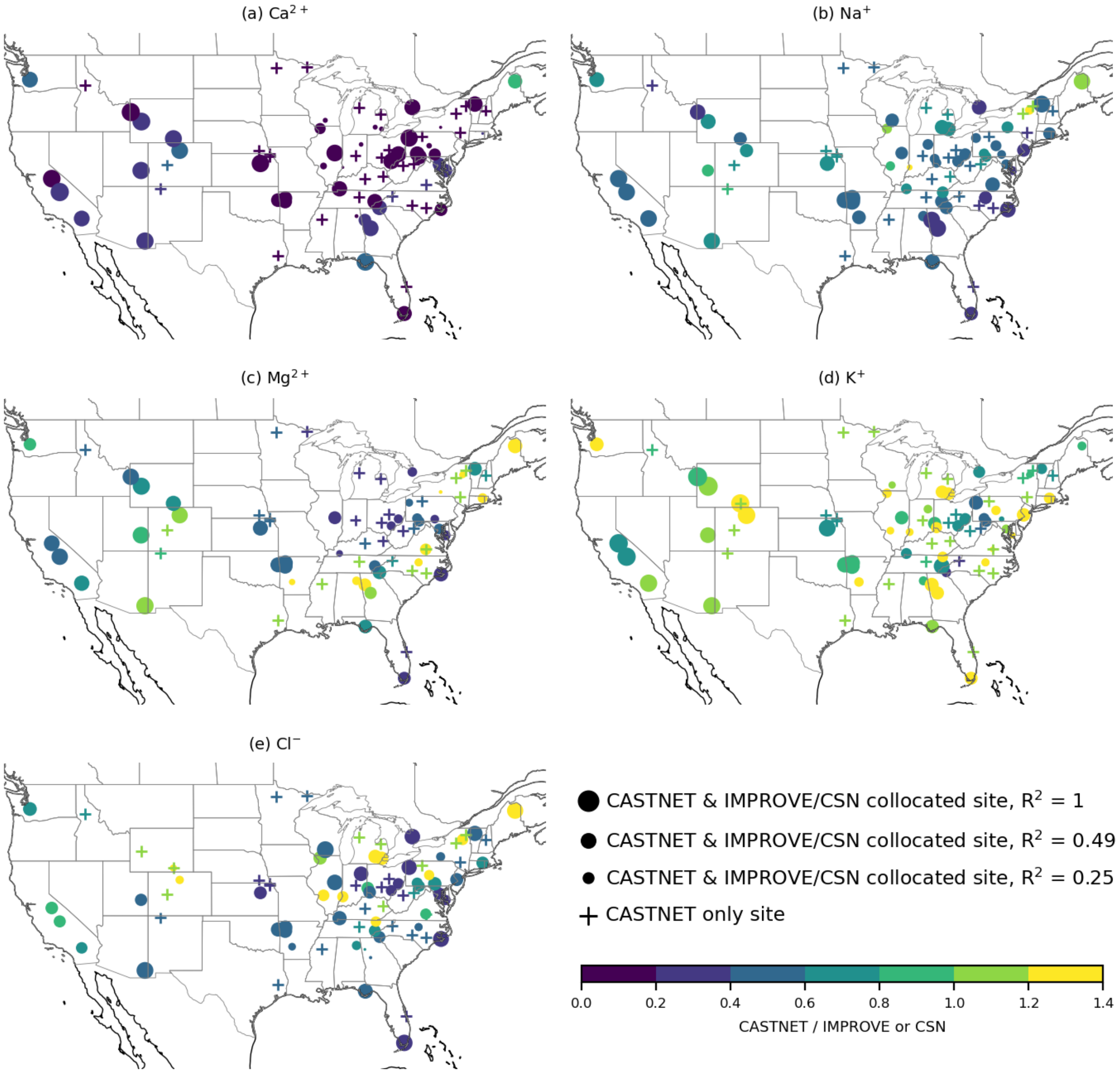

Supplementary Figure 20. Ratios of CASTNET and IMPROVE/EPA CSN NVC and Cl- observations (circles) and interpolated values for sites without IMPROVE/EPA CSN sites (crosses). Sizes of the circle represent the determination coefficient (R^2). The base map is obtained from Natural Earth.

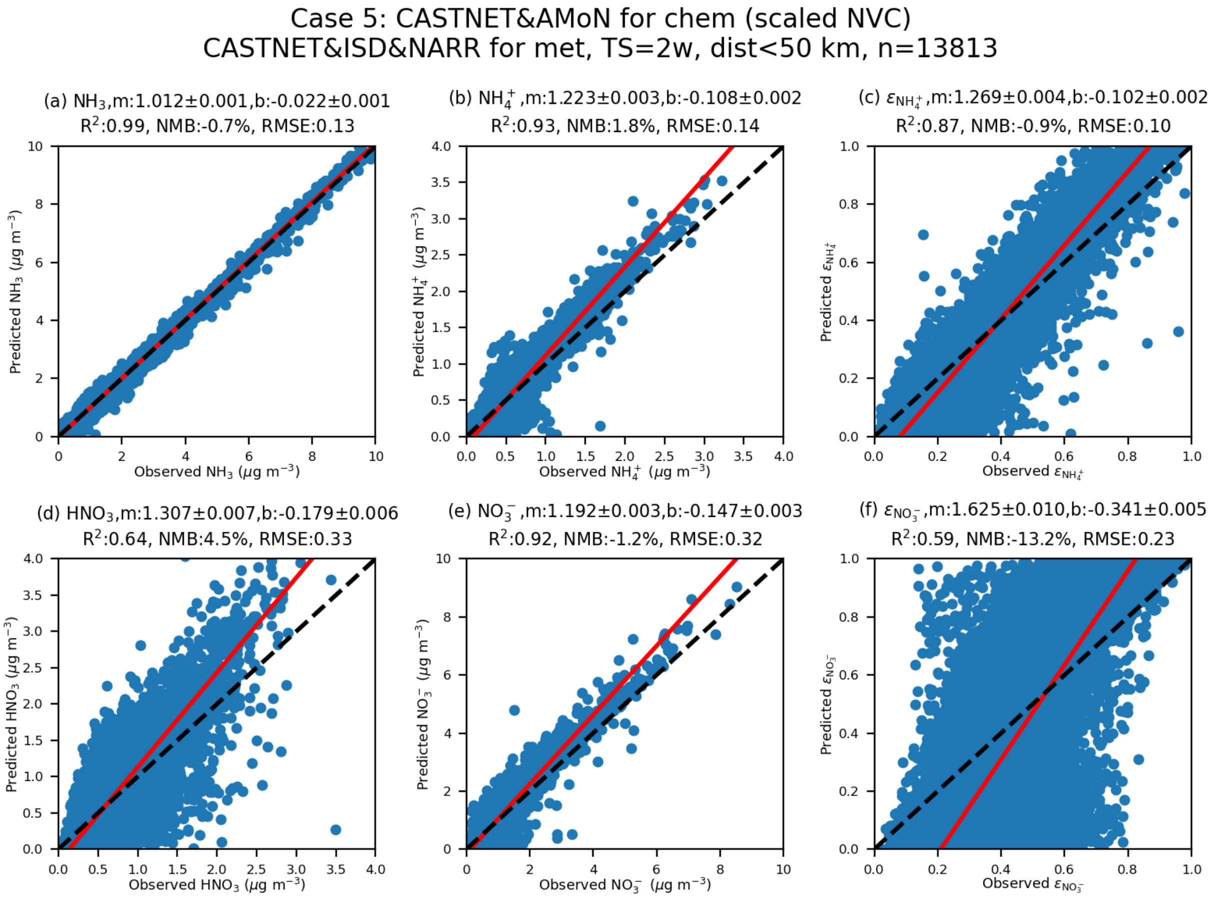

Supplementary Figure 21. Same as Supplementary Fig. 18 but for Case 5. The slope and offset uncertainties are the 95% CI of the regression (±1.96SD). Black dashed lines show the 1:1 line. The number of samples is 13813 for all panels.

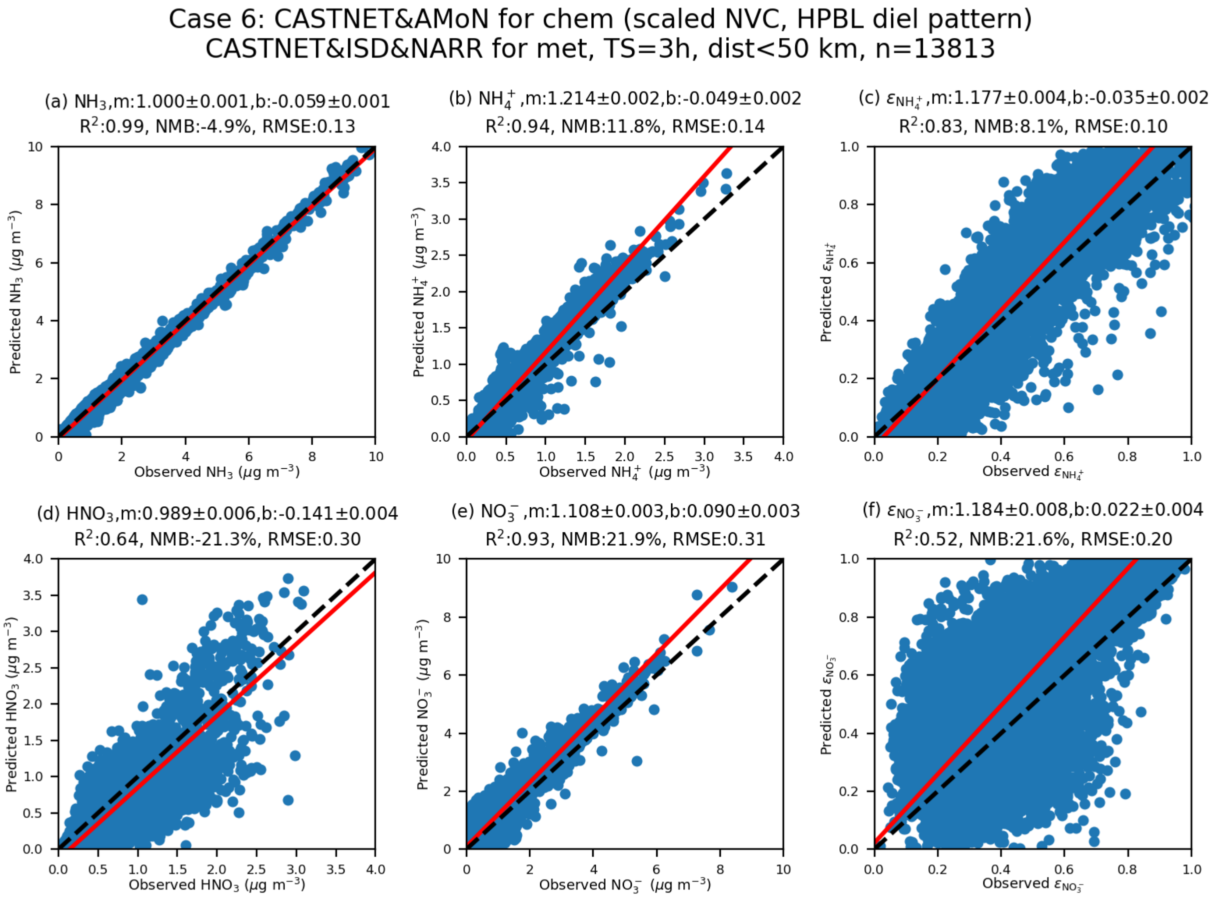

Supplementary Figure 22. Same as Supplementary Fig. 18 but for Case 6. The slope and offset uncertainties are the 95% CI of the regression (±1.96SD). Black dashed lines show the 1:1 line. The number of samples is 13813 for all panels.

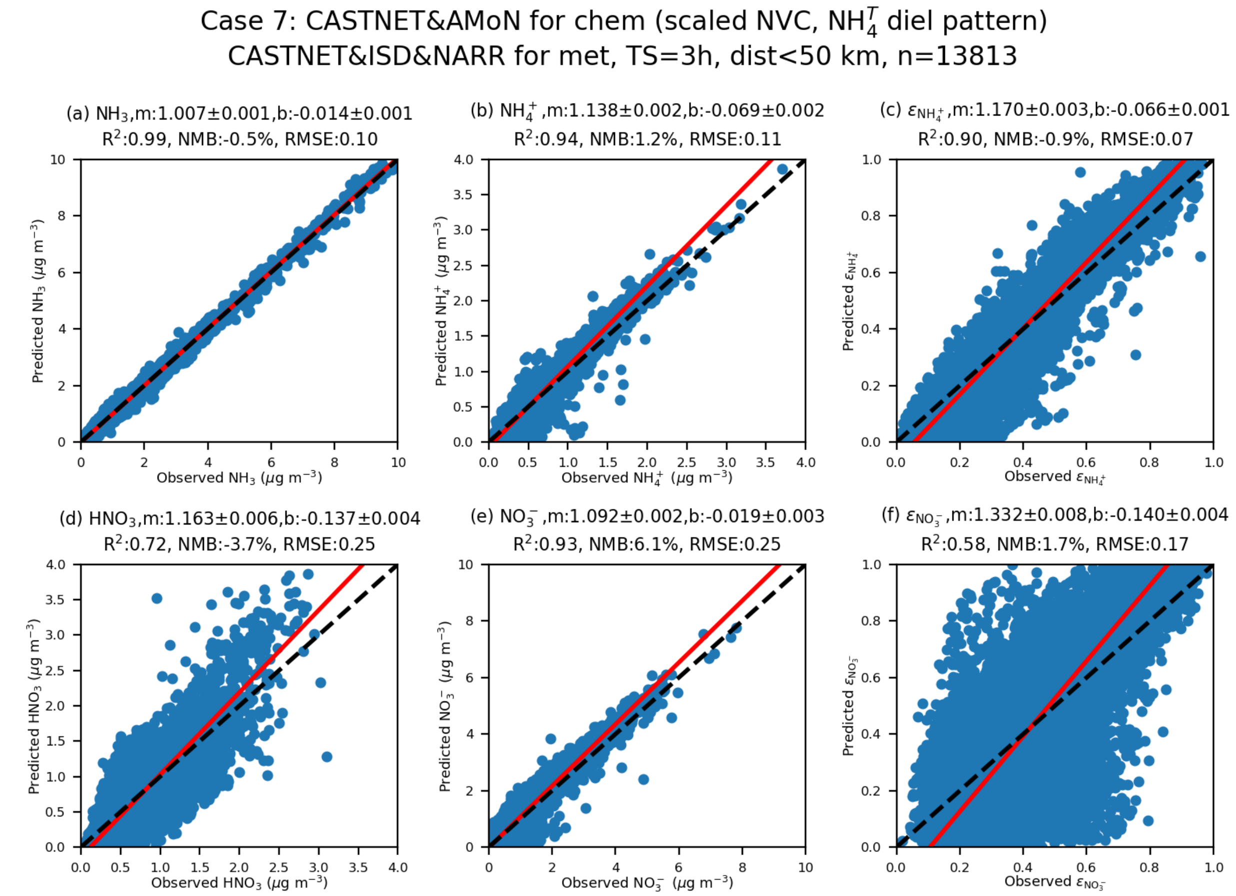

Supplementary Figure 23. Same as Supplementary Fig. 18 but for Case 7. The slope and offset uncertainties are the 95% CI of the regression (±1.96SD). Black dashed lines show the 1:1 line. The number of samples is 13813 for all panels.

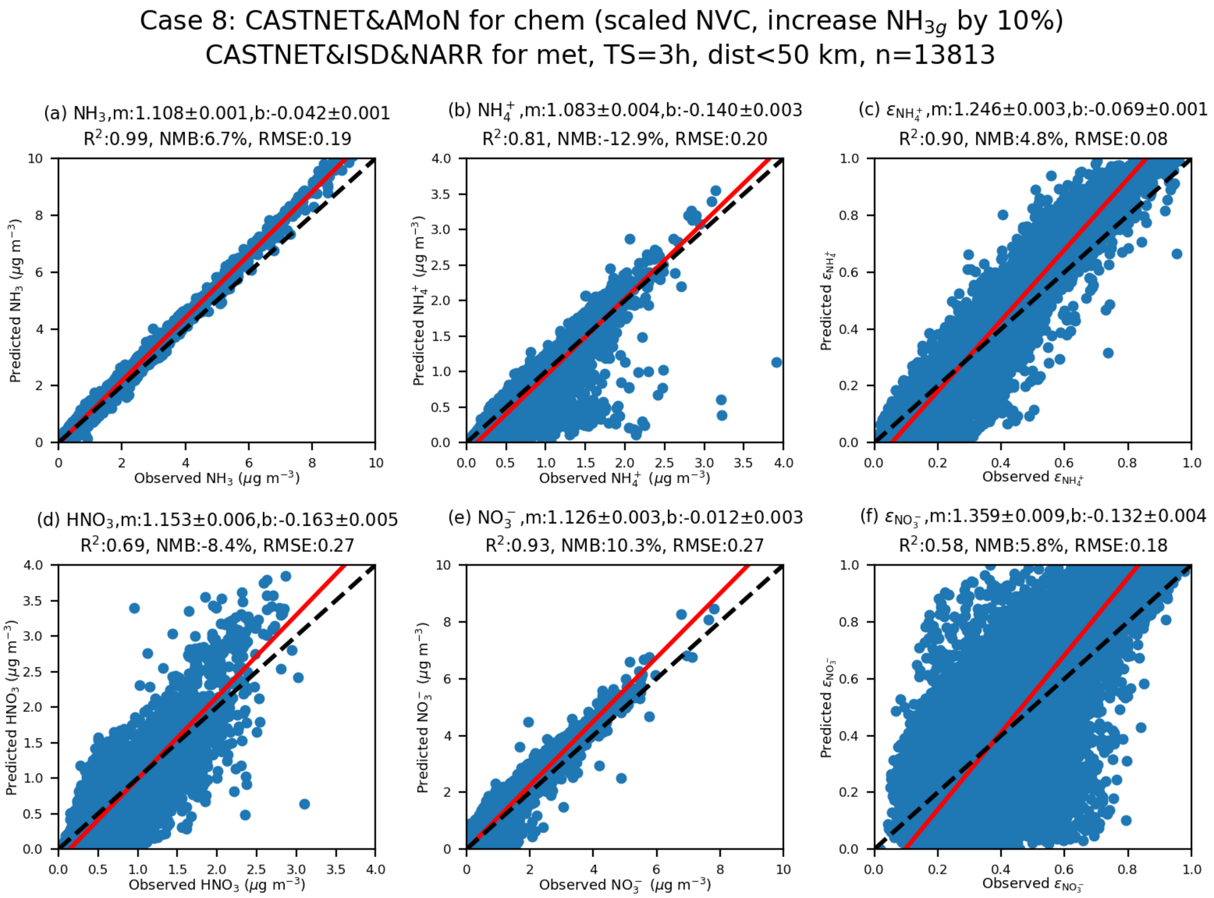

Supplementary Figure 24. Same as Supplementary Fig. 18 but for Case 8. The slope and offset uncertainties are the 95% CI of the regression (±1.96SD). Black dashed lines show the 1:1 line. The number of samples is 13813 for all panels.

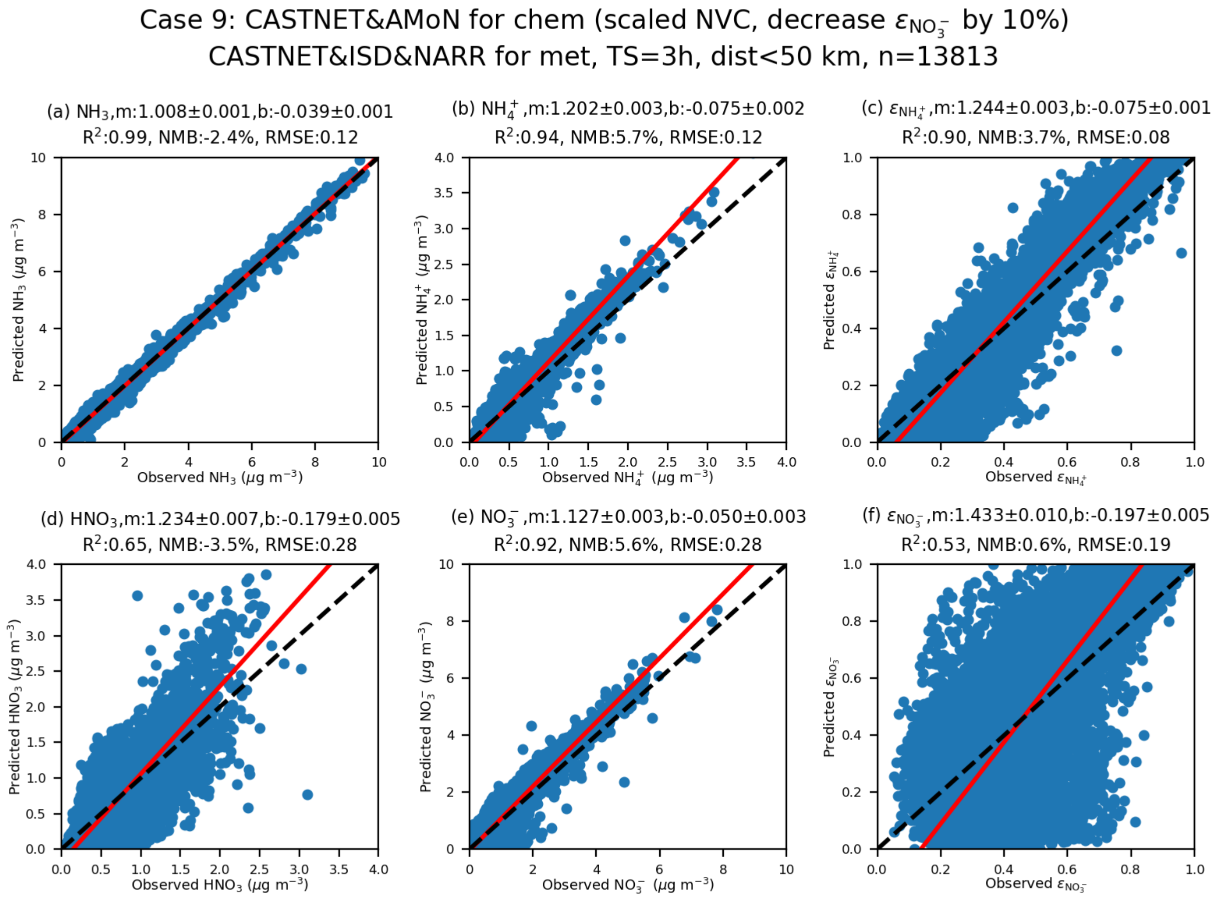

Supplementary Figure 25. Same as Supplementary Fig. 18 but for Case 9. The slope and offset uncertainties are the 95% CI of the regression (±1.96SD). Black dashed lines show the 1:1 line. The number of samples is 13813 for all panels.

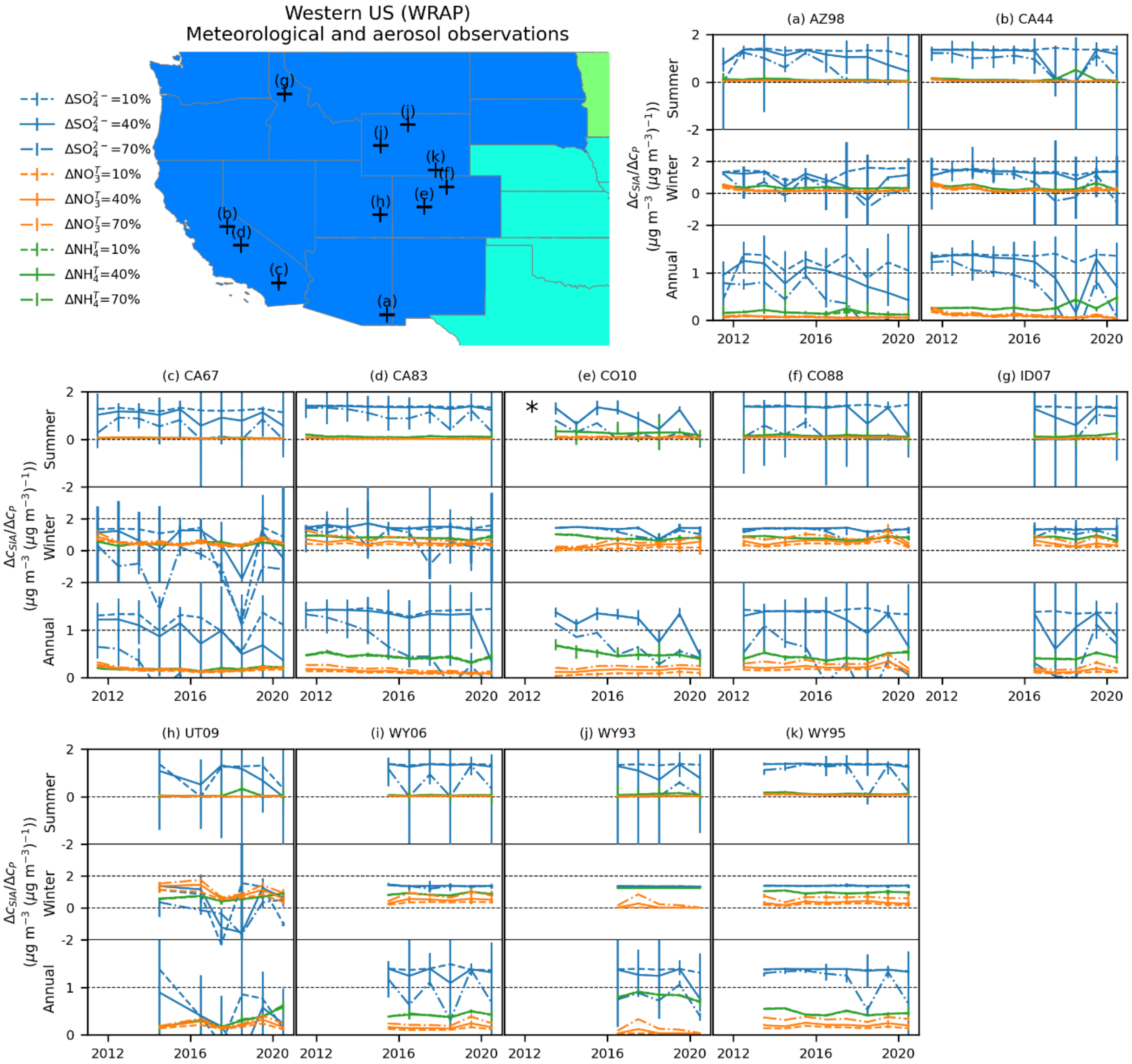

Supplementary Figure 26. Simulated $\Delta c_{\mathrm{SIA}}/\Delta c_{\mathrm{SO_4^{2-}}}$, $\Delta c_{\mathrm{SIA}}/\Delta c_{\mathrm{NO_3^T}}$, and $\Delta c_{\mathrm{SIA}}/\Delta c_{\mathrm{NH_4^T}}$ from sites within the Western US (WRAP). Lines and bars represent the mean values and the 95% CI (as the 2.5th to the 97.5th percentiles) of the corresponding variables derived from 500 Monte Carlo simulations. The base map is obtained from Natural Earth.

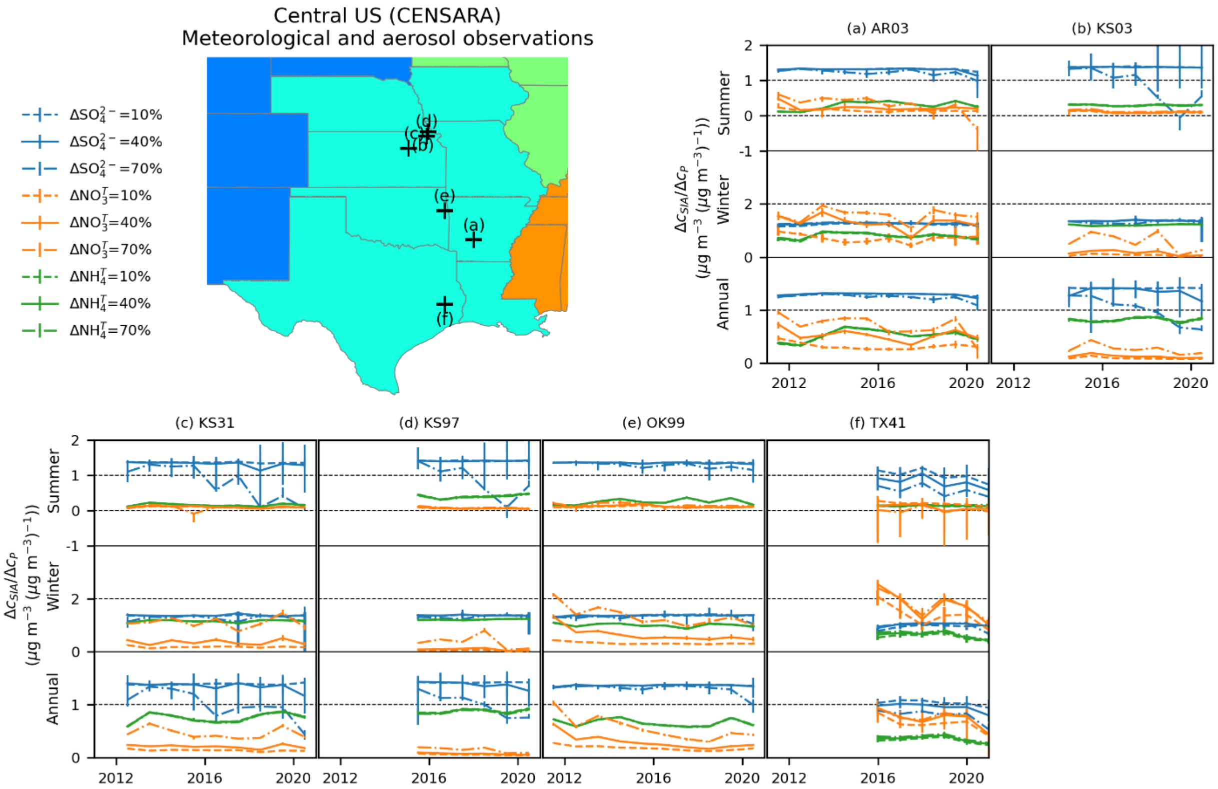

Supplementary Figure 27. Same as Supplementary Fig. 26 but for the Central US (CENSARA). Lines and bars represent the mean values and the 95% CI (as the 2.5th to the 97.5th percentiles) of the corresponding variables derived from 500 Monte Carlo simulations. The base map is obtained from Natural Earth.

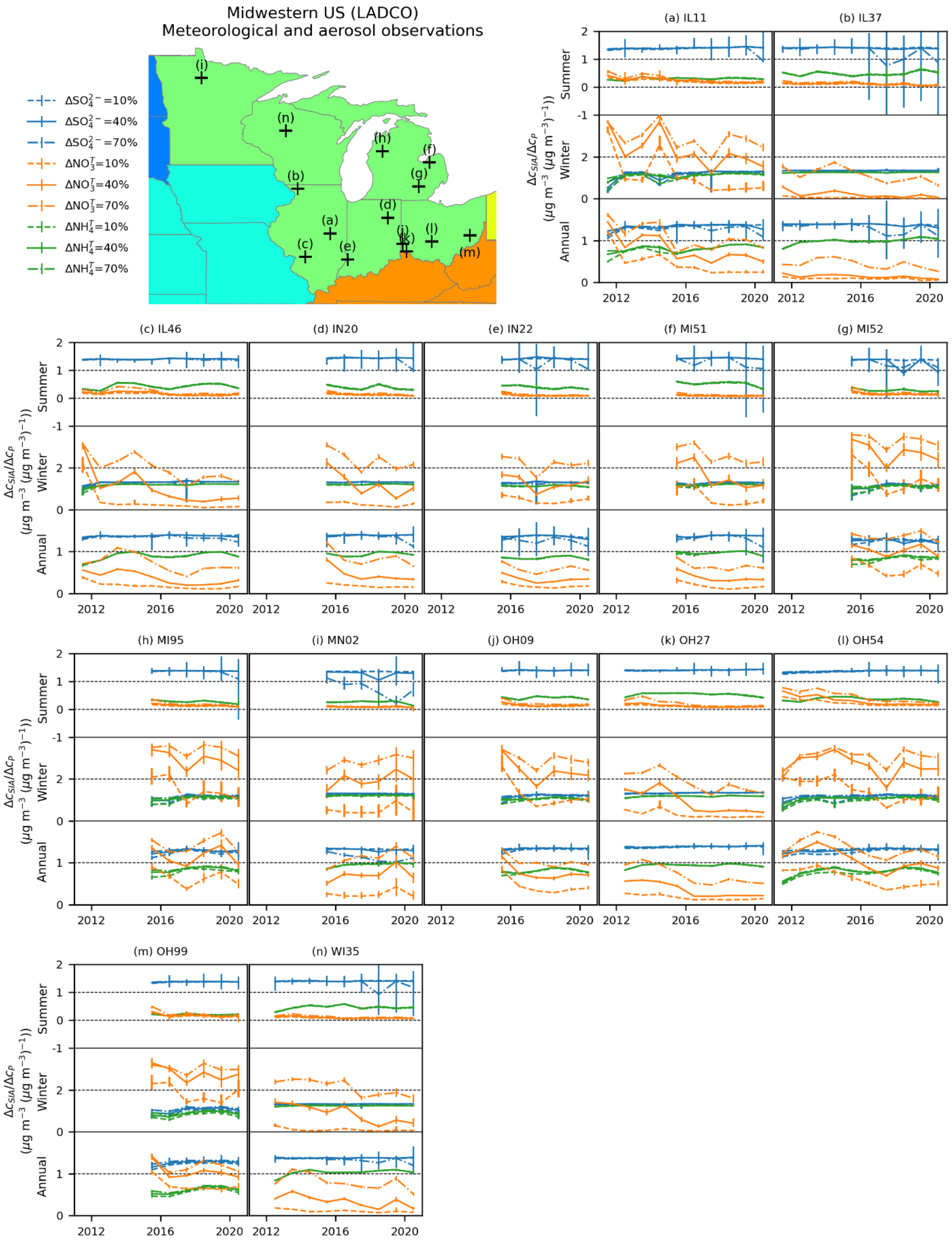

Supplementary Figure 28. Same as Supplementary Fig. 26 but for the Midwestern US (LADCO). Lines and bars represent the mean values and the 95% CI (as the 2.5th to the 97.5th percentiles) of the corresponding variables derived from 500 Monte Carlo simulations. The base map is obtained from Natural Earth.

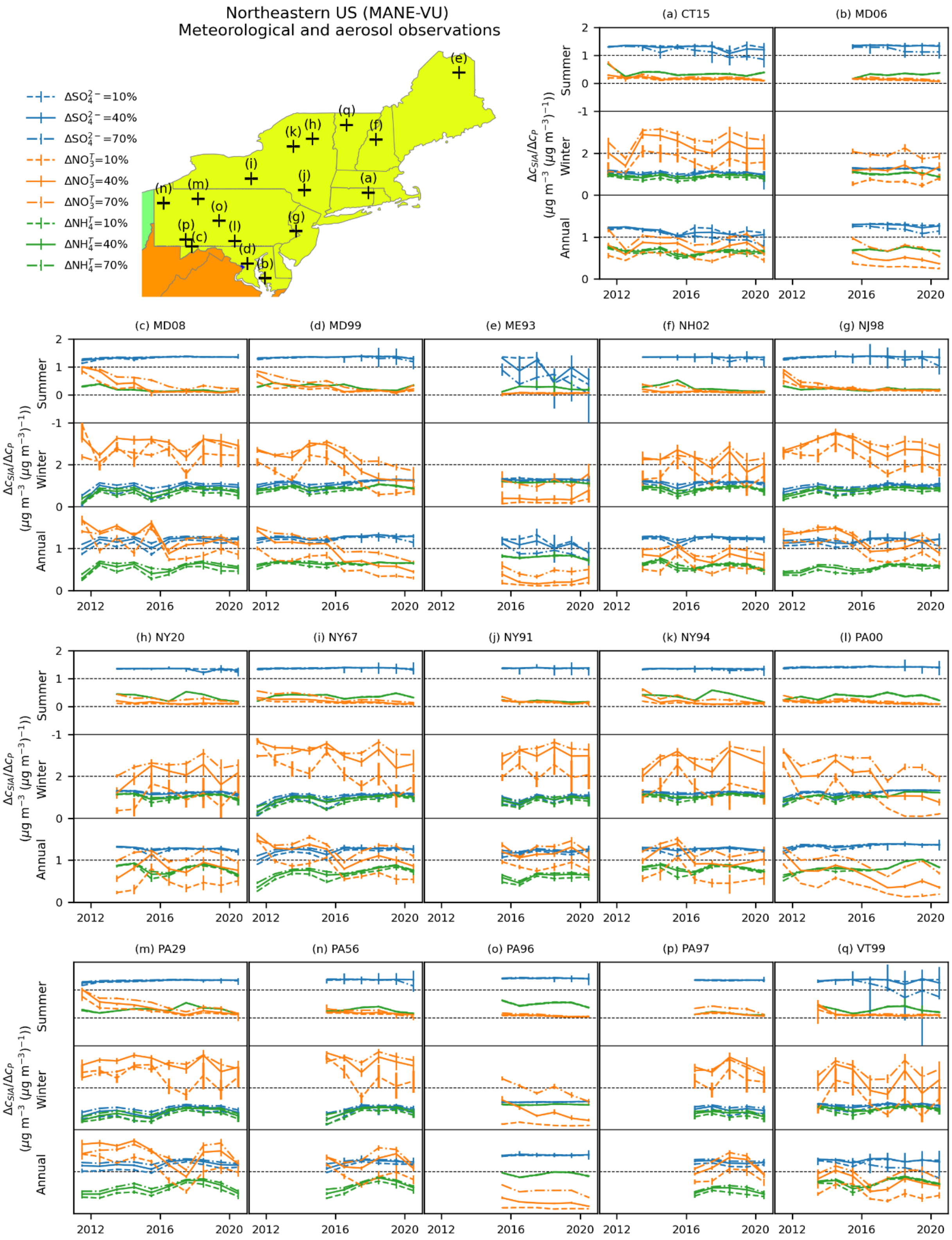

Supplementary Figure 29. Same as Supplementary Fig. 26 but for the Northeastern US (MANE-VU). Lines and bars represent the mean values and the 95% CI (as the 2.5th to the 97.5th percentiles) of the corresponding variables derived from 500 Monte Carlo simulations. The base map is obtained from Natural Earth.

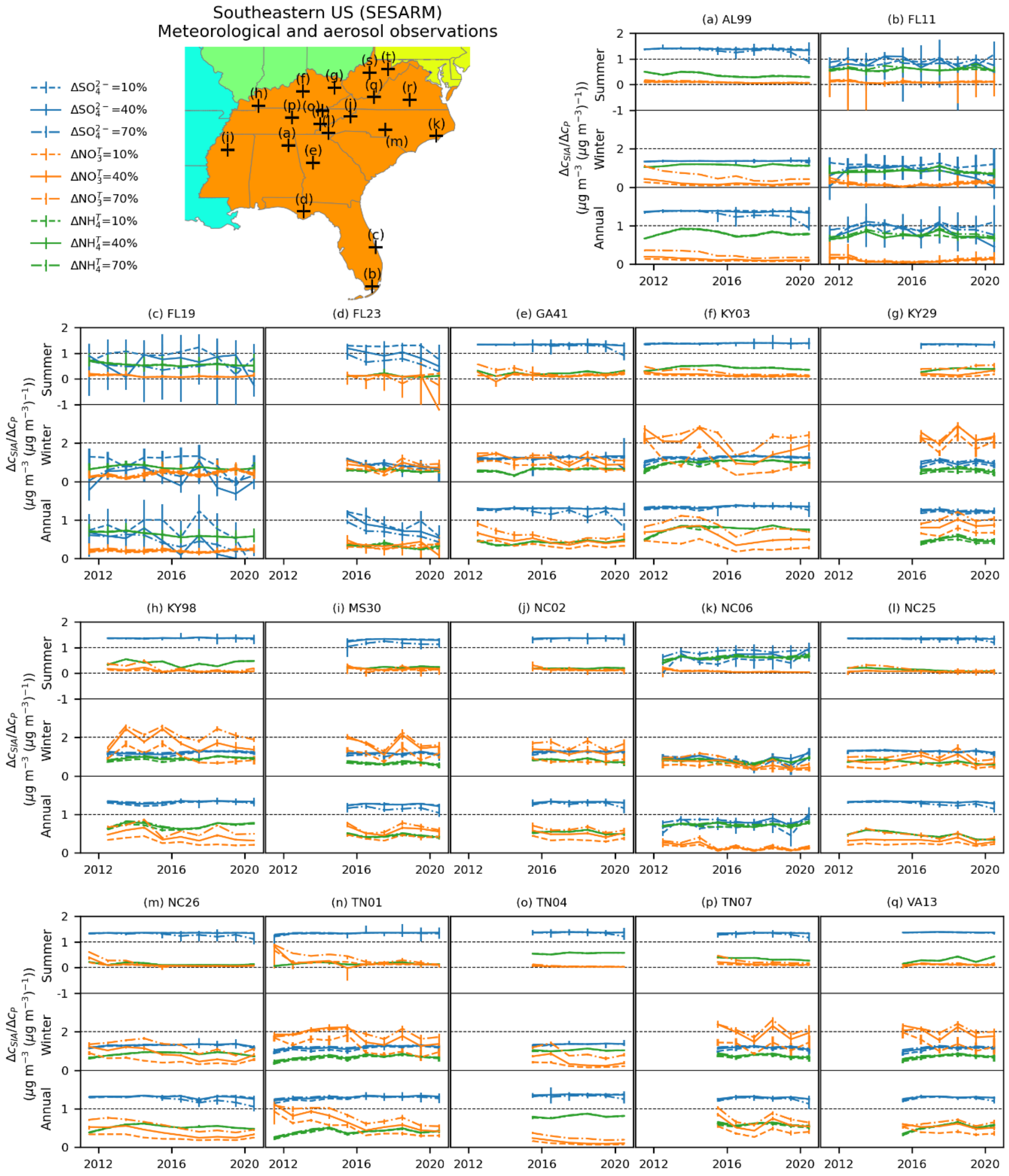

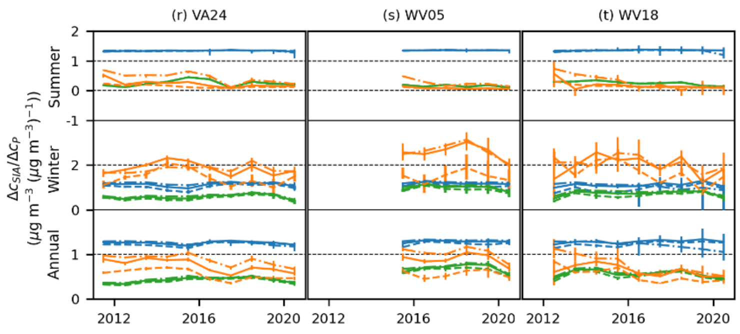

Supplementary Figure 30. Same as Supplementary Fig. 26 but for the Southeastern US (SESARM). Lines and bars represent the mean values and the 95% CI (as the 2.5th to the 97.5th percentiles) of the corresponding variables derived from 500 Monte Carlo simulations. The base map is obtained from Natural Earth.

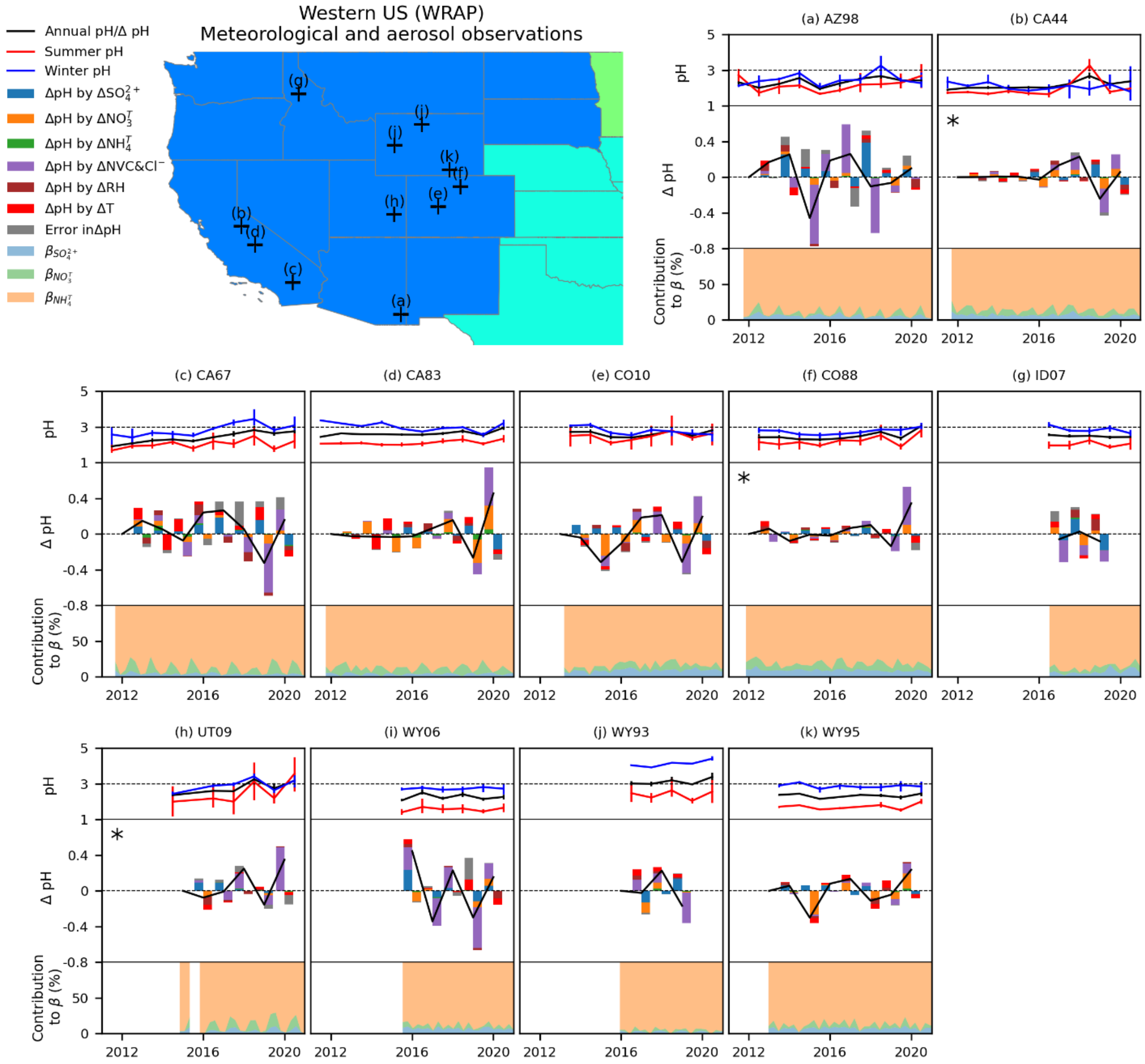

Supplementary Figure 31. ISORROPIA-II Simulated aerosol acidity related properties from sites within the Western US (WRAP). The top panels of each site plot show annual, summer, and winter pH, and lines and bars represent the mean values and the 95% CI (as the 2.5th to the 97.5th percentiles) derived from 1000 Monte Carlo simulations. The middle panels show ΔpH caused by changes in SO_4^{2-}, NO_3^T, NH_4^T, non-volatile ions and Cl^-, relative humidity (RH), temperature, and errors of the multivariable Taylor series method. The bottom panels show buffering capacities of SO_4^{2-}, NO_3^T, and NH_4^T. Vertical bars in the top panels are the 95% CI (as the 2.5th and the 97.5th percentiles) of the corresponding variables derived from 1000 Monte Carlo simulations. "*" indicates that ΔpH values are scaled by 0.5 to be fitted in the panel. The base map is obtained from Natural Earth.

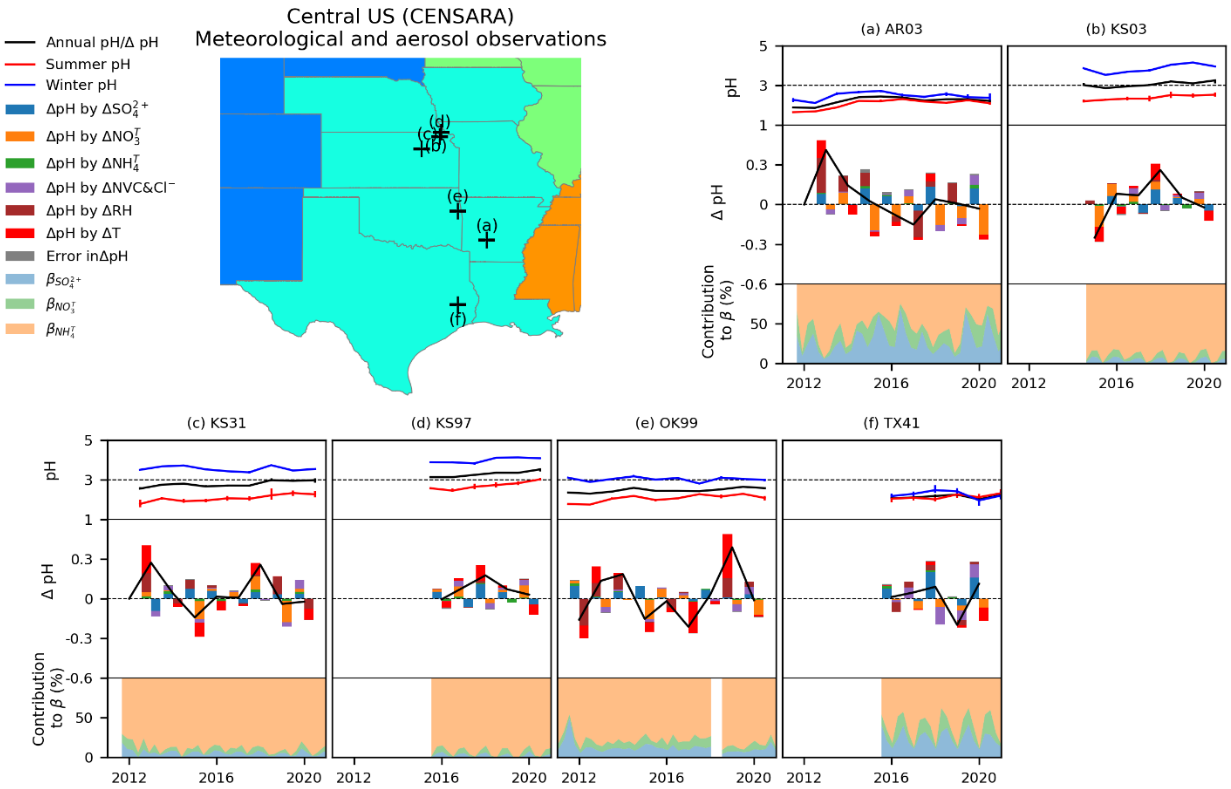

Supplementary Figure 32. Same as Supplementary Fig. 31 but for the Central US (CENSARA). The top panels of each site plot show annual, summer, and winter pH, and lines and bars represent the mean values and the 95% CI (as the 2.5th to the 97.5th percentiles) derived from 1000 Monte Carlo simulations. The base map is obtained from Natural Earth.

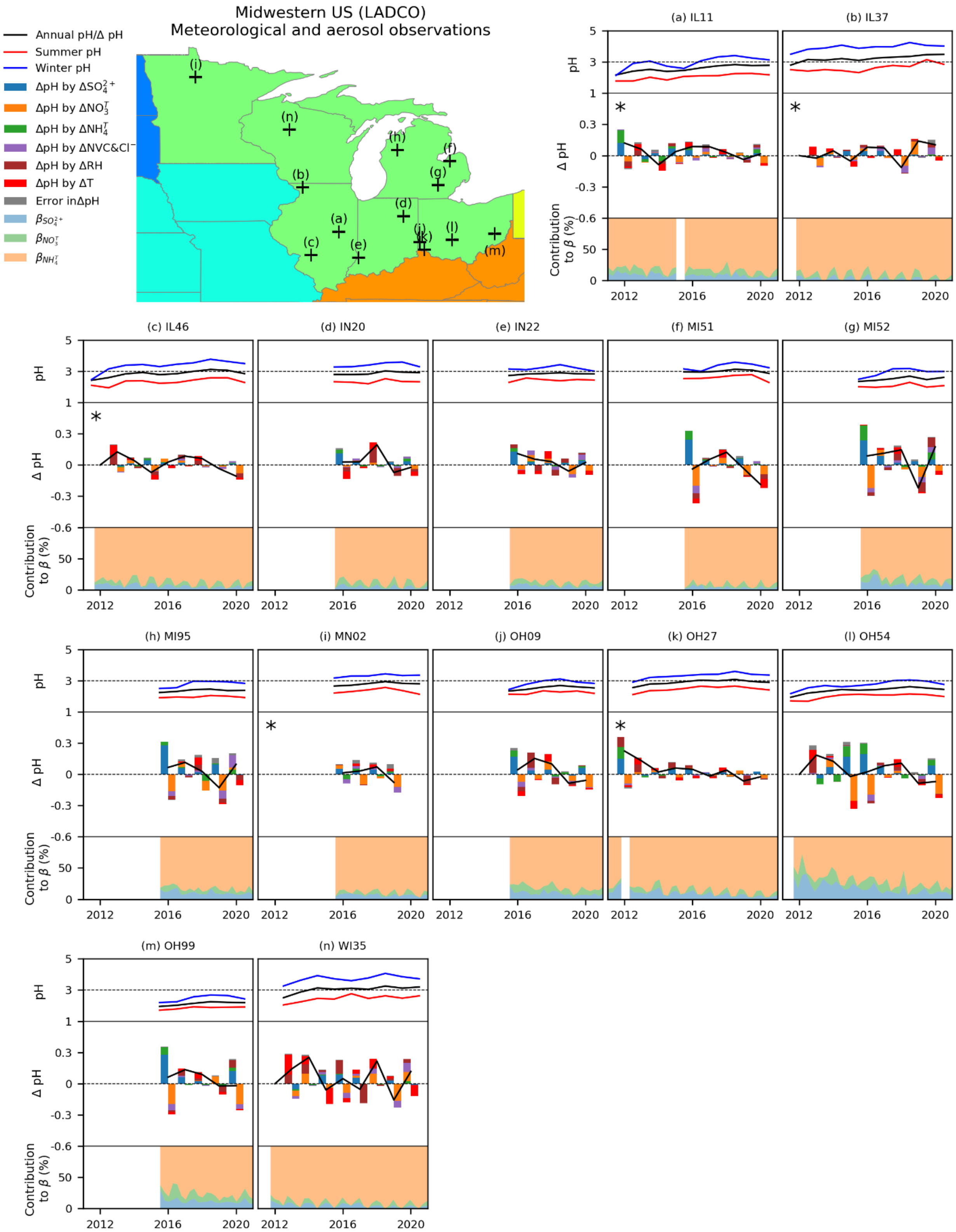

Supplementary Figure 33. Same as Supplementary Fig. 31 but for the Midwestern US (LADCO). The top panels of each site plot show annual, summer, and winter pH, and lines and bars represent the mean values and the 95% CI (as the 2.5th to the 97.5th percentiles) derived from 1000 Monte Carlo simulations. The base map is obtained from Natural Earth.

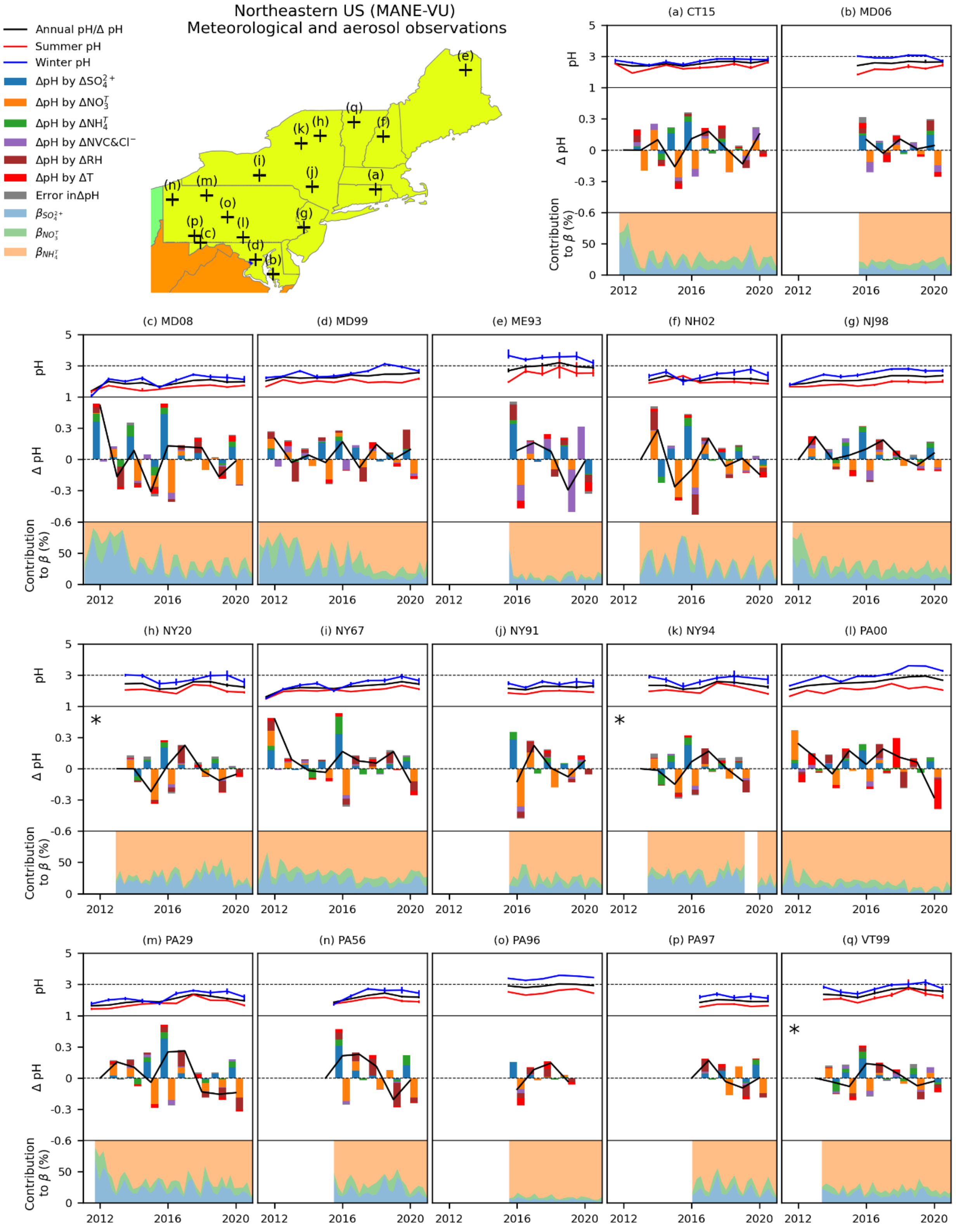

Supplementary Figure 34. Same as Supplementary Fig. 31 but for the Northeastern US (MANE-VU). The top panels of each site plot show annual, summer, and winter pH, and lines and bars represent the mean values and the 95% CI (as the 2.5th to the 97.5th percentiles) derived from 1000 Monte Carlo simulations. The base map is obtained from Natural Earth.

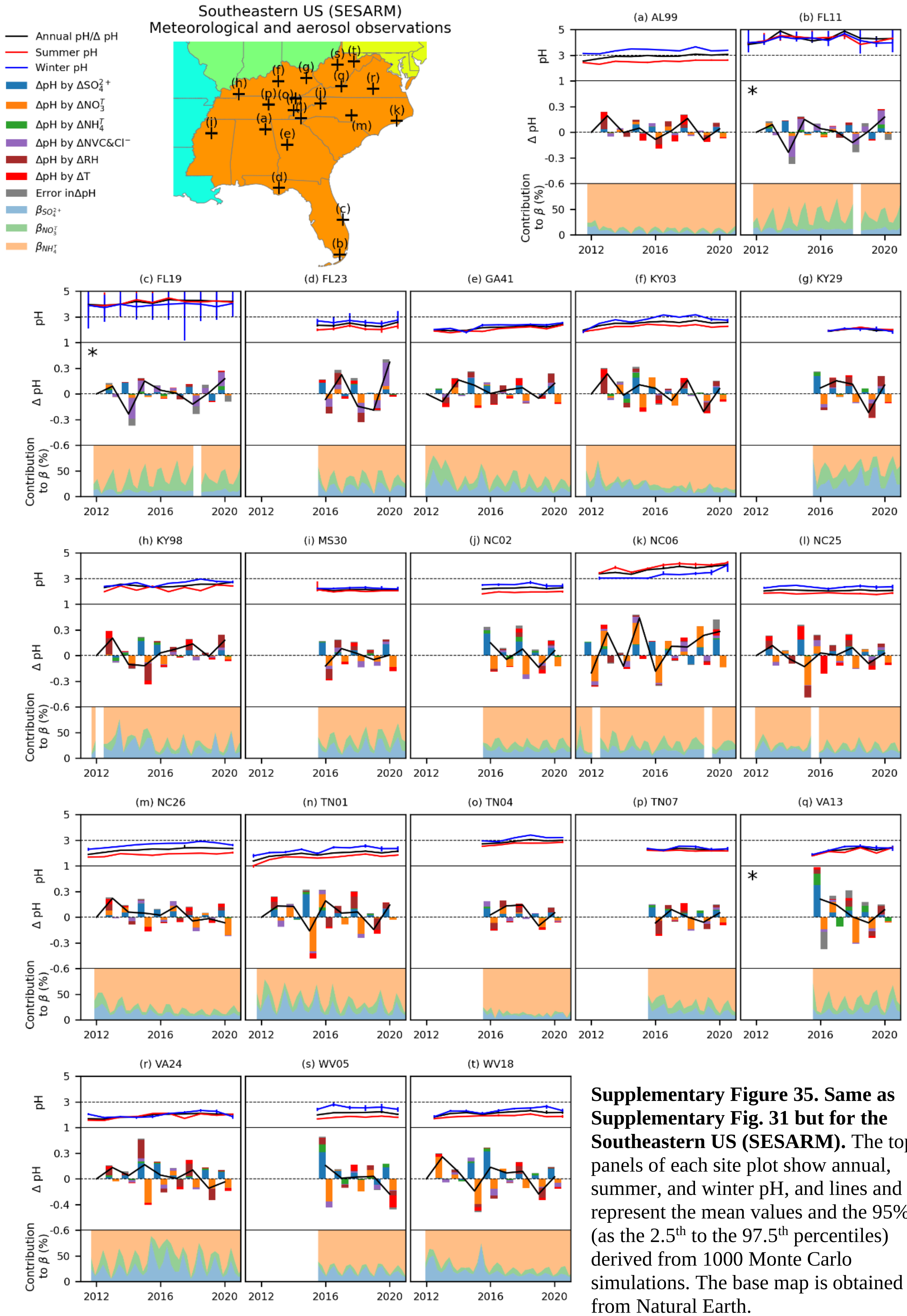

Supplementary Figure 35. Same as Supplementary Fig. 31 but for the Southeastern US (SESARM). The top panels of each site plot show annual, summer, and winter pH, and lines and bars represent the mean values and the 95% CI (as the 2.5th to the 97.5th percentiles) derived from 1000 Monte Carlo simulations. The base map is obtained from Natural Earth.

Supplementary Tables

Supplementary Table 1. Observations available at each site. Sites are named using AMoN site IDs. The first symbol in the paratheses (except for NARR) is the site name within the corresponding network. The number following the network name shows the distance of the site or the grid center for NARR to the AMoN site of the site.

Site (AMoN)	HNO_3, PM Composition, RH, and T	$PM_{2.5}$ Composition	RH and T	Land type[a]	Start date	End date
AL99	CAST(SND152,0.1)	CSN(131150003,59.5)	ISD(999999-63857,0.8)|NARR(12.5)	A	2011-03	N.A.
AR03	CAST(CAD150,0.0)	CSN(51190007,98.7)	ISD(720394-00428,9.4)|NARR(2.6)	F	2011-03	N.A.
AR09	CAST(CHE185,73.7)	IMP(STIL1,73.7)|CSN(400019009,73.7)	ISD(723445-93993,50.7)|NARR(11.0)	A	2015-10	N.A.
AR15	CAST(CHE185,76.7)	IMP(STIL1,76.7)|CSN(400019009,76.7)	ISD(723445-93993,30.0)|NARR(10.0)	A	2015-10	N.A.
AZ98	CAST(CHA467,0.0)	IMP(CHIR1,0.0)	ISD(999999-03085,0.0)|NARR(14.1)	OR	2011-03	N.A.
CA44	CAST(YOS404,0.3)	IMP(YOSE1,0.0)	NARR(18.9)	F	2011-03	N.A.
CA67	CAST(JOT403,0.2)	IMP(JOSH1,0.0)	NARR(13.0)	D	2011-03	N.A.
CA83	CAST(SEK430,0.0)	IMP(SEQU1,0.6)	NARR(7.7)	Unk	2011-03	N.A.
CO10	CAST(GTH161,0.2)	N.A.	ISD(724673-93009,65.2)|NARR(14.4)	OR	2012-09	N.A.
CO88	CAST(ROM206,0.1)	IMP(ROMO1,0.1)	NARR(15.8)	Unk	2011-05	N.A.
CT15	CAST(ABT147,0.1)	N.A.	ISD(725084-54767,18.1)|NARR(15.7)	U/A	2011-03	N.A.
FL11	CAST(EVE419,0.4)	IMP(EVER1,0.1)|CSN(120110034,85.1)	ISD(998297-99999,19.5)|NARR(8.7)	F/M	2011-03	N.A.
FL19	CAST(IRL141,0.0)	N.A.	ISD(994951-99999,1.8)|NARR(16.3)	M	2011-04	N.A.
FL23	CAST(SUM156,0.6)	IMP(SAMA1,79.8)|CSN(120730012,71.8)	ISD(997992-99999,37.9)|NARR(15.1)	F	2015-01	N.A.
GA40	CAST(SND152,94.2)	IMP(ATLA1,74.7)|CSN(130890002,74.7)	ISD(720714-00252,9.8)|NARR(11.4)	A	2011-12	2017-01
GA41	CAST(GAS153,0.5)	IMP(ATLA1,57.5)|CSN(130890002,57.5)	ISD(720966-00339,13.6)|NARR(7.7)	A	2011-06	N.A.
ID07	CAST(NPT006,0.1)	N.A.	ISD(726873-00452,37.7)|NARR(13.7)	Unk	2015-12	N.A.
IL11	CAST(BVL130,0.2)	IMP(BOND1,0.1)|CSN(170191001,0.1)	ISD(999999-54808,0.1)|NARR(14.6)	A	2007-10	N.A.
IL37	CAST(STK138,0.0)	CSN(191630015,97.2)	ISD(722204-04996,28.2)|NARR(20.9)	A	2011-04	N.A.
IL46	CAST(ALH157,0.0)	CSN(171190024,49.0)	ISD(722972-63878,33.0)|NARR(10.3)	A	2011-03	N.A.
IN20	CAST(SAL133,16.8)	CSN(180650003,92.5)	ISD(725330-14827,26.1)|NARR(13.1)	A	2015-01	N.A.
IN22	CAST(VIN140,0.1)	CSN(180372001,62.0)	ISD(725342-13809,10.8)|NARR(5.4)	A	2015-01	N.A.
KS03	CAST(KIC003,15.9)	N.A.	ISD(725533-94957,11.4)|NARR(5.9)	P	2014-02	N.A.
KS31	CAST(KNZ184,0.0)	IMP(TALL1,74.4)|CSN(200170001,74.4)	ISD(999999-53974,0.1)|NARR(22.0)	P	2011-03	N.A.
KS97	CAST(KIC003,10.6)	N.A.	ISD(725533-94957,35.7)|NARR(8.4)	P	2015-01	N.A.
KY03	CAST(MCK131,0.1)	N.A.	ISD(720448-00144,28.3)|NARR(20.5)	A	2011-03	N.A.
KY29	CAST(CKT136,0.0)	N.A.	ISD(720458-00476,42.2)|NARR(13.8)	A	2015-01	N.A.
KY98	CAST(CDZ171,0.2)	IMP(CADI1,0.0)	ISD(746710-13806,35.2)|NARR(11.6)	A	2011-03	N.A.
MD06	CAST(BWR139,0.0)	IMP(WASH1,93.4)|CSN(100010003,94.2)	ISD(722218-00356,11.9)|NARR(19.1)	F/M	2015-01	N.A.
MD08	CAST(LRL117,37.6)	IMP(FRRE1,0.1)|CSN(240230002,0.1)	ISD(725293-99999,31.3)|NARR(19.0)	F	2010-08	N.A.
MD99	CAST(BEL116,0.0)	IMP(WASH1,25.3)|CSN(240330030,6.1)	ISD(724065-99999,8.1)|NARR(14.4)	OR	2010-08	N.A.
ME93	CAST(ASH135,0.1)	IMP(PRIS1,30.8)|CSN(230031020,30.8)	ISD(727130-14604,29.1)|NARR(20.8)	A	2015-01	N.A.
MI51	CAST(UVL124,0.0)	N.A.	ISD(722007-54828,18.5)|NARR(20.5)	A	2015-01	N.A.
MI52	CAST(ANA115,0.0)	IMP(DETR1,60.7)|CSN(261630001,60.7)	ISD(725378-04887,24.6)|NARR(15.7)	F	2015-02	N.A.
MI95	CAST(HOX148,0.0)	N.A.	ISD(726384-14817,28.1)|NARR(17.9)	F	2015-01	N.A.
MI96	CAST(ANA115,60.6)	IMP(DETR1,2.5)|CSN(261630001,2.5)	ISD(725370-94847,11.0)|NARR(12.0)	F	2007-10	N.A.
MN02	CAST(RED004,0.0)	N.A.	ISD(727486-04983,40.0)|NARR(11.9)	F	2015-01	N.A.
MS30	CAST(CVL151,0.0)	N.A.	ISD(720541-53806,48.1)|NARR(16.0)	F	2015-01	N.A.
NC02	CAST(PNF126,0.2)	N.A.	NARR(8.9)	F	2015-01	N.A.
NC06	CAST(BFT142,0.1)	IMP(SWAN1,73.3)	ISD(723037-93765,17.2)|NARR(15.0)	A	2010-04	2018-08
NC25	CAST(COW137,0.0)	IMP(GRSM1,78.8)|CSN(370210034,97.8)	ISD(720259-63844,18.1)|NARR(13.1)	F	2011-05	N.A.
NC26	CAST(CND125,0.1)	N.A.	ISD(721038-99999,14.8)|NARR(6.4)	F	2011-04	N.A.
NH02	CAST(WST109,0.2)	N.A.	ISD(742078-64773,18.7)|NARR(14.8)	F	2012-06	N.A.
NJ98	CAST(WSP144,0.1)	N.A.	ISD(724095-14792,6.2)|NARR(8.3)	OR	2011-03	N.A.
NM98	CAST(MEV405,86.1)	N.A.	ISD(724625-93005,38.3)|NARR(17.9)	F	2008-01	N.A.
NY20	CAST(HWF187,0.0)	CSN(360310003,55.0)	ISD(726228-94740,45.8)|NARR(6.0)	F	2012-06	N.A.
NY67	CAST(CTH110,0.4)	N.A.	ISD(725155-94761,18.2)|NARR(16.4)	F	2007-10	N.A.
NY91	CAST(CAT175,0.1)	N.A.	NARR(14.0)	F	2015-01	N.A.
NY94	CAST(NIC001,0.6)	N.A.	ISD(726228-94740,99.7)|NARR(8.3)	F	2012-11	2018-09
OH02	CAST(QAK172,97.2)	IMP(QUCI1,97.2)	ISD(720397-00131,14.1)|NARR(14.2)	A	2007-10	N.A.
OH09	CAST(OXF122,0.1)	CSN(391351001,33.9)	ISD(725217-53855,25.2)|NARR(14.0)	A	2015-01	N.A.
OH27	CAST(OXF122,46.0)	CSN(390610040,2.6)	ISD(724297-93812,9.9)|NARR(13.2)	A	2007-10	N.A.
OH54	CAST(DCP114,0.2)	N.A.	ISD(724284-63825,31.3)|NARR(13.2)	A	2011-03	N.A.

OH99	CAST(QAK172,0.0)	IMP(QUCI1,0.0)|CSN(540511002,51.6)	ISD(724286-93824,47.2)|NARR(14.3)	A	2015-01	N.A.
OK99	CAST(CHE185,0.0)	IMP(STIL1,0.0)|CSN(400019009,0.0)	ISD(722199-53956,36.0)|NARR(15.1)	A	2007-10	N.A.
PA00	CAST(ARE128,0.0)	N.A.	ISD(722826-93762,23.7)|NARR(13.2)	A	2009-10	N.A.
PA29	CAST(KEF112,0.1)	N.A.	ISD(725266-04751,25.1)|NARR(7.1)	F	2011-03	N.A.
PA56	CAST(MKG113,0.2)	N.A.	ISD(725104-04843,22.9)|NARR(8.5)	F	2014-12	N.A.
PA96	CAST(PSU106,0.0)	N.A.	ISD(725128-54739,15.9)|NARR(5.4)	A	2015-01	N.A.
PA97	CAST(LRL117,0.1)	IMP(FRRE1,37.5)|CSN(240230002,37.5)	ISD(725127-04726,50.9)|NARR(10.8)	F	2015-07	N.A.
TN01	CAST(GRS420,0.2)	IMP(GRSM1,0.1)	ISD(720259-63844,65.8)|NARR(20.8)	F	2011-03	N.A.
TN04	CAST(SPD111,0.1)	CSN(470931020,50.3)	ISD(720353-63875,17.6)|NARR(12.6)	A	2015-01	N.A.
TN07	CAST(ESP127,0.1)	N.A.	ISD(723274-00372,18.3)|NARR(14.0)	F	2015-01	N.A.
TX41	CAST(ALC188,40.5)	N.A.	ISD(725021-99999,50.0)|NARR(13.4)	Unk	2015-01	N.A.
UT09	CAST(CAN407,0.0)	IMP(CANY1,0.0)	ISD(999999-53004,96.5)|NARR(15.5)	D	2014-05	N.A.
VA13	CAST(VPI120,0.1)	N.A.	ISD(724125-03859,57.2)|NARR(12.9)	A	2015-01	N.A.
VA24	CAST(PED108,0.1)	CSN(510870014,91.2)	ISD(724017-03707,24.4)|NARR(8.4)	F	2011-03	N.A.
VT99	CAST(UND002,0.0)	IMP(PMRF1,0.0)|CSN(500070007,0.0)	ISD(726114-54771,20.2)|NARR(11.8)	F	2012-11	N.A.
WI06	CAST(STK138,96.0)	CSN(550270001,80.7)	ISD(720656-99999,11.7)|NARR(17.9)	A	2019-02	N.A.
WI35	CAST(PRK134,0.0)	CSN(551198001,0.1)	ISD(726417-54911,25.9)|NARR(2.8)	A	2011-03	N.A.
WV05	CAST(CDR119,0.0)	CSN(540390011,87.0)	ISD(720366-63879,27.3)|NARR(2.4)	F	2015-01	N.A.
WV18	CAST(PAR107,0.1)	N.A.	ISD(724170-13729,27.8)|NARR(7.5)	F	2011-06	N.A.
WY06	CAST(PND165,0.8)	IMP(BOLA1,15.1)	ISD(720522-00443,69.3)|NARR(15.3)	F	2015-01	N.A.
WY92	CAST(YEL408,96.8)	IMP(YELL2,96.8)	ISD(726664-94173,93.9)|NARR(10.7)	A	2019-10	N.A.
WY93	CAST(BAS601,0.0)	N.A.	ISD(726667-24048,26.6)|NARR(15.6)	P	2015-06	N.A.
WY95	CAST(CNT169,0.8)	IMP(MOZI1,98.9)	NARR(20.4)	F	2012-06	N.A.

[a] Land types: "A"=Agricultural; "F"=Forest; "OR"=Open range; "D"=Desert; "M"=Marsh; "P"=Prairie; and "Unk"=Unknown.

Supplementary Table 2. Region definition, information, and sites within each region. Mean [5th, 95th percentiles] population densities and emission rates in the regions and near the sites, and annual number of biweekly samples within each region are listed. Sites are named after AMoN IDs. Population (2015) and emission (2017) data are obtained from the Gridded Population of the World, Version 4 (GPWv4, ~30 km×30 km) [28] and EPA's Air Quality Time Series Project Data(EQUATES, 12 km × 12 km) [2].

**Region**	**RPO**	**Sites**	**Regional mean population density (pop, persons km^{-2}) and emission rates (ton km^{-2} yr^{-1})**	**Mean population density (pop, persons km^{-2}) and emission rates (ton km^{-2} yr^{-1}) within 50 km of the sites**	**Year**	**Number of samples[1]**
Western US	Western Regional Air Partnership (WRAP)	AZ98, CA44, CA67, CA83, CO10, CO88, ID07, UT09, WY06, WY93, WY95	Pop: 32.6 [3×10^{-3}, 51.6]<br>SO_2: 0.11 [2×10^{-6}, 0.15]<br>NO_x: 0.55 [3×10^{-4}, 2.00]<br>NH_3: 0.36 [1×10^{-2}, 1.33]	Pop: 27.6 [3×10^{-3}, 73.3]<br>SO_2: 0.06 [6×10^{-6}, 0.16]<br>NO_x: 0.41 [1×10^{-4}, 1.63]<br>NH_3: 0.45 [6×10^{-3}, 0.96]	2011<br>2012<br>2013<br>2014<br>2015<br>2016<br>2017<br>2018<br>2019<br>2020	97<br>156<br>182<br>200<br>239<br>279<br>288<br>286<br>278<br>279
Central US	Central States Air Resource Agencies (CENSARA)	AR03, KS03, KS31, KS97, OK99, TX41	Pop: 33.2 [0.03, 99.2]<br>SO_2: 0.45 [6×10^{-5}, 0.35]<br>NO_x: 1.19 [0.02, 4.10]<br>NH_3: 0.81 [0.05, 2.53]	Pop: 18.7 [3×10^{-3}, 73.3]<br>SO_2: 0.15 [4×10^{-3}, 0.34]<br>NO_x: 0.95 [0.10, 2.68]<br>NH_3: 0.73 [0.12, 1.82]	2011<br>2012<br>2013<br>2014<br>2015<br>2016<br>2017<br>2018<br>2019<br>2020	64<br>79<br>71<br>102<br>156<br>156<br>150<br>150<br>146<br>151
Midwestern US	Lake Michigan Air Directors Consortium (LADCO)	IL11, IL37, IL46, IN20, IN22, MI51, MI52, MI95, MN02, OH09, OH27, OH54, OH99, WI35	Pop: 66.3 [0.3, 292.3]<br>SO_2: 0.43 [4×10^{-4}, 0.39]<br>NO_x: 1.56 [0.04, 6.11]<br>NH_3: 0.73 [0.02, 2.13]	Pop: 82.9 [0.5, 488.7]<br>SO_2: 0.73 [4×10^{-4}, 0.44]<br>NO_x: 2.03 [0.03, 8.79]<br>NH_3: 0.65 [0.03, 1.58]	2011<br>2012<br>2013<br>2014<br>2015<br>2016<br>2017<br>2018<br>2019<br>2020	116<br>157<br>156<br>149<br>356<br>364<br>365<br>362<br>364<br>363
Northeastern US	Mid-Atlantic/Northeast Visibility Union (MANE-VU)	CT15, MD06, MD08, MD99, ME93, NH02, NJ98, NY20, NY67, NY91, NY94, PA00, PA29, PA56, PA96, PA97, VT99	Pop: 159.3 [0.2, 689.5]<br>SO_2: 0.30 [4×10^{-4}, 0.53]<br>NO_x: 1.93 [0.02. 7.92]<br>NH_3: 0.35 [0.02, 1.13]	Pop: 141.2 [0.1, 746.4]<br>SO_2: 0.21 [3×10^{-4}, 0.36]<br>NO_x: 1.83 [0.03, 8.61]<br>NH_3: 0.32 [0.02, 1.05]	2011<br>2012<br>2013<br>2014<br>2015<br>2016<br>2017<br>2018<br>2019<br>2020	164<br>228<br>285<br>285<br>421<br>442<br>433<br>435<br>433<br>441
Southeastern US	Southeastern Air Pollution Control Agencies (SESARM)	AL99, FL19, FL23, GA41, KY03, KY29, KY98, MS30, NC02, NC06, NC25, NC26, TN01, TN04, TN07, VA13, VA24, WV05, WV18	Pop: 78.1 [0.6, 375.3]<br>SO_2: 0.42 [2×10^{-3}, 0.32]<br>NO_x: 1.64 [0.07, 6.60]<br>NH_3: 0.63 [0.08, 1.78]	Pop: 59.2 [0.7, 236.7]<br>SO_2: 0.21 [$9\times^{-4}$, 0.21]<br>NO_x: 1.22 [0.07, 4.29]<br>NH_3: 0.85 [0.06, 2.80]	2011<br>2012<br>2013<br>2014<br>2015<br>2016<br>2017<br>2018<br>2019<br>2020	208<br>316<br>303<br>304<br>498<br>519<br>503<br>503<br>513<br>520

Supplementary Table 3. Case studies of pre-processing of integrated observations for ISORROPIA simulations. The impacts of coarse mode NVCs and Cl^- and diel variations of meteorological and chemical conditions on ISORROPIA-II performance are investigated. $c_{SO_4^{2-}}$, $c_{NO_3^T}$, and $c_{NH_4^T}$ observations from CASTNET and AMoN and T and RH observations from CASTNET, ISD, and NARR are used for all cases. Case 1 is the default configuration for all analyses in this study. Case 2 represents the results using raw inputs from the integrated observations. Case 3 and 4 show that including coarse mode components leads to overestimation of $c_{NH_4^+}$ and $c_{NO_3^-}$. Cases 5 - 7 illustrate the importance of diel patterns of meteorological conditions, boundary layer height, and $c_{NH_4^T}$, respectively. Case 8 and 9 show that sampling biases have low impacts on model evaluation.

Case	Species	m	δ_m	b	δ_b	R^2	NMB	RMSE
Case 1 (default): Scaled CASTNET NVC and Cl^-; TS=3hr; *n*=13813	NH_4^+	1.202	0.003	-0.075	0.002	0.941	5.8%	0.123
	NO_3^-	1.118	0.003	-0.021	0.003	0.931	8.4%	0.262
	HNO_3	1.164	0.006	-0.156	0.005	0.699	-6.3%	0.261
	NH_3	1.008	0.001	-0.039	0.001	0.993	-2.4%	0.116
	$\varepsilon_{NH_4^+}$	1.243	0.003	-0.075	0.001	0.898	3.8%	0.084
	$\varepsilon_{NO_3^-}$	1.355	0.008	-0.141	0.004	0.581	3.7%	0.177
Case 2: IMPROVE/CSN NVC and Cl^-; TS=3hr; *n*=4424	NH_4^+	1.103	0.007	-0.017	0.004	0.832	6.6%	0.164
	NO_3^-	1.094	0.004	-0.004	0.005	0.937	8.7%	0.241
	HNO_3	1.127	0.008	-0.129	0.007	0.793	-4.6%	0.240
	NH_3	1.019	0.001	-0.057	0.003	0.991	-2.1%	0.155
	$\varepsilon_{NH_4^+}$	1.253	0.010	-0.091	0.004	0.834	-1.5%	0.101
	$\varepsilon_{NO_3^-}$	1.274	0.014	-0.103	0.006	0.618	-2.2%	0.169
Case 3: Raw CASTNET NVC and Cl^-; TS=2wk; *n*=13813	NH_4^+	1.100	0.004	-0.212	0.002	0.865	-26.7%	0.218
	NO_3^-	1.217	0.003	-0.081	0.003	0.933	8.4%	0.321
	HNO_3	1.231	0.007	-0.210	0.006	0.629	-6.5%	0.321
	NH_3	1.060	0.001	0.085	0.002	0.990	11.2%	0.206
	$\varepsilon_{NH_4^+}$	1.242	0.005	-0.206	0.002	0.825	-27.5%	0.153
	$\varepsilon_{NO_3^-}$	1.669	0.009	-0.296	0.004	0.662	-1.5%	0.210
Case 4: Raw CASTNET NVC and Cl^-; TS=3hr; *n*=13813	NH_4^+	1.072	0.003	-0.181	0.002	0.888	-24.2%	0.192
	NO_3^-	1.155	0.002	0.046	0.003	0.942	19.2%	0.301
	HNO_3	1.054	0.006	-0.176	0.005	0.663	-18.4%	0.291
	NH_3	1.057	0.001	0.071	0.001	0.992	10.3%	0.182
	$\varepsilon_{NH_4^+}$	1.196	0.004	-0.171	0.002	0.861	-24.4%	0.130
	$\varepsilon_{NO_3^-}$	1.367	0.007	-0.079	0.004	0.654	16.1%	0.182
Case 5: Scaled CASTNET NVC and Cl^-; TS=2wk; *n*=13813	NH_4^+	1.223	0.003	-0.108	0.002	0.928	1.8%	0.137
	NO_3^-	1.192	0.003	-0.147	0.003	0.917	-1.2%	0.321
	HNO_3	1.307	0.007	-0.179	0.006	0.642	4.5%	0.328
	NH_3	1.012	0.001	-0.022	0.001	0.990	-0.7%	0.129
	$\varepsilon_{NH_4^+}$	1.269	0.004	-0.102	0.002	0.872	-0.9%	0.095
	$\varepsilon_{NO_3^-}$	1.625	0.010	-0.341	0.005	0.593	-13.2%	0.228
Case 6: Scaled CASTNET NVC and Cl^-; diel pattern ∝ Planetary boundary layer height from NARR; TS=3hr; *n*=13813	NH_4^+	1.214	0.002	-0.049	0.002	0.945	11.8%	0.137
	NO_3^-	1.108	0.003	0.090	0.003	0.927	21.9%	0.308
	HNO_3	0.989	0.006	-0.141	0.004	0.640	-21.3%	0.297
	NH_3	1.000	0.001	-0.059	0.001	0.992	-4.9%	0.129
	$\varepsilon_{NH_4^+}$	1.177	0.004	-0.035	0.002	0.829	8.1%	0.104
	$\varepsilon_{NO_3^-}$	1.184	0.008	0.022	0.004	0.517	21.6%	0.197
Case 7: Scaled CASTNET NVC and Cl^-; NH_4^T diel pattern; TS=3hr; *n*=13813	NH_4^+	1.138	0.002	-0.069	0.002	0.939	1.2%	0.109
	NO_3^-	1.092	0.002	-0.019	0.003	0.931	6.1%	0.247
	HNO_3	1.163	0.006	-0.137	0.004	0.723	-3.7%	0.247
	NH_3	1.007	0.001	-0.014	0.001	0.994	-0.5%	0.103
	$\varepsilon_{NH_4^+}$	1.170	0.003	-0.066	0.001	0.896	-0.9%	0.075
	$\varepsilon_{NO_3^-}$	1.332	0.008	-0.140	0.004	0.582	1.7%	0.173
Case 8: Scaled CASTNET NVC and Cl^-; NH_3 increased by 10%; TS=3hr; *n*=13813	NH_4^+	1.083	0.004	-0.140	0.003	0.809	-12.9%	0.205
	NO_3^-	1.126	0.003	-0.012	0.003	0.930	10.3%	0.270
	HNO_3	1.153	0.006	-0.163	0.005	0.685	-8.4%	0.267
	NH_3	1.108	0.001	-0.042	0.001	0.994	6.7%	0.194
	$\varepsilon_{NH_4^+}$	1.246	0.003	-0.069	0.001	0.903	4.8%	0.082
	$\varepsilon_{NO_3^-}$	1.359	0.009	-0.132	0.004	0.576	5.8%	0.180
Case 9: Scaled CASTNET NVC and Cl^-; decrease $\varepsilon_{NO_3^-}$ for high T and low RH (T>20 °C and RH<60%) periods; TS=3hr; *n*=13813	NH_4^+	1.202	0.003	-0.075	0.002	0.941	5.7%	0.122
	NO_3^-	1.127	0.003	-0.050	0.003	0.920	5.6%	0.275
	HNO_3	1.234	0.007	-0.179	0.005	0.652	-3.5%	0.277
	NH_3	1.008	0.001	-0.039	0.001	0.993	-2.4%	0.116
	$\varepsilon_{NH_4^+}$	1.244	0.003	-0.075	0.001	0.897	3.7%	0.084
	$\varepsilon_{NO_3^-}$	1.433	0.010	-0.197	0.005	0.531	0.6%	0.187

Supplementary Table 4. Region trends derived from Theil-Sen regressions and Mann-Kendall tests. Trends of observed concentrations (c), aerosol composition (f), pH, partitionings of NH_4^+ and NO_3^- ($\varepsilon_{NH_4^+}$ and $\varepsilon_{NO_3^-}$), and $\Delta c_{SIA}/\Delta c_p$ (p=SO_4^{2-}, NO_3^T, NH_4^T). "*" and "**" indicate the trends are $p<0.05$ and $p<0.01$, respectively. The annual changing rates are the median annual changing rate of the sites within a region. "W US", "C US", "MW US", "NE US", "SE US" stand for the Western, Central, Midwestern, Northeastern, Southeastern US, respectively. Annual changing rates are normalized to the first values of the period. Long-term sites refer to the sites with data for 2011 – 20201. Trend analyses for 2011 – 2020 using data from all sites and long-term sites are consistent.

		All sites (n=41), 2011 – 2015		All sites (n=68), 2015 – 2020		All sites (n=68), 2011 – 2020		Long-term sites (n=41), 2011 – 2020	
**Species**	**Region**	**Trend**	**Annual rate (% yr^{-1})**	**Trend**	**Annual rate (% yr^{-1})**	**Trend**	**Annual rate (% yr^{-1})**	**Trend**	**Annual rate (% yr^{-1})**
$c_{SO_4^{2-}}$	W US	decreasing**	-6.9%	no trend	-0.2%	decreasing**	-3.6%	decreasing**	-3.6%
	C US	decreasing**	-6.8%	decreasing**	-5.7%	decreasing**	-5.3%	decreasing**	-5.4%
	MW US	decreasing**	-4.3%	decreasing**	-5.1%	decreasing**	-6.2%	decreasing**	-6.4%
	NE US	decreasing**	-7.8%	decreasing**	-5.3%	decreasing**	-6.7%	decreasing**	-6.8%
	SE US	decreasing**	-5.8%	decreasing**	-6.8%	decreasing**	-5.9%	decreasing**	-5.7%
$c_{NO_3^T}$	W US	no trend	-3.7%	no trend	0.3%	decreasing**	-1.9%	decreasing**	-2.0%
	C US	no trend	-7.4%	decreasing*	-3.1%	decreasing**	-3.8%	decreasing**	-3.8%
	MW US	no trend	0.0%	decreasing*	-1.2%	decreasing**	-2.3%	decreasing**	-2.4%
	W US	no trend	-1.5%	decreasing*	-1.5%	decreasing**	-3.1%	decreasing**	-3.1%
	SE US	no trend	-1.2%	decreasing**	-5.2%	decreasing**	-3.2%	decreasing**	-3.1%
$c_{NH_4^T}$	W US	increasing*	6.4%	no trend	0.4%	no trend	-1.2%	no trend	-1.2%
	C US	no trend	1.7%	no trend	-1.7%	decreasing**	-1.6%	decreasing**	-1.5%
	MW US	no trend	1.2%	decreasing**	-2.4%	decreasing**	-1.2%	no trend	-0.7%
	W US	no trend	1.6%	decreasing**	-3.5%	decreasing**	-3.4%	decreasing**	-3.4%
	SE US	no trend	-0.5%	decreasing**	-6.3%	decreasing**	-3.7%	decreasing**	-3.3%
c_{NVC} (as Na^+)	W US	no trend	-0.4%	no trend	2.0%	no trend	-0.3%	no trend	0.0%
	C US	no trend	-4.5%	no trend	-2.0%	decreasing*	-1.8%	decreasing*	-2.0%
	MW US	no trend	1.2%	no trend	-0.8%	decreasing**	-1.6%	decreasing**	-1.4%
	W US	increasing**	4.9%	no trend	0.4%	decreasing**	-0.6%	decreasing*	-0.6%
	SE US	decreasing*	-4.0%	decreasing**	-3.3%	decreasing**	-1.7%	decreasing**	-1.4%
c_{Cl^-}	W US	no trend	-2.7%	no trend	-0.3%	no trend	0.5%	no trend	0.6%
	C US	no trend	-0.6%	no trend	2.8%	no trend	-0.2%	no trend	-0.2%
	MW US	increasing**	10.1%	no trend	-0.3%	no trend	-0.8%	no trend	-0.5%
	W US	increasing**	11.7%	increasing**	2.3%	no trend	0.9%	no trend	1.0%
	SE US	decreasing*	-7.6%	no trend	0.1%	no trend	0.2%	no trend	0.6%
$f_{SO_4^{2-}}$	W US	no trend	-1.3%	decreasing**	-1.5%	decreasing**	-1.3%	decreasing**	-1.4%
	C US	no trend	-0.5%	decreasing**	-2.1%	decreasing**	-1.4%	decreasing**	-1.3%
	MW US	decreasing*	-1.8%	decreasing**	-2.1%	decreasing**	-2.7%	decreasing**	-2.5%
	W US	decreasing**	-2.1%	decreasing**	-1.7%	decreasing**	-2.4%	decreasing**	-2.5%
	SE US	decreasing**	-1.5%	decreasing**	-1.5%	decreasing**	-2.2%	decreasing**	-2.2%
$f_{NO_3^-}$	W US	increasing**	5.0%	no trend	0.5%	increasing**	1.4%	increasing**	1.3%
	C US	increasing*	3.6%	increasing**	2.1%	increasing**	2.3%	increasing**	2.3%
	MW US	no trend	2.7%	increasing**	2.7%	increasing**	3.6%	increasing**	3.5%
	W US	increasing**	7.1%	increasing**	2.6%	increasing**	4.3%	increasing**	4.5%
	SE US	increasing**	5.4%	no trend	1.0%	increasing**	4.2%	increasing**	4.5%
$f_{NH_4^+}$	W US	decreasing**	-1.9%	no trend	0.2%	decreasing**	-1.3%	decreasing**	-1.4%
	C US	no trend	0.1%	no trend	-1.1%	decreasing**	-1.5%	decreasing**	-1.4%
	MW US	no trend	-0.5%	decreasing*	-0.5%	decreasing**	-0.7%	decreasing**	-0.7%
	W US	decreasing**	-1.6%	decreasing**	-0.9%	decreasing**	-1.3%	decreasing**	-1.3%
	SE US	no trend	-0.1%	decreasing**	-1.3%	decreasing**	-1.7%	decreasing**	-1.8%
f_{NVC}	W US	no trend	2.8%	no trend	0.2%	increasing**	2.8%	increasing**	2.8%
	C US	no trend	-1.4%	increasing*	2.5%	increasing**	3.3%	increasing**	3.3%
	MW US	no trend	1.3%	increasing**	3.1%	increasing**	3.9%	increasing**	4.1%
	W US	increasing**	8.6%	increasing**	3.9%	increasing**	5.2%	increasing**	5.3%
	SE US	no trend	0.4%	increasing**	3.0%	increasing**	4.3%	increasing**	4.4%
f_{Cl^-}	W US	no trend	0.6%	no trend	-1.7%	increasing**	3.5%	increasing**	3.6%
	C US	no trend	5.4%	increasing*	3.8%	increasing**	3.4%	increasing**	3.4%

	MW US	increasing**	6.8%	increasing**	2.7%	increasing**	4.0%	increasing**	4.3%
	W US	increasing**	12.0%	increasing**	6.0%	increasing**	6.0%	increasing**	6.1%
	SE US	no trend	-5.7%	increasing**	5.7%	increasing**	4.5%	increasing**	4.5%
pH	W US	increasing*	3.5%	no trend	1.9%	increasing**	1.5%	increasing**	1.6%
	C US	increasing*	6.0%	increasing*	1.4%	increasing**	1.2%	increasing**	1.1%
	MW US	increasing**	4.9%	increasing*	0.8%	increasing**	1.8%	increasing**	2.1%
	W US	increasing*	3.6%	no trend	0.6%	increasing**	1.7%	increasing**	1.8%
	SE US	increasing**	4.9%	no trend	0.5%	increasing**	1.6%	increasing**	1.7%
$\varepsilon_{NH_4^+}$	W US	decreasing**	-9.5%	no trend	0.2%	decreasing**	-1.9%	decreasing**	-2.0%
	C US	no trend	-7.7%	no trend	-1.4%	decreasing**	-3.9%	decreasing**	-3.9%
	MW US	no trend	-2.1%	no trend	-0.9%	decreasing**	-3.5%	decreasing**	-3.8%
	W US	decreasing**	-7.1%	no trend	-0.4%	decreasing**	-3.2%	decreasing**	-3.3%
	SE US	decreasing**	-5.8%	no trend	-1.2%	decreasing**	-3.7%	decreasing**	-4.2%
$\varepsilon_{NO_3^-}$	W US	no trend	0.9%	no trend	1.0%	increasing**	0.7%	increasing**	0.6%
	C US	increasing*	3.9%	increasing**	1.6%	increasing**	1.2%	increasing*	1.1%
	MW US	increasing*	4.2%	increasing**	2.0%	increasing**	1.4%	increasing**	1.0%
	W US	increasing**	6.3%	increasing*	1.6%	increasing**	2.1%	increasing**	2.2%
	SE US	increasing**	5.1%	increasing*	1.0%	increasing**	1.8%	increasing**	1.9%
$\Delta c_{SIA}/\Delta c_{SO_4^{2-}}$	W US	no trend	-0.8%	decreasing**	-5.9%	decreasing**	-3.5%	decreasing**	-3.7%
	C US	no trend	0.8%	decreasing**	-0.8%	decreasing*	-0.2%	no trend	-0.2%
	MW US	no trend	0.2%	no trend	-0.2%	increasing**	0.2%	increasing**	0.2%
	W US	no trend	0.3%	decreasing**	-0.6%	no trend	0.2%	no trend	0.2%
	SE US	no trend	0.4%	decreasing**	-0.5%	no trend	-0.1%	no trend	0.0%
$\Delta c_{SIA}/\Delta c_{NO_3^T}$	W US	no trend	0.4%	no trend	2.3%	no trend	-0.6%	no trend	-0.7%
	C US	no trend	11.5%	no trend	0.1%	no trend	0.1%	no trend	0.3%
	MW US	increasing**	9.3%	increasing**	1.3%	increasing**	1.2%	increasing**	1.3%
	W US	increasing**	8.4%	no trend	0.4%	increasing*	1.0%	increasing*	1.2%
	SE US	increasing**	8.9%	no trend	0.1%	no trend	-0.1%	no trend	-0.2%
$\Delta c_{SIA}/\Delta c_{NH_4^T}$	W US	decreasing**	-9.3%	no trend	0.2%	decreasing**	-2.4%	decreasing**	-2.8%
	C US	no trend	-5.8%	no trend	-1.6%	decreasing**	-2.6%	decreasing**	-3.6%
	MW US	no trend	1.3%	no trend	-0.6%	decreasing**	-5.2%	decreasing**	-6.2%
	W US	no trend	-0.3%	no trend	0.5%	decreasing**	-3.6%	decreasing**	-3.6%
	SE US	no trend	-0.8%	no trend	0.4%	decreasing**	-4.0%	decreasing**	-4.1%

Supplementary Table 5. Literature review of studies that compared effectiveness of SO_2, NO_x, and NH_3 emission abatements on $PM_{2.5}$ reduction.

Notes:
Studies in green clearly stated that controlling NH_3 is the most effective way to reduce $PM_{2.5}$ in the regions investigated in certain seasons.
Studies in yellow indicated that controlling NH_3 is more effective than controlling SO_2 or NO_x in certain regions and certain years.
Studies in red clearly stated that controlling SO_2 or NO_x is more effective than controlling NH_3 in the regions investigated in certain seasons.

Authors (year)	Method	Year analyzed	Region	Species to control/most important species	Sectors to control	Comments
Pinder and Adams (2007) [29]	Chemical transport model and cost analyses	2002	LADCO	NH_3 in winter and SO_2 in summer	Power, industry, transportation, and agriculture	Ammonia control technologies are more cost-effective than controls on SO_2 and NO_x sources at reducing $PM_{2.5}$ in winter.
Tsimipidi et al. (2007) [30]	Chemical transport model	2001 - 2002	Eastern US	SO_2 in summer and NH_3 in winter	N.A.	A uniform 50% reduction in SO_2 emissions was predicted to produce an average decrease of $PM_{2.5}$ concentrations by 26% during July but only 6% during January. A 50% reduction of NH_3 emissions leads to an average 4 and 9% decrease in $PM_{2.5}$ in July and January, respectively.
Schiferl et al. (2014) [31]	In-situ observation and Chemical transport model	2010	Southern California and Central Valley, CA	NH_3	N.A.	The simulations suggested that more than half of the inorganic $PM_{2.5}$ throughout California was produced because of anthropogenic NH_3 emissions.
Lee et al. (2015) [32]	Adjoint Chemical transport model and cost analyses	2005	Global	SO_2	N.A.	The authors found a 10% decrease in SO_2 emissions was the most effective source to control for reducing $PM_{2.5}$-related premature mortality, but regional exceptions were large.
			North America	NH_3	N.A.	For North America, NH_3 had the stronger impacts on $PM_{2.5}$ related premature mortality than SO_2, NO_x, black carbon, and organic matter.
Lelieveld et al. (2015) [33]	Chemical transport model	2010	CONUS	SO_2 and NH_3	Power, transportation, agriculture, industry, residential/ commercial	The study analyzed premature mortality on a global scale. Power generation, agriculture, and land traffic contributed to 31%, 29%, and 21% of premature mortality in the CONUS.
Bauer et al. (2016) [34]	Climate model coupled with aerosol microphysical scheme	2010	CONUS	NH_3	Agriculture	NH_3 contributed significantly to aerosol formation in the eastern US.
		2100	CONUS	NO_x	Combustion sources	Under a future scenario, with doubled emission of agricultural ammonia but lower combustion emission, surface nitrate aerosol concentration will decrease to a level where "agricultural" $PM_{2.5}$ would no longer be the dominating contribution.
Guo et al. (2018) [35]	Thermodynamic analyses based on in-situ observations	2010 summer	Southeastern U.S. (Centreville, VA)	SO_2	N.A.	Control NO_3 was not effective because it only contributed 4% to sulfate-nitrate-ammonium aerosols.
		2015 winter	Northeastern US	NH_3	N.A.	PM response to NO_3^- and SO_4^{2-} reductions were equal in 2015 winter.
		2010 summer	Pasadena, CA	NH_3	N.A.	Effectiveness of NO_3^T reduction followed closely to that of NH_4^T reduction.
Gu et al. (2021) [36]	Chemical transport model and cost analyses	1990-2013	Global	NH_3	Agriculture	Benefit-to-cost ratios of NH_3 and NO_x mitigation (50% reduction) were about 5 and 0.5, respectively, in 2013.
			North America	NH_3	Agriculture	Benefit-to-cost ratios of NH_3 and NO_x mitigation (50% reduction) were about 20 and <1, respectively, in 2013.
Henze et al. (2009) [37]	Adjoint chemical transport model	2001-2002	CONUS	Vary by location and season	Power, industry, transportation, agricultural, commercial/ residential	U.S. SO_2 surface and stack emissions contributed around 30% of inorganic $PM_{2.5}$ concentrations in 2001 - 2002. NH_3 anthropogenic emissions contributed 20% of inorganic $PM_{2.5}$ concentrations in 2001-2002. $PM_{2.5}$ showed the strongest response to anthropogenic NH_3 emissions in the mid-west US.
Fann et al. (2009) [38]	Reduce-formed air quality model	2015	CONUS	Organic matter	Power, transportation, and industry	Reducing mobile NH_3 emissions have a larger benefit ($/ton) in terms of $PM_{2.5}$ reduction than reducing SO_2, NO_x controls.
Holt et al. (2015) [39]	Chemical transport model	2005	CONUS	NH_3	N.A.	NH_3 was most sensitive in the eastern U.S. 2005.
		2012	CONUS	SO_2 and NO_x	N.A.	NH_3 sensitivity decreased because of NO_x and SO_2 reductions.
Heo et al. (2016) [40]	Reduced-form quality model	2005	CONUS	Primary $PM_{2.5}$	Power, transportation,	Social costs were $330 billion, $320 billion, $210 billion, and $160 billion for primary

					agriculture, and industry	$PM_{2.5}$, SO_2, NO_x, and NH_3, respectively, in 2005. Emission-weighted costs were $88000 - 130000/t $PM_{2.5}$, $14000 - 24000/t SO_2, $3800 - 14000/t NO_x, and $23000 - 66000/t NH_3.
Dedoussi et al. (2020) [41]	Chemical transport model analysis	2005	CONUS	Primary $PM_{2.5}$	Power, industry, transportation, and commercial/ residential	Premature deaths attributed to NO_x, SO_2, NH_3, and primary $PM_{2.5}$ were 30000, 21000, 14400, and 34800, respectively, in 2005. These estimates include health impacts of $PM_{2.5}$ and ozone. The authors did not specifically analyze agriculture sector.
		2011	CONUS	Primary $PM_{2.5}$	Power, industry, transportation, and commercial/ residential	Premature deaths attributed to primary NO_x, SO_2, NH_3, and primary $PM_{2.5}$ were 28600, 9900, 16700, and 31000, respectively, in 2011.
		2018	CONUS	Primary $PM_{2.5}$	Power, industry, transportation, and commercial/ residential	Premature deaths attributed to primary NO_x, SO_2, NH_3, and primary $PM_{2.5}$ were 19600, 4300, 17400, and 30200, respectively, in 2018.
Stanier et al. (2012) [42]	Composition analysis	2008 - 2009	LADCO	NO_x	N.A.	Gas ratio was calculated and indicate the region was NO_x limited for NH_4NO_3 formation.
Brewer and Moore (2009) [43]	Composition and chemical transport model analyses	2002 and 2018	WRAP and SESARM	SO_2 and NO_x	Power and transportation	SO_2 emission reductions from electric generation units were most effective way to reduce visibility impairment in the two regions.
Chen et al. (2014) [44]	Chemical transport model analysis	2007, 2019	San Joaquin Valley, CA	Primary $PM_{2.5}$ and NO_x	N.A.	50% reductions of primary $PM_{2.5}$ and NO_x emissions in SJV could lead to 27% and 24% decreases in $PM_{2.5}$. 50% reductions of NH_3 only could lead to 4% decrease in $PM_{2.5}$.
Cheng and Li (2019) [45]	Thermodynamic analysis based on in-situ observations	2002-2004	North Carolina	SO_2 and NO_x	N.A.	North Carolina was determined to be NH_3-rich. $PM_{2.5}$ was insensitive to total NH_4^+.

Supplementary Table 6. Annual and December NH_3 emissions in 2017 in different regions from all sources and agricultural sources. Ammonia emissions are obtained from the EQUATES project[2].

NH_3 Emissions ($ton\ km^{-2}\ yr^{-1}$)	All sources		Agricultural sources	
	Annual	December	Annual	December
Western US	0.36	0.13	0.25	0.10
Central US	0.81	0.31	0.64	0.23
Midwestern US	0.73	0.25	0.55	0.20
Northeastern US	0.35	0.18	0.24	0.08
Southeastern US	0.63	0.34	0.44	0.18

Supplementary Table 7. Potential biases, precisions, and detection limits of observations from different monitoring networks.

Species	Network	Temporal resolution	Bias	Precision	Detection limit	Note
NH_4^+	CASTNET	Weekly	0	3%	0.5 µg filter^{-1}	Detection limits for concentration measurements (µg m^{-3}) are calculated as filter detection limits (µg filter^{-1}) divided by flow volume.<br><br>Precision of Na^+, K^+, Mg^{2+}, Ca^{2+} are increased to 100% to capture the uncertainties introduced by scaling raw CASTNET observations.
Na^+	CASTNET	Weekly	0	100%	0.125 µg filter^{-1}	
K^+	CASTNET	Weekly	0	100%	0.15 µg filter^{-1}	
Mg^{2+}	CASTNET	Weekly	0	100%	0.075 µg filter^{-1}	
Ca^{2+}	CASTNET	Weekly	0	100%	0.15 µg filter^{-1}	
SO_4^{2-}	CASTNET	Weekly	0	3%	1.0 µg filter^{-1}	
NO_3^-	CASTNET	Weekly	-10%	5%	0.2 µg filter^{-1}	
Cl^-	CASTNET	Weekly	0	50%	0.5 µg filter^{-1}	
HNO_3	CASTNET	Weekly	10%	5%	0.2 µg filter^{-1}	
NH_3	AMoN	Biweekly	-10%	5%	<6/2018: 0.047 mg L^{-1};<br>2018/6-12: 0.119 mg L^{-1};<br>2019: 0.104 mg L^{-1};<br>2020: 0.083 mg L^{-1}.	To derive the detection limit of NH_3 concentration (µg m^{-3}), the solution detection limit is scaled by the ratio of the reported gas concentration (µg m^{-3}) to the reported solution concentration (mg L^{-1}).
T	CASTNET	Hourly	0	1 °C	NA	ISD and NARR precisions are determined based on the intercomparison of meteorological observations.
	ISD	Hourly	0	2 °C	NA	
	NARR	Every three hours	0	4 °C	NA	
RH	CASTNET	Hourly	0	5%	NA	
	ISD	Hourly	0	8%	NA	
	NARR	Every three hours	0	18%	NA	

Supplementary Table 8. Intercomparison of data from CASTNET, IMPROVE, CSN, ISD, and NARR at different spatial windows. Hourly meteorological observations from CASTNET and ISD are averaged down to three-hour to match the time resolution of NARR. For chemical compounds, observations are averaged weekly to match the temporal resolution of CASTNET. m and b are the slopes and intercepts of orthogonal distance regressions (ODRs), and δ_m and δ_b are their uncertainties (95% CI defined as ±1.96SD), respectively.

Network 1 (x)	Network 2 (y)	Variable	Distance	m	δ_m	b	δ_b	R^2	NMB	RMSE	Sample #
CASTNET	ISD	T	<10	0.994	0.001	1.53	0.19	0.98	0.1%	1.36	40187
CASTNET	ISD	T	10-30	1.018	0.000	-4.94	0.07	0.98	-0.1%	1.64	300406
CASTNET	ISD	T	30-50	1.022	0.001	-6.58	0.18	0.98	0.1%	1.65	50607
CASTNET	ISD	T	50-100	1.039	0.004	-11.66	1.20	0.76	0.1%	4.83	17198
CASTNET	ISD	RH	<10	0.962	0.002	3.12	0.14	0.86	-0.6%	8.07	40187
CASTNET	ISD	RH	10-30	0.956	0.001	2.47	0.06	0.80	0.9%	9.34	300406
CASTNET	ISD	RH	30-50	0.940	0.002	4.18	0.14	0.83	0.3%	8.17	50607
CASTNET	ISD	RH	50-100	1.161	0.011	-11.13	0.80	0.19	-0.3%	22.43	17198
CASTNET	NARR	T	<10	1.037	0.001	-10.60	0.30	0.76	0.0%	5.58	266095
CASTNET	NARR	T	10-30	1.065	0.001	-18.70	0.17	0.74	0.1%	6.03	1032506
CASTNET	NARR	RH	<10	0.880	0.002	10.75	0.15	0.21	-3.3%	21.62	266095
CASTNET	NARR	RH	10-30	0.935	0.001	7.04	0.06	0.34	-4.3%	21.03	1032506
ISD	NARR	T	<10	1.007	0.000	-1.85	0.11	0.94	-0.1%	3.02	457431
ISD	NARR	T	10-30	1.001	0.000	0.11	0.07	0.93	-0.2%	3.08	1067370
ISD	NARR	RH	<10	0.831	0.001	16.06	0.08	0.46	-5.3%	14.99	457431
ISD	NARR	RH	10-30	0.825	0.001	16.12	0.05	0.45	-4.7%	15.03	1067370
IMPROVE	CASTNET	NH_4^+	<10	0.96	0.01	-0.06	0.00	0.85	16.9%	0.19	3659
IMPROVE	CASTNET	NH_4^+	10-30	0.85	0.03	-0.01	0.03	0.78	19.5%	0.28	172
IMPROVE	CASTNET	NH_4^+	30-50	0.96	0.02	-0.05	0.01	0.81	14.5%	0.16	548
IMPROVE	CASTNET	NH_4^+	50-100	0.82	0.02	-0.07	0.01	0.74	47.2%	0.21	473
IMPROVE	CASTNET	NO_3^-	<10	1.12	0.01	0.10	0.01	0.84	-23.0%	0.40	3659
IMPROVE	CASTNET	NO_3^-	10-30	0.87	0.04	-0.01	0.04	0.74	16.4%	0.44	172
IMPROVE	CASTNET	NO_3^-	30-50	1.10	0.03	-0.07	0.02	0.68	6.6%	0.26	548
IMPROVE	CASTNET	NO_3^-	50-100	1.25	0.04	-0.05	0.02	0.65	-9.9%	0.29	473
IMPROVE	CASTNET	SO_4^{2-}	<10	1.09	0.01	0.07	0.01	0.92	-13.3%	0.31	3659
IMPROVE	CASTNET	SO_4^{2-}	10-30	1.18	0.04	-0.08	0.07	0.84	-11.5%	0.53	172
IMPROVE	CASTNET	SO_4^{2-}	30-50	1.11	0.02	-0.02	0.03	0.87	-8.0%	0.34	548
IMPROVE	CASTNET	SO_4^{2-}	50-100	0.86	0.02	0.12	0.02	0.84	2.4%	0.25	473
CSN	CASTNET	NH_4^+	<10	1.09	0.06	0.16	0.02	0.55	-36.2%	0.27	208
CSN	CASTNET	NH_4^+	30-50	1.03	0.03	0.10	0.02	0.67	-21.6%	0.23	510
CSN	CASTNET	NH_4^+	50-100	1.08	0.02	0.12	0.01	0.65	-30.0%	0.23	1678
CSN	CASTNET	NO_3^-	<10	1.09	0.05	0.09	0.05	0.67	-16.6%	0.53	208
CSN	CASTNET	NO_3^-	30-50	0.97	0.02	-0.03	0.03	0.80	6.1%	0.49	510
CSN	CASTNET	NO_3^-	50-100	1.05	0.01	0.04	0.02	0.76	-9.6%	0.48	1678
CSN	CASTNET	SO_4^{2-}	<10	0.74	0.05	0.38	0.05	0.44	-11.1%	0.30	208
CSN	CASTNET	SO_4^{2-}	30-50	0.87	0.03	0.21	0.03	0.64	-6.0%	0.23	510
CSN	CASTNET	SO_4^{2-}	50-100	0.88	0.02	0.19	0.02	0.50	-6.2%	0.32	1678
CSN	IMPROVE	NH_4^+	<10	0.84	0.06	0.27	0.05	0.70	-21.7%	0.36	72
CSN	IMPROVE	NH_4^+	10-30	1.08	0.07	0.37	0.05	0.62	-39.4%	0.48	126
CSN	IMPROVE	NH_4^+	30-50	1.60	0.30	0.25	0.06	0.42	-67.5%	0.38	26
CSN	IMPROVE	NH_4^+	50-100	0.74	0.03	0.40	0.01	0.58	-50.4%	0.38	291
CSN	IMPROVE	NO_3^-	<10	0.74	0.05	0.09	0.05	0.73	15.5%	0.44	72
CSN	IMPROVE	NO_3^-	10-30	1.11	0.04	0.00	0.05	0.83	-9.7%	0.37	126
CSN	IMPROVE	NO_3^-	30-50	0.97	0.20	0.03	0.06	0.36	-7.4%	0.10	26
CSN	IMPROVE	NO_3^-	50-100	0.82	0.02	0.05	0.02	0.78	10.3%	0.30	291
CSN	IMPROVE	SO_4^{2-}	<10	0.92	0.06	0.03	0.12	0.77	6.6%	0.59	72
CSN	IMPROVE	SO_4^{2-}	10-30	1.05	0.05	0.01	0.11	0.73	-5.3%	0.41	126
CSN	IMPROVE	SO_4^{2-}	30-50	1.03	0.15	0.02	0.17	0.62	-4.2%	0.27	26
CSN	IMPROVE	SO_4^{2-}	50-100	0.73	0.04	0.30	0.05	0.47	4.9%	0.48	291

Supplementary Table 9. Observed and simulated annual $\boldsymbol{\varepsilon}_{\mathbf{NH_4^+}}$. "W US", "C US", "MW US", "NE US", "SE US" stand for the Western, Central, Midwestern, Northeastern, Southeastern US, respectively. The 25th and 75th percentiles of annual $\varepsilon_{NH_4^+}$ from the sites within the corresponding region show its regional variability. Uncertainty lower and upper bounds are the mean uncertainties (95% CI defined as the 2.5^{th} and the 97.5^{th} percentiles) of the sites within the region. Uncertainties of each site are calculated from 1000 Monte Carlo simulations based on the precisions and detection limits shown in Supplementary Table 7.

		**Observed annual $\varepsilon_{NH_4^+}$**					**Simulated annual $\varepsilon_{NH_4^+}$**				
Region	Year	Median	25th percentile	75th percentile	Uncertainty lower bound	Uncertainty upper bound	Median	25th percentile	75th percentile	Uncertainty lower bound	Uncertainty upper bound
W US (WRAP)	2011	0.34	0.39	0.30	-0.03	0.03	0.29	0.26	0.34	-0.03	0.04
	2012	0.26	0.33	0.28	-0.03	0.03	0.25	0.23	0.26	-0.02	0.03
	2013	0.20	0.26	0.25	-0.03	0.03	0.19	0.18	0.31	-0.02	0.03
	2014	0.15	0.25	0.25	-0.03	0.03	0.18	0.13	0.25	-0.03	0.03
	2015	0.17	0.28	0.27	-0.04	0.03	0.23	0.15	0.30	-0.03	0.04
	2016	0.18	0.26	0.29	-0.04	0.03	0.19	0.16	0.27	-0.03	0.04
	2017	0.19	0.26	0.33	-0.03	0.03	0.20	0.15	0.26	-0.03	0.05
	2018	0.15	0.25	0.38	-0.04	0.04	0.16	0.11	0.25	-0.04	0.06
	2019	0.18	0.30	0.42	-0.03	0.03	0.25	0.15	0.32	-0.04	0.06
	2020	0.18	0.24	0.56	-0.14	0.10	0.19	0.13	0.23	-0.06	0.16
C US (CENSARA)	2011	0.48	0.61	0.30	-0.01	0.01	0.57	0.51	0.65	-0.04	0.04
	2012	0.38	0.51	0.28	-0.01	0.01	0.47	0.41	0.55	-0.03	0.03
	2013	0.34	0.44	0.25	-0.01	0.01	0.43	0.38	0.50	-0.03	0.03
	2014	0.23	0.36	0.25	-0.01	0.01	0.33	0.27	0.42	-0.03	0.03
	2015	0.20	0.42	0.27	-0.01	0.01	0.32	0.24	0.51	-0.03	0.03
	2016	0.18	0.39	0.29	-0.01	0.01	0.28	0.21	0.47	-0.03	0.03
	2017	0.15	0.36	0.33	-0.01	0.01	0.27	0.19	0.43	-0.03	0.04
	2018	0.15	0.37	0.38	-0.01	0.01	0.24	0.19	0.42	-0.04	0.05
	2019	0.15	0.43	0.42	-0.01	0.01	0.28	0.18	0.48	-0.04	0.05
	2020	0.14	0.37	0.56	-0.01	0.01	0.23	0.16	0.34	-0.04	0.07
MW US (LADCO)	2011	0.50	0.68	0.30	-0.01	0.01	0.59	0.50	0.72	-0.03	0.03
	2012	0.45	0.50	0.28	-0.01	0.01	0.51	0.49	0.55	-0.02	0.02
	2013	0.42	0.50	0.25	-0.01	0.01	0.49	0.47	0.55	-0.02	0.02
	2014	0.39	0.48	0.25	-0.01	0.01	0.45	0.44	0.53	-0.03	0.03
	2015	0.33	0.48	0.27	-0.01	0.01	0.42	0.38	0.54	-0.02	0.02
	2016	0.30	0.41	0.29	-0.01	0.01	0.38	0.36	0.47	-0.02	0.02
	2017	0.26	0.39	0.33	-0.01	0.01	0.37	0.30	0.43	-0.03	0.03
	2018	0.31	0.43	0.38	-0.01	0.01	0.39	0.33	0.47	-0.03	0.04
	2019	0.31	0.42	0.42	-0.01	0.01	0.38	0.33	0.46	-0.03	0.04
	2020	0.27	0.37	0.56	-0.01	0.01	0.33	0.30	0.41	-0.06	0.07
NE US (MANE-VU)	2011	0.68	0.78	0.30	-0.02	0.02	0.79	0.75	0.84	-0.04	0.04
	2012	0.59	0.70	0.28	-0.02	0.02	0.72	0.61	0.82	-0.04	0.04
	2013	0.52	0.58	0.25	-0.02	0.02	0.61	0.59	0.66	-0.03	0.03
	2014	0.49	0.60	0.25	-0.02	0.02	0.59	0.55	0.67	-0.03	0.03
	2015	0.40	0.58	0.27	-0.02	0.02	0.59	0.45	0.64	-0.03	0.04
	2016	0.35	0.51	0.29	-0.02	0.02	0.50	0.40	0.58	-0.03	0.04
	2017	0.35	0.48	0.33	-0.02	0.02	0.48	0.35	0.55	-0.05	0.06
	2018	0.38	0.52	0.38	-0.03	0.02	0.51	0.41	0.61	-0.06	0.08
	2019	0.39	0.51	0.42	-0.02	0.02	0.52	0.41	0.57	-0.06	0.07
	2020	0.31	0.50	0.56	-0.02	0.02	0.47	0.36	0.52	-0.13	0.11
SE US (SESARM)	2011	0.47	0.73	0.30	-0.01	0.01	0.68	0.47	0.78	-0.04	0.04
	2012	0.45	0.57	0.28	-0.02	0.02	0.58	0.39	0.68	-0.03	0.03
	2013	0.35	0.51	0.25	-0.02	0.02	0.54	0.30	0.58	-0.03	0.03
	2014	0.34	0.48	0.25	-0.02	0.02	0.52	0.32	0.58	-0.03	0.04
	2015	0.33	0.48	0.27	-0.02	0.02	0.47	0.36	0.56	-0.03	0.03
	2016	0.30	0.46	0.29	-0.02	0.02	0.42	0.32	0.47	-0.03	0.03
	2017	0.27	0.43	0.33	-0.02	0.02	0.41	0.26	0.44	-0.04	0.05
	2018	0.29	0.46	0.38	-0.02	0.02	0.43	0.28	0.50	-0.05	0.06
	2019	0.28	0.42	0.42	-0.02	0.02	0.40	0.26	0.46	-0.05	0.06
	2020	0.26	0.42	0.56	-0.02	0.02	0.36	0.27	0.43	-0.09	0.11

Supplementary Table 10. Observed and simulated annual $\varepsilon_{NO_3^-}$. "W US", "C US", "MW US", "NE US", "SE US" stand for the Western, Central, Midwestern, Northeastern, Southeastern US, respectively. The 25th and 75th percentiles of annual $\varepsilon_{NO_3^-}$ from the sites within the corresponding region show its regional variability. Uncertainty lower and upper bounds are the mean uncertainties (95% CI, defined as the 2.5th and the 97.5th percentiles) of the sites within the region. Uncertainties of each site are calculated from 1000 Monte Carlo simulations based on the precisions and detection limits shown in Supplementary Table 7.

		**Observed annual $\varepsilon_{NO_3^-}$**					**Simulated annual $\varepsilon_{NO_3^-}$**				
**Region**	**Year**	**Median**	**25th percentile**	**75th percentile**	**Uncertainty lower bound**	**Uncertainty upper bound**	**Median**	**25th percentile**	**75th percentile**	**Uncertainty lower bound**	**Uncertainty upper bound**
W US (WRAP)	2011	0.40	0.33	0.42	-0.03	0.04	0.19	0.12	0.20	-0.04	0.05
	2012	0.37	0.34	0.39	-0.02	0.03	0.22	0.16	0.32	-0.04	0.04
	2013	0.37	0.36	0.42	-0.02	0.03	0.34	0.19	0.43	-0.05	0.07
	2014	0.37	0.35	0.41	-0.03	0.03	0.25	0.13	0.38	-0.04	0.05
	2015	0.37	0.34	0.40	-0.03	0.04	0.30	0.21	0.33	-0.06	0.09
	2016	0.37	0.35	0.41	-0.03	0.03	0.30	0.17	0.33	-0.07	0.10
	2017	0.35	0.33	0.42	-0.04	0.06	0.32	0.20	0.36	-0.14	0.14
	2018	0.39	0.38	0.45	-0.05	0.09	0.31	0.21	0.33	-0.07	0.08
	2019	0.36	0.34	0.39	-0.05	0.08	0.32	0.20	0.38	-0.15	0.11
	2020	0.38	0.38	0.40	-0.08	0.31	0.33	0.19	0.36	-0.16	0.20
C US (CENSARA)	2011	0.50	0.41	0.53	-0.02	0.02	0.32	0.29	0.38	-0.02	0.02
	2012	0.49	0.42	0.53	-0.02	0.02	0.35	0.29	0.36	-0.02	0.02
	2013	0.51	0.42	0.58	-0.01	0.01	0.46	0.41	0.49	-0.02	0.02
	2014	0.61	0.50	0.69	-0.02	0.02	0.51	0.50	0.52	-0.02	0.04
	2015	0.56	0.46	0.67	-0.02	0.02	0.47	0.42	0.52	-0.02	0.03
	2016	0.57	0.49	0.67	-0.01	0.02	0.43	0.40	0.49	-0.02	0.03
	2017	0.58	0.44	0.69	-0.03	0.04	0.42	0.38	0.52	-0.03	0.03
	2018	0.61	0.50	0.66	-0.04	0.05	0.45	0.38	0.57	-0.05	0.04
	2019	0.60	0.52	0.69	-0.04	0.05	0.50	0.44	0.56	-0.05	0.04
	2020	0.60	0.54	0.68	-0.05	0.09	0.45	0.35	0.54	-0.04	0.05
MW US (LADCO)	2011	0.52	0.49	0.55	-0.02	0.02	0.49	0.44	0.53	-0.02	0.02
	2012	0.57	0.48	0.58	-0.02	0.02	0.53	0.51	0.55	-0.01	0.01
	2013	0.57	0.52	0.62	-0.01	0.01	0.67	0.62	0.68	-0.01	0.01
	2014	0.57	0.51	0.63	-0.02	0.02	0.67	0.63	0.68	-0.02	0.02
	2015	0.50	0.48	0.56	-0.02	0.02	0.57	0.54	0.63	-0.02	0.02
	2016	0.53	0.50	0.56	-0.02	0.02	0.56	0.53	0.64	-0.03	0.03
	2017	0.55	0.49	0.57	-0.02	0.03	0.57	0.54	0.64	-0.03	0.02
	2018	0.59	0.53	0.61	-0.03	0.04	0.60	0.58	0.67	-0.03	0.03
	2019	0.60	0.52	0.63	-0.03	0.04	0.59	0.56	0.65	-0.04	0.04
	2020	0.57	0.50	0.58	-0.06	0.08	0.58	0.53	0.60	-0.03	0.01
NE US (MANE-VU)	2011	0.37	0.30	0.38	-0.04	0.05	0.43	0.36	0.43	-0.04	0.03
	2012	0.41	0.34	0.45	-0.03	0.04	0.43	0.39	0.50	-0.04	0.03
	2013	0.40	0.38	0.44	-0.03	0.03	0.51	0.46	0.53	-0.04	0.03
	2014	0.42	0.41	0.46	-0.03	0.04	0.52	0.49	0.57	-0.04	0.03
	2015	0.41	0.38	0.45	-0.03	0.04	0.47	0.41	0.50	-0.04	0.03
	2016	0.42	0.39	0.45	-0.03	0.04	0.43	0.42	0.49	-0.03	0.03
	2017	0.44	0.42	0.47	-0.06	0.08	0.50	0.45	0.57	-0.05	0.05
	2018	0.48	0.44	0.51	-0.08	0.11	0.53	0.44	0.57	-0.07	0.05
	2019	0.48	0.45	0.51	-0.07	0.11	0.47	0.44	0.50	-0.07	0.06
	2020	0.44	0.42	0.48	-0.14	0.22	0.44	0.39	0.49	-0.16	0.08
SE US (SESARM)	2011	0.31	0.27	0.43	-0.03	0.03	0.29	0.22	0.43	-0.05	0.04
	2012	0.39	0.31	0.50	-0.02	0.02	0.36	0.29	0.47	-0.04	0.04
	2013	0.41	0.33	0.52	-0.02	0.02	0.46	0.38	0.60	-0.04	0.03
	2014	0.41	0.32	0.52	-0.03	0.03	0.43	0.37	0.60	-0.04	0.03
	2015	0.39	0.34	0.43	-0.02	0.03	0.41	0.33	0.56	-0.04	0.04
	2016	0.38	0.35	0.43	-0.02	0.03	0.37	0.32	0.52	-0.05	0.04
	2017	0.40	0.37	0.46	-0.04	0.06	0.38	0.33	0.54	-0.05	0.05
	2018	0.41	0.38	0.48	-0.06	0.08	0.43	0.36	0.55	-0.06	0.05
	2019	0.39	0.35	0.45	-0.06	0.09	0.35	0.32	0.53	-0.07	0.06
	2020	0.41	0.36	0.46	-0.12	0.20	0.38	0.31	0.53	-0.12	0.06

Supplementary Table 11. Simulated annual pH and aerosol water content (AWC). “W US”, “C US”, “MW US”, “NE US”, “SE US” stand for the Western, Central, Midwestern, Northeastern, Southeastern US, respectively. The 25th and 75th percentiles of annual pH and AWC from the sites within the corresponding RPO show its regional variability. Uncertainty lower and upper bounds are the mean uncertainties (95% CI defined as the 2.5^{th} and the 97.5^{th} percentiles) of the sites within the RPO. Uncertainties of each site are calculated from 1000 Monte Carlo simulations based on the precisions and detection limits shown in Supplementary Table 7.

		pH					AWC				
**Region**	**Year**	**Median**	**25th percentile**	**75th percentile**	**Uncertainty lower bound**	**Uncertainty upper bound**	**Median**	**25th percentile**	**75th percentile**	**Uncertainty lower bound**	**Uncertainty upper bound**
W US (WRAP)	2011	1.9	1.9	2.3	-0.2	0.2	1.2	0.9	4.3	-0.2	0.5
	2012	2.1	2.0	2.4	-0.1	0.2	0.8	0.7	1.2	-0.4	0.8
	2013	2.4	2.2	2.5	-0.1	0.2	1.0	0.9	1.8	-0.3	0.6
	2014	2.4	2.3	2.5	-0.1	0.1	0.9	0.8	1.2	-0.3	0.6
	2015	2.2	2.1	2.4	-0.2	0.2	1.2	1.0	2.1	-0.3	0.6
	2016	2.4	2.3	2.6	-0.2	0.2	1.2	0.7	1.9	-0.3	0.6
	2017	2.5	2.4	2.6	-0.2	0.2	1.2	0.6	2.5	-0.4	0.6
	2018	2.7	2.6	2.8	-0.1	0.1	1.2	0.7	1.6	-0.4	0.7
	2019	2.4	2.3	2.6	-0.2	0.2	1.0	0.7	1.6	-0.4	0.6
	2020	2.7	2.4	3.0	-0.2	0.2	0.7	0.5	1.7	-0.2	0.4
C US (CENSARA)	2011	2.0	1.9	2.1	-0.1	0.1	42.4	37.3	45.1	-2.0	5.7
	2012	2.0	1.8	2.1	-0.1	0.1	8.8	6.7	9.0	-5.0	9.2
	2013	2.3	2.2	2.5	-0.1	0.2	16.7	12.1	23.8	-5.5	7.6
	2014	2.7	2.5	2.8	-0.1	0.2	19.1	6.5	41.8	-3.9	6.1
	2015	2.5	2.3	2.8	-0.1	0.2	6.9	5.2	18.9	-3.8	5.9
	2016	2.5	2.3	2.8	-0.2	0.2	7.9	5.3	18.1	-3.4	5.9
	2017	2.5	2.2	2.9	-0.2	0.2	8.2	5.0	18.1	-2.7	5.3
	2018	2.7	2.2	3.2	-0.2	0.2	8.1	5.9	15.4	-3.7	5.8
	2019	2.7	2.2	3.1	-0.3	0.3	7.4	4.4	21.5	-3.0	5.2
	2020	2.7	2.1	3.2	-0.2	0.2	5.8	5.6	10.9	-0.4	5.0
MW US (LADCO)	2011	2.1	2.1	2.3	-0.1	0.1	15.4	11.4	26.7	-3.8	8.2
	2012	2.3	2.3	2.5	-0.1	0.1	11.3	8.3	18.4	-6.1	10.1
	2013	2.8	2.5	2.8	-0.1	0.1	18.8	10.2	40.4	-4.8	7.6
	2014	2.8	2.5	3.0	-0.1	0.1	20.1	11.3	28.9	-4.6	7.9
	2015	2.7	2.4	2.9	-0.1	0.1	12.8	8.7	22.3	-4.2	7.0
	2016	2.7	2.4	2.9	-0.1	0.2	12.3	7.1	22.1	-5.0	7.7
	2017	2.8	2.6	3.0	-0.2	0.1	16.1	8.3	28.3	-4.9	7.7
	2018	2.9	2.7	3.1	-0.2	0.2	18.0	8.7	27.3	-3.3	6.0
	2019	2.8	2.5	3.0	-0.2	0.2	10.7	9.0	15.1	-3.6	6.3
	2020	2.8	2.6	2.9	-0.2	0.1	12.1	5.8	20.6	-1.0	5.2
NE US (MANE-VU)	2011	1.7	1.5	1.8	-0.1	0.1	75.9	29.3	77.2	-4.6	6.1
	2012	2.0	1.8	2.1	-0.1	0.1	8.2	7.0	14.3	-2.6	5.0
	2013	2.1	2.1	2.2	-0.1	0.1	8.4	6.2	11.2	-2.2	4.5
	2014	2.1	2.0	2.3	-0.1	0.1	7.0	5.1	10.3	-2.9	4.9
	2015	2.1	1.8	2.2	-0.1	0.1	5.8	4.4	14.6	-2.8	4.6
	2016	2.1	1.9	2.3	-0.1	0.1	7.2	3.6	14.1	-3.7	6.5
	2017	2.4	2.2	2.5	-0.2	0.2	12.4	7.6	20.6	-4.3	6.6
	2018	2.4	2.2	2.4	-0.2	0.2	15.6	6.5	21.4	-3.1	5.2
	2019	2.2	2.1	2.4	-0.3	0.2	8.1	4.5	12.0	-3.1	4.9
	2020	2.2	2.0	2.5	-0.5	0.4	5.8	3.2	11.0	-4.3	10.5
SE US (SESARM)	2011	1.7	1.5	2.2	-0.1	0.2	31.7	20.3	53.4	-5.4	8.8
	2012	1.9	1.8	2.4	-0.2	0.2	15.6	9.7	30.8	-6.0	10.1
	2013	2.3	1.9	2.7	-0.1	0.2	13.4	8.6	21.8	-5.6	8.9
	2014	2.3	1.9	2.6	-0.2	0.2	16.1	11.5	20.3	-4.9	7.5
	2015	2.1	1.9	2.5	-0.2	0.2	16.0	9.3	30.9	-3.9	6.2
	2016	2.1	1.9	2.6	-0.2	0.2	11.6	6.4	25.2	-4.2	6.6
	2017	2.2	2.0	2.6	-0.2	0.2	16.3	6.8	29.1	-4.8	6.9
	2018	2.2	2.0	2.7	-0.3	0.2	14.3	8.2	25.3	-4.1	6.2
	2019	2.1	2.0	2.6	-0.3	0.3	10.3	6.9	20.8	-4.5	6.6
	2020	2.2	2.0	2.7	-0.3	0.3	12.2	7.9	20.9	-4.6	8.9

Supplementary Table 12. Simulated annual $\Delta c_{\mathrm{SIA}}/\Delta c_{\mathrm{SO_4^{2-}}}$, $\Delta c_{\mathrm{SIA}}/\Delta c_{\mathrm{NO_3^T}}$, and $\Delta c_{\mathrm{SIA}}/\Delta c_{\mathrm{NH_4^T}}$ with 10% reductions. "W US", "C US", "MW US", "NE US", "SE US" stand for the Western, Central, Midwestern, Northeastern, Southeastern US, respectively. The 25th and 75th percentiles (perc) of values from the sites within the corresponding region show their regional variability. Uncertainty lower and upper bounds (Unc LB and Unc UB) are the mean uncertainties (95% CI defined as the 2.5th and the 97.5th percentiles) of the sites within the region. Uncertainties of each site are estimated using 500 Monte Carlo simulations based on the precisions and detection limits shown in Supplementary Table 7.

		$\Delta c_{\mathrm{SIA}}/\Delta c_{\mathrm{SO_4^{2-}}}$ (10%)					$\Delta c_{\mathrm{SIA}}/\Delta c_{\mathrm{NO_3^T}}$ (10%)					$\Delta c_{\mathrm{SIA}}/\Delta c_{\mathrm{NH_4^T}}$ (10%)				
**Region**	**Year**	**Median**	**25th perc**	**75th perc**	**Unc LB**	**Unc UB**	**Median**	**25th perc**	**75th perc**	**Unc LB**	**Unc UB**	**Median**	**25th perc**	**75th perc**	**Unc LB**	**Unc UB**
W US (WRAP)	2011	1.33	1.09	1.37	-0.04	0.31	0.23	0.19	0.31	-0.01	0.03	0.16	0.12	0.19	0.00	0.01
	2012	1.39	1.38	1.42	-0.09	0.28	0.27	0.17	0.40	-0.01	0.02	0.13	0.10	0.16	0.00	0.01
	2013	1.37	1.37	1.39	-0.14	0.34	0.45	0.25	0.54	-0.01	0.07	0.13	0.09	0.14	0.00	0.01
	2014	1.37	1.11	1.39	-0.14	0.37	0.33	0.18	0.51	-0.01	0.07	0.12	0.07	0.14	0.00	0.01
	2015	1.37	1.34	1.38	-0.16	0.35	0.41	0.25	0.44	-0.01	0.03	0.12	0.09	0.15	0.00	0.01
	2016	1.35	1.27	1.37	-0.31	0.64	0.41	0.25	0.42	-0.02	0.05	0.10	0.08	0.14	0.00	0.01
	2017	1.39	1.31	1.40	-0.36	0.72	0.41	0.23	0.45	-0.03	0.05	0.09	0.07	0.12	0.00	0.01
	2018	1.34	0.87	1.39	-0.54	1.08	0.39	0.34	0.45	-0.05	0.07	0.10	0.07	0.14	0.00	0.01
	2019	1.37	1.35	1.38	-0.09	0.38	0.45	0.29	0.51	-0.02	0.03	0.13	0.08	0.17	0.00	0.03
	2020	1.33	1.08	1.35	-0.49	0.85	0.46	0.41	0.51	-0.05	0.07	0.10	0.07	0.12	0.00	0.01
C US (CENSARA)	2011	1.29	1.27	1.30	0.00	0.00	0.54	0.45	0.63	0.00	0.01	0.37	0.33	0.42	-0.01	0.02
	2012	1.36	1.32	1.37	0.00	0.08	0.59	0.46	0.60	0.00	0.01	0.21	0.19	0.30	0.00	0.01
	2013	1.35	1.33	1.36	0.00	0.01	0.72	0.61	0.79	0.00	0.01	0.22	0.18	0.26	0.00	0.01
	2014	1.37	1.35	1.39	-0.01	0.12	0.79	0.75	0.81	0.00	0.01	0.17	0.13	0.21	0.00	0.01
	2015	1.37	1.32	1.40	-0.02	0.26	0.68	0.64	0.77	-0.01	0.02	0.16	0.14	0.24	0.00	0.02
	2016	1.39	1.32	1.40	-0.02	0.15	0.65	0.59	0.77	-0.01	0.01	0.15	0.10	0.24	0.00	0.01
	2017	1.39	1.32	1.41	-0.01	0.12	0.64	0.52	0.83	-0.01	0.01	0.14	0.10	0.23	0.00	0.01
	2018	1.38	1.32	1.40	-0.03	0.13	0.71	0.54	0.86	0.00	0.01	0.11	0.09	0.26	0.00	0.02
	2019	1.37	1.31	1.41	-0.04	0.24	0.77	0.62	0.83	-0.01	0.02	0.14	0.09	0.30	0.00	0.03
	2020	1.39	1.28	1.42	-0.10	0.20	0.70	0.48	0.84	-0.01	0.02	0.15	0.09	0.27	0.00	0.03
MW US (LADCO)	2011	1.22	1.12	1.32	-0.01	0.03	0.58	0.50	0.70	-0.01	0.01	0.60	0.33	0.92	-0.01	0.02
	2012	1.36	1.32	1.37	0.00	0.03	0.81	0.75	0.83	0.00	0.01	0.25	0.19	0.42	0.00	0.02
	2013	1.35	1.31	1.36	0.00	0.01	0.95	0.86	0.98	0.00	0.01	0.22	0.17	0.47	0.00	0.02
	2014	1.36	1.27	1.37	0.00	0.02	0.98	0.82	1.02	0.00	0.03	0.21	0.12	0.56	-0.01	0.03
	2015	1.30	1.22	1.37	-0.02	0.08	0.85	0.69	0.95	-0.01	0.02	0.30	0.20	0.78	0.00	0.05
	2016	1.34	1.29	1.38	-0.01	0.06	0.85	0.72	0.94	-0.01	0.01	0.21	0.16	0.49	-0.01	0.03
	2017	1.37	1.33	1.38	-0.01	0.14	0.87	0.82	0.95	0.00	0.01	0.19	0.13	0.34	0.00	0.02
	2018	1.35	1.32	1.38	-0.01	0.04	0.93	0.88	1.00	0.00	0.01	0.18	0.11	0.39	0.00	0.04
	2019	1.36	1.31	1.38	-0.02	0.09	0.93	0.84	1.00	-0.01	0.02	0.21	0.12	0.46	-0.01	0.06
	2020	1.34	1.31	1.38	-0.08	0.21	0.86	0.79	0.92	-0.01	0.03	0.19	0.16	0.45	-0.01	0.05
NE US (MANE-VU)	2011	1.07	0.96	1.14	-0.01	0.02	0.39	0.31	0.51	-0.01	0.02	1.14	0.95	1.45	-0.03	0.06
	2012	1.14	1.10	1.22	-0.01	0.01	0.60	0.46	0.64	-0.01	0.02	0.95	0.58	1.08	-0.02	0.04
	2013	1.23	1.14	1.25	-0.01	0.02	0.64	0.54	0.75	-0.02	0.03	0.64	0.49	0.99	-0.03	0.05
	2014	1.16	1.14	1.22	-0.02	0.03	0.67	0.57	0.72	-0.02	0.03	0.71	0.60	0.90	-0.05	0.06
	2015	1.08	1.01	1.17	-0.02	0.04	0.49	0.46	0.61	-0.02	0.03	0.86	0.62	1.08	-0.03	0.05
	2016	1.23	1.18	1.26	-0.02	0.03	0.62	0.48	0.66	-0.02	0.02	0.53	0.35	0.74	-0.02	0.04
	2017	1.25	1.24	1.29	-0.01	0.03	0.69	0.60	0.80	-0.01	0.02	0.45	0.30	0.67	-0.02	0.04
	2018	1.22	1.17	1.29	-0.03	0.05	0.75	0.59	0.83	-0.02	0.04	0.56	0.34	0.83	-0.02	0.07
	2019	1.20	1.14	1.27	-0.04	0.06	0.67	0.58	0.81	-0.03	0.05	0.53	0.33	0.96	-0.03	0.08
	2020	1.22	1.17	1.28	-0.03	0.06	0.62	0.55	0.69	-0.02	0.04	0.53	0.30	0.74	-0.03	0.07
SE US (SESARM)	2011	1.22	0.94	1.28	0.00	0.13	0.46	0.35	0.66	-0.01	0.03	0.33	0.16	0.53	-0.01	0.10
	2012	1.25	1.09	1.30	-0.01	0.02	0.55	0.43	0.69	-0.01	0.01	0.34	0.21	0.49	-0.01	0.02
	2013	1.24	1.18	1.30	0.00	0.02	0.68	0.54	0.77	-0.01	0.01	0.36	0.17	0.51	-0.01	0.02
	2014	1.23	1.14	1.30	-0.01	0.02	0.64	0.55	0.70	-0.01	0.02	0.29	0.20	0.53	-0.01	0.02
	2015	1.22	1.16	1.28	-0.02	0.07	0.56	0.45	0.66	-0.01	0.02	0.36	0.21	0.54	-0.01	0.03
	2016	1.29	1.23	1.33	-0.01	0.11	0.52	0.44	0.68	-0.01	0.02	0.30	0.18	0.37	-0.01	0.02
	2017	1.30	1.24	1.32	-0.01	0.07	0.56	0.45	0.67	-0.01	0.02	0.25	0.17	0.35	0.00	0.02
	2018	1.27	1.22	1.32	-0.01	0.07	0.60	0.49	0.71	-0.01	0.03	0.31	0.19	0.46	-0.01	0.03
	2019	1.30	1.21	1.33	-0.01	0.15	0.50	0.42	0.68	-0.02	0.03	0.29	0.18	0.37	-0.01	0.04
	2020	1.27	1.21	1.30	-0.03	0.12	0.50	0.41	0.69	-0.02	0.04	0.32	0.22	0.40	-0.02	0.04

Supplementary Table 13. Simulated annual $\Delta c_{SIA}/\Delta c_{SO_4^{2-}}$, $\Delta c_{SIA}/\Delta c_{NO_3^T}$, and $\Delta c_{SIA}/\Delta c_{NH_4^T}$ with 40% reductions. "W US", "C US", "MW US", "NE US", "SE US" stand for the Western, Central, Midwestern, Northeastern, Southeastern US, respectively. The 25th and 75th percentiles (perc) of values from the sites within the corresponding region show their regional variability. Uncertainty lower and upper bounds (Unc LB and Unc UB) are the mean uncertainties (95% CI defined as the 2.5th and the 97.5th percentiles) of the sites within the region. Uncertainties of each site are estimated using 500 Monte Carlo simulations based on the precisions and detection limits shown in Supplementary Table 7.

		$\Delta c_{SIA}/\Delta c_{SO_4^{2-}}$ (40%)					$\Delta c_{SIA}/\Delta c_{NO_3^T}$ (40%)					$\Delta c_{SIA}/\Delta c_{NH_4^T}$ (40%)				
**Region**	**Year**	**Median**	**25th perc**	**75th perc**	**Unc LB**	**Unc UB**	**Median**	**25th perc**	**75th perc**	**Unc LB**	**Unc UB**	**Median**	**25th perc**	**75th perc**	**Unc LB**	**Unc UB**
W US (WRAP)	2011	1.27	1.15	1.35	-0.04	0.15	0.23	0.19	0.31	0.00	0.02	0.20	0.16	0.23	0.00	0.01
	2012	1.30	1.25	1.36	-0.09	0.17	0.26	0.17	0.40	0.00	0.01	0.17	0.12	0.20	0.00	0.01
	2013	1.37	1.28	1.38	-0.09	0.20	0.45	0.24	0.54	0.00	0.04	0.16	0.12	0.18	0.00	0.01
	2014	1.22	0.88	1.39	-0.14	0.20	0.33	0.17	0.50	-0.01	0.05	0.15	0.11	0.17	0.00	0.01
	2015	1.35	1.26	1.38	-0.09	0.19	0.41	0.25	0.43	-0.01	0.03	0.16	0.14	0.18	0.00	0.01
	2016	1.25	1.14	1.35	-0.15	0.28	0.40	0.26	0.42	-0.02	0.04	0.14	0.10	0.19	0.00	0.01
	2017	1.21	0.89	1.30	-0.15	0.25	0.41	0.25	0.45	-0.03	0.05	0.14	0.10	0.15	0.00	0.01
	2018	0.93	0.60	1.17	-0.22	0.32	0.39	0.34	0.44	-0.05	0.07	0.13	0.09	0.17	0.00	0.01
	2019	1.34	0.99	1.37	-0.10	0.19	0.44	0.28	0.51	-0.02	0.03	0.19	0.09	0.25	0.00	0.03
	2020	0.65	0.39	0.80	-0.17	0.27	0.44	0.41	0.51	-0.06	0.08	0.13	0.08	0.17	0.00	0.02
C US (CENSARA)	2011	1.30	1.29	1.32	0.00	0.00	0.55	0.47	0.64	0.00	0.01	0.68	0.66	0.71	-0.02	0.04
	2012	1.37	1.34	1.39	-0.02	0.06	0.59	0.46	0.59	0.00	0.01	0.34	0.29	0.41	-0.01	0.01
	2013	1.36	1.34	1.37	-0.01	0.03	0.72	0.62	0.79	0.00	0.00	0.40	0.31	0.46	-0.01	0.02
	2014	1.34	1.30	1.37	-0.02	0.04	0.78	0.75	0.81	0.00	0.01	0.27	0.21	0.38	-0.01	0.01
	2015	1.37	1.33	1.41	-0.03	0.08	0.68	0.64	0.76	-0.01	0.02	0.24	0.19	0.47	0.00	0.02
	2016	1.34	1.31	1.41	-0.02	0.11	0.64	0.60	0.76	-0.01	0.01	0.23	0.16	0.39	-0.01	0.01
	2017	1.39	1.32	1.41	-0.02	0.10	0.63	0.52	0.82	0.00	0.01	0.20	0.14	0.31	0.00	0.02
	2018	1.34	1.30	1.35	-0.04	0.13	0.71	0.55	0.85	0.00	0.01	0.16	0.13	0.42	0.00	0.02
	2019	1.37	1.31	1.37	-0.03	0.13	0.76	0.63	0.82	-0.01	0.02	0.24	0.12	0.53	-0.01	0.03
	2020	1.19	1.16	1.25	-0.08	0.12	0.69	0.49	0.82	-0.02	0.02	0.21	0.12	0.39	0.00	0.03
MW US (LADCO)	2011	1.27	1.20	1.34	-0.02	0.04	0.69	0.63	0.73	-0.01	0.01	0.86	0.47	1.23	-0.01	0.02
	2012	1.37	1.35	1.38	0.00	0.04	0.81	0.76	0.84	0.00	0.00	0.50	0.42	0.77	-0.01	0.02
	2013	1.36	1.34	1.37	0.00	0.01	0.96	0.88	0.98	0.00	0.00	0.58	0.58	0.99	-0.02	0.03
	2014	1.37	1.31	1.37	-0.01	0.02	0.99	0.90	1.02	0.00	0.01	0.54	0.46	0.98	-0.02	0.02
	2015	1.35	1.29	1.38	-0.02	0.04	0.86	0.78	0.96	-0.01	0.01	0.58	0.46	1.14	-0.02	0.03
	2016	1.36	1.33	1.39	-0.01	0.06	0.85	0.78	0.95	0.00	0.01	0.56	0.37	0.83	-0.01	0.02
	2017	1.38	1.33	1.39	-0.03	0.06	0.88	0.82	0.95	0.00	0.01	0.40	0.25	0.69	-0.01	0.02
	2018	1.36	1.30	1.38	-0.02	0.07	0.94	0.89	1.00	0.00	0.01	0.52	0.23	0.86	-0.01	0.04
	2019	1.36	1.32	1.38	-0.02	0.07	0.93	0.87	1.00	-0.01	0.01	0.52	0.34	1.01	-0.02	0.05
	2020	1.31	1.29	1.37	-0.05	0.11	0.87	0.81	0.92	-0.01	0.02	0.42	0.32	0.83	-0.02	0.04
NE US (MANE-VU)	2011	1.15	1.06	1.20	-0.01	0.01	0.46	0.38	0.58	-0.01	0.02	1.43	1.26	1.63	-0.03	0.05
	2012	1.22	1.17	1.26	-0.01	0.01	0.65	0.54	0.67	-0.01	0.02	1.29	0.95	1.39	-0.02	0.03
	2013	1.27	1.19	1.29	-0.01	0.02	0.68	0.60	0.78	-0.01	0.02	0.98	0.85	1.35	-0.02	0.04
	2014	1.24	1.18	1.28	-0.02	0.02	0.74	0.64	0.80	-0.02	0.02	1.11	0.86	1.34	-0.03	0.04
	2015	1.18	1.11	1.22	-0.02	0.03	0.61	0.53	0.70	-0.02	0.04	1.13	0.84	1.31	-0.03	0.04
	2016	1.26	1.23	1.29	-0.01	0.03	0.65	0.51	0.70	-0.01	0.02	0.79	0.57	0.93	-0.02	0.03
	2017	1.28	1.26	1.30	-0.01	0.02	0.73	0.62	0.81	-0.01	0.02	0.71	0.58	0.99	-0.02	0.03
	2018	1.26	1.23	1.29	-0.02	0.03	0.79	0.66	0.87	-0.01	0.03	0.88	0.58	1.12	-0.03	0.05
	2019	1.23	1.18	1.28	-0.03	0.05	0.69	0.64	0.83	-0.02	0.04	0.85	0.56	1.24	-0.04	0.07
	2020	1.23	1.22	1.27	-0.03	0.06	0.65	0.59	0.71	-0.02	0.04	0.76	0.45	1.03	-0.03	0.05
SE US (SESARM)	2011	1.27	0.94	1.30	0.00	0.04	0.49	0.37	0.66	-0.01	0.02	0.52	0.19	0.79	-0.03	0.08
	2012	1.28	1.13	1.33	0.00	0.02	0.57	0.46	0.70	-0.01	0.01	0.49	0.26	0.68	-0.01	0.05
	2013	1.28	1.20	1.32	0.00	0.03	0.69	0.56	0.81	-0.01	0.01	0.54	0.20	0.77	-0.02	0.03
	2014	1.29	1.18	1.32	-0.01	0.02	0.66	0.56	0.75	-0.01	0.01	0.46	0.26	0.83	-0.01	0.02
	2015	1.25	1.20	1.31	-0.02	0.04	0.58	0.48	0.73	-0.01	0.02	0.56	0.26	0.68	-0.01	0.04
	2016	1.31	1.27	1.34	-0.01	0.06	0.55	0.44	0.70	-0.01	0.02	0.41	0.28	0.52	-0.01	0.02
	2017	1.31	1.25	1.32	-0.01	0.06	0.57	0.46	0.69	-0.01	0.01	0.38	0.22	0.51	-0.01	0.02
	2018	1.29	1.24	1.32	-0.01	0.06	0.62	0.50	0.76	-0.01	0.02	0.47	0.25	0.66	-0.02	0.03
	2019	1.31	1.26	1.34	-0.02	0.08	0.51	0.44	0.73	-0.01	0.03	0.42	0.23	0.52	-0.02	0.04
	2020	1.28	1.22	1.31	-0.04	0.09	0.52	0.44	0.69	-0.01	0.03	0.43	0.22	0.53	-0.02	0.04

Supplementary Table 14. Simulated annual $\Delta c_{SIA}/\Delta c_{SO_4^{2-}}$, $\Delta c_{SIA}/\Delta c_{NO_3^T}$, and $\Delta c_{SIA}/\Delta c_{NH_4^T}$ with 70% reductions. "W US", "C US", "MW US", "NE US", "SE US" stand for the Western, Central, Midwestern, Northeastern, Southeastern US, respectively. The 25th and 75th percentiles (perc) of values from the sites within the corresponding region show their regional variability. Uncertainty lower and upper bounds (Unc LB and Unc UB) are the mean uncertainties (95% CI defined as the 2.5th and the 97.5th percentiles) of the sites within the region. Uncertainties of each site are estimated using 500 Monte Carlo simulations based on the precisions and detection limits shown in Supplementary Table 7.

		$\Delta c_{SIA}/\Delta c_{SO_4^{2-}}$ (70%)					$\Delta c_{SIA}/\Delta c_{NO_3^T}$ (70%)					$\Delta c_{SIA}/\Delta c_{NH_4^T}$ (70%)				
**Region**	**Year**	**Median**	**25th perc**	**75th perc**	**Unc LB**	**Unc UB**	**Median**	**25th perc**	**75th perc**	**Unc LB**	**Unc UB**	**Median**	**25th perc**	**75th perc**	**Unc LB**	**Unc UB**
W US (WRAP)	2011	1.00	0.75	1.24	-0.11	0.17	0.14	0.19	0.31	-0.01	0.02	0.27	0.22	0.29	-0.01	0.01
	2012	0.75	0.60	1.24	-0.10	0.21	0.12	0.17	0.39	0.00	0.02	0.22	0.14	0.27	0.00	0.01
	2013	1.09	0.93	1.06	-0.07	0.13	0.14	0.24	0.53	0.00	0.04	0.21	0.18	0.27	-0.01	0.02
	2014	0.81	0.26	1.05	-0.06	0.12	0.11	0.17	0.49	-0.01	0.04	0.19	0.16	0.22	0.00	0.01
	2015	0.94	0.64	1.17	-0.05	0.14	0.08	0.24	0.43	-0.01	0.03	0.22	0.18	0.28	0.00	0.01
	2016	0.44	0.10	0.82	-0.09	0.11	0.07	0.26	0.43	-0.02	0.04	0.20	0.12	0.27	0.00	0.01
	2017	0.35	0.17	0.60	-0.07	0.13	0.09	0.23	0.44	-0.03	0.05	0.16	0.15	0.26	0.00	0.01
	2018	-0.15	-0.48	-0.15	-0.09	0.15	0.12	0.35	0.43	-0.04	0.07	0.17	0.14	0.23	0.00	0.01
	2019	0.56	0.14	1.18	-0.07	0.11	0.09	0.28	0.50	-0.02	0.03	0.26	0.12	0.38	-0.01	0.03
	2020	0.06	-0.12	0.27	-0.08	0.14	0.08	0.40	0.52	-0.05	0.06	0.19	0.10	0.24	-0.01	0.01
C US (CENSARA)	2011	1.29	1.27	1.33	-0.02	0.02	0.13	0.47	0.64	0.00	0.01	1.00	0.98	1.02	-0.02	0.03
	2012	1.29	1.19	1.34	-0.02	0.06	0.11	0.46	0.58	0.00	0.01	0.59	0.52	0.64	-0.01	0.02
	2013	1.34	1.32	1.31	-0.02	0.04	0.22	0.62	0.79	0.00	0.00	0.79	0.72	0.79	-0.02	0.02
	2014	1.29	1.28	1.32	-0.03	0.07	0.31	0.74	0.80	0.00	0.01	0.59	0.44	0.71	-0.02	0.02
	2015	1.26	1.21	1.33	-0.04	0.08	0.27	0.64	0.75	-0.01	0.02	0.48	0.40	0.76	-0.01	0.02
	2016	1.12	0.90	1.20	-0.04	0.06	0.24	0.60	0.75	-0.01	0.01	0.43	0.31	0.55	-0.01	0.03
	2017	1.11	0.98	1.28	-0.03	0.05	0.30	0.52	0.80	0.00	0.01	0.36	0.27	0.54	-0.01	0.02
	2018	0.98	0.96	1.01	-0.04	0.07	0.24	0.55	0.84	-0.01	0.01	0.34	0.29	0.56	-0.01	0.03
	2019	0.90	0.77	1.07	-0.05	0.06	0.31	0.63	0.80	-0.01	0.02	0.53	0.23	0.74	-0.01	0.03
	2020	0.70	0.56	0.91	-0.04	0.06	0.21	0.49	0.81	-0.01	0.02	0.33	0.20	0.42	-0.01	0.06
MW US (LADCO)	2011	1.31	1.26	1.39	-0.01	0.02	0.32	0.67	0.76	-0.01	0.01	0.97	0.64	1.32	-0.01	0.02
	2012	1.36	1.33	1.40	-0.02	0.05	0.27	0.77	0.83	0.00	0.00	0.87	0.77	1.10	-0.01	0.03
	2013	1.37	1.34	1.41	-0.01	0.03	0.49	0.89	0.98	0.00	0.00	1.10	1.08	1.33	-0.02	0.02
	2014	1.37	1.34	1.41	-0.01	0.04	0.50	0.92	1.01	0.00	0.01	1.02	0.97	1.34	-0.02	0.02
	2015	1.33	1.31	1.40	-0.03	0.04	0.41	0.82	0.95	0.00	0.01	0.85	0.77	1.36	-0.02	0.02
	2016	1.33	1.30	1.41	-0.04	0.06	0.33	0.79	0.95	0.00	0.01	0.84	0.58	1.05	-0.01	0.02
	2017	1.32	1.25	1.41	-0.04	0.06	0.35	0.81	0.95	0.00	0.01	0.74	0.51	1.09	-0.01	0.02
	2018	1.32	1.28	1.41	-0.04	0.05	0.39	0.90	0.99	0.00	0.01	0.86	0.60	1.21	-0.01	0.03
	2019	1.33	1.30	1.40	-0.05	0.06	0.35	0.89	1.00	0.00	0.01	0.96	0.66	1.29	-0.01	0.03
	2020	1.20	1.12	1.37	-0.05	0.06	0.29	0.81	0.91	-0.01	0.02	0.74	0.57	1.10	-0.02	0.03
NE US (MANE-VU)	2011	1.22	1.15	1.34	-0.01	0.01	0.30	0.46	0.63	-0.01	0.02	1.42	1.36	1.45	-0.02	0.03
	2012	1.25	1.22	1.36	-0.01	0.01	0.34	0.60	0.70	-0.01	0.01	1.35	1.17	1.37	-0.01	0.02
	2013	1.29	1.24	1.36	-0.01	0.03	0.29	0.64	0.79	-0.01	0.02	1.21	1.01	1.43	-0.02	0.03
	2014	1.29	1.23	1.37	-0.01	0.02	0.38	0.71	0.82	-0.01	0.02	1.22	1.13	1.45	-0.02	0.03
	2015	1.22	1.16	1.37	-0.02	0.03	0.30	0.59	0.75	-0.01	0.03	1.26	1.01	1.37	-0.02	0.04
	2016	1.27	1.25	1.38	-0.03	0.05	0.25	0.53	0.71	-0.01	0.02	0.96	0.81	1.07	-0.02	0.03
	2017	1.28	1.20	1.38	-0.02	0.06	0.33	0.64	0.81	-0.01	0.01	1.01	0.80	1.24	-0.02	0.03
	2018	1.26	1.21	1.36	-0.03	0.06	0.34	0.69	0.89	-0.01	0.02	1.07	0.90	1.32	-0.03	0.04
	2019	1.24	1.12	1.36	-0.04	0.07	0.23	0.65	0.84	-0.02	0.03	1.11	0.91	1.34	-0.03	0.06
	2020	1.20	1.15	1.33	-0.05	0.06	0.18	0.60	0.73	-0.02	0.03	0.93	0.69	1.13	-0.02	0.05
SE US (SESARM)	2011	1.29	1.07	1.35	-0.01	0.04	0.30	0.38	0.68	-0.01	0.02	0.72	0.31	0.90	-0.02	0.05
	2012	1.30	1.21	1.36	-0.01	0.04	0.32	0.48	0.71	-0.01	0.01	0.72	0.35	0.94	-0.02	0.05
	2013	1.29	1.24	1.36	-0.01	0.03	0.40	0.56	0.83	-0.01	0.01	0.73	0.33	0.98	-0.02	0.05
	2014	1.31	1.22	1.36	-0.01	0.03	0.38	0.56	0.80	-0.01	0.01	0.63	0.38	0.92	-0.02	0.05
	2015	1.24	1.18	1.36	-0.02	0.04	0.31	0.52	0.76	-0.01	0.02	0.60	0.37	0.87	-0.02	0.05
	2016	1.28	1.19	1.35	-0.04	0.06	0.25	0.45	0.71	-0.01	0.01	0.52	0.29	0.60	-0.01	0.04
	2017	1.26	1.15	1.37	-0.04	0.06	0.25	0.47	0.73	0.00	0.01	0.49	0.25	0.66	-0.01	0.04
	2018	1.25	1.13	1.35	-0.04	0.07	0.29	0.52	0.78	-0.01	0.02	0.68	0.28	0.78	-0.02	0.04
	2019	1.25	1.15	1.35	-0.04	0.07	0.24	0.46	0.75	-0.01	0.03	0.52	0.27	0.70	-0.02	0.05
	2020	1.13	0.92	1.32	-0.05	0.07	0.27	0.45	0.72	-0.01	0.03	0.53	0.35	0.64	-0.02	0.05

Supplementary Table 15. Simulated summer $\Delta c_{SIA}/\Delta c_{SO_4^{2-}}$, $\Delta c_{SIA}/\Delta c_{NO_3^T}$, and $\Delta c_{SIA}/\Delta c_{NH_4^T}$ with 10% reductions. "W US", "C US", "MW US", "NE US", "SE US" stand for the Western, Central, Midwestern, Northeastern, Southeastern US, respectively. The 25th and 75th percentiles (perc) of values from the sites within the corresponding region show their regional variability. Uncertainty lower and upper bounds (Unc LB and Unc UB) are the mean uncertainties (95% CI defined as the 2.5th and the 97.5th percentiles) of the sites within the region. Uncertainties of each site are estimated using 500 Monte Carlo simulations based on the precisions and detection limits shown in Supplementary Table 7.

		$\Delta c_{SIA}/\Delta c_{SO_4^{2-}}$ (10%)					$\Delta c_{SIA}/\Delta c_{NO_3^T}$ (10%)					$\Delta c_{SIA}/\Delta c_{NH_4^T}$ (10%)				
**Region**	**Year**	**Median**	**25th perc**	**75th perc**	**Unc LB**	**Unc UB**	**Median**	**25th perc**	**75th perc**	**Unc LB**	**Unc UB**	**Median**	**25th perc**	**75th perc**	**Unc LB**	**Unc UB**
W US (WRAP)	2011	1.31	0.71	1.37	-0.02	0.55	0.14	0.10	0.17	0.00	0.05	0.07	0.06	0.08	0.00	0.00
	2012	1.37	1.37	1.39	-0.04	0.32	0.13	0.11	0.13	0.00	0.01	0.06	0.05	0.07	0.00	0.00
	2013	1.37	1.34	1.41	-0.17	0.90	0.15	0.11	0.17	0.00	0.13	0.06	0.05	0.08	0.00	0.00
	2014	1.36	1.28	1.38	-0.11	0.83	0.12	0.09	0.20	0.00	0.09	0.05	0.04	0.07	0.00	0.00
	2015	1.37	1.34	1.38	-0.05	0.32	0.09	0.07	0.12	0.00	0.01	0.05	0.04	0.07	0.00	0.00
	2016	1.33	1.27	1.37	-0.19	1.90	0.07	0.05	0.11	0.00	0.15	0.04	0.02	0.05	0.00	0.00
	2017	1.37	1.27	1.38	-0.38	1.35	0.10	0.08	0.13	0.00	0.10	0.04	0.03	0.05	0.00	0.00
	2018	1.33	1.29	1.36	-1.09	2.32	0.13	0.10	0.22	-0.05	0.15	0.04	0.03	0.05	0.00	0.00
	2019	1.37	1.30	1.37	-0.03	0.36	0.09	0.06	0.15	0.00	0.01	0.04	0.03	0.06	0.00	0.00
	2020	1.33	1.10	1.35	-1.07	1.59	0.08	0.05	0.11	-0.09	0.13	0.03	0.02	0.04	0.00	0.00
C US (CENSARA)	2011	1.32	1.31	1.33	0.00	0.00	0.13	0.12	0.14	0.00	0.00	0.16	0.12	0.20	-0.01	0.04
	2012	1.36	1.34	1.37	0.00	0.01	0.11	0.10	0.13	0.00	0.00	0.08	0.07	0.12	0.00	0.00
	2013	1.35	1.33	1.36	-0.01	0.03	0.23	0.22	0.24	-0.01	0.01	0.12	0.11	0.16	0.00	0.00
	2014	1.36	1.34	1.37	0.00	0.02	0.33	0.29	0.35	-0.01	0.01	0.12	0.11	0.14	0.00	0.00
	2015	1.34	1.32	1.37	-0.02	0.03	0.28	0.17	0.36	-0.01	0.01	0.12	0.12	0.14	0.00	0.01
	2016	1.35	1.34	1.38	-0.02	0.08	0.25	0.15	0.31	-0.01	0.01	0.09	0.07	0.11	0.00	0.01
	2017	1.37	1.34	1.40	-0.01	0.06	0.31	0.19	0.36	0.00	0.01	0.07	0.07	0.12	0.00	0.00
	2018	1.34	1.32	1.37	-0.08	0.30	0.24	0.14	0.31	-0.01	0.01	0.08	0.06	0.14	0.00	0.01
	2019	1.35	1.33	1.37	-0.03	0.15	0.33	0.22	0.40	-0.01	0.01	0.10	0.08	0.12	0.00	0.01
	2020	1.35	1.27	1.36	-0.13	0.36	0.21	0.16	0.30	-0.01	0.02	0.09	0.08	0.11	0.00	0.05
MW US (LADCO)	2011	1.34	1.30	1.37	0.00	0.01	0.32	0.30	0.38	0.00	0.01	0.27	0.17	0.37	0.00	0.01
	2012	1.38	1.37	1.39	0.00	0.02	0.28	0.26	0.37	0.00	0.01	0.14	0.11	0.20	0.00	0.00
	2013	1.37	1.36	1.39	0.00	0.01	0.51	0.41	0.57	0.00	0.01	0.17	0.14	0.26	0.00	0.00
	2014	1.37	1.36	1.39	0.00	0.01	0.52	0.46	0.55	0.00	0.01	0.14	0.11	0.21	0.00	0.00
	2015	1.37	1.36	1.39	0.00	0.01	0.43	0.35	0.48	-0.01	0.01	0.16	0.11	0.20	0.00	0.00
	2016	1.39	1.38	1.41	-0.01	0.06	0.35	0.29	0.47	0.00	0.01	0.11	0.07	0.12	0.00	0.00
	2017	1.40	1.38	1.41	-0.01	0.05	0.38	0.29	0.45	0.00	0.01	0.09	0.07	0.11	0.00	0.00
	2018	1.39	1.37	1.41	-0.01	0.06	0.40	0.33	0.51	-0.01	0.02	0.10	0.07	0.12	0.00	0.00
	2019	1.41	1.38	1.42	-0.01	0.11	0.38	0.31	0.50	-0.01	0.01	0.09	0.07	0.12	0.00	0.00
	2020	1.39	1.37	1.40	-0.01	0.51	0.32	0.26	0.36	-0.01	0.02	0.08	0.07	0.13	0.00	0.00
NE US (MANE-VU)	2011	1.26	1.20	1.30	0.00	0.01	0.27	0.25	0.29	-0.01	0.01	0.45	0.22	0.64	-0.01	0.07
	2012	1.32	1.30	1.35	0.00	0.00	0.36	0.21	0.38	0.00	0.01	0.22	0.18	0.31	0.00	0.01
	2013	1.34	1.32	1.34	0.00	0.00	0.29	0.25	0.42	-0.01	0.01	0.19	0.15	0.26	0.00	0.01
	2014	1.35	1.34	1.36	0.00	0.01	0.39	0.32	0.41	-0.01	0.01	0.15	0.10	0.21	0.00	0.00
	2015	1.35	1.34	1.36	0.00	0.00	0.31	0.17	0.36	0.00	0.01	0.14	0.10	0.18	0.00	0.01
	2016	1.36	1.35	1.37	0.00	0.01	0.26	0.18	0.32	0.00	0.01	0.11	0.09	0.14	0.00	0.00
	2017	1.37	1.35	1.37	0.00	0.02	0.34	0.23	0.51	-0.01	0.01	0.11	0.08	0.13	0.00	0.00
	2018	1.35	1.33	1.36	0.00	0.06	0.35	0.19	0.41	-0.01	0.02	0.11	0.08	0.14	0.00	0.01
	2019	1.36	1.35	1.37	0.00	0.08	0.24	0.15	0.32	-0.01	0.01	0.09	0.07	0.11	0.00	0.01
	2020	1.35	1.34	1.36	-0.01	0.10	0.18	0.16	0.32	-0.01	0.02	0.08	0.08	0.11	0.00	0.01
SE US (SESARM)	2011	1.30	0.94	1.32	0.00	0.26	0.30	0.20	0.51	0.00	0.03	0.20	0.12	0.22	0.00	0.12
	2012	1.33	1.23	1.35	0.00	0.02	0.32	0.19	0.39	0.00	0.01	0.14	0.12	0.16	0.00	0.01
	2013	1.33	1.26	1.35	0.00	0.04	0.41	0.21	0.53	-0.01	0.02	0.11	0.09	0.16	0.00	0.01
	2014	1.33	1.22	1.35	0.00	0.01	0.38	0.24	0.50	-0.01	0.01	0.11	0.08	0.16	0.00	0.00
	2015	1.33	1.30	1.36	0.00	0.05	0.32	0.16	0.51	-0.01	0.01	0.11	0.08	0.13	0.00	0.01
	2016	1.35	1.33	1.36	0.00	0.02	0.25	0.17	0.39	-0.01	0.01	0.09	0.06	0.11	0.00	0.01
	2017	1.36	1.34	1.37	0.00	0.01	0.26	0.20	0.39	0.00	0.01	0.08	0.05	0.11	0.00	0.00
	2018	1.35	1.34	1.36	0.00	0.02	0.30	0.19	0.44	-0.01	0.01	0.08	0.06	0.11	0.00	0.01
	2019	1.35	1.33	1.36	0.00	0.03	0.25	0.18	0.41	-0.01	0.01	0.09	0.06	0.11	0.00	0.01
	2020	1.35	1.31	1.36	-0.01	0.22	0.29	0.14	0.44	-0.01	0.02	0.09	0.07	0.13	0.00	0.01

Supplementary Table 16. Simulated summer $\Delta c_{\mathrm{SIA}}/\Delta c_{\mathrm{SO_4^{2-}}}$, $\Delta c_{\mathrm{SIA}}/\Delta c_{\mathrm{NO_3^T}}$, and $\Delta c_{\mathrm{SIA}}/\Delta c_{\mathrm{NH_4^T}}$ with 40% reductions. “W US”, “C US”, “MW US”, “NE US”, “SE US” stand for the Western, Central, Midwestern, Northeastern, Southeastern US, respectively. The 25th and 75th percentiles (perc) of values from the sites within the corresponding region show their regional variability. Uncertainty lower and upper bounds (Unc LB and Unc UB) are the mean uncertainties (95% CI defined as the 2.5th and the 97.5th percentiles) of the sites within the region. Uncertainties of each site are estimated using 500 Monte Carlo simulations based on the precisions and detection limits shown in Supplementary Table 7.

		$\Delta c_{\mathrm{SIA}}/\Delta c_{\mathrm{SO_4^{2-}}}$ (40%)					$\Delta c_{\mathrm{SIA}}/\Delta c_{\mathrm{NO_3^T}}$ (40%)					$\Delta c_{\mathrm{SIA}}/\Delta c_{\mathrm{NH_4^T}}$ (40%)				
**RPO**	**Year**	**Median**	**25th perc**	**75th perc**	**Unc LB**	**Unc UB**	**Median**	**25th perc**	**75th perc**	**Unc LB**	**Unc UB**	**Median**	**25th perc**	**75th perc**	**Unc LB**	**Unc UB**
W US (WRAP)	2011	1.19	0.96	1.36	-0.05	0.29	0.14	0.10	0.17	0.00	0.04	0.08	0.06	0.10	0.00	0.00
	2012	1.36	1.35	1.38	-0.06	0.33	0.12	0.11	0.13	0.00	0.01	0.07	0.05	0.08	0.00	0.00
	2013	1.35	1.33	1.36	-0.12	0.29	0.15	0.11	0.17	0.00	0.11	0.07	0.06	0.09	0.00	0.00
	2014	1.22	1.07	1.37	-0.22	0.43	0.11	0.09	0.20	0.00	0.08	0.06	0.04	0.09	0.00	0.00
	2015	1.35	1.34	1.37	-0.09	0.22	0.09	0.07	0.12	0.00	0.01	0.05	0.05	0.09	0.00	0.00
	2016	1.27	1.19	1.31	-0.39	0.69	0.07	0.05	0.11	0.00	0.13	0.04	0.02	0.06	0.00	0.00
	2017	1.09	0.92	1.36	-0.32	0.53	0.10	0.08	0.13	0.00	0.10	0.04	0.03	0.06	0.00	0.00
	2018	0.78	0.62	1.22	-0.47	0.67	0.12	0.10	0.22	-0.05	0.16	0.04	0.03	0.05	0.00	0.00
	2019	1.35	1.19	1.36	-0.11	0.30	0.09	0.06	0.15	-0.01	0.01	0.04	0.03	0.06	0.00	0.00
	2020	0.78	0.29	1.24	-0.38	0.51	0.08	0.05	0.11	-0.11	0.15	0.03	0.02	0.05	0.00	0.00
C US (CENSARA)	2011	1.33	1.32	1.34	0.00	0.01	0.13	0.13	0.14	0.00	0.00	0.30	0.20	0.39	-0.01	0.02
	2012	1.36	1.35	1.37	0.00	0.09	0.11	0.10	0.13	0.00	0.00	0.10	0.09	0.13	0.00	0.01
	2013	1.35	1.33	1.35	-0.03	0.08	0.22	0.21	0.24	-0.01	0.01	0.13	0.13	0.15	0.00	0.05
	2014	1.36	1.34	1.36	-0.01	0.05	0.32	0.28	0.35	-0.01	0.01	0.15	0.14	0.18	0.00	0.01
	2015	1.33	1.32	1.37	-0.05	0.12	0.27	0.17	0.36	-0.01	0.01	0.14	0.13	0.18	0.00	0.01
	2016	1.34	1.32	1.38	-0.06	0.21	0.24	0.15	0.30	-0.01	0.01	0.10	0.08	0.15	0.00	0.01
	2017	1.36	1.34	1.37	-0.03	0.18	0.30	0.19	0.36	0.00	0.01	0.09	0.08	0.16	0.00	0.01
	2018	1.31	1.17	1.37	-0.11	0.34	0.24	0.15	0.30	-0.01	0.01	0.07	0.05	0.10	0.00	0.01
	2019	1.34	1.32	1.36	-0.05	0.37	0.32	0.22	0.40	-0.01	0.01	0.10	0.07	0.11	0.00	0.03
	2020	1.29	1.17	1.35	-0.12	0.27	0.20	0.15	0.29	-0.01	0.02	0.09	0.06	0.11	0.00	0.06
MW US (LADCO)	2011	1.36	1.33	1.38	0.00	0.02	0.32	0.30	0.38	0.00	0.01	0.32	0.20	0.48	0.00	0.01
	2012	1.39	1.39	1.40	0.00	0.11	0.28	0.26	0.36	0.00	0.01	0.17	0.13	0.24	0.00	0.00
	2013	1.38	1.38	1.40	0.00	0.01	0.50	0.41	0.55	0.00	0.01	0.24	0.18	0.33	0.00	0.00
	2014	1.38	1.37	1.40	0.00	0.01	0.51	0.46	0.54	0.00	0.01	0.19	0.13	0.30	0.00	0.00
	2015	1.39	1.37	1.40	0.00	0.02	0.42	0.35	0.47	-0.01	0.01	0.21	0.14	0.26	0.00	0.01
	2016	1.40	1.39	1.41	-0.01	0.19	0.34	0.29	0.46	0.00	0.01	0.13	0.09	0.15	0.00	0.00
	2017	1.40	1.38	1.42	-0.01	0.15	0.36	0.28	0.43	0.00	0.01	0.11	0.08	0.13	0.00	0.00
	2018	1.39	1.38	1.41	-0.02	0.21	0.40	0.32	0.50	-0.01	0.02	0.12	0.08	0.15	0.00	0.00
	2019	1.40	1.39	1.42	-0.01	0.28	0.37	0.30	0.49	-0.01	0.01	0.11	0.09	0.13	0.00	0.00
	2020	1.39	1.37	1.40	-0.09	0.28	0.31	0.25	0.35	-0.01	0.03	0.09	0.08	0.15	0.00	0.01
NE US (MANE-VU)	2011	1.30	1.26	1.32	0.00	0.01	0.28	0.26	0.30	-0.01	0.01	0.73	0.30	0.89	-0.02	0.06
	2012	1.34	1.32	1.36	0.00	0.00	0.35	0.21	0.39	0.00	0.01	0.32	0.23	0.49	-0.01	0.02
	2013	1.34	1.33	1.35	0.00	0.00	0.29	0.24	0.42	0.00	0.01	0.27	0.22	0.39	-0.01	0.03
	2014	1.35	1.34	1.36	0.00	0.02	0.39	0.32	0.40	-0.01	0.01	0.19	0.13	0.26	0.00	0.01
	2015	1.35	1.34	1.37	0.00	0.01	0.30	0.16	0.36	0.00	0.01	0.18	0.11	0.22	0.00	0.01
	2016	1.36	1.35	1.38	0.00	0.04	0.25	0.18	0.31	0.00	0.01	0.13	0.11	0.16	0.00	0.00
	2017	1.37	1.35	1.38	0.00	0.04	0.34	0.22	0.50	-0.01	0.01	0.12	0.10	0.16	0.00	0.01
	2018	1.35	1.32	1.37	0.00	0.08	0.35	0.18	0.41	-0.01	0.02	0.13	0.09	0.16	-0.01	0.02
	2019	1.36	1.35	1.37	0.00	0.08	0.23	0.15	0.31	-0.01	0.01	0.10	0.08	0.12	0.00	0.01
	2020	1.34	1.29	1.36	-0.02	0.13	0.18	0.16	0.31	-0.01	0.01	0.10	0.09	0.15	0.00	0.01
SE US (SESARM)	2011	1.32	1.07	1.34	0.00	0.07	0.30	0.20	0.52	0.00	0.03	0.27	0.14	0.46	-0.03	0.06
	2012	1.34	1.19	1.36	0.00	0.02	0.32	0.19	0.38	0.00	0.01	0.17	0.14	0.19	-0.01	0.12
	2013	1.34	1.18	1.35	0.00	0.04	0.40	0.21	0.53	-0.01	0.02	0.12	0.07	0.17	0.00	0.04
	2014	1.34	1.28	1.35	0.00	0.02	0.38	0.24	0.51	-0.01	0.01	0.15	0.09	0.22	0.00	0.01
	2015	1.34	1.25	1.36	0.00	0.07	0.31	0.16	0.52	-0.01	0.01	0.12	0.08	0.18	0.00	0.03
	2016	1.35	1.33	1.37	-0.01	0.05	0.25	0.17	0.39	0.00	0.01	0.12	0.07	0.13	0.00	0.01
	2017	1.36	1.34	1.38	0.00	0.04	0.26	0.19	0.39	0.00	0.01	0.10	0.05	0.13	0.00	0.01
	2018	1.35	1.33	1.37	-0.01	0.04	0.30	0.19	0.43	-0.01	0.01	0.11	0.06	0.14	0.00	0.02
	2019	1.35	1.32	1.36	-0.01	0.07	0.25	0.18	0.41	-0.01	0.01	0.11	0.06	0.13	0.00	0.02
	2020	1.33	1.30	1.36	-0.05	0.13	0.28	0.14	0.44	-0.01	0.02	0.10	0.06	0.14	0.00	0.04

Supplementary Table 17. Simulated summer $\Delta c_{\mathrm{SIA}}/\Delta c_{\mathrm{SO_4^{2-}}}$, $\Delta c_{\mathrm{SIA}}/\Delta c_{\mathrm{NO_3^T}}$, and $\Delta c_{\mathrm{SIA}}/\Delta c_{\mathrm{NH_4^T}}$ with 70% reductions. "W US", "C US", "MW US", "NE US", "SE US" stand for the Western, Central, Midwestern, Northeastern, Southeastern US, respectively. The 25th and 75th percentiles (perc) of values from the sites within the corresponding region show their regional variability. Uncertainty lower and upper bounds (Unc LB and Unc UB) are the mean uncertainties (95% CI defined as the 2.5th and the 97.5th percentiles) of the sites within the region. Uncertainties of each site are estimated using 500 Monte Carlo simulations based on the precisions and detection limits shown in Supplementary Table 7.

		$\Delta c_{\mathrm{SIA}}/\Delta c_{\mathrm{SO_4^{2-}}}$ (70%)					$\Delta c_{\mathrm{SIA}}/\Delta c_{\mathrm{NO_3^T}}$ (70%)					$\Delta c_{\mathrm{SIA}}/\Delta c_{\mathrm{NH_4^T}}$ (70%)				
**Region**	**Year**	**Median**	**25th perc**	**75th perc**	**Unc LB**	**Unc UB**	**Median**	**25th perc**	**75th perc**	**Unc LB**	**Unc UB**	**Median**	**25th perc**	**75th perc**	**Unc LB**	**Unc UB**
W US (WRAP)	2011	1.00	0.65	1.24	-0.16	0.29	0.14	0.10	0.17	0.00	0.03	0.08	0.06	0.11	0.00	0.01
	2012	1.21	0.92	1.24	-0.22	0.36	0.12	0.10	0.13	0.00	0.01	0.07	0.06	0.09	0.00	0.00
	2013	1.00	0.83	1.06	-0.16	0.27	0.14	0.11	0.17	0.00	0.11	0.08	0.06	0.11	0.00	0.00
	2014	0.58	0.28	1.05	-0.11	0.21	0.11	0.08	0.20	0.00	0.09	0.07	0.04	0.11	0.00	0.01
	2015	1.00	0.79	1.17	-0.11	0.25	0.08	0.07	0.12	0.00	0.01	0.05	0.05	0.09	0.00	0.00
	2016	-0.30	-0.87	0.82	-0.15	0.26	0.07	0.05	0.10	0.00	0.13	0.05	0.02	0.06	0.00	0.00
	2017	0.12	-0.22	0.60	-0.13	0.23	0.09	0.08	0.13	-0.01	0.11	0.04	0.03	0.06	0.00	0.00
	2018	-0.55	-0.88	-0.15	-0.20	0.31	0.12	0.10	0.22	-0.06	0.16	0.04	0.03	0.06	0.00	0.01
	2019	0.84	0.18	1.18	-0.09	0.25	0.09	0.06	0.14	-0.01	0.01	0.04	0.03	0.07	0.00	0.01
	2020	-0.10	-0.98	0.27	-0.16	0.24	0.08	0.05	0.11	-0.10	0.14	0.04	0.02	0.06	0.00	0.00
C US (CENSARA)	2011	1.31	1.29	1.33	-0.02	0.02	0.13	0.13	0.14	0.00	0.00	0.41	0.30	0.51	-0.04	0.04
	2012	1.33	1.21	1.34	-0.04	0.13	0.11	0.10	0.12	0.00	0.00	0.11	0.10	0.24	-0.01	0.05
	2013	1.30	1.29	1.31	-0.06	0.09	0.22	0.21	0.23	-0.01	0.01	0.22	0.19	0.36	-0.04	0.05
	2014	1.28	1.24	1.32	-0.06	0.15	0.31	0.27	0.34	-0.01	0.01	0.19	0.15	0.28	-0.04	0.07
	2015	1.24	1.19	1.33	-0.10	0.18	0.27	0.17	0.36	-0.01	0.01	0.16	0.04	0.19	-0.02	0.09
	2016	1.09	0.70	1.20	-0.09	0.17	0.24	0.15	0.29	-0.01	0.01	0.10	0.09	0.10	0.00	0.07
	2017	1.18	1.03	1.28	-0.04	0.08	0.30	0.19	0.35	-0.01	0.01	0.13	0.09	0.15	0.00	0.02
	2018	0.56	0.43	1.01	-0.06	0.13	0.24	0.16	0.29	-0.01	0.02	0.07	0.06	0.10	0.00	0.07
	2019	0.49	0.17	1.07	-0.08	0.13	0.31	0.21	0.39	-0.01	0.01	0.12	0.09	0.14	0.00	0.04
	2020	0.63	0.43	0.91	-0.06	0.13	0.21	0.16	0.28	-0.01	0.03	0.07	-0.01	0.11	0.00	0.17
MW US (LADCO)	2011	1.38	1.36	1.39	0.00	0.03	0.32	0.30	0.37	0.00	0.01	0.44	0.29	0.62	-0.01	0.03
	2012	1.40	1.36	1.40	-0.04	0.13	0.27	0.26	0.35	0.00	0.01	0.25	0.17	0.32	0.00	0.04
	2013	1.40	1.40	1.41	-0.02	0.06	0.49	0.41	0.54	0.00	0.01	0.44	0.27	0.48	-0.01	0.03
	2014	1.39	1.38	1.41	-0.03	0.07	0.50	0.45	0.53	0.00	0.01	0.33	0.20	0.40	-0.01	0.02
	2015	1.38	1.37	1.40	-0.05	0.10	0.41	0.34	0.46	-0.01	0.01	0.26	0.21	0.39	0.00	0.03
	2016	1.39	1.38	1.41	-0.09	0.14	0.33	0.28	0.45	0.00	0.01	0.16	0.14	0.19	0.00	0.01
	2017	1.39	1.21	1.41	-0.12	0.17	0.35	0.27	0.42	-0.01	0.01	0.14	0.11	0.18	0.00	0.01
	2018	1.39	1.16	1.41	-0.11	0.16	0.39	0.31	0.48	-0.01	0.02	0.17	0.10	0.19	0.00	0.01
	2019	1.39	1.37	1.40	-0.11	0.17	0.35	0.29	0.47	-0.01	0.01	0.14	0.12	0.16	0.00	0.01
	2020	1.09	1.02	1.37	-0.13	0.15	0.29	0.24	0.34	-0.01	0.03	0.12	0.10	0.19	0.00	0.05
NE US (MANE-VU)	2011	1.32	1.30	1.34	0.00	0.01	0.30	0.26	0.32	0.00	0.01	0.85	0.64	0.95	-0.01	0.02
	2012	1.34	1.33	1.36	0.00	0.01	0.34	0.21	0.39	0.00	0.01	0.46	0.34	0.70	-0.02	0.03
	2013	1.35	1.34	1.36	0.00	0.03	0.29	0.24	0.42	0.00	0.01	0.49	0.40	0.57	-0.02	0.03
	2014	1.35	1.34	1.37	-0.01	0.03	0.38	0.32	0.40	-0.01	0.01	0.27	0.25	0.43	-0.01	0.03
	2015	1.35	1.35	1.37	0.00	0.05	0.30	0.16	0.35	0.00	0.01	0.31	0.19	0.39	-0.02	0.03
	2016	1.36	1.34	1.38	-0.02	0.10	0.25	0.18	0.31	0.00	0.01	0.19	0.17	0.26	0.00	0.03
	2017	1.36	1.34	1.38	-0.03	0.09	0.33	0.22	0.49	-0.01	0.01	0.18	0.16	0.24	0.00	0.02
	2018	1.30	1.18	1.36	-0.06	0.14	0.34	0.18	0.40	-0.01	0.02	0.18	0.13	0.27	-0.01	0.04
	2019	1.34	1.27	1.36	-0.08	0.14	0.23	0.15	0.31	-0.01	0.01	0.14	0.11	0.19	-0.01	0.03
	2020	1.26	1.12	1.33	-0.10	0.14	0.18	0.16	0.31	-0.01	0.02	0.12	0.09	0.15	-0.01	0.04
SE US (SESARM)	2011	1.34	1.07	1.35	0.00	0.07	0.30	0.20	0.54	0.00	0.03	0.48	0.19	0.64	-0.02	0.05
	2012	1.35	1.25	1.36	0.00	0.07	0.32	0.19	0.41	0.00	0.01	0.30	0.19	0.52	-0.01	0.04
	2013	1.34	1.21	1.36	-0.02	0.05	0.40	0.21	0.54	-0.01	0.02	0.30	0.16	0.40	-0.02	0.05
	2014	1.35	1.27	1.36	-0.02	0.05	0.38	0.24	0.50	-0.01	0.01	0.32	0.10	0.46	-0.02	0.05
	2015	1.32	1.09	1.36	-0.04	0.09	0.31	0.15	0.51	-0.01	0.01	0.19	0.10	0.35	-0.02	0.11
	2016	1.32	1.21	1.35	-0.08	0.12	0.25	0.17	0.39	-0.01	0.01	0.13	0.07	0.20	-0.01	0.07
	2017	1.35	1.27	1.37	-0.05	0.13	0.25	0.19	0.38	0.00	0.01	0.12	0.06	0.18	-0.01	0.05
	2018	1.34	1.19	1.35	-0.08	0.16	0.29	0.19	0.42	-0.01	0.01	0.13	0.07	0.21	-0.01	0.08
	2019	1.33	1.22	1.35	-0.08	0.14	0.24	0.18	0.41	-0.01	0.01	0.14	0.07	0.18	-0.01	0.09
	2020	1.19	0.91	1.32	-0.08	0.12	0.27	0.13	0.43	-0.01	0.02	0.13	0.09	0.16	-0.02	0.08

Supplementary Table 18. Simulated winter $\Delta c_{\mathrm{SIA}}/\Delta c_{\mathrm{SO_4^{2-}}}$, $\Delta c_{\mathrm{SIA}}/\Delta c_{\mathrm{NO_3^T}}$, and $\Delta c_{\mathrm{SIA}}/\Delta c_{\mathrm{NH_4^T}}$ with 10% reductions. "W US", "C US", "MW US", "NE US", "SE US" stand for the Western, Central, Midwestern, Northeastern, Southeastern US, respectively. The 25th and 75th percentiles (perc) of values from the sites within the corresponding region show their regional variability. Uncertainty lower and upper bounds (Unc LB and Unc UB) are the mean uncertainties (95% CI defined as the 2.5th and the 97.5th percentiles) of the sites within the region. Uncertainties of each site are estimated using 500 Monte Carlo simulations based on the precisions and detection limits shown in Supplementary Table 7.

		$\Delta c_{\mathrm{SIA}}/\Delta c_{\mathrm{SO_4^{2-}}}$ (10%)					$\Delta c_{\mathrm{SIA}}/\Delta c_{\mathrm{NO_3^T}}$ (10%)					$\Delta c_{\mathrm{SIA}}/\Delta c_{\mathrm{NH_4^T}}$ (10%)				
**Region**	**Year**	**Median**	**25th perc**	**75th perc**	**Unc LB**	**Unc UB**	**Median**	**25th perc**	**75th perc**	**Unc LB**	**Unc UB**	**Median**	**25th perc**	**75th perc**	**Unc LB**	**Unc UB**
W US (WRAP)	2011	1.35	0.40	0.53	-0.03	0.08	0.54	0.50	0.66	-0.02	0.05	0.45	0.40	0.53	-0.03	0.08
	2012	1.43	0.22	0.39	-0.02	0.06	0.44	0.36	0.88	-0.03	0.06	0.36	0.22	0.39	-0.02	0.06
	2013	1.38	0.15	0.32	-0.03	0.04	0.80	0.54	0.98	-0.02	0.04	0.24	0.15	0.32	-0.03	0.04
	2014	1.38	0.11	0.36	-0.01	0.09	0.68	0.37	0.87	-0.03	0.07	0.20	0.11	0.36	-0.01	0.09
	2015	1.39	0.12	0.33	-0.01	0.06	0.79	0.38	0.84	-0.01	0.05	0.24	0.12	0.33	-0.01	0.06
	2016	1.35	0.16	0.40	-0.01	0.09	0.76	0.62	0.89	-0.04	0.06	0.34	0.16	0.40	-0.01	0.09
	2017	1.36	0.13	0.36	-0.02	0.06	0.76	0.49	0.85	-0.04	0.10	0.27	0.13	0.36	-0.02	0.06
	2018	1.35	0.15	0.32	-0.02	0.06	0.68	0.42	0.80	-0.05	0.09	0.29	0.15	0.32	-0.02	0.06
	2019	1.37	0.17	0.51	-0.03	0.12	0.80	0.65	0.98	-0.05	0.09	0.24	0.17	0.51	-0.03	0.12
	2020	1.37	0.19	0.34	-0.04	0.11	0.83	0.48	0.93	-0.07	0.09	0.21	0.19	0.34	-0.04	0.11
C US (CENSARA)	2011	1.22	0.57	0.83	-0.04	0.09	0.88	0.78	0.98	-0.01	0.03	0.70	0.57	0.83	-0.04	0.09
	2012	1.36	0.31	0.62	-0.01	0.03	0.96	0.77	1.02	-0.01	0.01	0.37	0.31	0.62	-0.01	0.03
	2013	1.34	0.24	0.53	-0.01	0.06	1.05	0.99	1.12	0.00	0.01	0.34	0.24	0.53	-0.01	0.06
	2014	1.34	0.14	0.36	-0.01	0.03	1.13	1.02	1.20	0.00	0.01	0.23	0.14	0.36	-0.01	0.03
	2015	1.36	0.14	0.51	-0.01	0.06	1.06	0.91	1.16	0.00	0.02	0.23	0.14	0.51	-0.01	0.06
	2016	1.35	0.13	0.60	-0.01	0.08	1.07	0.82	1.18	0.00	0.03	0.25	0.13	0.60	-0.01	0.08
	2017	1.38	0.11	0.39	-0.01	0.05	0.98	0.77	1.17	-0.01	0.02	0.25	0.11	0.39	-0.01	0.05
	2018	1.36	0.10	0.62	0.00	0.08	1.13	0.89	1.21	0.00	0.03	0.22	0.10	0.62	0.00	0.08
	2019	1.34	0.07	0.63	-0.01	0.15	1.11	0.82	1.23	0.00	0.05	0.26	0.07	0.63	-0.01	0.15
	2020	1.38	0.07	0.66	-0.01	0.15	1.06	0.73	1.22	-0.01	0.05	0.23	0.07	0.66	-0.01	0.15
MW US (LADCO)	2011	0.77	1.59	2.46	-0.06	0.13	0.66	0.49	0.91	-0.02	0.06	2.13	1.59	2.46	-0.06	0.13
	2012	1.29	0.31	0.84	-0.02	0.07	1.11	1.07	1.18	0.00	0.01	0.44	0.31	0.84	-0.02	0.07
	2013	1.30	0.15	0.74	-0.02	0.07	1.19	1.14	1.24	0.00	0.01	0.29	0.15	0.74	-0.02	0.07
	2014	1.30	0.11	1.83	-0.03	0.17	1.20	0.91	1.25	0.00	0.08	0.48	0.11	1.83	-0.03	0.17
	2015	1.20	0.29	1.93	-0.01	0.22	1.11	0.83	1.19	0.00	0.05	0.85	0.29	1.93	-0.01	0.22
	2016	1.22	0.25	1.34	-0.03	0.17	1.10	0.95	1.19	0.00	0.04	0.73	0.25	1.34	-0.03	0.17
	2017	1.30	0.23	0.68	-0.01	0.08	1.14	1.11	1.18	-0.01	0.02	0.35	0.23	0.68	-0.01	0.08
	2018	1.30	0.17	0.96	-0.01	0.16	1.20	1.12	1.23	0.00	0.02	0.35	0.17	0.96	-0.01	0.16
	2019	1.28	0.15	1.01	-0.03	0.25	1.19	1.07	1.23	-0.01	0.05	0.44	0.15	1.01	-0.03	0.25
	2020	1.28	0.22	1.06	-0.03	0.21	1.16	1.06	1.20	-0.01	0.05	0.46	0.22	1.06	-0.03	0.21
NE US (MANE-VU)	2011	0.57	2.34	3.25	-0.10	0.20	0.38	0.26	0.60	-0.03	0.04	2.64	2.34	3.25	-0.10	0.20
	2012	0.84	1.38	2.59	-0.08	0.13	0.72	0.53	0.77	-0.03	0.04	1.91	1.38	2.59	-0.08	0.13
	2013	1.10	0.97	2.41	-0.13	0.20	0.86	0.70	1.04	-0.03	0.06	1.30	0.97	2.41	-0.13	0.20
	2014	0.85	1.70	2.41	-0.18	0.28	0.78	0.64	0.83	-0.06	0.09	2.12	1.70	2.41	-0.18	0.28
	2015	0.78	1.47	2.74	-0.08	0.24	0.67	0.46	0.82	-0.03	0.08	2.25	1.47	2.74	-0.08	0.24
	2016	0.99	1.20	2.12	-0.15	0.22	0.75	0.65	0.89	-0.05	0.07	1.63	1.20	2.12	-0.15	0.22
	2017	1.15	0.71	1.54	-0.10	0.18	0.94	0.86	1.01	-0.03	0.05	1.08	0.71	1.54	-0.10	0.18
	2018	1.04	0.64	2.53	-0.08	0.34	0.91	0.83	1.08	-0.02	0.09	1.67	0.64	2.53	-0.08	0.34
	2019	1.17	0.51	1.96	-0.11	0.35	0.96	0.84	1.10	-0.04	0.10	0.95	0.51	1.96	-0.11	0.35
	2020	1.03	0.81	2.07	-0.19	0.34	0.84	0.70	0.91	-0.05	0.10	1.50	0.81	2.07	-0.19	0.34
SE US (SESARM)	2011	1.14	0.26	1.40	-0.08	0.11	0.60	0.51	0.68	-0.03	0.15	0.89	0.26	1.40	-0.08	0.11
	2012	1.13	0.42	1.19	-0.05	0.10	0.74	0.53	0.79	-0.02	0.04	0.76	0.42	1.19	-0.05	0.10
	2013	1.09	0.33	1.55	-0.04	0.10	0.81	0.69	0.86	-0.02	0.04	0.73	0.33	1.55	-0.04	0.10
	2014	1.10	0.34	1.64	-0.04	0.11	0.83	0.68	0.88	-0.02	0.05	0.67	0.34	1.64	-0.04	0.11
	2015	1.07	0.50	1.63	-0.05	0.15	0.71	0.59	0.88	-0.03	0.04	0.88	0.50	1.63	-0.05	0.15
	2016	1.15	0.43	1.24	-0.05	0.12	0.68	0.61	0.87	-0.03	0.05	0.82	0.43	1.24	-0.05	0.12
	2017	1.24	0.31	0.87	-0.03	0.08	0.74	0.61	0.84	-0.01	0.04	0.62	0.31	0.87	-0.03	0.08
	2018	1.10	0.42	1.39	-0.07	0.18	0.78	0.68	0.92	-0.03	0.06	0.73	0.42	1.39	-0.07	0.18
	2019	1.21	0.38	0.86	-0.07	0.20	0.70	0.64	0.88	-0.04	0.07	0.70	0.38	0.86	-0.07	0.20
	2020	1.16	0.45	1.25	-0.08	0.19	0.66	0.57	0.77	-0.05	0.09	0.84	0.45	1.25	-0.08	0.19

Supplementary Table 19. Simulated winter $\Delta c_{\mathrm{SIA}}/\Delta c_{\mathrm{SO_4^{2-}}}$, $\Delta c_{\mathrm{SIA}}/\Delta c_{\mathrm{NO_3^T}}$, and $\Delta c_{\mathrm{SIA}}/\Delta c_{\mathrm{NH_4^T}}$ with 40% reductions. "W US", "C US", "MW US", "NE US", "SE US" stand for the Western, Central, Midwestern, Northeastern, Southeastern US, respectively. The 25th and 75th percentiles (perc) of values from the sites within the corresponding region show their regional variability. Uncertainty lower and upper bounds (Unc LB and Unc UB) are the mean uncertainties (95% CI defined as the 2.5th and the 97.5th percentiles) of the sites within the region. Uncertainties of each site are estimated using 500 Monte Carlo simulations based on the precisions and detection limits shown in Supplementary Table 7.

		$\Delta c_{\mathrm{SIA}}/\Delta c_{\mathrm{SO_4^{2-}}}$ (40%)					$\Delta c_{\mathrm{SIA}}/\Delta c_{\mathrm{NO_3^T}}$ (40%)					$\Delta c_{\mathrm{SIA}}/\Delta c_{\mathrm{NH_4^T}}$ (40%)				
**Region**	**Year**	**Median**	**25th perc**	**75th perc**	**Unc LB**	**Unc UB**	**Median**	**25th perc**	**75th perc**	**Unc LB**	**Unc UB**	**Median**	**25th perc**	**75th perc**	**Unc LB**	**Unc UB**
W US (WRAP)	2011	1.19	1.13	1.36	-0.08	0.46	0.54	0.50	0.67	-0.02	0.03	0.64	0.53	0.75	-0.03	0.08
	2012	1.36	1.24	1.45	-0.08	0.36	0.43	0.36	0.89	-0.01	0.03	0.51	0.26	0.53	-0.01	0.04
	2013	1.35	1.37	1.45	-0.01	0.19	0.81	0.52	0.97	-0.01	0.02	0.35	0.22	0.38	-0.02	0.04
	2014	1.22	1.13	1.42	-0.03	0.62	0.69	0.36	0.87	-0.02	0.05	0.28	0.14	0.51	-0.02	0.05
	2015	1.35	1.34	1.42	-0.03	0.18	0.78	0.36	0.83	-0.01	0.04	0.31	0.23	0.45	-0.01	0.04
	2016	1.27	1.26	1.37	-0.06	0.40	0.76	0.62	0.90	-0.03	0.05	0.47	0.27	0.60	-0.02	0.11
	2017	1.09	0.85	1.42	-0.14	0.62	0.74	0.49	0.85	-0.03	0.08	0.38	0.29	0.48	-0.03	0.06
	2018	0.78	0.13	1.37	-0.12	0.61	0.68	0.42	0.78	-0.04	0.08	0.38	0.19	0.45	-0.01	0.06
	2019	1.35	1.25	1.36	-0.05	0.37	0.79	0.64	0.98	-0.04	0.08	0.44	0.26	0.66	-0.03	0.13
	2020	0.78	1.22	1.37	-0.06	0.60	0.81	0.48	0.90	-0.06	0.09	0.32	0.24	0.47	-0.04	0.14
C US (CENSARA)	2011	1.33	1.24	1.28	-0.01	0.01	0.90	0.81	1.00	-0.01	0.02	1.43	1.37	1.49	-0.06	0.09
	2012	1.36	1.31	1.39	-0.02	0.07	0.96	0.79	1.02	0.00	0.01	0.74	0.59	1.00	-0.02	0.03
	2013	1.35	1.31	1.36	-0.01	0.02	1.06	1.01	1.13	0.00	0.01	0.78	0.52	1.24	-0.02	0.07
	2014	1.36	1.33	1.35	-0.01	0.03	1.13	1.03	1.20	0.00	0.01	0.54	0.36	0.82	-0.02	0.04
	2015	1.33	1.31	1.37	-0.01	0.05	1.06	0.93	1.16	0.00	0.01	0.41	0.26	1.04	-0.01	0.07
	2016	1.34	1.29	1.36	-0.04	0.05	1.07	0.84	1.18	0.00	0.02	0.50	0.31	1.07	-0.03	0.07
	2017	1.36	1.31	1.41	-0.02	0.11	0.97	0.77	1.16	-0.01	0.02	0.41	0.19	0.68	-0.01	0.06
	2018	1.31	1.28	1.37	-0.01	0.02	1.13	0.91	1.21	0.00	0.02	0.37	0.23	1.15	-0.03	0.10
	2019	1.34	1.28	1.37	-0.02	0.04	1.11	0.84	1.23	0.00	0.04	0.53	0.15	1.17	-0.02	0.14
	2020	1.29	1.26	1.37	-0.06	0.10	1.06	0.75	1.22	-0.01	0.05	0.38	0.12	0.88	-0.01	0.11
MW US (LADCO)	2011	1.36	0.79	1.13	-0.03	0.06	0.88	0.76	1.03	-0.03	0.05	2.72	1.91	3.23	-0.04	0.11
	2012	1.39	1.25	1.32	-0.01	0.01	1.14	1.10	1.20	0.00	0.01	1.46	1.13	1.89	-0.05	0.08
	2013	1.38	1.27	1.33	0.00	0.01	1.20	1.18	1.25	0.00	0.00	1.33	1.29	2.23	-0.06	0.09
	2014	1.38	1.15	1.33	0.00	0.04	1.21	1.11	1.25	0.00	0.03	1.81	1.34	3.01	-0.09	0.11
	2015	1.39	1.10	1.32	0.00	0.05	1.17	0.99	1.20	0.00	0.02	2.07	1.25	3.20	-0.10	0.17
	2016	1.40	1.17	1.32	-0.01	0.04	1.15	1.05	1.20	0.00	0.02	2.15	1.33	2.60	-0.08	0.12
	2017	1.40	1.26	1.35	-0.03	0.08	1.15	1.12	1.20	0.00	0.01	1.20	0.63	1.73	-0.05	0.10
	2018	1.39	1.23	1.33	0.00	0.02	1.22	1.16	1.24	0.00	0.01	1.65	0.65	2.69	-0.06	0.17
	2019	1.40	1.22	1.33	-0.01	0.05	1.20	1.13	1.23	0.00	0.03	1.54	0.55	2.47	-0.09	0.22
	2020	1.39	1.20	1.33	-0.02	0.05	1.17	1.10	1.21	0.00	0.03	1.46	0.68	2.33	-0.11	0.21
NE US (MANE-VU)	2011	1.30	0.46	0.87	-0.04	0.06	0.46	0.39	0.77	-0.03	0.05	3.09	2.70	3.25	-0.08	0.17
	2012	1.34	0.88	1.09	-0.02	0.04	0.80	0.70	0.85	-0.02	0.03	2.45	2.22	3.14	-0.06	0.07
	2013	1.34	0.97	1.24	-0.02	0.05	0.97	0.85	1.10	-0.02	0.04	2.34	2.06	3.24	-0.09	0.16
	2014	1.35	0.96	1.13	-0.05	0.07	0.95	0.79	0.99	-0.05	0.06	3.04	2.78	3.19	-0.12	0.17
	2015	1.35	0.80	1.16	-0.05	0.08	0.84	0.70	0.99	-0.04	0.07	2.80	2.43	3.22	-0.10	0.17
	2016	1.36	0.98	1.16	-0.05	0.07	0.89	0.78	0.97	-0.03	0.06	2.50	2.18	2.99	-0.09	0.14
	2017	1.37	1.13	1.25	-0.02	0.04	0.99	0.91	1.03	-0.02	0.03	2.06	1.68	2.67	-0.10	0.15
	2018	1.35	1.02	1.26	-0.03	0.08	1.06	0.95	1.13	-0.02	0.07	2.75	1.44	3.29	-0.16	0.22
	2019	1.36	1.08	1.29	-0.05	0.10	1.02	0.96	1.13	-0.03	0.09	1.98	1.21	2.82	-0.17	0.32
	2020	1.34	0.98	1.24	-0.07	0.10	0.87	0.85	0.99	-0.04	0.09	2.39	1.35	2.74	-0.19	0.28
SE US (SESARM)	2011	1.32	0.50	1.17	-0.03	0.18	0.63	0.57	0.69	-0.03	0.14	1.22	0.39	1.74	-0.08	0.12
	2012	1.34	0.98	1.24	-0.01	0.06	0.77	0.58	0.80	-0.01	0.03	1.14	0.68	1.47	-0.04	0.11
	2013	1.34	1.11	1.27	-0.01	0.03	0.83	0.76	0.90	-0.01	0.03	1.33	0.57	2.02	-0.06	0.07
	2014	1.34	1.04	1.26	-0.02	0.05	0.86	0.76	0.95	-0.02	0.04	1.15	0.62	2.23	-0.06	0.08
	2015	1.34	1.05	1.24	-0.03	0.05	0.80	0.68	0.89	-0.02	0.04	1.67	0.82	2.24	-0.05	0.10
	2016	1.35	1.02	1.27	-0.03	0.07	0.70	0.66	0.90	-0.02	0.04	1.30	0.79	1.90	-0.05	0.09
	2017	1.36	1.18	1.30	-0.03	0.08	0.77	0.66	0.90	-0.01	0.03	1.00	0.43	1.47	-0.04	0.07
	2018	1.35	1.08	1.28	-0.03	0.07	0.84	0.74	0.96	-0.02	0.05	1.36	0.57	2.15	-0.11	0.15
	2019	1.35	1.15	1.30	-0.05	0.11	0.71	0.68	0.89	-0.03	0.06	1.18	0.52	1.55	-0.08	0.19
	2020	1.33	1.05	1.23	-0.06	0.11	0.69	0.58	0.83	-0.04	0.08	1.36	0.61	1.78	-0.08	0.17

Supplementary Table 20. Simulated winter $\Delta c_{SIA}/\Delta c_{SO_4^{2-}}$, $\Delta c_{SIA}/\Delta c_{NO_3^T}$, and $\Delta c_{SIA}/\Delta c_{NH_4^T}$ with 70% reductions. "W US", "C US", "MW US", "NE US", "SE US" stand for the Western, Central, Midwestern, Northeastern, Southeastern US, respectively. The 25th and 75th percentiles (perc) of values from the sites within the corresponding region show their regional variability. Uncertainty lower and upper bounds (Unc LB and Unc UB) are the mean uncertainties (95% CI defined as the 2.5th and the 97.5th percentiles) of the sites within the region. Uncertainties of each site are estimated using 500 Monte Carlo simulations based on the precisions and detection limits shown in Supplementary Table 7.

		$\Delta c_{SIA}/\Delta c_{SO_4^{2-}}$ (70%)					$\Delta c_{SIA}/\Delta c_{NO_3^T}$ (70%)					$\Delta c_{SIA}/\Delta c_{NH_4^T}$ (70%)				
**Region**	**Year**	**Median**	**25th perc**	**75th perc**	**Unc LB**	**Unc UB**	**Median**	**25th perc**	**75th perc**	**Unc LB**	**Unc UB**	**Median**	**25th perc**	**75th perc**	**Unc LB**	**Unc UB**
W US (WRAP)	2011	1.18	0.92	1.28	-0.26	0.47	0.54	0.50	0.67	-0.01	0.05	0.92	0.66	1.17	-0.04	0.08
	2012	1.11	0.22	1.17	-0.06	0.52	0.42	0.35	0.89	-0.01	0.03	0.54	0.35	0.86	-0.02	0.05
	2013	1.41	1.06	1.43	-0.01	0.51	0.79	0.51	0.96	-0.01	0.02	0.53	0.38	0.72	-0.02	0.04
	2014	0.79	0.15	1.41	-0.02	0.35	0.69	0.34	0.84	-0.02	0.04	0.48	0.21	0.66	-0.02	0.05
	2015	1.38	1.17	1.42	-0.03	0.27	0.77	0.35	0.81	-0.01	0.04	0.42	0.35	0.73	-0.01	0.05
	2016	1.15	0.45	1.29	-0.04	0.16	0.76	0.61	0.89	-0.02	0.05	0.79	0.49	0.91	-0.03	0.09
	2017	0.66	-0.43	1.28	-0.08	0.34	0.73	0.47	0.84	-0.02	0.08	0.66	0.45	0.73	-0.04	0.06
	2018	0.69	-0.58	1.19	-0.11	0.30	0.68	0.42	0.76	-0.04	0.08	0.51	0.30	0.67	-0.02	0.06
	2019	1.16	0.31	1.32	-0.07	0.21	0.78	0.63	0.98	-0.04	0.07	0.64	0.47	0.94	-0.03	0.14
	2020	0.80	0.10	1.17	-0.08	0.29	0.80	0.47	0.90	-0.05	0.09	0.48	0.35	0.73	-0.05	0.15
C US (CENSARA)	2011	1.28	1.26	1.30	-0.01	0.01	0.92	0.82	1.01	-0.01	0.02	1.86	1.71	2.01	-0.03	0.04
	2012	1.26	1.19	1.26	-0.06	0.10	0.95	0.80	1.01	0.00	0.01	1.31	1.19	1.36	-0.02	0.03
	2013	1.32	1.31	1.34	-0.02	0.04	1.05	1.01	1.12	0.00	0.01	1.67	1.39	1.81	-0.03	0.03
	2014	1.30	1.28	1.32	-0.02	0.03	1.12	1.03	1.20	0.00	0.00	1.38	1.08	1.54	-0.03	0.04
	2015	1.31	1.29	1.36	-0.03	0.05	1.05	0.93	1.16	0.00	0.01	1.05	0.96	1.53	-0.03	0.04
	2016	1.27	1.24	1.28	-0.02	0.04	1.07	0.85	1.18	0.00	0.01	1.22	0.88	1.51	-0.02	0.04
	2017	1.24	1.16	1.27	-0.03	0.08	0.96	0.78	1.15	0.00	0.02	0.87	0.54	1.05	-0.02	0.06
	2018	1.37	1.27	1.38	-0.04	0.05	1.12	0.91	1.20	0.00	0.02	1.09	0.99	1.62	-0.05	0.09
	2019	1.37	1.30	1.39	-0.03	0.04	1.11	0.85	1.22	0.00	0.04	1.39	0.38	1.56	-0.02	0.08
	2020	1.17	1.06	1.26	-0.04	0.12	1.05	0.76	1.21	0.00	0.04	0.97	0.43	1.10	-0.03	0.10
MW US (LADCO)	2011	1.10	0.99	1.19	-0.03	0.05	1.00	0.93	1.08	-0.02	0.03	2.61	1.94	3.28	-0.06	0.08
	2012	1.32	1.28	1.33	-0.01	0.01	1.16	1.11	1.20	0.00	0.01	2.32	2.07	2.60	-0.04	0.05
	2013	1.32	1.29	1.33	0.00	0.01	1.21	1.18	1.25	0.00	0.00	2.42	2.30	2.93	-0.04	0.06
	2014	1.32	1.22	1.34	0.00	0.03	1.22	1.16	1.26	0.00	0.01	2.71	2.53	3.34	-0.05	0.07
	2015	1.28	1.18	1.33	-0.01	0.03	1.18	1.08	1.20	0.00	0.02	2.77	2.33	3.19	-0.06	0.10
	2016	1.27	1.22	1.33	-0.01	0.03	1.17	1.10	1.21	0.00	0.01	2.83	2.45	3.14	-0.05	0.07
	2017	1.28	1.21	1.30	-0.02	0.06	1.17	1.12	1.20	0.00	0.01	2.10	1.55	2.61	-0.05	0.07
	2018	1.31	1.25	1.33	-0.01	0.02	1.22	1.19	1.24	0.00	0.01	2.73	1.91	3.24	-0.07	0.10
	2019	1.31	1.25	1.33	-0.01	0.03	1.20	1.16	1.24	0.00	0.02	2.54	1.90	3.08	-0.08	0.14
	2020	1.30	1.22	1.33	-0.02	0.08	1.17	1.11	1.22	0.00	0.02	2.36	1.75	3.00	-0.09	0.14
NE US (MANE-VU)	2011	0.83	0.70	1.01	-0.03	0.05	0.62	0.54	0.88	-0.03	0.05	2.56	2.51	2.81	-0.05	0.11
	2012	1.12	1.01	1.16	-0.02	0.03	0.88	0.81	0.91	-0.02	0.03	2.49	2.29	2.76	-0.03	0.04
	2013	1.24	1.08	1.27	-0.02	0.03	1.03	0.93	1.12	-0.02	0.03	2.74	2.44	2.93	-0.04	0.06
	2014	1.14	1.08	1.20	-0.03	0.05	1.02	0.90	1.06	-0.03	0.04	3.00	2.72	3.21	-0.05	0.08
	2015	1.07	0.97	1.22	-0.03	0.06	0.94	0.82	1.05	-0.03	0.05	2.90	2.45	3.08	-0.06	0.10
	2016	1.17	1.03	1.20	-0.04	0.05	0.94	0.87	1.01	-0.02	0.05	2.78	2.40	3.00	-0.06	0.09
	2017	1.22	1.18	1.25	-0.03	0.05	1.03	0.95	1.07	-0.01	0.02	2.60	2.16	2.86	-0.07	0.09
	2018	1.18	1.08	1.27	-0.02	0.06	1.09	1.00	1.16	-0.02	0.05	3.13	2.43	3.43	-0.10	0.15
	2019	1.22	1.14	1.29	-0.04	0.08	1.05	0.99	1.14	-0.03	0.06	2.63	2.10	3.05	-0.10	0.21
	2020	1.14	1.04	1.20	-0.07	0.08	0.93	0.88	1.02	-0.04	0.08	2.63	1.88	2.92	-0.10	0.21
SE US (SESARM)	2011	1.07	0.82	1.18	-0.03	0.16	0.66	0.63	0.70	-0.03	0.12	1.35	0.78	1.71	-0.06	0.13
	2012	1.23	1.12	1.28	-0.01	0.08	0.77	0.62	0.85	-0.01	0.03	1.45	0.93	1.69	-0.03	0.12
	2013	1.22	1.17	1.29	-0.01	0.03	0.87	0.79	0.91	-0.01	0.02	1.52	0.91	2.04	-0.03	0.08
	2014	1.19	1.11	1.28	-0.03	0.05	0.86	0.83	0.97	-0.01	0.03	1.41	0.87	1.99	-0.04	0.10
	2015	1.17	1.11	1.23	-0.02	0.04	0.85	0.70	0.94	-0.01	0.04	1.78	1.11	2.33	-0.04	0.07
	2016	1.24	1.08	1.29	-0.03	0.08	0.78	0.68	0.92	-0.02	0.04	1.58	1.14	2.05	-0.04	0.08
	2017	1.26	1.06	1.31	-0.06	0.13	0.78	0.67	0.91	-0.01	0.02	1.30	0.69	1.74	-0.06	0.11
	2018	1.20	0.98	1.28	-0.04	0.13	0.87	0.78	0.96	-0.02	0.04	1.99	1.03	2.37	-0.07	0.12
	2019	1.26	1.11	1.30	-0.06	0.14	0.72	0.68	0.88	-0.03	0.06	1.43	0.75	1.86	-0.09	0.13
	2020	1.13	0.94	1.22	-0.09	0.14	0.72	0.62	0.87	-0.03	0.07	1.64	0.85	1.92	-0.08	0.12

Supplementary References

1 McClure, C. D. & Jaffe, D. A. US particulate matter air quality improves except in wildfire-prone areas. *Proceedings of the National Academy of Sciences* **115**, 7901-7906 (2018).

2 Foley, K. M. *et al.* 2002–2017 anthropogenic emissions data for air quality modeling over the United States. *Data in Brief* **47**, 109022 (2023). https://doi.org/10.1016/j.dib.2023.109022

3 Hand, J., Prenni, A., Copeland, S., Schichtel, B. & Malm, W. Thirty years of the Clean Air Act Amendments: Impacts on haze in remote regions of the United States (1990–2018). *Atmospheric Environment* **243**, 117865 (2020).

4 Paulot, F., Fan, S. & Horowitz, L. Contrasting seasonal responses of sulfate aerosols to declining SO2 emissions in the Eastern US: Implications for the efficacy of SO_2 emission controls. *Geophysical Research Letters* **44**, 455-464 (2017).

5 Pye, H. O. *et al.* The acidity of atmospheric particles and clouds. *Atmospheric Chemistry and Physics* **20**, 4809-4888 (2020).

6 Liu, T., Clegg, S. L. & Abbatt, J. P. Fast oxidation of sulfur dioxide by hydrogen peroxide in deliquesced aerosol particles. *Proceedings of the National Academy of Sciences* **117**, 1354-1359 (2020).

7 Jiang, Z. *et al.* Unexpected slowdown of US pollutant emission reduction in the past decade. *Proceedings of the National Academy of Sciences* **115**, 5099-5104 (2018).

8 Duncan, B. N. *et al.* A space-based, high-resolution view of notable changes in urban NOx pollution around the world (2005–2014). *Journal of Geophysical Research: Atmospheres* **121**, 976-996 (2016).

9 Dix, B. *et al.* Nitrogen oxide emissions from US oil and gas production: Recent trends and source attribution. *Geophysical Research Letters* **47**, e2019GL085866 (2020).

10 Womack, C. C. *et al.* An Odd Oxygen Framework for Wintertime Ammonium Nitrate Aerosol Pollution in Urban Areas: NO_x and VOC Control as Mitigation Strategies. *Geophysical Research Letters* **46**, 4971-4979 (2019). https://doi.org/10.1029/2019GL082028

11 National Atmospheric Deposition Program. (ed National Atmospheric Deposition Program) (2023).

12 Schwede, D. B. & Lear, G. G. A novel hybrid approach for estimating total deposition in the United States. *Atmospheric Environment* **92**, 207-220 (2014).

13 US EPA. (ed US EPA) (2021).

14 Appel, K. W. *et al.* Description and evaluation of the Community Multiscale Air Quality (CMAQ) modeling system version 5.1. *Geoscientific Model Development* **10**, 1703-1732 (2017).

15 US EPA. in *Air Pollutant Emissions Trends Data Air Pollutant Emissions Trends Data* (2023).

16 Pleim, J. E., Ran, L., Appel, W., Shephard, M. W. & Cady-Pereira, K. New bidirectional ammonia flux model in an air quality model coupled with an agricultural model. *Journal of Advances in Modeling Earth Systems* **11**, 2934-2957 (2019).

17 Walker, J. T. *et al.* Aspects of uncertainty in total reactive nitrogen deposition estimates for North American critical load applications. *Science of the Total Environment* **690**, 1005-1018 (2019).

18 Sen, P. K. Estimates of the regression coefficient based on Kendall's tau. *Journal of the American Statistical Association* **63**, 1379-1389 (1968).

19 Wang, R. *et al.* Monthly patterns of ammonia over the contiguous United States at 2-km resolution. *Geophysical Research Letters* **48**, e2020GL090579 (2021).

20 Nenes, A., Pandis, S. N., Weber, R. J. & Russell, A. Aerosol pH and liquid water content determine when particulate matter is sensitive to ammonia and nitrate availability. *Atmospheric Chemistry and Physics* **20**, 3249-3258 (2020).

21 Nenes, A. *et al.* Aerosol acidity and liquid water content regulate the dry deposition of inorganic reactive nitrogen. *Atmospheric Chemistry and Physics* **21**, 6023-6033 (2021).

22 Helsel, D. R. & Frans, L. M. Regional Kendall test for trend. *Environmental Science & Technology* **40**, 4066-4073 (2006).

23 Zhang, L. *et al.* Nitrogen deposition to the United States: distribution, sources, and processes. *Atmospheric Chemistry and Physics* **12**, 4539-4554 (2012).
24 Luo, G., Yu, F. & Moch, J. M. Further improvement of wet process treatments in GEOS-Chem v12. 6.0: impact on global distributions of aerosols and aerosol precursors. *Geoscientific Model Development* **13**, 2879-2903 (2020).
25 Yahya, K., Wang, K., Gudoshava, M., Glotfelty, T. & Zhang, Y. Application of WRF/Chem over North America under the AQMEII Phase 2: Part I. Comprehensive evaluation of 2006 simulation. *Atmospheric Environment* **115**, 733-755 (2015).
26 Tessum, C., Hill, J. & Marshall, J. Twelve-month, 12 km resolution North American WRF-Chem v3. 4 air quality simulation: performance evaluation. *Geoscientific Model Development* **8**, 957-973 (2015).
27 Chen, Y., Shen, H. & Russell, A. G. Current and Future Responses of Aerosol pH and Composition in the U.S. to Declining SO2 Emissions and Increasing NH3 Emissions. *Environmental Science & Technology* **53**, 9646-9655 (2019). https://doi.org/10.1021/acs.est.9b02005
28 Center for International Earth Science Information Network (CIESIN). in *Revision 11* (ed Columbia University) (NASA Socioeconomic Data and Applications Center (SEDAC), Palisades, New York, 2018).
29 Pinder, R. W., Adams, P. J. & Pandis, S. N. (ACS Publications, 2007).
30 Tsimpidi, A. P., Karydis, V. A. & Pandis, S. N. Response of fine particulate matter to emission changes of oxides of nitrogen and anthropogenic volatile organic compounds in the Eastern United States. *Journal of the Air & Waste Management Association* **58**, 1463-1473 (2008).
31 Schiferl, L. D. *et al.* An investigation of ammonia and inorganic particulate matter in California during the CalNex campaign. *Journal of Geophysical Research: Atmospheres* **119**, 1883-1902 (2014).
32 Lee, C. J. *et al.* Response of global particulate-matter-related mortality to changes in local precursor emissions. *Environmental Science & Technology* **49**, 4335-4344 (2015).
33 Lelieveld, J., Evans, J. S., Fnais, M., Giannadaki, D. & Pozzer, A. The contribution of outdoor air pollution sources to premature mortality on a global scale. *Nature* **525**, 367-371 (2015).
34 Bauer, S. E., Tsigaridis, K. & Miller, R. Significant atmospheric aerosol pollution caused by world food cultivation. *Geophysical Research Letters* **43**, 5394-5400 (2016).
35 Guo, H. *et al.* Effectiveness of ammonia reduction on control of fine particle nitrate. *Atmospheric Chemistry and Physics* **18**, 12241-12256 (2018).
36 Gu, B. *et al.* Abating ammonia is more cost-effective than nitrogen oxides for mitigating $PM_{2.5}$ air pollution. *Science* **374**, 758-762 (2021). https://doi.org/10.1126/science.abf8623
37 Henze, D. K., Seinfeld, J. H. & Shindell, D. T. Inverse modeling and mapping US air quality influences of inorganic PM 2.5 precursor emissions using the adjoint of GEOS-Chem. *Atmospheric Chemistry and Physics* **9**, 5877-5903 (2009).
38 Fann, N., Fulcher, C. M. & Hubbell, B. J. The influence of location, source, and emission type in estimates of the human health benefits of reducing a ton of air pollution. *Air Quality, Atmosphere & Health* **2**, 169-176 (2009).
39 Holt, J., Selin, N. E. & Solomon, S. Changes in inorganic fine particulate matter sensitivities to precursors due to large-scale US emissions reductions. *Environmental Science & Technology* **49**, 4834-4841 (2015).
40 Heo, J., Adams, P. J. & Gao, H. O. Public health costs of primary PM2. 5 and inorganic PM2. 5 precursor emissions in the United States. *Environmental Science & Technology* **50**, 6061-6070 (2016).
41 Dedoussi, I. C., Eastham, S. D., Monier, E. & Barrett, S. R. H. Premature mortality related to United States cross-state air pollution. *Nature* **578**, 261-265 (2020). https://doi.org/10.1038/s41586-020-1983-8

42 Stanier, C. *et al.* Overview of the LADCO winter nitrate study: hourly ammonia, nitric acid and PM 2.5 composition at an urban and rural site pair during $PM_{2.5}$ episodes in the US Great Lakes region. *Atmospheric Chemistry and Physics* **12**, 11037-11056 (2012).
43 Brewer, P. & Moore, T. Source contributions to visibility impairment in the southeastern and western United States. *Journal of the Air & Waste Management Association* **59**, 1070-1081 (2009).
44 Chen, J. *et al.* Seasonal Modeling of $PM_{2.5}$ in California's San Joaquin Valley. *Atmospheric Environment* **92**, 182-190 (2014).
45 Cheng, B. & Wang-Li, L. Responses of Secondary Inorganic PM2.5 to Precursor Gases in an Ammonia Abundant Area In North Carolina. *Aerosol and Air Quality Research* **19**, 1126-1138 (2019). https://doi.org/10.4209/aaqr.2018.10.0384
