# Supplementary material for: Regime shift in secondary inorganic aerosol formation and nitrogen deposition in the rural United States
Source: Nat Geosci. 2024 Jun 20;17(7):617–23. doi: 10.1038/s41561-024-01455-9 (PMC11245397; doi:10.1038/s41561-024-01455-9)
Supplement: Supplementary file 2 — Reporting Summary [file 41561_2024_1455_MOESM2_ESM.pdf]

nature portfolio

Corresponding author(s): Da Pan
Mark A. Zondlo

Last updated by author(s): 03/18/2024

Reporting Summary

Nature Portfolio wishes to improve the reproducibility of the work that we publish. This form provides structure for consistency and transparency in reporting. For further information on Nature Portfolio policies, see our Editorial Policies and the Editorial Policy Checklist.

Statistics

For all statistical analyses, confirm that the following items are present in the figure legend, table legend, main text, or Methods section.

n/a	Confirmed	
☐	☒	The exact sample size (*n*) for each experimental group/condition, given as a discrete number and unit of measurement
☐	☒	A statement on whether measurements were taken from distinct samples or whether the same sample was measured repeatedly
☐	☒	The statistical test(s) used AND whether they are one- or two-sided<br>*Only common tests should be described solely by name; describe more complex techniques in the Methods section.*
☐	☒	A description of all covariates tested
☒	☐	A description of any assumptions or corrections, such as tests of normality and adjustment for multiple comparisons
☐	☒	A full description of the statistical parameters including central tendency (e.g. means) or other basic estimates (e.g. regression coefficient) AND variation (e.g. standard deviation) or associated estimates of uncertainty (e.g. confidence intervals)
☐	☒	For null hypothesis testing, the test statistic (e.g. *F*, *t*, *r*) with confidence intervals, effect sizes, degrees of freedom and *P* value noted<br>*Give P values as exact values whenever suitable.*
☒	☐	For Bayesian analysis, information on the choice of priors and Markov chain Monte Carlo settings
☒	☐	For hierarchical and complex designs, identification of the appropriate level for tests and full reporting of outcomes
☐	☒	Estimates of effect sizes (e.g. Cohen's *d*, Pearson's *r*), indicating how they were calculated

Our web collection on statistics for biologists contains articles on many of the points above.

Software and code

Policy information about availability of computer code

Data collection	We did not use specific software to collect data in this study.
Data analysis	ISORROPIA II (v2.3) developed by Georgia Institute of Technology was used for Scripts and packages in Python 3 (v3.9.13) were used for data analysis. Packages used in this study include Numpy (v1.21.5), Scipy (v1.9.1), Pandas (v1.4.4), matplotlib (v3.5.2), pymannkendall (v1.4.2), and Cartopy (0.22.0).

For manuscripts utilizing custom algorithms or software that are central to the research but not yet described in published literature, software must be made available to editors and reviewers. We strongly encourage code deposition in a community repository (e.g. GitHub). See the Nature Portfolio guidelines for submitting code & software for further information.

Data

Policy information about availability of data

All manuscripts must include a data availability statement. This statement should provide the following information, where applicable:

- Accession codes, unique identifiers, or web links for publicly available datasets
- A description of any restrictions on data availability
- For clinical datasets or third party data, please ensure that the statement adheres to our policy

The integrated observation data that support the findings of this study and the source data for figures presented in the main text, Extended Data, and Supplementary Information are available in Dryad with the identifier doi:10.5061/dryad.zpc866tg3.

Research involving human participants, their data, or biological material

Policy information about studies with human participants or human data. See also policy information about sex, gender (identity/presentation), and sexual orientation and race, ethnicity and racism.

Reporting on sex and gender	Not applicable.
Reporting on race, ethnicity, or other socially relevant groupings	Not applicable.
Population characteristics	Not applicable.
Recruitment	Not applicable.
Ethics oversight	Not applicable.

Note that full information on the approval of the study protocol must also be provided in the manuscript.

Field-specific reporting

Please select the one below that is the best fit for your research. If you are not sure, read the appropriate sections before making your selection.

☐ Life sciences ☐ Behavioural & social sciences ☒ Ecological, evolutionary & environmental sciences

For a reference copy of the document with all sections, see nature.com/documents/nr-reporting-summary-flat.pdf

Ecological, evolutionary & environmental sciences study design

All studies must disclose on these points even when the disclosure is negative.

Study description	In this study, we integrated observations of concentrations of gaseous precursors, aerosol composition, and meteorological conditions from multiple monitoring networks. The integrated observations were then used for trend analyses (Mann-Kendall regional trend tests) and as inputs for aerosol thermodynamic modeling (ISORROPIA II). 1000 Monte Carlo runs were conducted to estimate uncertainties of the simulations.
Research sample	We used existing data from routine monitoring networks in the US, including CASTNET (https://www.epa.gov/castnet/download-data), IMPROVE (http://views.cira.colostate.edu/fed/DataWizard/Default.aspx), EPA CSN (http://views.cira.colostate.edu/fed/DataWizard/Default.aspx), AMoN (https://nadp.slh.wisc.edu/networks/ammonia-monitoring-network/), and NOAA ISD (https://www.ncei.noaa.gov/products/land-based-station/integrated-surface-database) as well as reanalysis meteorological data from North America Regional Reanalysis (http://nomads.ncdc.noaa.gov/#narr_datasets). The observation data were quality controlled and assured by original providers.
Sampling strategy	Sites included in this study at least had observations for atmospheric concentrations of ammonia, nitric acid, particulate ammonium, particulate nitrate, particulate sulfate, sodium, potassium, magnesium, calcium, and chloride as well as temperature and relative humidity. These are the inputs required for aerosol thermodynamic analysis. The integrated dataset is the largest of its kind to our knowledge. It covers 68 sites in the rural US. These sites are representative of regional atmospheric conditions. We focused on the rural US because there was no urban site met the requirement at the time of analysis. This is reflected in the title and discussed in the manuscript.<br>For the Monte Carlo uncertainty analysis, simulation results converge (relative standard deviation of sample mean and variance < 5%) for all sites after 700 runs. Therefore, the sample size we chose (n=1000) is sufficient.
Data collection	Data were collected by Da Pan directly from sources listed above.
Timing and spatial scale	Site selection started with AMoN sites that have monitoring sites from other networks within 50 km. The spatial window was determined by comparing observations for same variable from sites within 10 km, 25 km, 50 km, and 100 km. Since agreement of meteorological observations (T and RH) deteriorated significantly with a spatial window of 100 km, we used a 50 km spatial to achieve the largest spatial coverage while maintaining minimizing uncertainties caused by site separation. Our analysis started from 2011 since AMoN monitoring program officially started in that year. More sites with sufficient inputs for aerosol thermodynamic analysis became available in 2015. We conducted trend tests for periods of 2011 - 2015, 2016 - 2020, and 2011 - 2020 using all sites and sites established in 2011. We did not find significant differences. Thus, 2011 - 2020 results using all sites are presented in the main text and presented results of these tests in the SI.
Data exclusions	We excluded sites with only data records shorter than two years. Because annual and seasonal averages were discussed extensively in this study, we also excluded data from a site in a year if it missed or had invalid data for more than 30% of the time during any season in that year.
Reproducibility	We conducted reproducibility test by replacing observations of aerosol composition from CASTNET with IMPROVE and EPN CSN. The reproducibility test passed since the results were comparable.

Randomization	We grouped our observations into five regions based on the boundaries of Regional Planning Organizations. Randomization is irrelevant to this grouping because its purpose is to illustrate regional differences.
Blinding	Blinding was irrelevant to this study because the unique properties of each sample (i.e., observations from certain site at a given time) is critical to our analysis.

Did the study involve field work? ☐ Yes ☒ No

Reporting for specific materials, systems and methods

We require information from authors about some types of materials, experimental systems and methods used in many studies. Here, indicate whether each material, system or method listed is relevant to your study. If you are not sure if a list item applies to your research, read the appropriate section before selecting a response.

Materials & experimental systems

n/a	Involved in the study
☒	☐ Antibodies
☒	☐ Eukaryotic cell lines
☒	☐ Palaeontology and archaeology
☒	☐ Animals and other organisms
☒	☐ Clinical data
☒	☐ Dual use research of concern
☒	☐ Plants

Methods

n/a	Involved in the study
☒	☐ ChIP-seq
☒	☐ Flow cytometry
☒	☐ MRI-based neuroimaging
